# Broad snouted cladoselachian with sensory specialization at the base of modern chondrichthyans

**DOI:** 10.1186/s13358-023-00266-6

**Published:** 2023-03-28

**Authors:** Christian Klug, Michael Coates, Linda Frey, Merle Greif, Melina Jobbins, Alexander Pohle, Abdelouahed Lagnaoui, Wahiba Bel Haouz, Michal Ginter

**Affiliations:** 1 Paläontologisches Institut und Museum, University of Zurich, Karl-Schmid-Strasse 4, 8006 Zürich, Switzerland; 2 Department of Organismal Biology and Anatomy, University of Chicago, 1027 E. 57Th St., Chicago, 60637 USA; 3 Institute for Geology, Mineralogy, and Geophysics, Ruhr University Bochum, Universitätsstraβe 150, 44801 Bochum, Germany; 4 Interdisciplinary Research Laboratory in Sciences, Education and Training, Higher School of Education and Training Berrechid (ESEFB), Hassan First University, Avenue de l’Université, B.P:218, 26100 Berrechid, Morocco; 5 Laboratory of Stratigraphy of Oil-and-Gas Bearing Reservoirs, Department of Paleontology and Stratigraphy, Institute of Geology and Petroleum Technologies, Federal University, Kremlyovskaya Str. 18, 420008 Kazan, Volga Region Russia; 6 Geosciences Laboratory, Department of Geology, Faculty of Sciences Ain Chock, Hassan II University, Km 8 Route d’El Jadida, 20100 Casablanca, Morocco; 7 Faculty of Geology, University of Warsaw, Al. Żwirki I Wigury 93, 02-089 Warsaw, Poland

## Abstract

Throughout the Silurian and Devonian, cartilaginous fish successively evolved their specialized skeletal and dental characteristics, and increasingly refined their sensory systems. The Late Devonian shark taxon *Maghriboselache mohamezanei* gen. et sp. n. from the eastern Anti-Atlas of Morocco is known from multiple specimens preserving most of its skeletal features, which in some instances are preserved in three dimensions. Key details of the dentition, jaws, and pectoral skeleton are shared with the iconic genus *Cladoselache*. Phylogenetic analyses place the family Cladoselachidae as the sister group of symmoriiforms and these groups as sister group of the holocephalans. Further phylogenetic results corroborate that the initial evolutionary radiation of crown chondrichthyans occurred within or before the Late Devonian. Remarkably, this new stem holocephalan is equipped with a wide snout and large laterally separated nasal capsules: the earliest known example of this condition in the chondrichthyan and (perhaps) gnathostome record. This suggests sensory specialization approaching that of extant broad-rostrum elasmobranchs and represents a significant addition to increasingly apparent ecomorphological diversity among early chondrichthyans.

**ZooBank LSID:** urn:lsid:zoobank.org:pub:85F45912-9EBA-4061-B62B-5937180E807A.

**Supplementary Information:**

The online version contains supplementary material available at 10.1186/s13358-023-00266-6.

## Introduction

Our understanding of chondrichthyan phylogeny has been vastly improved in recent years by the addition of anatomical information from three-dimensional crania (including endocasts) and enriched sampling of postcrania (Coates & Sequeira, 2001; Coates et al., 2017; Davis et al., 2012; Frey et al., 2020a, b; Pradel et al., 2009, 2011). Emerging consensuses include the wholesale movement of acanthodians on to the chondrichthyan stem (Brazeau, 2009; Davis et al., 2012; Dearden et al., 2019; Maisey et al., 2017; Miller et al., 2003; Zhu et al., 2013), and, more contentiously, recognition of numerous Paleozoic clades as stem holocephalans (Coates et al., 2017, 2018; Dearden et al., 2019; Frey et al., 2020a, b). With this change has come increasing recognition that total group Chondrichthyes (Andreev et al. 2022; Coates et al., 2018), and thus all crown gnathostomes, originated at least as far back as the early Silurian (Andreev et al., 2022; Zhu et al., 2022). Among the stem holocephalans, the Symmoriiformes are an important group, comprising a series of bizarre species, several of which bear unusual fin spines (Coates & Sequeira, 1998, 2001; Lund, 1985, 1986; Williams, 1985; Zangerl, 1990; Zangerl & Case, 1976). However, alternative hypotheses remain current, and these posit Symmoriiformes as either stem chondrichthyans (Denton & Goolsby, 2021) or stem elasmobranchs (Hodnett et al., 2021).

*Cladoselache*, the classic primitive shark of older textbooks (e.g., Romer 1966) and occasional model for a gnathostome archetype (Jarvik 1980), is now most commonly associated with the symmoriiform grade or clade (e.g., Coates et al., 2017; Maisey, 2007). However, with successive discoveries of new taxa preserved in greater detail, *Cladoselache* is increasingly marginalized in spite of its exceptional soft-tissue preservation and completeness (Dean, 1909a, 1909b). Thus, rather than shedding light on conditions in the earliest jawed vertebrate, *Cladoselache* likely manifests conditions close to the base of the holocephalan lineage and/ or early crown chondrichthyans in general. But there is an outstanding problem: lack of three-dimensional anatomical detail. With one notable exception (Maisey, 1989), cladoselachian specimens are mostly flattened and have never been subjected to thorough, comprehensive, description. Instead, the Cleveland Shale cladoselachian specimens are mostly flattened and are reported in a multitude of shorter studies (Dean 1893, 1909a, b; Harris, 1938, a, b; Woodward & White 1938, Bendix-Almgreen, 1975, Maisey, 1989, 2007, Williams, 2001, Tomita, 2015, Jacquemin et al. 2016), and questionable plethora of species (see Ginter et al., 2010 for a review of the tooth-based criteria).

**Fig. 1:**
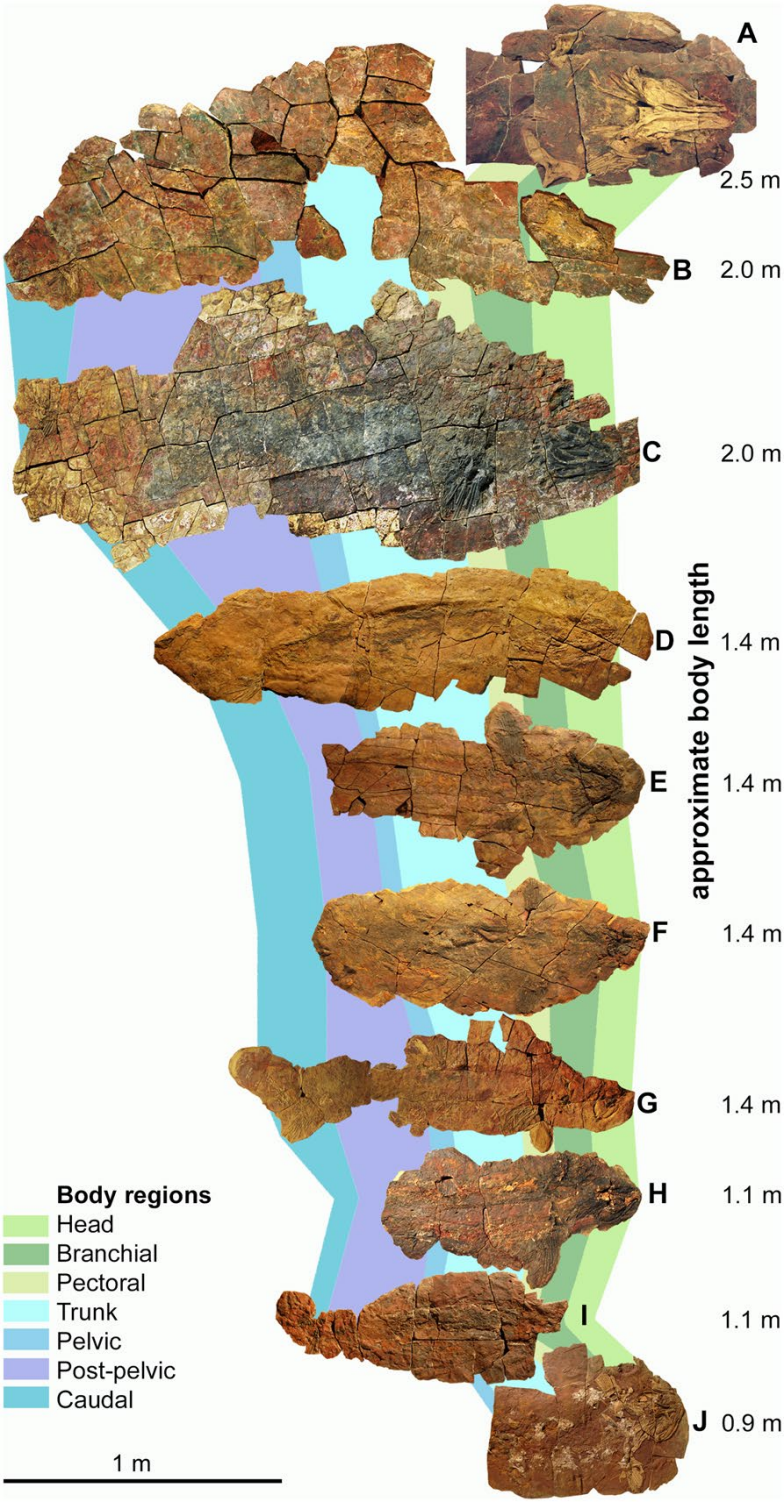
Skeletons of *Maghriboselache mohamezanei* n. gen. et sp. Famennian, eastern Anti-Atlas (for locality details, see supplement). Homologous body regions are indicated by colored fields. Only J is from the southern Tafilalt, all others are from the southern Maïder region. **A** PIMUZ A/I 5152; **B** AA.MEM.DS.12; **C**, AA.MEM.DS.6; **D** PIMUZ A/I 5153; **E** PIMUZ A/I 5154; **F** PIMUZ A/I 5155; **G** AA.BER.DS.01; **H** PIMUZ A/I 5156; **I** PIMUZ A/I 5157; **J** PIMUZ A/I 5158

Here, we report the first discovery of a likely *Cladoselache* sister-genus, represented by several individuals (Fig. 1), some of which include a three-dimensionally preserved neurocranium. These specimens, like those of other chondrichthyans from the eastern Anti-Atlas (Frey et al., 2019, 2020a; b; Greif et al., 2022), are nearly complete, nearly fully articulated and several examples include remains of the integument, musculature, digestive tract and the liver. Along with many other chondrichthyans, this material was extracted from the Thylacocephalan layer (middle Famennian) (Frey et al., 2018, 2019) of the highly fossiliferous Devonian marine sedimentary outcrops laid down in two small epicontinental basins (Frey et al., 2018, 2019; Wendt, 1985, 1988, 2021), now located in the Maïder and Tafilalt regions of the eastern Anti-Atlas of Morocco. Besides chondrichthyans, these sediments have also yielded diverse invertebrates, placoderms, acanthodian stem-chondrichthyans, and actinopterygians (Frey et al., 2018, 2019, 2020a, b; Jobbins et al., 2022).

The aims of this study are to present a detailed record of the skeletal anatomy of the new genus, and to further test hypotheses (Coates et al., 2017; Denton & Goolsby, 2021; Frey et al., 2020a, b; Maisey, 2007) concerning (a) the relationship of cladoselachians to the symmoriiform clade or grade, and (b) the relationship of symmoriiforms to primary divisions of Chondrichthyes as defined by current phylogenies. Time-calibrated trees shed new light on the chronology of evolutionary events, and the morphological data yields unexpected implications for the evolution of sensory systems in at least some of these archetypically ancient chondrichthyans.

## Material

The strata bearing the here described material (Additional file 1: Table S1 and supplementary descriptions) of *Maghriboselache* is situated in the southern parts of the Maïder Basin and of the Tafilalt Platform (eastern Anti-Atlas) of Morocco (see map in (Frey et al., 2018, 2020a, b)). The skeletal remains occur at various localities such as Bid er Ras (PIMUZ A/I 5156, 30°45`606``N, 4°55`637``W), Mousgar (AA.MEM.DS.12, 30°46`557``N, 4°41`257``W), Tizi n`Aarrat (PIMUZ A/I 5160, teeth), Oufatene (PIMUZ A/I 5153, 30°47`057``N, 4°53`476```W) and Madene (PIMUZ A/I 5152, 30°45`056``N, 4°42`829``W; PIMUZ A/I 5155, PIMUZ A/I 5155, 30°43`235``N, 4°43`568``W). Most remains were found in the Thylacocephalan Layer, which is of late early to early middle Famennian age, which measures 4 m in thickness (Frey et al. 2020a, b; Jobbins et al., 2020). It was given this name because of the great abundance of thylacocephalan arthropods.

## Methods

All specimens were prepared by airscribes and by sandblasting using aluminium oxide powder. The anterior parts of the skeletons are often preserved in ferruginous nodules of reddish color, while the posterior part is more rarely preserved. If present, the posterior part usually lies on the concretions. The overall outline of each concretion often corresponds approximately to the body outline of the preserved individual (Jobbins et al., 2022). In many skeletons, however, the caudal regions are missing, because that part was not fully encased in the nodule but surrounded by clay and thus weathered away more quickly. The cranial parts are often strongly compressed and crushed. However, in specimen AA.MEM.DS.12 and PIMUZ A/I 5159, parts of the neurocranium and endocast are preserved in three dimensions, yielding a lot of anatomical details. From these specimens, computer tomography scans were acquired using a Nikon XT H 225 ST industrial CT-scanner housed at the School of Earth Science, University of Bristol, United Kingdom and at an industrial CT-scanner at Qualitech in Mägenwil, Switzerland. AA.MEM.DS.12 was scanned in two parts to get a higher resolution. Data acquisition and image reconstruction parameters: 224 kV, 535 µA; acquisition time: 6 h for each scan; filter: 3 mm of copper; voxel sizes in mm: x = 0.085, y = 0.085, z = 0.134; total slices: 2000 in each direction; 16-bit TIFF images were acquired; 8-bit TIFF images were used for reconstruction. PIMUZ A/I 5159 was scanned with the following parameters: 520 kV, 1.3 µA; filter: aluminum; voxel sizes in mm: *x* = *Y* = *Z* = 0.0534 mm; total slices: 2430; 16-bit TIFF images were acquired; 8-bit TIFF images were used for reconstruction. Reconstruction was performed using Mimics v.17 (http://www.biomedical.materialise.com/mimics; Materialise, Leuven, Belgium) and the reconstructed 3D-object was edited (smoothing, colors and lighting) in MeshLab v. 2016 (http://www.meshlab.net; (Cignoni et al., 2008)) and blender v2.79b (https://www.blender.org; Amsterdam, Netherlands).

The data obtained from the new taxon was integrated in the data matrix of Frey et al., (2020a, b). It comprises 230 characters of both chondrichthyan and non-chondrichthyan genera. In addition, another matrix was tested using eight additional characters (details in Additional file 1 to 6). A Bayesian tip-dated analysis was carried out in BEAST 2.6.3 (Bouckaert et al., 2019) using the fossilized birth–death model (Gavryushkina et al., 2014; Heath et al., 2014; Stadler, 2010) (see Additional file 1, 2, 3, 4, 5, 6 for details).

### Systematic palaeontology

Chondrichthyes (Huxley, 1880).

Holocephali (Bonaparte, 1838).

Symmoriiformes (Zangerl, 1981).

Cladoselachidae (Dean, 1909a).

**Diagnosis:** Symmoriiform chondrichthyans with pectoral fins with distally broad, flat, strap-like radials. Cleaver-shaped palatoquadrate with otic process shorter than palatine process; jaw articulation barely posterior to occipital level. Teeth with tall median cusp flanked by much smaller lateral cusps and tooth base with a deep basolabial depression flanked by adjacent projections.

**Remarks**: This family now comprises the genera *Cladoselache* and *Maghriboselache*, thus far limited to the Famennian of the USA and Morocco.

#### *Maghriboselache *gen. n.

**Etymology**: From the Arabic word *al Maghrib* for Morocco and the Greek word σέλαχος (*selachos*) for cartilaginous fish.

**Zoobank:** LSID urn:lsid:zoobank.org:act:8F3F6DEA-D544-4AF2-AD65-99332DC36DAE.

**Type species:** *Maghriboselache mohamezanei* sp. n.

**Diagnosis:** Cladoselachiid distinguished by neurocranium with broad flattened rostrum enclosing large, widely separated, nasal capsules. Rostral span, including the nasal capsules, matches span of the postorbital arcade. Postorbital arcade surrounds small jugular foramen. Ventral extremity of postorbital arcade extends anteriorly, contributing to orbit floor and almost meeting posterolateral extremity of rostral cartilage and postnasal wall. Otic process of palatoquadrate anteroposteriorly short, in lateral view terminating level with, or just caudal to, the posterior extremity of the occipital cartilage. Dentition with enlarged teeth on the mandibular symphysis. Scapulocoracoid with ventrally broad scapula process and sturdy coracoid region with slender, distinct, procoracoid cartilage. Anterior dorsal fin spine (if present): long, laterally flattened and smooth, curved posteriorly throughout length.

#### *Maghriboselache mohamezanei* sp. n.

Figures 1, 2, 3, 4, 5, 6, 7, 8, 9, 10, 11, 12, Additional file 1: Figs. 1–40.

**Holotype:** AA.MEM.DS.12, which preserves the 3D neurocranium, teeth, shoulder girdle, and most fins, and hence most of the relevant body parts.

**Etymology**: Referring to Moha Mezane (El Khraouia & Merzouga, Morocco), French linguist and amateur geologist, specialized in fossils and minerals from the southern Tafilalt. He found many important specimens including some of the material described here.

**Zoobank:** LSID urn:lsid:zoobank.org:act:637BB65B-A77F-45DB-BCE2-383C66C46F82.

**Age:** Thylacocephalan Layer, late early to early middle Famennian, Late Devonian.

**Type locality:** Mousgar (30°46`557``N, 4°41`257``W), southern Maïder, southeastern Anti-Atlas, Morocco (see material).

**Material:** Bid er Ras: PIMUZ A/I 5156, 30°45`606``N, 4°55`637``W; Mousgar: AA.MEM.DS.8, AA.MEM.DS.12, 30°46`557``N, 4°41`257``W, Tizi n`Aarrat: PIMUZ A/I 5160, Oufatene: PIMUZ A/I 5153, 30°47`057``N, 4°53`476``W, Madene: PIMUZ A/I 5152, 30°45`056``N, 4°42`829``W; PIMUZ A/I 5155, 5159, 30°43`235``N, 4°43`568``W; PIMUZ A/I, Jebel Aoufilal: PIMUZ A/I 5158, N30°56′25.2″, W4°01′14.9″. Most remains were found in the early/middle Famennian Thylacocephalan Layer. A complete list is provided in Additional file 1: Table S1.

**Diagnosis:** As for genus.

### Description

**Fig. 2:**
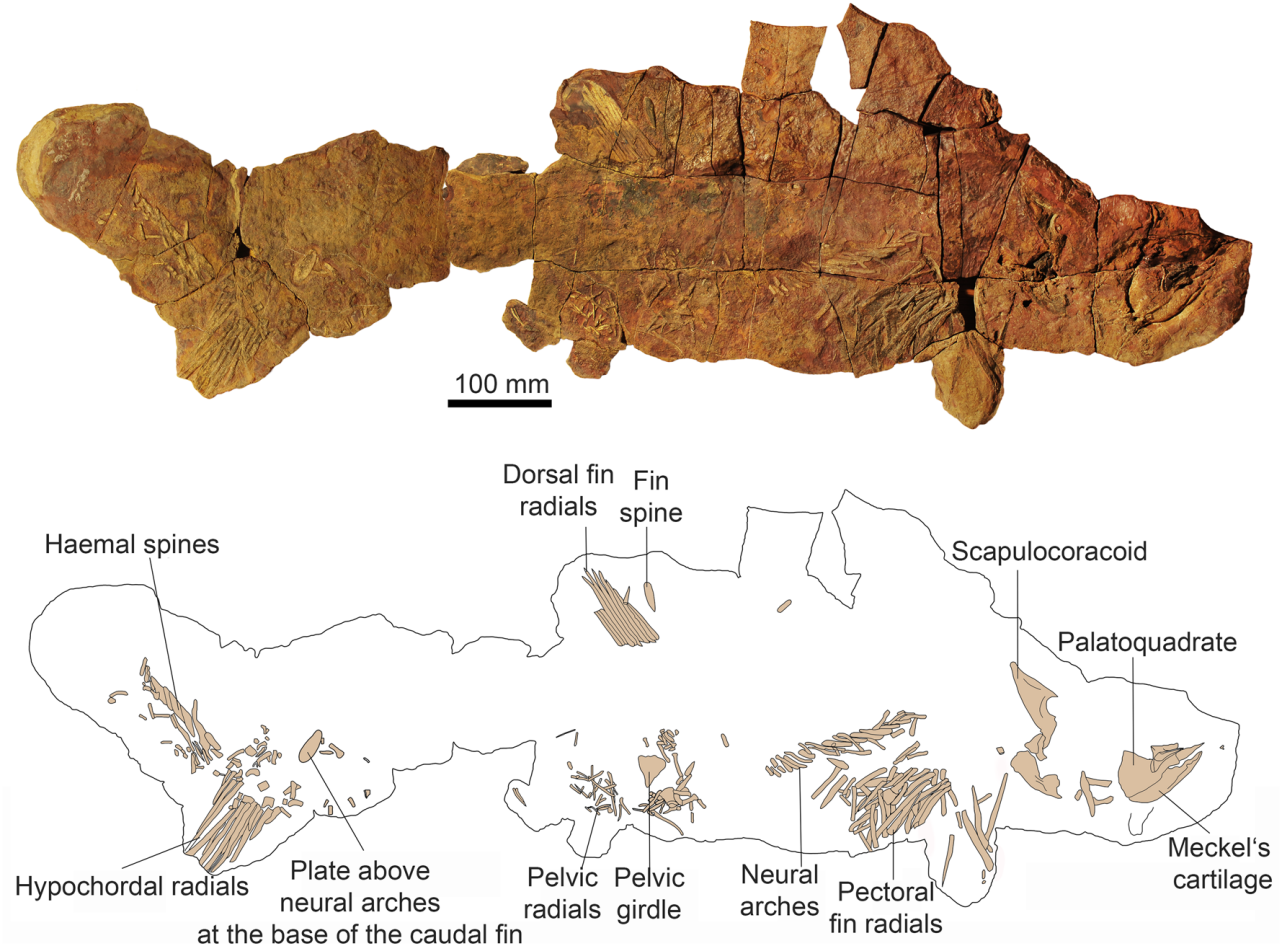
Photo and drawing of a nearly complete skeleton of *Maghriboselache mohamezanei* n. gen. et sp., AA.BER.DS.01. The marked fin spine belongs to the posterior fin

**Body form:** Estimated in-life lengths of individuals available for study range between 0.8 and 2.5 m (based on proportions measured in the more or less complete specimens AA.MEM.DS.12, AA.MEM.DS.6, PIMUZ A/I 5153 and AA.BER.DS.01; Fig. 1), with the majority being slightly over one meter long (Figs. 1, 2). The head, exclusive of the gill region, is rather short compared to, e.g., *Akmonistion*, and has a triangular appearance when flattened; in this respect, the material resembles the Cleveland Shale skeletons of *Cladoselache*. The first dorsal fin spine is present in about half of those specimens preserving more than the head (PIMUZ A/I 5153, 5154, 5158, AA.BER.DS.01). This might suggest a sexual dimorphism like that proposed for certain Carboniferous chondrichthyans (Lund, 1985), but we cannot be certain in the absence of clasper preservation.

**Fig. 3:**
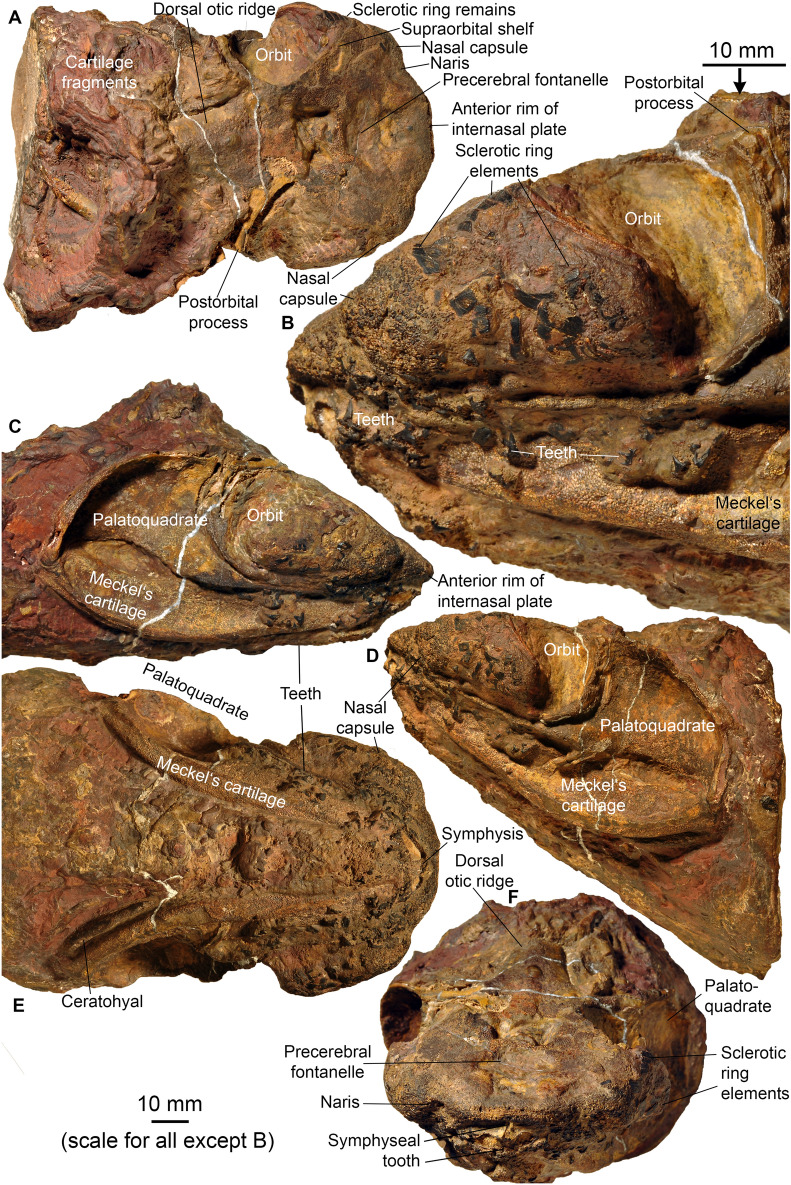
Head of *Maghriboselache mohamezanei* n. gen. et sp., PIMUZ A/I 5156 with neurocranium, jaws, hyoid arches and disarticulated gill remains.** A** dorsal posterior, **B** detail of D, **C** right lateral anterior, **D** left lateral, **E** ventral, and **F** anterior views

**Fig. 4:**
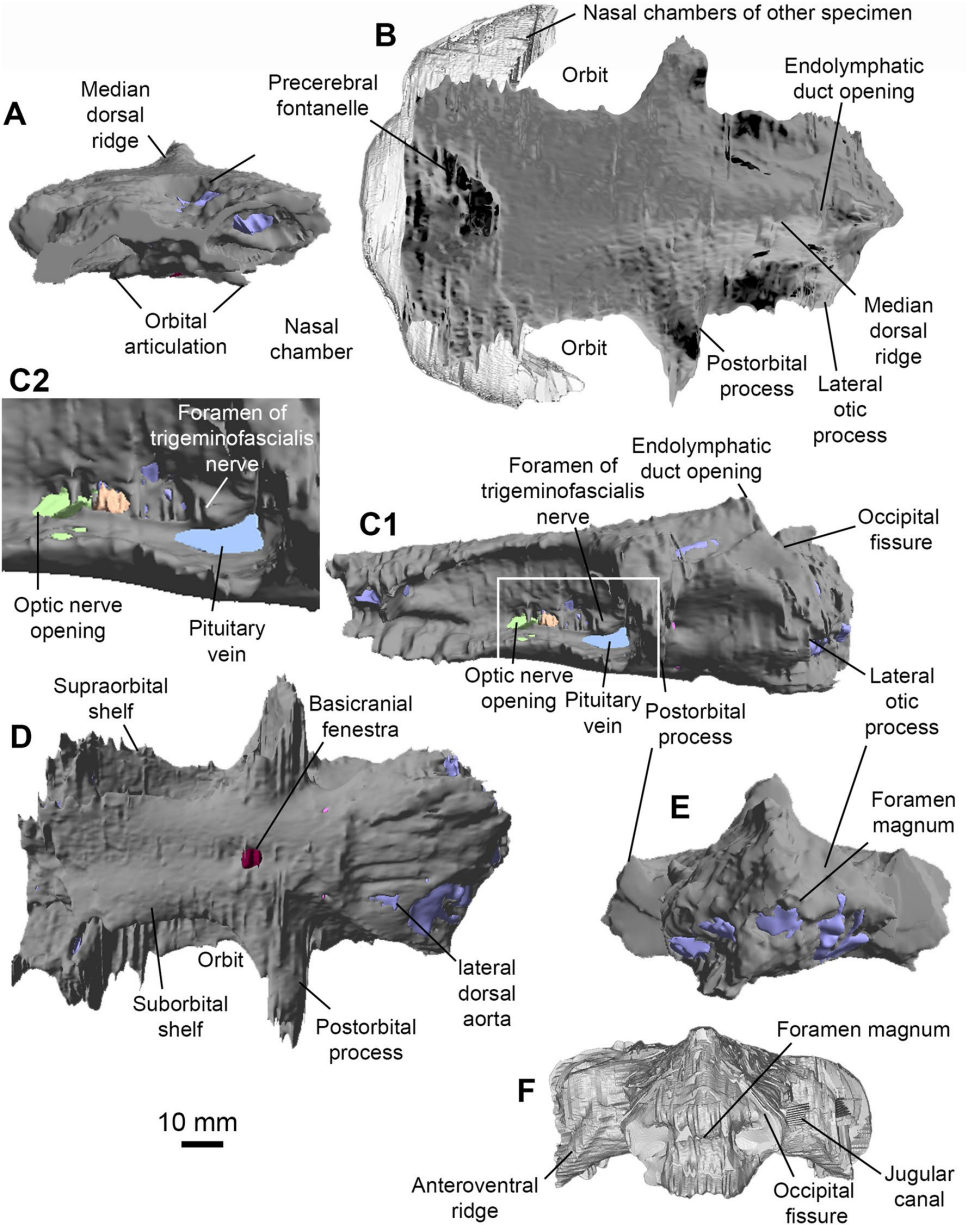
Neurocranium of *Maghriboselache mohamezanei* n. gen. et sp. **A** to** E** holotype, AA.MEM.DS.12. **A** anterior, **B** dorsal (other specimen: PIMUZ A/I 5156), **C** left lateral (C2: detail of C1, marked by rectangle in C1), **D** ventral, and **E** posterior view. **F** PIMUZ A/I 5156, posterior view

**Fig. 5:**
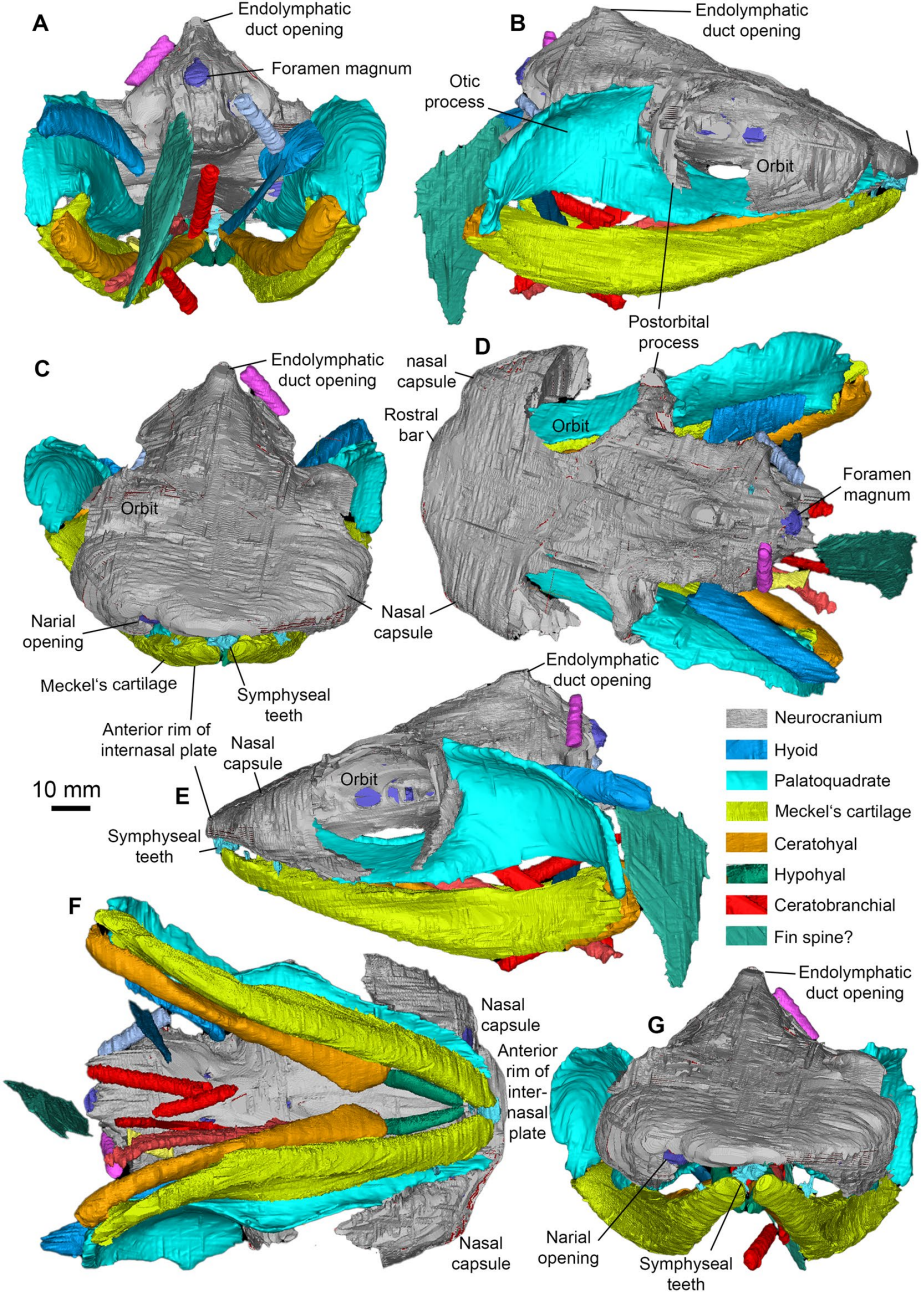
Head of *Maghriboselache mohamezanei* n. gen. et sp., PIMUZ A/I 5156 with neurocranium, jaws, hyoid arches and disarticulated gill remains.** A** posterior, **B** right lateral, **C** anterior, **D** dorsal, **E** left lateral, **F** ventral, **G** anterior views. Note the color-coded cranial elements

**Fig. 6:**
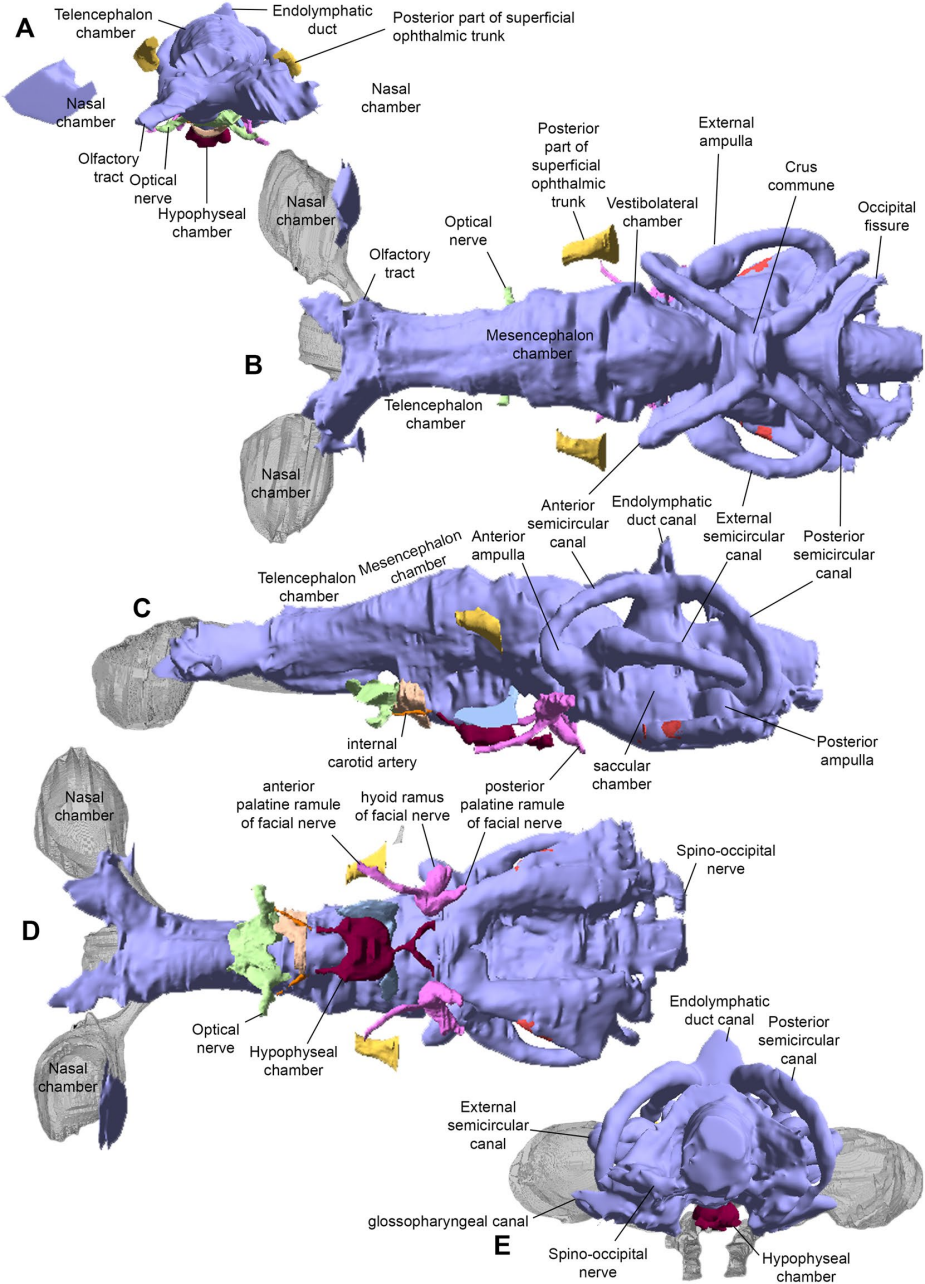
Endocast of *Maghriboselache mohamezanei* n. gen. et sp., holotype, AA.MEM.DS.12. Nasal chambers (in grey) copied from PIMUZ A/I in **A**, **B** and **D**. **A** anterior, **B** dorsal, **C** left lateral, **D** ventral, and **E** posterior views

**Neurocranium ** The neurocranium (Figs. 3, 4, 5, 6) is known from two specimens: AA.MEM.DS.12 (Figs. 1, 4, 6; see also Additional file 1: Figs. S3, S4) and PIMUZ A/I 5159 (Figs. 3, 5; see also Additional file 1: Figs. S38, S41). PIMUZ A/I 5159 exhibits a three-dimensionally preserved neurocranium measuring a total of 166.8 mm in length. The nasal capsules and postorbital processes of AA.MEM.DS.12 are incomplete, but the specimen contains a cast of the cranial cavity that reveals exquisite anatomical detail (Fig. 6). PIMUZ A/I 5159 (Figs. 3, 5; see also Additional file 1: Fig. S41) is smaller, 105 mm long and 81 mm wide, and includes a broad ethmoid cartilage enclosing widely separated nasal capsules flanking an internasal plate. The postorbital wall and process is similarly complete, and the otico-occipital region is preserved in articulation with hyoid and mandibular arches as well as scattered gill arch cartilages. CT-scans of PIMUZ A/I 5159 are less contrasted (Additional file 1: Figs. S29–S32, S35) than those of AA.MEM.DS.12.

The general shape and proportions of the neurocranium display a previously unknown combination of features: a symmoriiform core, cf. *Dwykaselachus* (Coates et al., 2017), but with ethmoid and postorbital extremities almost meeting ventrally. This near-enclosure of the orbit in a cartilage ring is otherwise known only in the New Brunswick *Doliodus* (Maisey et al., 2009), a genus of interest because of the light it might shed on conditions close to the chondrichthyan crown node (Coates et al., 2017; Maisey et al. 2018). In lateral view, PIMUZ A/I 5159 is wedge-shaped (Fig. 3C), except for the bulging nasal chambers and near-circular orbits. Around one third of orbit diameter projects ventrally relative to neurocranial (ventral) midline (Figs. 3–5, Additional file 1: Figs. S29-S32, S35). In contrast, AA.MEM.DS.12 (Fig. 4C) displays orbits with an oval outline, but this results from corrosion of its venter (including the postorbital processes) as well as a possible slight dorsoventral compression (not visible in the endocast).

The ethmoid region is exceptionally broad and walled externally with cartilage. Uniquely among early chondrichthyans, the snout is the widest part of the neurocranium; wider than the total span of the postorbital processes (Figs. 3A, 4B, 5D, G). A large precerebral fontanelle is present (visible in photographs of PIMUZ A/I 5159 but absent from CT rendering; fontanelle margins damaged in AA.MEM.DS.12: Figs. 3A, 4B), and extends posteriorly to the anterior level of the orbits. Nasal capsules are large, but capsule details are poorly resolved. Nostrils appear to be subterminal: a notch and depression on the right side of the PIMUZ A/I 5159 snout marks a plausible location (Figs. 3A, F, 5C, D, G). Furthermore, these nasal capsules appear to be anteriorly enclosed (except for narial apertures), quite unlike the anterolaterally directed cartilage cups present in other early chondrichthyans. The subnasal space identified in *Dwykaselachus* and other symmoriiforms (Coates et al., 2017) seems to be absent.

The rear wall of the ethmoid complex, anterior wall of the orbit, is strongly concave, thinning and extending posteriorly around the ventrolateral rim of the orbit (Figs. 4D, 5D). As in the New Brunswick *Doliodus* (Maisey et al., 2009), this cartilage resembles a neoselachian ectethmoid process (although homology is doubtful: these structures are very likely independently derived—see Maisey 1983 for comparison of this process in *Egertonodus* and *Chlamydoselachus*) and terminates as a prong directed towards the tip of the postorbital process. Thus, like the New Brunswick *Doliodus*, the orbit is almost completely ringed in cartilage (Figs. 4B, 5D). The ventral surface of the snout includes a distinct internasal plate, the lateral extremities of which project ventrolaterally, forming grooved attachment surfaces for the palatoquadrate (the orbital/ ethmoid articulation). The form of the grooved surface closely resembles examples in symmoriiforms (Coates & Sequeira, 2001; Coates et al., 2017) and *Cladodoides* (Maisey, 2005).

The orbits are not especially large and, in dorsal view, the roof of each is deeply embayed. The supraorbital shelf is evidently much narrower than in *Cladoselache* (Harris 1938b; Maisey, 2007), *Akmonistion* (Coates & Sequeira, 2001), and *Dwykaselachus* (Coates et al., 2017). Details within the orbit, observed in lateral aspect, are mostly obtained from specimen AA.MEM.DS.12 (Fig. 4). A broad antotic pila divides a large optic nerve foramen from a prootic fenestra (Fig. 4C). In *Pucapampella* and *Gydoselache* (Maisey et al., 2018), the pila support an optic pedicel (eye stalk) but no pedicel is evident in *Maghriboselache*. The posteromedial recess of the orbit includes the exit of branches of the facial nerve (VII), preserved in detail in AA.MEM.DS.12 (Fig. 4C). There is no interorbital septum: unlike *Dwykaselachus* and other symmoriiforms, the medial wall of each orbit is separated widely from its counterpart. The deeply concave surface of the postorbital wall is pierced by a circular foramen for the jugular (lateral head) vein close to the anteriormost portion of the otic wall; the foramen is strikingly similar to the same feature in *Dwykaselachus*. A suborbital shelf is present (Fig. 4D): narrow but slightly broader than the minimal examples in other symmoriiforms. The efferent pseudobranchial foramen is marked by a slight dimple evident in rendered scans, located level with the optic nerve foramen.

The postorbital process and wall (or arcade) of PIMUZ A/I 5159, although worn away both laterally and dorsally, is notably short in lateral extent (Fig. 5D) and quite unlike the slender, laterally elongate, examples in other symmoriiforms including *Cladoselache* (Maisey, 2007: Figs. 58, 59, 61). In *Maghriboselache*, this robust, anteriorly concave process extends ventrally to rim the orbit floor, ultimately approaching the ‘ectethmoid’ process (as noted above). Once again, a similar process is present in the New Brunswick *Doliodus* (Pradel et al., 2009), and a further example might be found in *Gladbachus* (Coates et al., 2018), which also appears to contribute to the orbit margin and floor. The posterior wall of the process in *Maghriboselache* resembles those of *Cladodoides* and *Dwykaselachus*, bearing a gently angled ridge and groove for articulation with the palatoquadrate.

Unlike the ethmosphenoid, the otico-occipital division conforms closely to that of *Dwykaselachus*, resembling a low-resolution copy of standard symmoriiform shape. The dorsal otic ridge ascends posteriorly to peak at a median, short rounded endolymphatic duct opening (Figs. 4A–C, 5A–D). The lateral otic ridge, in this instance lacking a periotic process, broadens posteriorly to surround the posterior ampulla of the otic labyrinth. There is no lateral otic process cf. *Tamiobatis* (Schaeffer, 1981): the cartilage wall of the otic region simply bulges where it forms a broad convexity that encloses the posterior ampulla. The occipital arch is short and embedded in the rear of the cranium; presence of an occipital fissure is evident from the endocast (Fig. 6B). Dorsal and ventral paroccipital processes are present, bracketing the cotylus.

The ventral surface is poorly preserved posteriorly in PIMUZ A/I 5159 and AA.MEM.DS.12. The likely division of the lateral dorsal aortae anterior to the occiput is inferred from a mid-line depression in the rendering of PIMUZ A/I 5159. Directly posterior to the level of the postorbital process, the basicranium exhibits the characteristic waist of symmoriiforms. Level with the posterior of this restriction, the basicranium shows a large, laterally directed foramen (Fig. 4D) marking the likely emergence of a lateral dorsal aorta and union with the efferent hyoid artery (cf. *Dwykaselachus* in Coates et al., 2017; “*Cobelodus*” in Maisey, 2007). Just anterior to the base of the postorbital processes, a simple, midline, basicranial fenestra marks the buccohypophyseal foramen (Fig. 4D).

**Endocast.** This description is largely based on AA.MEM.DS.12 (Fig. 6) with additions from PIMUZ A/I 5159. The overall appearance (Fig. 6) of the endocast resembles *Cladodoides* (Maisey, 2005), with a broad endocranial space running from anterior to posterior extremities. The nasal chambers (Fig. 6B–D) are similarly proportioned to those of *Dwykaselachus* (Coates et al., 2017) but with greater separation across the midline contributing to the exceptional width of the entire ethmoid region. There is no extreme narrowing of the space for the telencephalon between the orbits. Mesencephalon and vestibulolateral chambers are marked only by modest lateral expansions of the pre-otic region (Fig. 6D). The transition between telencephalon and mesencephalon spaces lacks any distinct boundary; the endocranial roof rises gently, lacking the almost step-like rise evident in *Dwykaselachus*. The mesencephalon chamber is proportionally broader than in *Cladodoides*, and both dorsal and lateral views show a shallow constriction (Fig. 6B, C) preceding the domed vestibulolateral chamber seated between the anterior semicircular canals.

Symmoriiform endocasts described thus far show a marked elevation of the endocranial floor reflecting the angle and extent of the dorsum sellae posterior to the hypophyseal space. In *Maghriboselache*, although steeper than in *Cladodoides*, the angle of inclination is much less than in *Dwykaselachus*. Similarly, the roof of the hindbrain-midbrain chamber junction is lower than the height of the otic labyrinth in *Cladodoides*, slightly higher in *Maghriboselache*, and considerably higher in *Dwykaselachus*, in which the bulk of the vestibulolateral chamber is nested above the anterior semi-circular canals. Little is known of the hindbrain region, sandwiched between the otic capsules. An occipital fissure persists, arched anteriorly between the posterior semi-circular canals (Fig. 6B). The occipital division of the braincase is short, barely projecting beyond the posterior limit of the otic capsule and including three spino-occipital nerve foramena (unlike five in *Dwykaselachus*).

The otic labyrinth (Fig. 6B–E) conforms closely to the pattern present in *Dwykaselachus* and ‘*Cobelodus’*/ *Ozarcus* except in one key respect: the anterior ampulla is ventral to the level of the external ampulla as in *Cladodoides* and outgroups (Schaeffer, 1981). In symmoriiforms and extant holocephalans, the anterior ampullae is level with or the slightly dorsal to the external ampulla. The endolymphatic duct of *Maghriboselache* is entirely separate from the occipital fissure and projects vertically level with the crus commune, in this regard consistent with *Dwykaselachus* and ‘*Cobelodus’*/ *Ozarcus*. Ventral to the external semicircular canal, the saccular chamber is visible, with the glossopharyngeal canal extending along its ventrolateral margins. Unlike *Cladodoides*, endocasts of specimen AA.MEM.DS.12, *Dwykaselachus* and *Cobelodus’*/ *Ozarcus* show no distinct pipe (in-fill) for the exit of the vagus nerve.

The ventral side of the endocast of AA.MEM.DS.12 (Fig. 6D) preserves imprints of chambers and ducts for several cranial nerves and blood vessels. The exit of the optic nerve (II) is positioned ventral to the posterior part of the telencephalon. Directly ventral to this, an infilled forked channel marks the likely entry for the efferent pseudobranchial artery. The hypophyseal chamber includes the foramen for the pituitary vein. Ventrolateral to the vestibulolateral chamber, the openings for the paired facial nerve (VII) are present. The anterior palatine branch of the facial nerve projects anteriorly, the posterior palatine branch extends posteroventrally, and the hyoid ramus extends posterodorsally.

**Mandibular and hyoid arches.** In PIMUZ A/I 5159, mandibular and hyoid arches are nearly complete and articulated (Figs. 3, 5, Additional file 1: Fig. S30, S33, S34). Notably, the jaws are much shorter relative to the neurocranium than in *Akmonistion* (Coates & Sequeira, 2001), *Ozarcus* (Pradel et al., 2014), and *Ferromirum* (Frey et al., 2020a, b), where the jaw joint is offset posteriorly, well behind the occiput. The palatoquadrate exhibits the cleaver shape (Schaeffer, 1975), which is characteristic of many early chondrichthyans; it has an otic process some 40% of total jaw length (Fig. 5B, D, E). The otic process is both concave laterally, forming a deep space to house adductor muscles, and concave anteriorly where it articulates with the postorbital process rear wall. The anterior, palatine, ramus is dorsoventrally flattened and gently concave dorsally where it contributes to the orbit floor (Additional file 1: Fig. S33). The medial rim of the ramus ethmoid extremity bears, ventrally, a short sequence of three transverse ridges with intervening grooves where it engages with the orbital/ethmoid articulation (correspondingly corrugated) of the neurocranium. The labial margin of the ramus is gently scalloped on the ventral surface to form a dental platform with spaces for at least eight tooth families (estimated from the number of depressions in the scalloped margin). Palatoquadrate proportions resemble the flattened specimen of *Cladoselache kepleri* figured by Harris (Harris, 1938) but differ from the Tennessee cladoselachian (Maisey, 1989) in which the otic process is only 30% of total length with perhaps a dozen tooth families per jaw ramus. Maisey (1989) argued that cladoselachians lack a condylar jaw joint, but *Maghriboselache* displays, laterally, a ventrally prominent quadrate condyle and mesially a concavity to receive the mandibular knob (Additional file 1: Fig. S33). The jaw articulation is not as well preserved as that of *Ferromirum* (Frey et al., 2020a, b), but appears to have been quite similar, thus it might well have driven the mesio-lateral mandibular rotation described by Frey et al., (2020a, b).

In ventral aspect, the body of Meckel’s cartilage (Figs. 3, 5, Additional file 1: Fig. S33) exhibits a gradual anteromesial curvature towards the symphysis, but in dorsal view, the tooth-bearing platform is straighter, curving mostly in the parasymphysial region. The tooth platform is gently scalloped; there is no continuous dental groove (Additional file 1: Fig. S33E to G). A rounded ventrolateral ridge delimits a shallow adductor fossa on the lateral surface, the fossa extending for almost 60% of mandible length. The jaw articulation resembles *Ferromirum* (Frey et al., 2020a, b) with the quadrate condyle articulating with a well-formed outer (lateral) glenoid posterior and lateral to the mandibular knob (Figs. 3, 5, Additional file 1: Figs. S30, S33). Thus, both primary and secondary components of the jaw joint are present. The mandibular symphysis, although shallow, is deeper than that of *Ferromirum* (Frey et al., 2020a, b), and flexibility was possibly reduced by the presence of large symphyseal teeth in the lower jaw.

The hyoid arch, like that of *Ozarcus* (Pradel et al., 2014), includes hyomandibula, ceratohyal, and hypohyal (Additional file 1: Fig. S33). In *Maghriboselache* the hyomandibula is gently curved, anteriorly expanded, and ~ 50% of ceratohyal length. The ceratohyal is slender, broader and slightly flattened anteriorly, but without the posterolateral flange present in *Ferromirum* (Frey et al., 2020a, b). The lateral fossa is similarly developed as in *Ferromirum* (Additional file 1: Fig. S34). The hypohyal is a simple rod, around 20% of ceratohyal length. Maisey (1989) debated the possible presence of an interhyal in cladoselachians; PIMUZ A/I 5159 corroborates his conclusion that none is present.

**Fig. 7:**
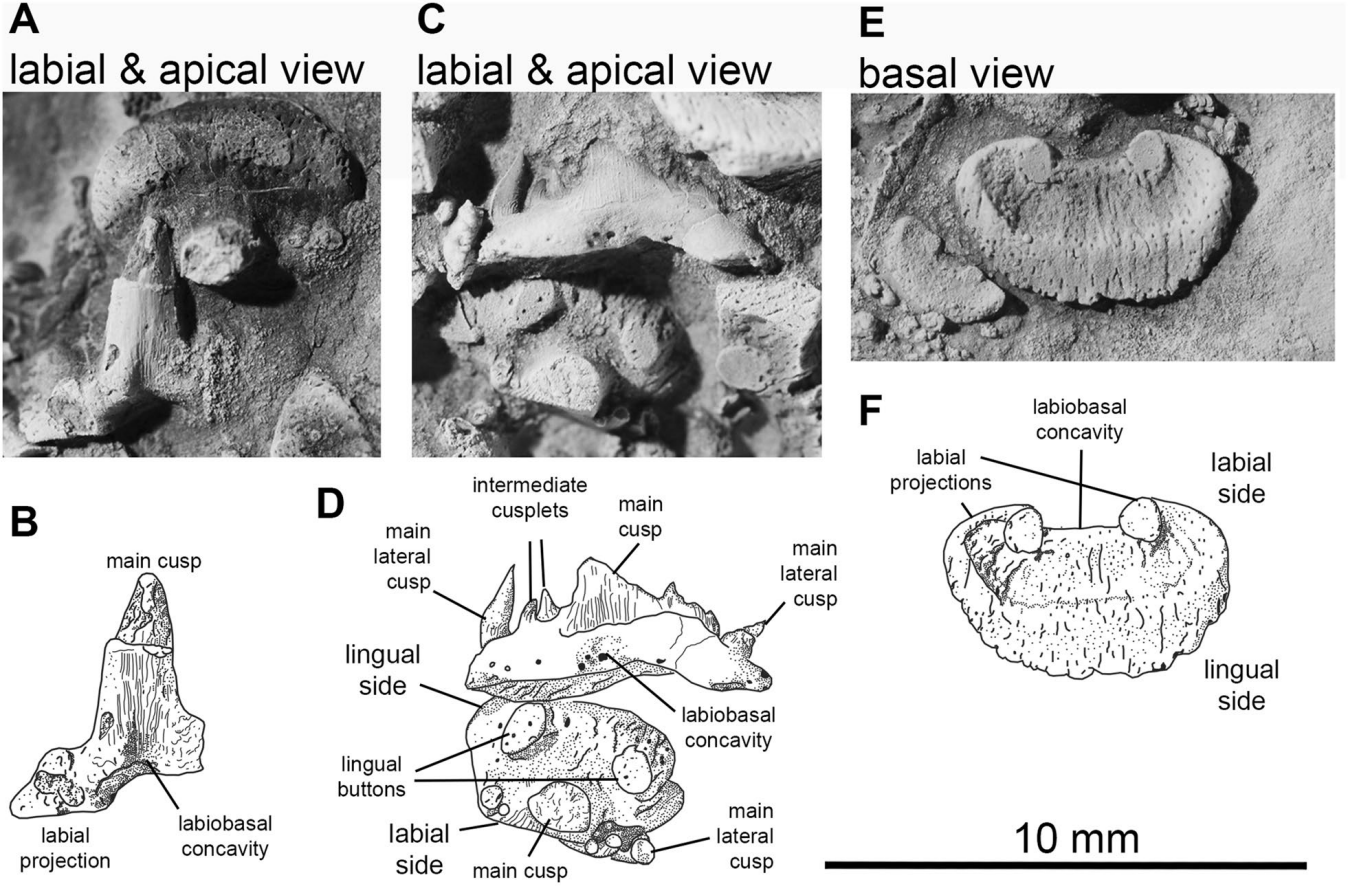
Photos and drawings of the teeth of *Maghriboselache mohamezanei* n. gen. et sp., PIMUZ A/I 5160. 2 tooth rows. **A**, **C**, **E**, photos and **B**, **D**, **F** interpretative drawings of these photos

**Dentition.** The dentition is partially visible in several specimens, including PIMUZ A/I 5160, 5161 and PIMUZ A/I 5154 (Fig. 7). Generative tooth sets are spaced nearly equidistantly along Meckel`s cartilage, with a gap of about 10% of tooth base width between each set (Additional file 1: Fig. S37, S38, S39). Palatoquadrate tooth sets are positioned to oppose those on Meckel’s cartilage. PIMUZ A/I 5162 and AA.TJR.DS.1 show 11 sets per jaw ramus with a distinctive symphyseal set (Additional file 1: Figs. S38, S39), about twice the linear dimensions of teeth in adjacent sets (PIMUZ A/I 5159), capping the mandible. A symphyseal tooth set is reported present in *Cladoselache*, but without the enlarged size relative to the rest of the dentition (Jacquemin et al., 2020; Williams, 2001). The dentition is “cladodont” (Fig. 7): each tooth has a large, finely striated, gently sigmoidal main cusp with one to three pairs of smaller lateral cusps (the outermost being the largest). There appears to be ontogenetic variation in lateral cusp number. As in *Cladoselache* and non-symmoriiform cladodonts, crown material appears to be continuous between cusps (Fig. 7C). Notably, each tooth base includes a deep basolabial depression with adjacent projections, recognized by Ginter et al. (Ginter et al., 2010, p. 58) as the outstanding characteristic of *Cladoselache* teeth. In articulated tooth sets, this depression brackets the main cusp of the preceding (ontogenetically older) tooth. The orolingual surface of the tooth base bears two rounded buttons (Fig. 7C, D). This surface in *Cladoselache* is unknown, but symmoriiforms usually bear a single button (Coates & Sequeira, 2001; Ginter et al., 2010; Zangerl, 1990).

**Branchial skeleton.** Where the branchial arch cartilages are present and preserved, they are mostly exposed in ventral view revealing ceratobranchials and what might be a small, lanceolate, copula (Additional file 1: Fig. S2, S11, S28). Other, smaller, branchial cartilages are preserved, but in all specimens with branchial skeletons the components are jumbled and/or compacted, prohibiting a full reconstruction. PIMUZ A/I 5155 exhibits four to five pairs of ceratobranchials arranged in an anteromedial to posterolateral direction, extending 56 mm posterior to occipital level (measured at the midline); individual ceratobranchial lengths range from 65 to 73 mm. In contrast, the ceratobranchials of PIMUZ A/I 5152 extend some 155 mm beyond the braincase, and a likely copula measures 93 mm long and 40 mm wide.

**Vertebral Column.** PIMUZ A/I 5156 and 5153 preserve 30 to 40 pre-caudal neural arches (Additional file 1: Fig. S21) in original position, distributed above the likely path of an unconstricted notochord: there are no traces of centra. As in *Akmonistion* (Coates & Sequeira, 2001) axial regionalization is evident, especially so in PIMUZ A/I 5156. The cervical region (preceding the pectoral girdle) is the most poorly preserved. Where visible, the neural arches are anteroposteriorly broad: at least ten members are evident. Neural arches become leaner and longer in the thoracic region (see also *Cobelodus*, Zangerl & Case, 1976), but preservation is insufficient to describe finer details of the regional transition. In general, trunk neural arches are slender and tightly packed between their anterior and posterior neighbors. All arches lean posteriorly. The peduncular, pre-caudal, region extends posteriorly from the level of the pelvic girdle to the anterior boundary of the tail. Not as well preserved as the trunk neural arches, these arches are thinner, inclined more posteriorly, and decrease in size towards the tail.

**Fig. 8:**
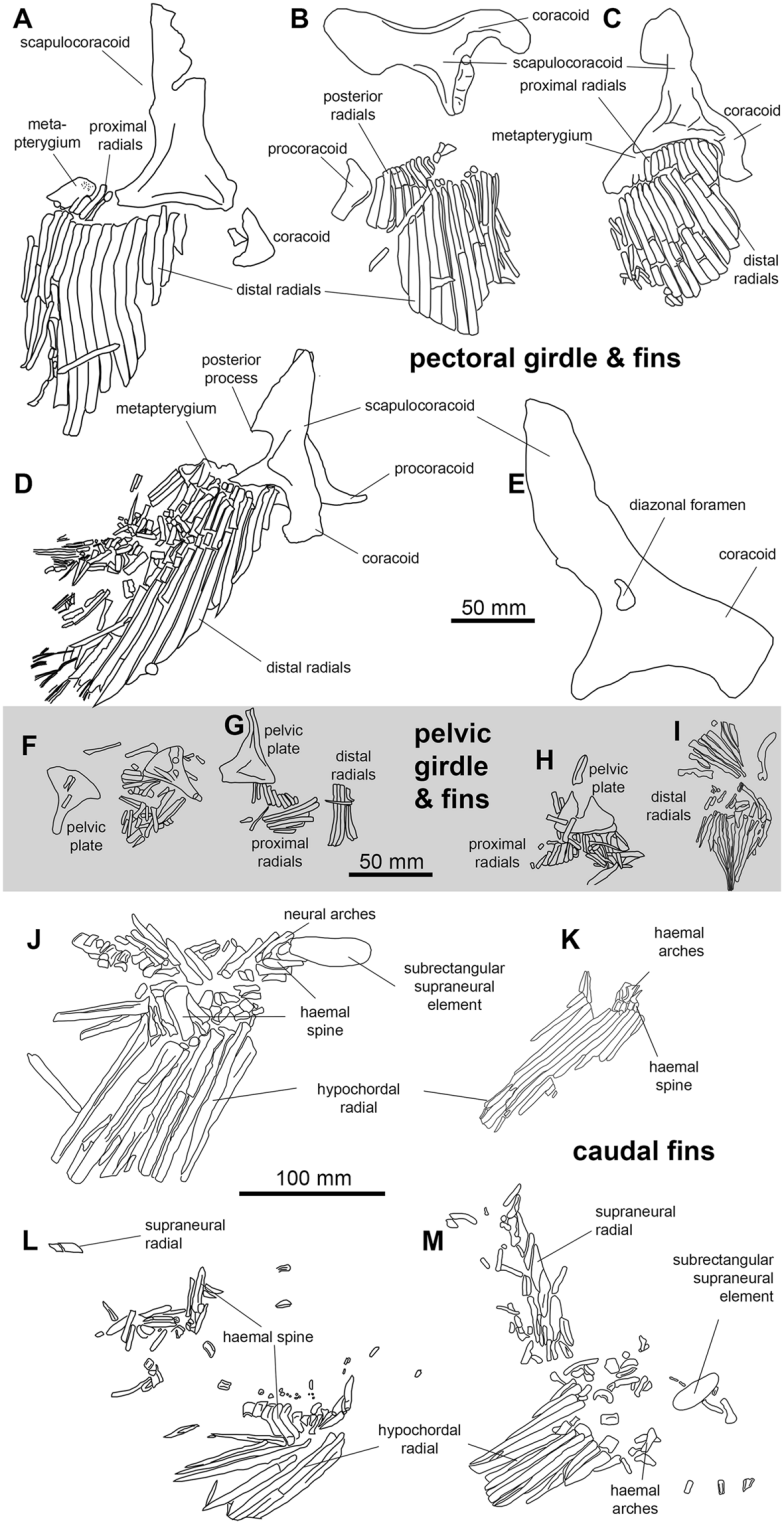
Fins of *Maghriboselache mohamezanei* n. gen. et sp. **A** left pectoral fin of the holotype AA.MEM.DS.12. **B, C** right and left pectoral girdle elements and pectoral fins of PIMUZ A/I 5155; **D** left pectoral girdle elements and left pectoral fin of PIMUZ A/I 5156, **E** left scapulocoracoid of PIMUZ A/I 5152. **F** to **I** Pelvic girdle and pelvic fins. **F** PIMUZ A/I 5156, **G** PIMUZ A/I 5163, **H** PIMUZ A/I 5155, **I** PIMUZ A/I 5153. **J** to **M** Caudal fins. **J** AA.MEM.DS.12, **K** isolated fin, PIMUZ A/I 5163, **L** PIMUZ A/I 5153, **M** AA.BER.DS.01. Photos of the fins are in Fig. 1 and in Additional file 1: Fig. S5 to S11, S13, S14, S16 to S18, S20 to S25, S27, S28

**Pectoral girdle and fins.** As in *Cladoselache* (Bendix-Almgreen, 1975; Dean, 1909a; Tomita, 2015), the pectoral girdle and fins﻿ are large and well developed. The scapulocoracoids (Fig. 8A–E) are best preserved in PIMUZ A/I 5155 (146 mm long from anteroventral to dorsal extremities), PIMUZ A/I 5152 (194 mm long) and PIMUZ A/I 5158 (104 mm). However, like most Paleozoic chondrichthyan specimens, these cartilages are flattened and distorted, although those of *Maghriboselache* are more robust than other symmoriiform examples (Additional file 1: Fig. S48). The distinctively shaped scapular process approximates to a right-angled triangle, the posterior edge of which (the hypotenuse) is slightly convex. A deep embayment in the posterior margin divides the bulk of the process from the articular region. The process forming the posteroventral extremity of the scapular region (or posterodorsal angle of the embayment) might be the homologue of the posterior process present in other symmoriiforms pectoral girdles, but here present in an unusually ventral location. A small diazonal foramen is located dorsal to the horizontal articular crest, which projects posteriorly to terminate as a condyle for the metapterygial plate (Fig. 8E). The coracoid region is less well preserved than the scapular process but appears to project ventro-medially for a substantial distance. A small, separate, L-shaped procoracoid cartilage is present, well-preserved in PIMUZ A/I 5156.

**Fig. 9:**
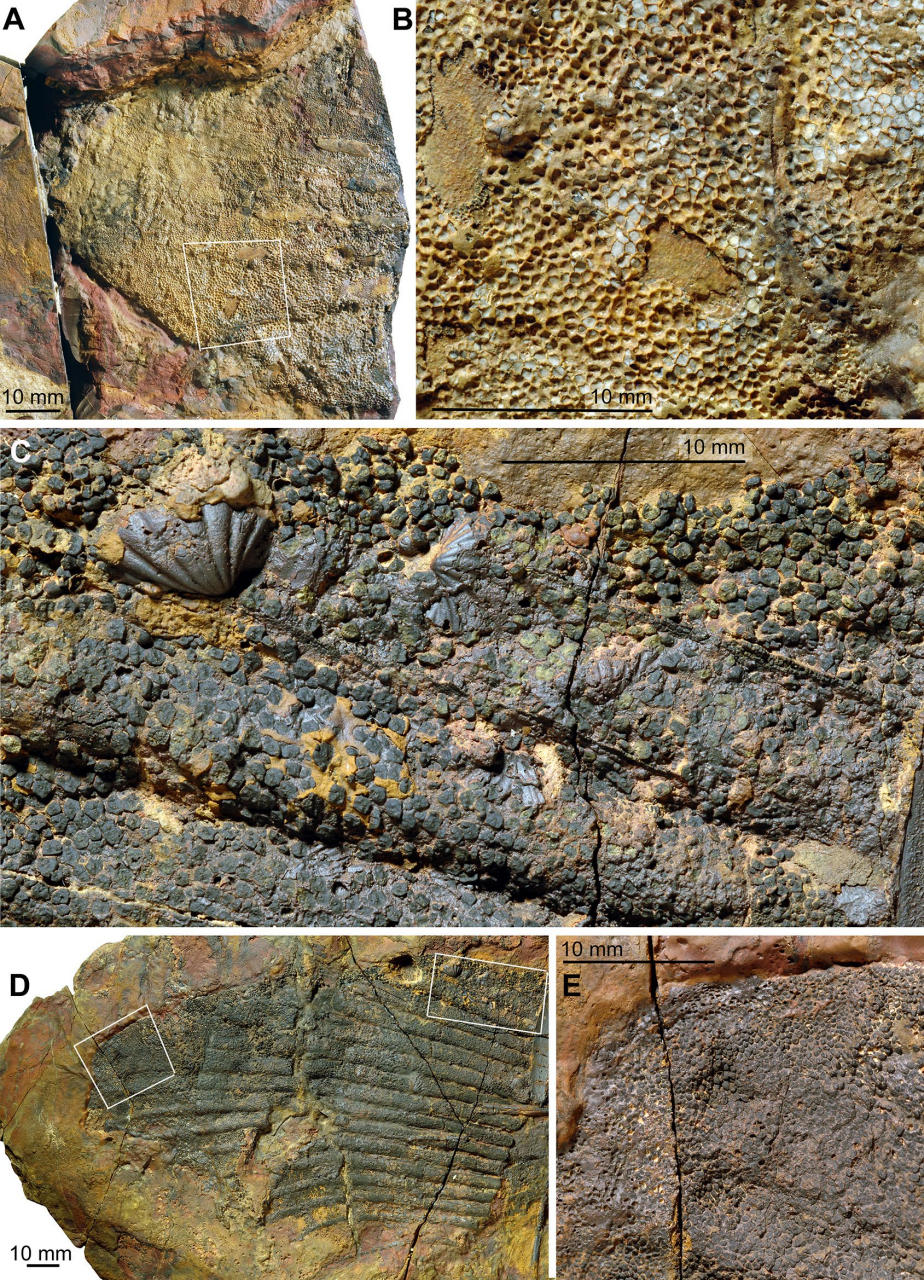
Details of the pectoral fins of *Maghriboselache mohamezanei* n. gen. et sp., PIMUZ A/I 5154. **A** tip of the left fin with articulated, though weathered integument, showing the localization of figure B. **B** detail of the fin with dermal denticles. **C** detail of the right pectoral fin showing the dermal denticles overlying the radials. **D** overview over the right fin, showing the localization of figure C and E. **E** detail showing the integument at the distal tip of the fin. Note the reduced denticle size

The pectoral fin is known from five specimens (Figs. 8, 9, Additional file 1: Fig. S49). The overall shape is strikingly like that of *Cladoselache*, resembling an elliptical wing with a strongly convex leading edge. Around fifteen or sixteen pre-metapterygial radials largely fill the fin envelope (Figs. 8A–D, 9D), with a distal fringe of ceratotrichia contributing to the trailing edge (Additional file 1: Fig. S23). Excluding the first three or four unsegmented members, each radial consists of a short proximal and much longer distal segment. The proximal segments are cylindrical, but the distal segment, although cylindrical proximally, broadens and flattens distally into a strap-like form (Fig. 8D) also evident in *Cladoselache* (Bendix-Almgreen, 1975; Tomita, 2015) and *Fadenia* (Zangerl, 1981). In PIMUZ A/I 5156 (Additional file 1: Fig. S22) and PIMUZ A/I 5154 (Additional file 1: Fig. S13), there appear to be additional articulations within the single rays, indicating the presence of intercalated segments. The metapterygium (preserved in PIMUZ A/I 5158) is small and bears three facets for radials and a distal articulation for a presumably short, trailing whip (Fig. 8, Additional file 1: Fig. S28). The articulation surface with the scapulocoracoid is a shallow concavity. Unlike *Cladoselache* (Tomita, 2015), there are likely segments of a metapterygial whip, around six pieces in total. Where the pectoral fin skin is preserved (PIMUZ A/I 5154), dermal denticle size changes from larger (0.8 mm) to smaller (c. 0.4 mm) distal denticles with polygonal outline.

**Pelvic girdle and fins.** The pelvic girdle is preserved in several specimens (AA.MEM.DS.12, PIMUZ A/I 5155, 5156, 5158). Each half (left and right) consists of a fan-shaped, or subtriangular, plate (Fig. 8F–I). In many reconstructions of similar pelves, the narrow process of such plates is restored directed dorsally. It seems more likely that this process was directed medially and horizontally towards its counterpart, contributing to a divided pelvic bar (as suggested by the dorsoventrally flattened remains of PIMUZ A/I 5155. In this orientation the distal, broadest side of the plate, serving as articular surface for the pelvic fin, is directed more-or-less laterally, like the articular crest for the pectoral fin.

In PIMUZ A/I 5155 and 5158, a few short, rod-shaped proximal radials articulate to the pelvic girdle; in PIMUZ A/I 5153, the total reaches 10. Proximal segments are short and cylindrical and distal segments are long and slender, tapering distally. Anterior and posterior radials are the shortest with length increasing gradually towards the midpoint of the fin. No trace of pelvic clasper has been found.

**Caudal fin.** The caudal fin is known from specimens AA.MEM.DS.12, PIMUZ A/I 5152, 5153 and AA.BER.DS.01 (Fig. 8J–M). None of these preserves a complete articulated tail. The restoration is modelled after *Akmonistion* (Coates & Sequeira, 2001) rather than *Cladoselache* (Zangerl, 1981) because individual parts display proportions resembling those of the former rather than the latter example.

Specimen AA.BER.DS.01 (Fig. 8M) includes a caudal skeleton with six to eight neural arches or spines in the upturned chordal, dorsal lobe, and eleven or more elongate hypochordal radials in the ventral lobe. The angle between dorsal and ventral lobe axes exceeds 90 degrees, consistent with a caudal fin outline resembling that of *Akmonistion*, and the high aspect ratio tails of symmoriiforms and edestids in general. The neural arch/ spine and supraneural cartilages are broad (three examples preserved in AA.MEM.DS.6) but not as broad as those of *Cladoselache*. A scatter of subjacent cartilages might be the remains of dorsal lobe hypochordal arches and spines. A scattering of short proximal radials and hypochordal arches lie proximal to the elongate radials of the ventral lobe. Some of these have the characteristic elbow-shape of those situated directly beneath point of notochordal upturn in *Akmonistion* (Coates & Sequeira, 2001) and *Cobelodus* (Zangerl & Case, 1976). In AA.MEM.DS.12 a dorsal, oval to subrectangular supraneural structure some 50 mm long and 17 mm wide lies directly anterior to the dorsal lobe. A similarly position cartilage in the most complete specimen of *Akmonistion* (Coates & Sequeira, 2001: Fig. 9A) is a displaced radial. In AA.MEM.DS.12, the item appears to be part of the caudal axial skeleton.

**Fig. 10:**
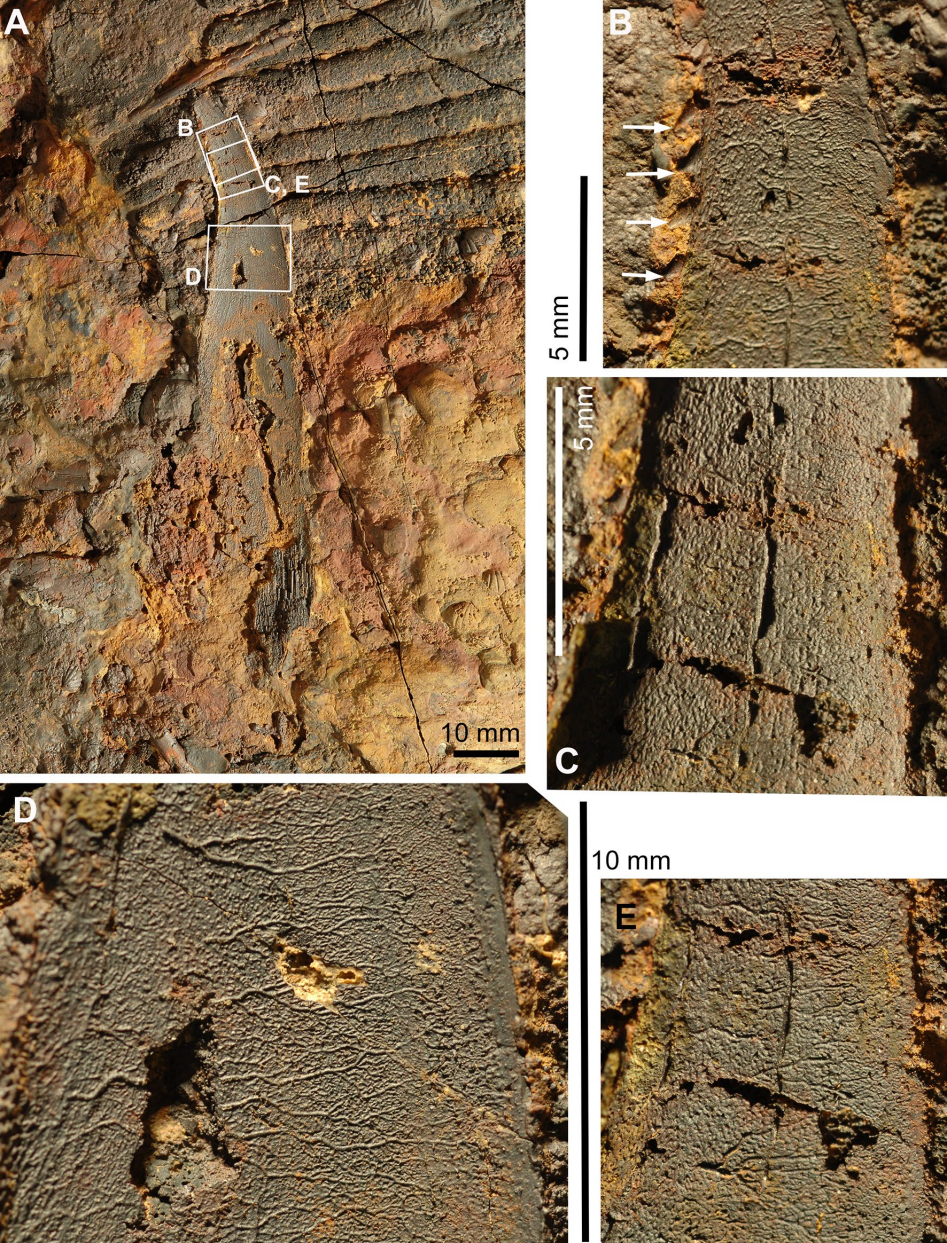
Details of the fin spine of the anterior dorsal fin of *Maghriboselache mohamezanei* n. gen. et sp., PIMUZ A/I 5154. **A** overview, showing the localization of figures B to E. **B** detail of the tip with denticles at the posterior edge. **C** detail showing the fine longitudinal striation. **D, E** details showing imprints of blood vessels

**Dorsal fin spines and fins.** The anterior dorsal fin spine is known mostly from a natural mold in PIMUZ A/I 5154 (Fig. 10); spine fragments are present in PIMUZ A/I 5153, 5157 and 5158. The general shape resembles spines-forms *Ctenacanthus major* and *C. varians* (Maisey, 1981): stout, broad-based, and curved posteriorly. As in other spine-bearing symmoriiforms, surface ornament is absent but the mold suggests presence of an anterior keel. Furthermore, at least four well-formed, non-overlapping denticles are present on the trailing edge close to the spine apex (Fig. 10B). The spine base is in poor condition, but it appears that it was inserted at an angle of about 50° to the dorsum. Longitudinal striations marking the spine base are probably weathering artefacts or remains of internal structures rather than ornament. The cast surface in PIMUZ A/I 5154 shows fine details of blood vessel imprints (Fig. 10D). These originate near the trailing edge (0.1 to 0.2 mm thick) and finely branch anteriorly (ca. 0.01 mm wide), entering the fin spine behind the putative keel. This suggests that in life much of the spine was covered by living tissue.

The posterior dorsal fin spine is preserved in AA.MEM.DS.12, AA.MEM.DS.6 and AA.BER.DS.01 (Additional file 1: Figs. S19, S24). The spine is squat and approximately one third of first dorsal fin spine height. The most complete specimen AA.BER.DS.01 is preserved with the leading edge uppermost (Additional file 1: Fig. S19). The course textured surface shows no ornament and no anterior keel; denticles presence is unknown.

**Fig. 11:**
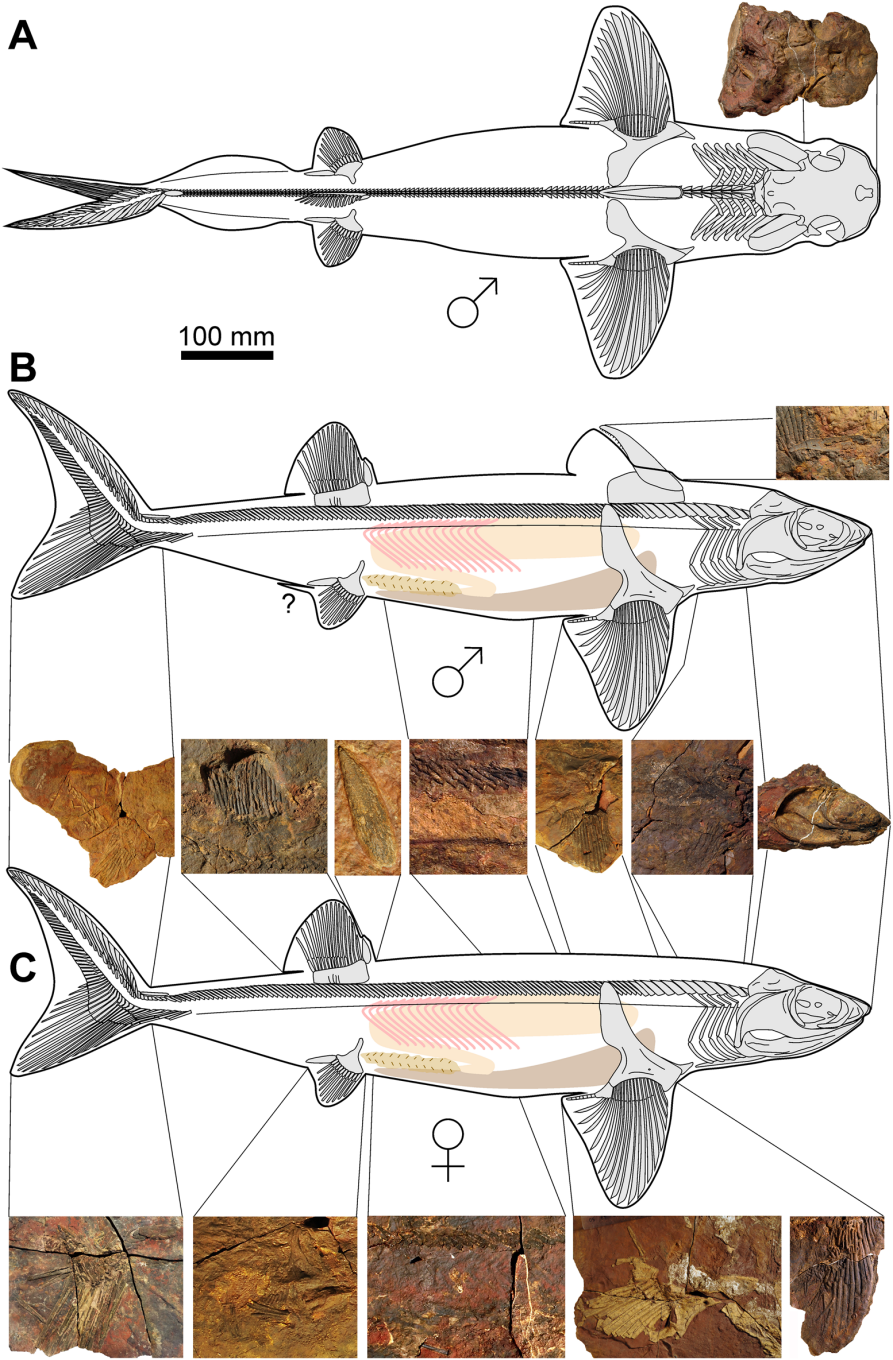
Skeletal reconstruction of possibly a male (**A** dorsal. **B** lateral) and possibly a female (**C** bottom, lateral) of *Maghriboselache mohamezanei* n. gen. et sp. The according details were collected from the mentioned specimens. Note the presence of anterior dorsal fin spine in the supposed males, possibly serving a role during copulation. Claspers are not present in the supposed males of our sample. The photos show the according regions of specimens displayed in Fig. 1 and the Additional file 1

Presence of an anterior dorsal fin is uncertain (Fig. 11). No radials are preserved; hence this fin is likely absent in specimens lacking an anterior dorsal spine. Fin evidence is limited to the shape of basal cartilage associated with the fin spine. The partially preserved basal cartilage in PIMUZ A/I 5158 articulates with the entire fin spine base (Additional file 1: Fig. S28). Visible over a length of about 33 mm, the cartilage ventral, dorsal and anterior edges are covered by phosphatized musculature; thus, the outline is obscured. It appears to be subrectangular, elongate, and projects for about a third of its length posterior to the fin spine. This suggests service as fin support as well as spine support.

The posterior dorsal fin is preserved in most of the skeletons; in some positioned nearly vertically or obliquely relative to the bedding plane (AA.MEM.DS.12, PIMUZ A/I 5155, 5156; Fig. 1, Additional file 1: Figs. S1, S7–S9, S26). The fin has a conventionally rounded outline with a steeper leading edge and more gradually inclined, convex, trailing edge. AA.MEM.DS.12 preserves 19 radials and PIMUZ A/I 5156 at least 16, with 8 + contributing to the leading edge. Radials are divided into proximal (short) and distal (long) segments, with the single intra-radial articulations more-or-less aligned with the level of the body outline. A broad basal cartilage supporting the diminutive fin spine articulates with at least the anteriormost 10 radials. There is no evidence of a delta-shaped cartilage (cf. the ‘V’-shaped element noted by Zangerl and Case (1976).

**Fig. 12:**
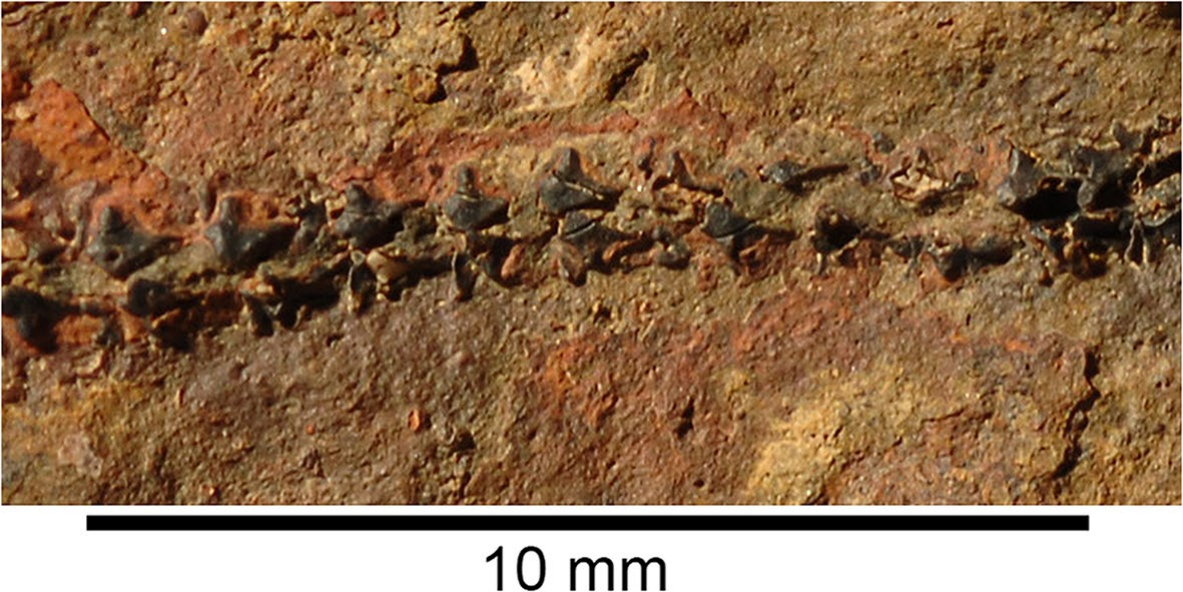
Detail of thorax of Maghriboselache mohamezanei n. gen. et sp., PIMUZ A/I 5156, showing the modified scales of the lateral line

**Scales.** The integument bears small individual ‘polyodontode’ (Ørvig, 1977) scales, approximately 0.3 mm wide to 0.8 mm long (Figs. 9C, 12, Additional file 1: Fig. S16.—PIMUZ A/I 5156). Scale crowns are mostly flattened with two to four longitudinal striations on the external surface. Some crowns are not completely flat, but with a convex surface.

Specialized lateral line scales are present (Fig. 12). These scales are neither crescent- nor ring-shaped, as in fossil taxa such as *Akmonistion* (Coates & Sequeira, 2001) and *Orestiacanthus* (Lund, 1984), and extant holocephalans. In contrast, the scales have more angular bases combining in pairs to form the lateral line groove. The scale crowns (single pointed cusps) are oriented away from the groove, directed dorsally or ventrally.

**Soft tissues.** Soft tissue-remains are frequently preserved (Additional file 1: Figs. S9, S14, S20, S26 to S28). Muscle fibers can be seen in several specimens. PIMUZ A/I 5156 preserves still distinguishable V-shaped myomeres and collagenous myosepta (Additional file 1: Figs. S20, S26–S28). These haematized muscle tissues (Frey et al., 2019, 2020a, b) appear as elongated black rolls with fine longitudinal striations. Consequently, the body outline is discernible in most specimens (Fig. 1). In specimen PIMUZ A/I 5153, the posterior tip of one lobe of the liver is preserved in a position just anterior to the pelvic fins. In PIMUZ A/I 5155, the stomach contains a more or less articulated small actinopterygian (40 mm long).

### Phylogenetic analysis

**Fig. 13:**
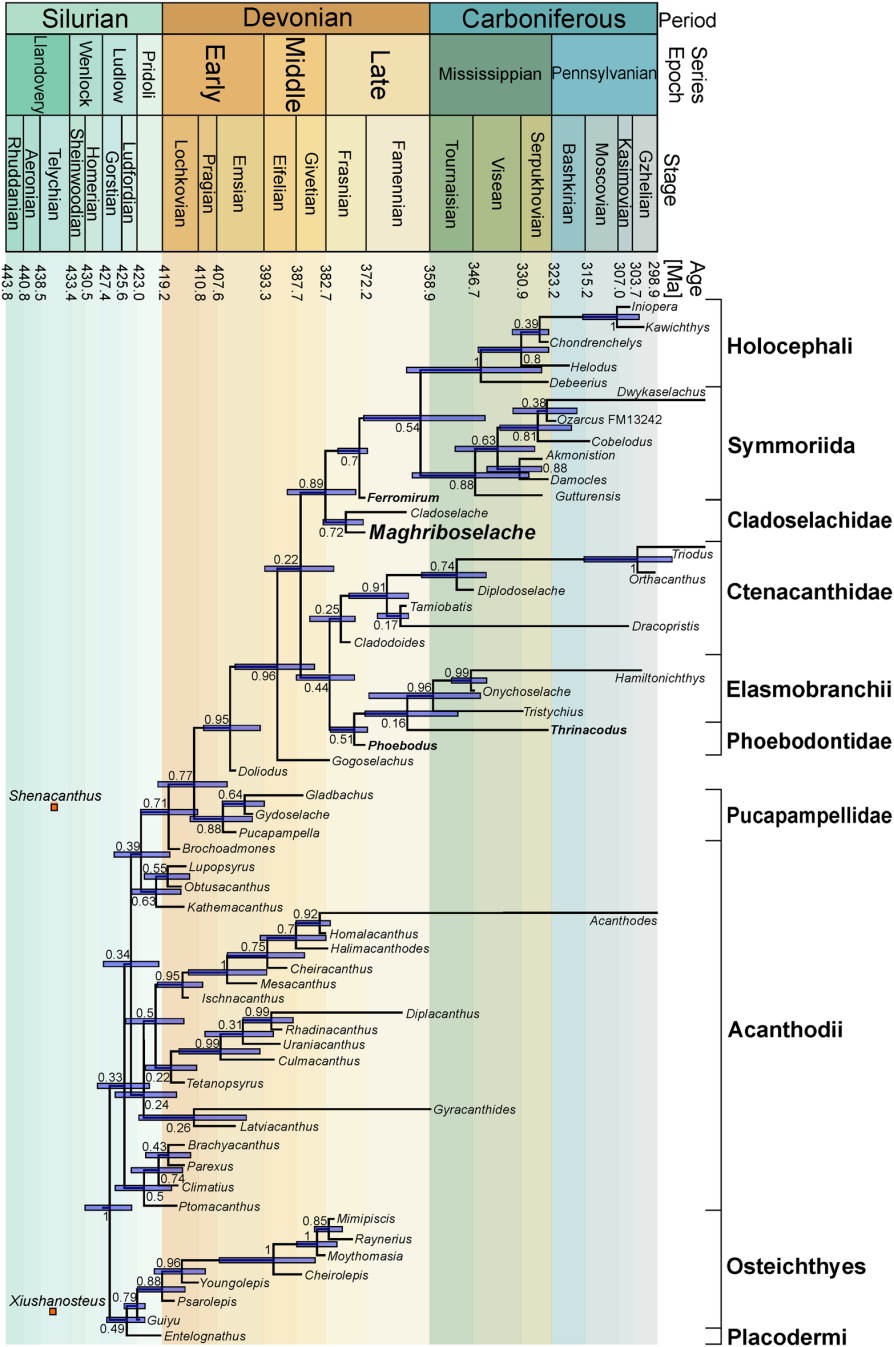
Phylogeny based on the emended character matrix from Coates et al. (2017) and Frey et al., (2020a, b)**.** Bayesian tip-dating analyses of 230 characters were performed using the fossilized birth–death model. Early Silurian species after Zhu et al. (2022)

Bayesian tip-dated analyses were performed in BEAST 2.6.3 (Bouckaert et al., 2019) with a data matrix developed from Coates et al., (2017, 2018), Dearden et al. (2019) and Frey et al., (2020a, b). The Nexus- files are available as Additional files 7, 8, 9 and 10. We added characters capturing body proportions (see Additional files 2 to 6) as well as some tooth characters used by Hodnett et al. (2021). Accordingly, we ran separate analyses with between 230 (Fig. 13; details see Additional file 1) and 238 characters, respectively. The results of both analyses using 230 and 238 characters recovered *Maghriboselache mohamezanei* as sister taxon of *Cladoselache* (Fig. 13, Additional file 1: Figs. S41, S42). In the analysis of the 230-character matrix, this clade was recovered as sister group to all other total-group holocephalans (Fig. 13). The analysis using the 238-character matrix resulted in *Maghriboselache* and *Cladoselache* as sister group to symmoriids, with holocephalans as sister group to symoriids and cladoselachiids (Additional file 1: Fig. S42).

*Cladoselache* and *Maghriboselache* share the distinctive tooth morphology and the shape of the central and posterior part of the neurocranium. The known neurocrania of *Cladoselache* are mostly flattened, usually with poor detail of the ethmoid region (Maisey, 2007: Fig. 60, CMNH 5611). However, Dean (1909b: pl. 288) figured a specimen displaying the narrow V-shaped arrangement of the lower jaw and the broad nasal capsule of the neurocranium in a flattened state. *Maghriboselache* has a broad anterior rim of the internasal plate and nasal chambers. The two genera share the large paired fins with a similar outline, but relative to body size, the pectoral fins are bigger in *Cladoselache*. In both genera and the pectoral radials are strap-like, although in *Cladoselache*, this morphology is present from leading to trailing edge.

In *Maghriboselache*, two dorsal fin spines are present at least in one sex, while the posterior fin spine in *Cladoselache* is ‘hypothetical’ (Zangerl, 1981: Fig. 73). This character might be shared with some symmoriids like *Falcatus* and perhaps *Stethacanthus*. The overall shape of the caudal fin (lunate, high aspect ratio) is characteristic for symmoriiform taxa. Accordingly, we suggest that *Cladoselache* and *Maghriboselache* form a monophylum, which likely is sister to all other symmoriids, all are geologically younger except *Ferromirum*, which is of identical age. *Maghriboselache* was found in the Thylacocephalan layer, which is older (middle Famennian, 369 – 369.5 Ma; Frey et al., 2018, 2020a, b; Jobbins et al., 2020) than the Cleveland shale (late Famennian, 360 – 358.9 Ma; Cushing, 1912) from where *Cladoselache* is provenant. Presuming our phylogenetic scheme is correct, *Maghriboselache* and *Ferromirum* are the oldest stem holocephalans known from articulated material.

The presence of two dorsal fin spines in association with the corresponding basal fin cartilage is possibly the plesiomorphic condition in early chondrichthyans (Frey et al., 2019, 2020a) because it occurs in several Famennian representatives of the main clades of the chondricthyan crown group. The overall shape of the anterior fin spine resembles that of *Ferromirum* in the caudally oriented curvature close to the apex and the smooth surface (Frey et al., 2020a). By contrast, symmoriiforms may have spine brush complexes, ctenacanths and phoebodontids have nearly straight and striated anterior fin spines.

## Discussion

Our results, placing *Maghriboselache* and *Cladoselache* as monophyletic sister group to either all other total-group holocephalans (Fig. 13) or to symmoriids (Additional file 1: Fig. S42), are broadly consistent with others (Coates et al., 2018; Dearden et al., 2019; Frey et al. 2019, 2020a, b) derived from the same core matrix (Coates et al., 2017) on *Dwykaselachus*, see Additional file 1). Given the interrelatedness of these trees, other data sets yielding different outcomes deserve acknowledgement, notably those that place symmoriiforms as stem elasmobranchs (Hodnett et al., 2021; Lund & Grogan, 2004) and stem chondrichthyans (Denton & Goolsby, 2021; Pradel et al., 2011). These results are linked to different approaches such as a focus on dental characters (Hodnett et al., 2021), a lower number of more or less different characters (Grogan et al., 2015; Pradel et al., 2011) and, in each of these analyses, a far more restricted use of morphologically instructive taxa. In the present work, the discussion is focused solely on the stem holocephalan hypothesis shown in Fig. 13, the light it sheds on the timing of early chondrichthyan phylogeny, and consequent patterns of character (trait) evolution. The introduction of *Maghriboselache* as perhaps the earliest diverging member of the holocephalan total group and the oldest together with *Ferromirum* is central to this work, offering insights into transitional states between conditions at the chondrichthyan crown node and specializations evident in more derived Paleozoic members of the chimaeroid stem lineage.

**Timing: clade origins.** As suggested by Zhu et al. (2022) and Andreev et al. (2022), the roots of the total clade Chondrichthyes date back to the early Silurian or even late Ordovician (Fig. 13). These findings challenge our understanding of acanthodian interrelationships and the early evolution of chondrichthyan traits, but are of less immediate relevance, both chronologically and phylogenetically, to the evolution of the chondrichthyan crown group. In fact, fossils documenting the crown group origin appear to correlate with the Devonian Nekton Revolution (Klug et al., 2010), where the free water column was increasingly occupied by nekton. The set of taxa used in the present phylogenetic tree exceeds previous editions in terms of anatomical detail and disparity but remains limited in terms of diversity: genera and species are restricted to those known from at least partly articulated material. Taxa based on isolated teeth, dental plates, and fin spines are excluded because inclusion would likely obscure phylogenetic signal. Nevertheless, such fragment-based taxa can add to estimates of macroevolutionary pattern when incorporated post-hoc with reference to established supra-generic taxonomic assignments. Teeth, spines, and dental plates might change divergence time estimates and inferences on trait evolution even if their phylogenetic position is underdetermined, i.e., occupying no more than *incertae sedis* positions on the tree. Further to this, the temporal relation of clade origins and morphological innovations to the end Devonian Hangenberg extinction event is of particular interest, given the likely impact of this biotic crisis on subsequent vertebrate biodiversity (Sallan & Coates, 2010).

The time-calibrated chondrichthyan phylogeny presented in Fig. 13 shows 63% of tips and 76% of mean divergence time estimates predating the end-Devonian mass extinction event. Thus, most branching events of interest (in the present analysis) are embedded within the Devonian, which corresponds well with molecular clock data (Inoue et al., 2010) and a recently presented tree including earliest chondrichthyans (Andreev et al., 2022). In particular, the divergence of the chondrichthyan crown clade is estimated between 393.9 (Emsian-Eifelian boundary) and 377.1 mya (middle Frasnian), with the mean estimate rooting elasmobranchs and holocephalans into the Givetian (Middle Devonian) some twelve million years earlier than *Maghriboselache*. A mere six lineages survive through to the Carboniferous and two of these are ‘acanthodian’ stem chondrichthyans.

Regarding trait evolution, holocephalan body fossils with durophagous/crushing dentitions and/or specialized teeth (e.g., petalodonts) are exclusively post-Devonian, suggesting that this morphological innovation occurred in an extinction aftermath. However, the Late Devonian includes a diverse range of isolated chondrichthyan durophagous teeth and dental plates. Famennian records of holocephalan dental items include three helodontiforms (*Helodus devonicus*, *incipiens*, and *rowleyi*), two cochliodontiforms (*Sandalodus minor*, *Thoralodus cabrieri*), and one psammodontiform (*Psammodus* sp.; Stahl, 1999). Frasnian records add a further three copodontiforms (*Acmoniodus clarkei*, *Synthetodus trisulcatus*, *S. calvini;* Stahl, 1999), and the Givetian includes a likely copodontiform: *Melanodus loonesi* (Darras et al., 2008). Two further symmoriiforms (*Stethacanthus* sp., *Denaea* sp.; Ginter et al., 2010) are known from the Fammenian, as well as a putative petalodontiform (*Ageleodus pectinatus;* Ginter et al., 2010). Orodontiforms and eugeneodontiforms (‘edestids’) have strikingly symmoriiform-like tails and holocephalan-like tooth histologies (Janvier, 2002; Zangerl, 1981). Although known mostly from the Pennsylvanian and Permian, Famennian records include at least one orodontiform (*Orodus elongatus;* Ginter et al., 2010).

This record of dental fragments transforms the phylogenetic picture. Except for a few distinctive groups such as the Iniopterygii (Zangerl & Case, 1976) and the Chondrenchelyidae (Berg, 1940), all major early divisions of a broadly inclusive hypothesis of holocephalan membership are already present in the Late Devonian. The variety of dental morphologies evident in the pre-Carboniferous record signals that the specialized feeding apparatuses of these groups were also in place. Some major morphological innovations preceded the Hangenberg extinction. Several of these records might be used as a new hard minimum calibration for the chondrichthyan crown node. It is also noteworthy that this preliminary search found no putative stem holocephalans that might exceed the existing Givetian crown-node date estimate. This, at least, is consistent (in terms of minimum range extensions) with a crown rather than stem chondrichthyan position for *Maghriboselache* and its relatives. A direct reading of the stratigraphic occurrences suggests that all major clades, including those with crushing dental plates, evolved in rapid succession and persisted to radiate further in the post-Devonian Paleozoic seas.

Stem elasmobranch records (obtained from Ginter et al., 2010) deliver a different signal, summarized here for comparison. Phoebodonts increase from two Givetian taxa to three Frasnian to twelve Famennian species, of which representatives of two genera survive into the Mississippian. Post-Devonian phoebodonts (e.g., *Thrinacodus/ Thrinacoselache*) are rare. Cladodonts are represented by at least three Frasnian and seven Famennian taxa, of which at least one genus persists in the Mississippian, alongside a multitude of post-Devonian cladodont forms. Protacrodonts include one Frasnian and three Famennian taxa, but none are sustained into the Mississippian. Hybodontiforms, however, include at least two Famennian genera, one of which persists in the Mississippian. The addition of these ‘shadow’ taxa to the elasmobranch division would shunt the phoebodont node back into the Givetian, and the earliest hybodontiforms into the latest Devonian. The number of Hangenberg event-surviving lineages would be modestly enlarged, but the general picture of Devonian diversity is changed mostly to accommodate an increased range of Devonian-specific taxa, such as phoebodonts, which die out at, or before, the boundary. Unlike in the holocephalan subtree, there is no signal pulling the Mississippian evolutionary radiation back into the Devonian. But there is some consensus, too: there are no instances of total-group elasmobranch tooth forms exceeding the Givetian crown-date estimate, just as there are no instances of total-group holocephalan teeth or dental plates that exceed the Givetian crown-date estimate.

**Traits: *Maghriboselache and morphological innovation***. Symmoriiforms and chimaeroids are united by a suite of neurocranial features, leading to the question of whether *Maghriboselache*, as the apparent outgroup to all other known members, exhibits these shared derived traits. The most visible of these specializations is presence of a compact otico-occipital unit, with a midline endolymphatic duct, a closed otico-occipital roof, and a small occipital arch tucked between the otic capsules (Figs. 2, 3, 9). For contrasting states, in *Doliodus* and pucapampellids (Maisey et al., 2018), *Cladodoides* (Maisey, 2005), *Tristychius* (Coates & Tietjen, 2018), xenacanths and ctenacanths (Schaeffer, 1981), the otic roof is interrupted by a median fossa and large posterodorsal fontanelle; see also the presence of splayed lateral otic processes, broad lateral otic ridges, and posteriorly projecting occipital units.

**Fig. 14:**
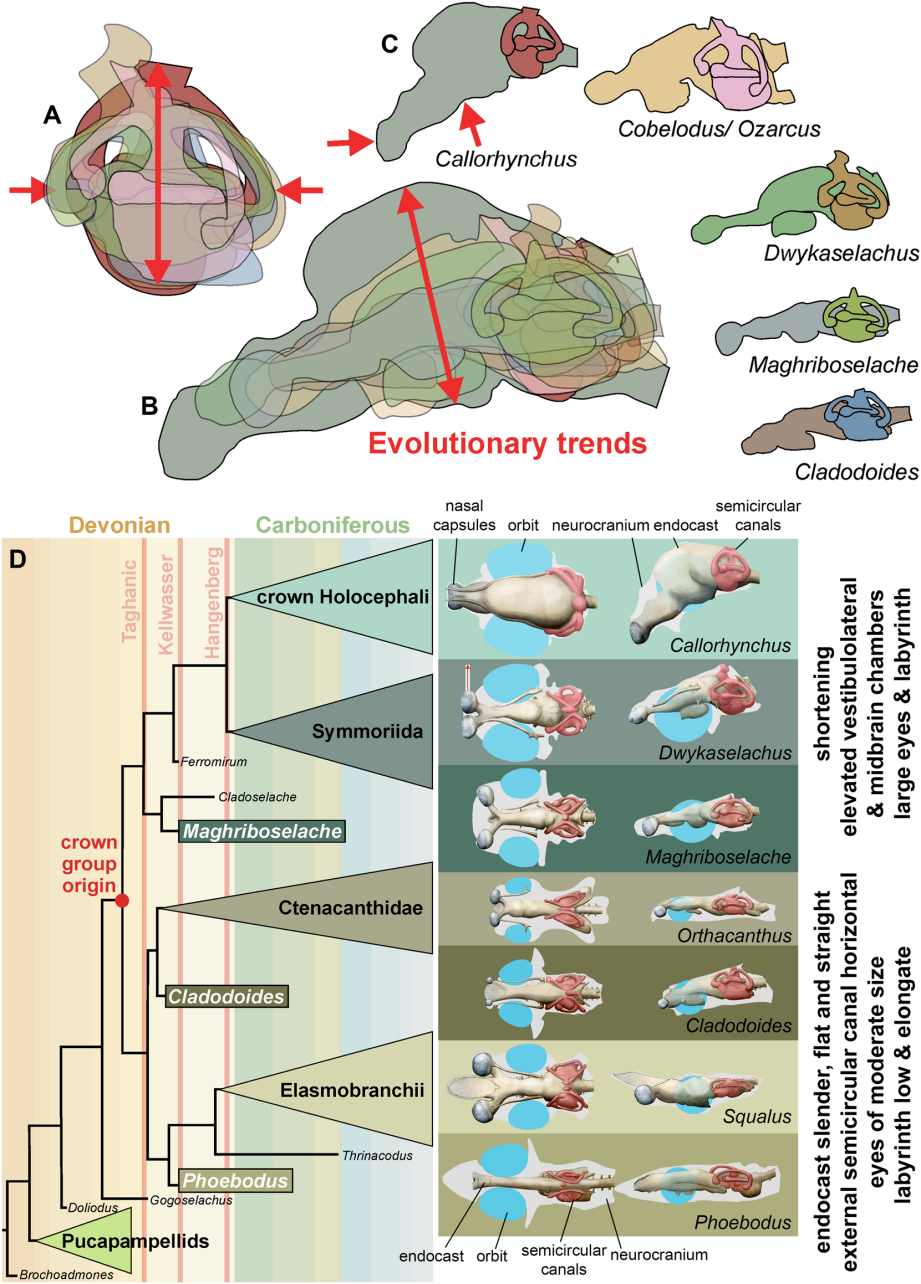
Endocasts of various extinct and extant chondrichthyans of the main chondrichthyan clades and evolutionary change in the holocephalan branch.** A** Overlain labyrinths of the genera in F (note the increase in height and decreasing length). **B** Overlain endocasts of the genera in C (note the increasing volume of the mesencephalon and the dorsal bulging). **C** Outlines of endocasts of some chondrichthyans. **D** Evolution of neurocrania, sensory organs, brain endocasts and labyrinths exemplified with seven genera. Eyes reconstructed in blue, labyrinth in red, neurocranium outline in grey. Most endocasts after Coates et al. (2017), *Phoebodus* after Frey et al. (2019), and unpublished data, *Maghriboselache* after this paper

Nevertheless, beneath the strong characteristic similarities of the external shapes of the otico-occipital units of *Maghriboselache*, *Dwykaselachus* and others, there is evidence of an incremental transformation. Remarkably, especially for the early record of chondrichthyans, endocasts of three symmoriiforms are now available: *Maghriboselache*, *Dwykaselachus* (Coates et al., 2017) and “*Cobelodus*” (Maisey, 2007). Relative to these, the endocast of *Cladodoides* (Maisey, 2005) adds a reasonable model of general, early chondrichthyan conditions. If all four endocasts are aligned and scaled to the same size of otic capsule with the utricular recess and crus commune apex super-positioned, the sequence running from *Cladodoides* to *Maghriboselache, Dwykaselachus,* and *“Cobelodus”* presents a transitional series (Fig. 14). Compared to the approximately linear arrangement (in lateral view) hindbrain to forebrain endocast of *Cladodoides*, the cranial endocasts of *Maghriboselache, Dwykaselachus,* and *“Cobelodus”* show, in sequence, successively more elevated vestibulolateral and midbrain chambers (Fig. 14A–C).

The roofs of these endocranial spaces rise from below to well above the anterior semi-circular canal; the boundary at the rear of the vestibulolateral chamber is displaced dorsally and posteriorly to lie level with the endolymphatic duct canal; the hypophyseal space rises, and the canal for the anterior of the spinal cord and spino-occipital nerves is shortened and confined to the ventral space between the posterior extremities of the otic capsules. The net effect of these changes is strikingly like those reconstructed for the dinosaur-to-bird skull transition (Bhullar et al., 2012). In both instances the main chamber enclosing the brain is tilted up and back in the skull, and in both this is accompanied by, if not driven by, increased orbit size (cf. discussions in Bhullar et al. (2012) and Coates et al., (2017: Fig. 3).

The semi-circular canal arrangement is similarly transformed (Fig. 14A). Maisey (Maisey, 2007: Fig. 32) recognized a change in the trajectory of the external canal relative to the endocast long axis: the external canal is tilted anterodorsally. The present data set reveals two further labyrinth-related changes: the anterior semi-circular canal ampulla, instead of lying ventral to the external (horizontal) ampulla, is repositioned to lie level with or dorsal to the external ampulla; the posterior semi-circular canal swings outward, reducing the angle (in dorsal view) between anterior and posterior canals. The functional/sensory significance of these transformations (if any) is unknown, but both conditions are also evident in extant chimaeroids.

*Maghriboselache*, however, exhibits little of the more derived states of these endocranial conditions exemplified by ‘standard’ symmoriiforms. But, in one prominent detail the cranium is quite exceptional: the breadth of the snout and lateral separation of the nasal capsules. As far as the authors are aware, this is the earliest chondrichthyan known with such a widely separated and generously proportioned pair of nasal capsules (Figs.5, 6). This feature is unknown in other contemporary or subsequent Paleozoic sharks and might even be the earliest instance in all gnathostomes; there is some evidence that the slightly younger sister genus *Cladoselache* had a similarly broad nasal region (Dean, 1909a: pl. 28). Where preserved, other symmoriiforms have quite the opposite condition: a laterally narrow and acute rostrum, as suggested by skeletal remains (Coates et al., 2017; Frey et al., 2019; Pradel et al., 2014; Williams, 1985) and represented by soft tissue outlines preserved in Bear Gulch sharks (Lund, 1985). In this respect, at least, we might be obtaining some insight into a previously unknown defining specialization of cladoselachians, departing from general trends towards more derived holocephalan total group conditions. What, then, might be the functional, sensory, significance of this novel morphology in *Maghriboselache*?

**Fig. 15:**
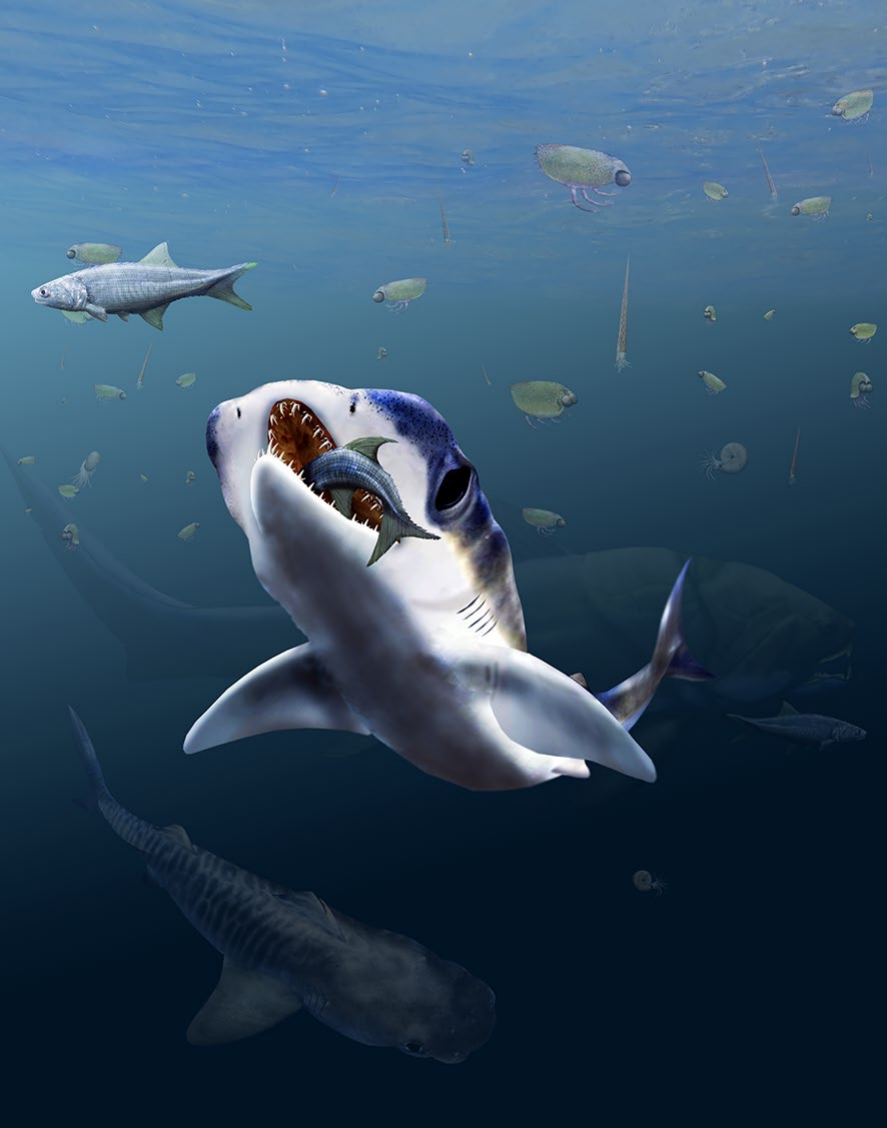
Reconstruction of *Maghriboselache mohamezanei* n. gen. et sp. in its habitat. Thylacocephalans, cephalopods with orthoconic conchs, placoderms such as *Dunkleosteus* and osteichthyans populated the water column during the middle Famennian in the Anti-Atlas

Compared to similarly sized teleost fishes or chimaeroid holocephalans, modern elasmobranchs have rather large nasal capsules. This characteristic has been linked to olfactory performance, with the suggestion that elasmobranchs are peculiarly sensitive to chemical cues. However, this has been challenged: the form-function link has proved difficult to demonstrate (Ferrando et al., 2019; Meredith & Kajiura, 2010) based on chemical cue sensitivity relative to elasmobranch nasal rosette surface area estimates and lamellar counts. Separation of the nasal capsules might provide a more instructive morphological correlate of olfactory specialization. In hammerhead sharks (carcharhiniforms, Sphyrnidae, e.g., *Sphyrna*), the laterally expanded and dorsoventrally compressed cranium, the ‘cephalofoil’, supports similarly separated, but individually low and extremely wide, nasal capsules. It is argued that the cephalofoil serves two functions linked to olfaction: to sample a wider swath of water, and to enhance olfactory klinotaxis (Kajiura et al., 2005). The first function increases the chance of sampling an odor molecule, and the second provides a means for ‘stereo-olfaction’ and thus ability to compare signal strengths from left and right nasal rosettes and orient accordingly. Somewhat similar, the head of the spadenose shark (*Scoliodon*) has rounded broad and dorsoventrally flattened rostrum with very widely separated narial openings. *Maghriboselache* (Fig. 15) is morphologically remote from the extreme cranial specialization of hammerheads, although somewhat closer to the arrangement in spadenose sharks; nevertheless, it provides the earliest evidence of a rostral skeleton approaching these conditions: dorsoventrally flattened, wide, and with laterally displaced olfactory organs. The suggested sensory specialization provides the first indication of a previously unconsidered dimension of ecomorphological diversity among early chondrichthyans. Moreover, as implied earlier, this represents an early diversion from the sensory specializations more generally associated with other members of the holocephalan lineage (e.g., increased orbit size, Coates et al., 2017).

## Conclusions

Here, we describe well-preserved skeletons of the new symmoriiform *Maghriboselache mohamezanei* gen. et sp. n. from the Famennian of the Moroccan Anti-Atlas. Highlights of the fossil material include three-dimensional cranial remains, and traces of postcranial soft tissues including internal organs and parts of the integument. Significantly, this is the first *Cladoselache*-like symmoriiform to yield substantial details of the jaws and braincase, including endocranial detail. With *Cladoselache*, *Maghriboselache* shares a highly distinctive tooth morphology (each tooth base includes a deep basolabial depression with adjacent projections), a characteristic upper jaw shape (the quadrate process is shorter than the palatine process), and specialized pectoral fin structure (supported by strap-like radials reaching the fin perimeter). With symmoriiforms, *Maghriboselache* shares a high aspect ratio tail and a braincase with a narrow-waisted basicranium, and with holocephalans in general, *Maghriboselache* shares a braincase with a greater ethmosphenoid unit bearing large orbits and a shorter, compact, closed, otico-occipital unit. But, *Maghriboselache* is also set apart, distinguished from all other early sharks, by the possession of a spectacularly broad, cartilage-enclosed, snout with widely spaced, large nasal capsules.

The geological age of *Maghriboselache*, alongside *Ferromirum* (Frey et al., 2020a), pegs the early evolutionary radiation of the symmoriiforms and holocephalan clade in general to at least the mid-Famennian, and a divergence time estimate projects the chondrichthyan crown-node into the Eifelian (Middle Devonian). This date estimate is corroborated by the rich record of well-documented, Devonian, chondrichthyan isolated teeth, dental plates, scales, and spines. Importantly, none of the crown-associated items among these collections exceeds Givetian dates. But these same data also imply that numerous lineages better known from Carboniferous material can be drawn back into at least the Late Devonian. During the Late Devonian, a significant diversification in terms of taxa and traits happened (e.g., Klug et al., 2010), establishing a series of phylogenetic fuses for the post-Devonian chondrichthyan evolutionary radiation.

In terms of traits and implied functions, *Maghriboselache* consolidates previously proposed trends and reveals unexpected innovation among the earliest members of the holocephalan total group. Sediments of Late Devonian age are the source of the first chondrichthyans (and, indeed crown group gnathostomes) with high aspect ratio tails, and the first with dental plates. Further to this, *Maghriboselache* provides evidence of the stepwise remodeling of neurocranial morphology to accommodate large orbits. However, an unanticipated sensory innovation is manifest in the broad rostrum and nasal capsules. For chondrichthyans, if not other modern gnathostome divisions, it is increasingly apparent that the major new varieties of body plan were established in ecosystems dominated by extinct grades and clades, and thus ready-made to repopulate aquatic systems in the aftermath of the End Devonian biotic crisis.

## Supplementary Information

**Additional file 1.** Supplementary information including Supplementary Table 1 with a list of includedlist of specimens, detailed description of specimens, remarks on phylogenetic analyses, taxon and character lists,Supplementary Figs. 1 to 55, and supplementary references.

**Additional file 2.** Excel sheet withdata for head height/ body length ratio of all taxa included in the phylogenetic analyses as used for character 234.

**Additional file 3.** Excel sheet with data for nasal capsule width/ head length ratio of all taxaincluded in the phylogenetic analyses as used for character 237.

**Additional file 4.** Excel sheetwith data for orbit length relative to neurocranium length ratio of all taxa included in the phylogenetic analyses asused for character 236.

**Additional file 5.** Excel sheet with data for pectoral fin length relative tobody length ratio of all taxa included in the phylogenetic analyses as used for character 235.

**Additional file 6.** Excel sheet with data for postorbital to preorbital length ratio (position of the orbit within theneurocranium) of all taxa included in the phylogenetic analyses as used for character 238.

**Additional file 7.** This matrix is based on Frey et al. (2020), which is based on Coates et al. (2017). Weadditionally coded *Dracopristis hoffmanorum* Hodnett et al., 2021. Further, we added eightcharacters describing body proportions etc. For details see the character list, characters 231 to 238. We further addedcharacters 239 to 261 from Hodnett et al. (30), which are predominantly dental characters. The latter caused a biastowards dental characters.

**Additional file 8.** Nexus file. This is the same matrix asAdditional file 7, but without the characters 239 to 261 from Hodnett et al. (2021).

**Additional file 9.** Nexus file. This is the same matrix as Additional file 8, but without the new characters 231 to 238 andwithout characters 239 to 261 from Hodnett et al. (2021).

**Additional file 10.** Nexus file.This is largely the same matrix as Additional file 7, but with only selected characters from 231 to 261.

## Data Availability

All the fossil specimens were deposited at the Cadi-Ayyad University, Marrakesh (Morocco) with numbers combined with letters beginning with AA. All other specimens are stored at the Paleontological Institute at the University of Zurich (PIMUZ-specimens). The supporting computer tomographic data are available at Morphosource with the link https://www.morphosource.org/projects/000492799?locale=en . All data needed to evaluate the conclusions in the paper are present in the paper and/ or the Supplementary Materials. Additional data related to this paper may be requested from the authors.
