# Supplementary material for: Broad snouted cladoselachian with sensory specialization at the base of modern chondrichthyans
Source: Swiss J Palaeontol. 2023 Mar 28;142(1):2. doi: 10.1186/s13358-023-00266-6 (PMC10050047; doi:10.1186/s13358-023-00266-6)
Supplement: Supplementary file 1 — Additional file 1. Supplementary information including Supplementary Table 1 with a list of includedlist of specimens, detailed description of specimens, remarks on phylogenetic analyses, taxon and character lists,Supplementary Figs. 1 to 55, and supplementary references. [file 13358_2023_266_MOESM1_ESM.pdf]

Supplementary Materials for

Broad snouted cladoselachian with sensory specialization at the base of modern chondrichthyans

Christian Klug, Michael Coates, Linda Frey, Merle Greif, Melina Jobbins, Alexander Pohle, Abdelouahed Lagnaoui, Wahiba Bel Haouz & Michal Ginter

Correspondence to: chklug@pim.uzh.ch, mcoates@uchicago.edu

This PDF file includes:

Taphonomy

Supplementary Table 1 (list of specimens)

Description of specimens

Remarks on phylogenetic analyses

Taxon and Character lists

Supplementary Figs. 1 to 55

Taphonomy

Skeletons and skulls of *Maghriboselache* were found in various localities of the southern Maïder and Tafilalt (Moroccan Anti-Atlas). Skeletal remains of three other chondrichthyan taxa were found in that region. *Phoebodus* is already known, and its remains found in the Maider were described by (Frey et al. 2019). A second taxon was newly introduced (Frey, Coates, et al. 2020) as *Ferromirum*. They identified the small individual as an early symmoriid. There are also large skeletal remains of *Ctenacanthus*, the description of which is in progress. Additional chondrichthyan species are limited to teeth and fin spines (Michal Ginter et al. 2002). Abundant placoderms (*Alienacanthus* sp., *Dunkleosteus* sp., *Titanichthys* sp., *Driscollaspis* sp. nov.) and sarcopterygians are also present in the Famennian strata of the same region. Remarkably, many of these latter taxa are also found as more or less complete skeletons.

The predominance of gnathostomes living in the water column coincides with the high abundance of pelagic invertebrates and with the deposition of reddish ferruginous rocks, both indicating an at least temporarily rather poorly oxygenated seafloor during the Famennian at Madene (Frey et al. 2018). All specimens are preserved in red ferruginous nodules. The concretions contain skeletal remains ranging from partially preserved animal to nearly complete skeletons. The preservation condition is exceptional with articulated skeletal elements that occasionally include mineralized soft tissues such as internal organs, body outline, muscle tissue, and even gut content. In mineralized muscle tissues, it is possible to see histological details including muscle fiber striations and myomeric muscle segments. In most specimens, these muscle fibers are preserved in hematite (Frey, Pohle, et al. 2020) except for the only skeleton available from the Tafilalt, where most of the soft parts are phosphatized. The pattern of preservation is reminiscent of that of *Cladoselache* (B. Dean 1909) from the Cleveland Shale of Ohio.

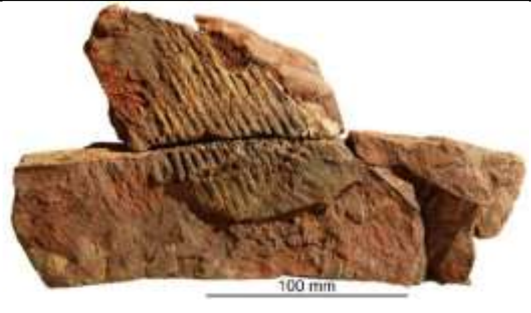

Supplementary Fig. 1. Posterior dorsal fin of the holotype AA.MEM.DS.12, which penetrates the nodule downward at an angle of about 45°.

Like in *Cladoselache*, the cartilaginous remains lie generally in one single plane, while the dorsal fins appear sometimes in a vertical plane and can be traced through the matrix (Supplementary Fig. 1). However, while *Cladoselache* is usually found in dorsal or ventral aspect (*74*, *75*)(B. Dean 1909), our specimens appear sometimes in an oblique or lateral position (Fig. 1). We speculate that this dorsal position is linked with the formation of putrefaction gases in the digestive tract turning the belly more or less upward (Elder and Smith 1988; Reisdorf et al. 2012). The sediment allowed the dorsal fin to sink into the sediment, while the large pectoral fins stabilized this position.

Probably due to the early diagenetic formation of ferruginous nodules incorporating the fossil to varying degrees (Frey, Pohle, et al. 2020), these chondrichthyan fossils are less compacted and thus less deformed than their American counterparts from the Famennian Cleveland Shale. This explains why we find moderately abundant 3D-preserved neurocrania of chondrichthyans in the Thylacocephalan Layer (Frey et al. 2019; Frey, Pohle, et al. 2020; Jobbins et al. 2020) and other parts of the Famennian. Correspondingly, the neurocranium of *Cladoselache* is still poorly known (B. Dean 1909), while much of the head of *Maghriboselache* is now known. Additionally, this semi-3D-preservation allowed the nearly vertical preservation of the dorsal fins in several cases (Supplementary Table 1).

List of included specimens with information on preservation

Specimen Number	3D cranium	Teeth	Complete head	Gills	Pectorals	Pelvic fin	Dorsal fin	Caudal	Soft parts	Skin
PIMUZ A/I 5152		x	x	x	(x)					
AA.MEM.DS.12	x	x	x	x	x			x		
AA.MEM.DS.6										
PIMUZ A/I 5153			x	x	x	x	x	x	x	
PIMUZ A/I 5154		x	x	x	x	x	x		x	x
PIMUZ A/I 5155			x	x	x	x	x		x	x
AA.BER.DS.01			x		x	x	x	x		
PIMUZ A/I 5156		x	x	x	x	x	x		x	
PIMUZ A/I 5157					x	x	x	x	x	x
PIMUZ A/I 5158		x	x	x	x	x	x		x	x
PIMUZ A/I 5159	x	x	x	x						
PIMUZ A/I 5160		x	x							
PIMUZ A/I 5161		x	x							
PIMUZ A/I 5162		x	x							
AA.TJR.DS.1		x	x							
AA.MEM.DS.7		x	x							
PIMUZ A/I 5163								x		

Table S1. List of specimens included in this study. PIMUZ-specimens are stored at the Paleontological Institute at the University of Zurich, All other numbers (AA. etc.) are stored at the Cadi-Ayyad University, Marrakesh (Morocco).

Descriptions of skeletons ordered by size

PIMUZ A/I 5152 (Supplementary Fig. 2)

Locality: Madene El Mrakib, southern Maïder, N30°45.056’, W4°42.829’

Stratigraphic position: Thylacocephalan Layer,

Finder: Saïd Oukherbouch (Tafraoute)

Preparator: Ben Pabst (Zürich)

Description: The most distinctive feature of this specimen is its size. The total length of the nodule is approximately 164 cm, but the relative proportions of the elements present suggest that the individual was much longer. Although most of the animal’s body is not preserved, the skull measures almost 20% of the total length of the slab, excluding the branchial arches. When comparing the skull with this of the other skeletons, we estimate a body length exceeding two meters, potentially reaching 2.5 meters. The skull is exposed in ventral view, and a number of its elements can be seen in great detail not available in the other specimens. Elements of the pectoral girdle, the pectoral and pelvic fins are also preserved.

The total skull length is 335 mm, from the most anterior to the most posterior elements. The neurocranium is approximately 250 mm long, the Meckel’s cartilages are approximately 300 mm long and the left palatoquadrate measures about 320 mm in length. The cartilages are only slightly deformed. Its light beige coloration suggests the presence of limonite, which distinguishes the cartilage optically from the hematite-red matrix. Except by the presence of a number of fractures, the different elements of the skull are quite well preserved and articulated. The skull is exposed in ventral view, but slightly compressed. The most noticeable elements are the Meckel’s cartilages (Supplementary Fig. 2). These two robust elements have collapsed medially exposing their labial sides, which are deeply concave. Due to compaction, they lost the primary laterally convex curvature. Extending medially, parallel to the midline, the ventrolateral mandibular ridge is distinctly noticeable, particularly on the left Meckel’s cartilage. The left palatoquadrate is also discernible in lateral view due to its lateral rotation along its longitudinal axis. The palatine ramus projects antero-ventrally, with an incomplete anterior end. Posteriorly, the palatoquadrate displays the complete otic process.

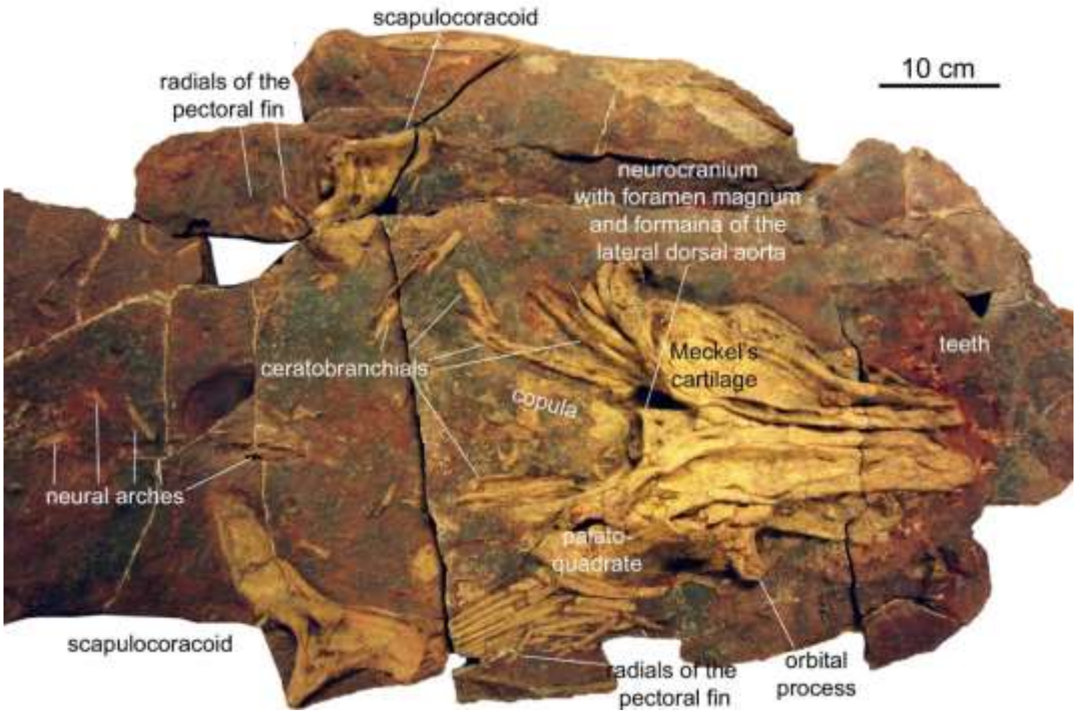

Supplementary Fig. 2 Overview of the incomplete specimen PIMUZ A/I 5152. Note the slightly compacted skull. Both Meckel's cartilages are well visible as well as a few teeth scattered around the snout. This is the specimens with the largest of this species.

Due to the position of the Meckel's cartilages, the chondrocranium is not accessible, except for a few details. The left postorbital process extends beyond the lateral limit of the left Meckel's cartilage. The most posterior part of the neurocranium can also be seen between the Meckel's cartilages. The occipital region shows two posterior foramina for the lateral dorsal aorta. The foramen magnum has collapsed, but in posterior view, the occipital condyle is exposed. Extending posteriorly beyond the Meckel's cartilage is the lateral otic process (Supplementary Fig. 2), a robust process that curves laterally. The branchial arches are partially preserved and in situ. Proportionally to the larger size of this animal, the branchial arches are long and robust. Approximately three ceratobranchials extend in an anteromedial-posterolateral direction, on each side. They are up to 120 mm long and 20 mm wide, with a strongly convex side and a spatulate posterolateral end. Between them, flat and elongate oval copula is apparent, which is 93 mm long, although incomplete, and 40 mm wide, at its widest point.

The dentition much resembles that of AA.MEM.DS.12; one central main cusp is flanked by one relatively smaller cusp on each side and even finer cusplets in between. The main cusp is

conical, with a circular cross section and a sharply pointed tip. The lingual side of both the tooth base and the main cusp are concave. The lateral cusps are much smaller in relation to the central one. In comparison to AA.MEM.DS.12, however, the teeth show a proportionally smaller size in relation to the skull. We have measured the tooth base of one well preserved tooth of each specimen, and while Ben's neurocranium is 68.5% the length of Skully's, the measured tooth of the former is 90% the length of the latter. This shows that there is not much difference in tooth size for a difference of 78.8 mm between the size of the two neurocrania.

Further postcranial material present is restricted to elements of the pectoral girdle and fins, some displaced neural arches, and a questionable dorsal fin spine. The left scapulocoracoid is relatively well preserved and, although fractured, it is complete. It is 194 mm long, measured diagonally between its proximal and distal most points. The base (proximal end) is 25.9 mm wide, and the distal portion measures 107 mm. The narrow medial shaft is quite robust in this specimen and tapers only slightly medially. It widens progressively in a lateral direction. The wider lateral portion has a concave lateral side for the articulation with the pectoral fin. In this specimen, the posterior side of the scapulocoracoid is concave. The left pectoral fin preserves some of its radial elements. The fin is taphonomically separated from the pectoral girdle with the radials positioned anterior to the latter instead of extending laterally. The radials are long and slender, but again more robust than in the specimens previously described. The longest radials are nearly 200 mm long. Of the right pectoral appendage, only a partial and fractured scapulocoracoid is preserved, as well as few radial elements of the right fin dispersed in its proximity.

The neural arches are slim, elongated elements (Supplementary Fig. 2). They are usually ventrally bifurcated, and frequently fragmented with only one of the two rami. They reach a length of about 40 mm.

Medial to the base of the left scapulocoracoid, extending along the length of the animal, is an elongated fragment of 65 mm whose structure and position is suggestive of a dorsal fin spine. The poor condition of preservation, and the limited exposure, does not allow for a more detailed and conclusive identification of these elements.

AA.MEM.DS.12 (Supplementary Fig. 3 to 7)

Locality: Mousgar, southern Maïder, N30°46.557’, W4°41.257’

Stratigraphic position: Thylacocephalan Layer, early Middle Famennian.

Finder: Saïd Oukherbouch (Tafraoute)

Preparator: Ben Pabst (Zürich)

Description: This is the largest, more or less complete specimen. The complete slab measures about 180 cm in length (Supplementary Fig. 3). The skeleton is exposed in ventral position and has a kink in the middle. Taking this into account, the animal might have reached a body length of nearly two meters. The left pectoral fin is nearly completely preserved, while the right pectoral fin is incomplete and disarticulated. Most of the dorsal fin lies on the left side, approximately in the middle of the skeleton. This specimen preserves parts of the caudal fin. The pelvic fins and pectoral girdle are discernible, partially preserved and disarticulated.

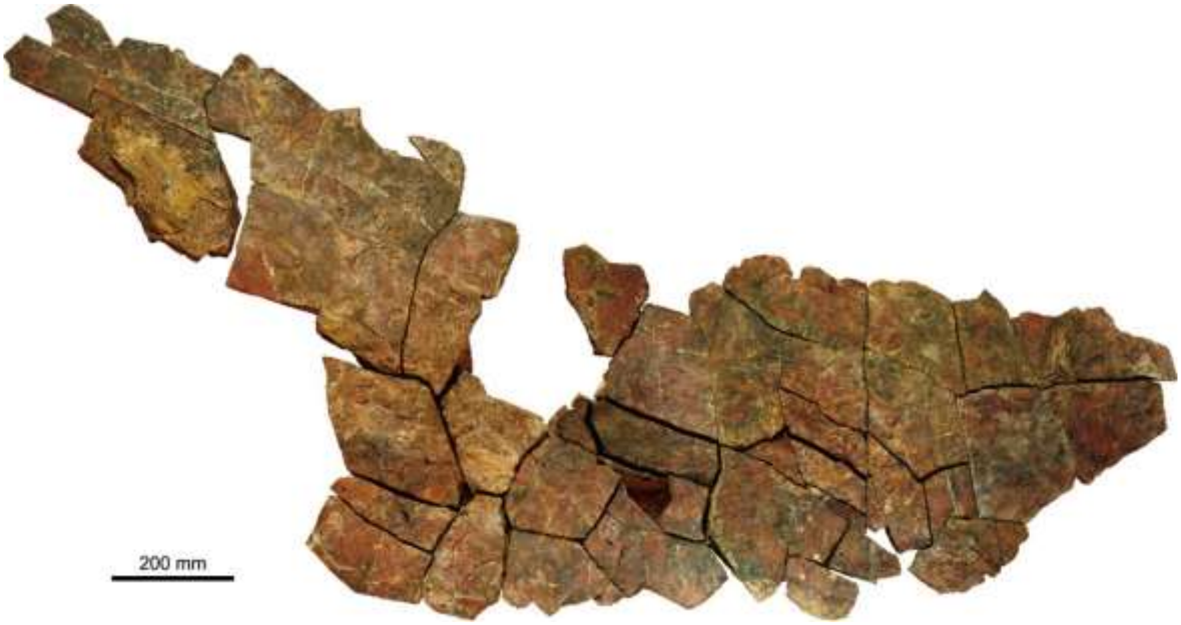

Supplementary Fig. 3 Overview of the incomplete specimen AA.MEM.DS.12. Note the weathered skull on the left, which still preserved most of the neurocranium three-dimensionally. The left pectoral, the posterior dorsal and the caudal fins are preserved. This is one of the largest specimens of this species available for this study.

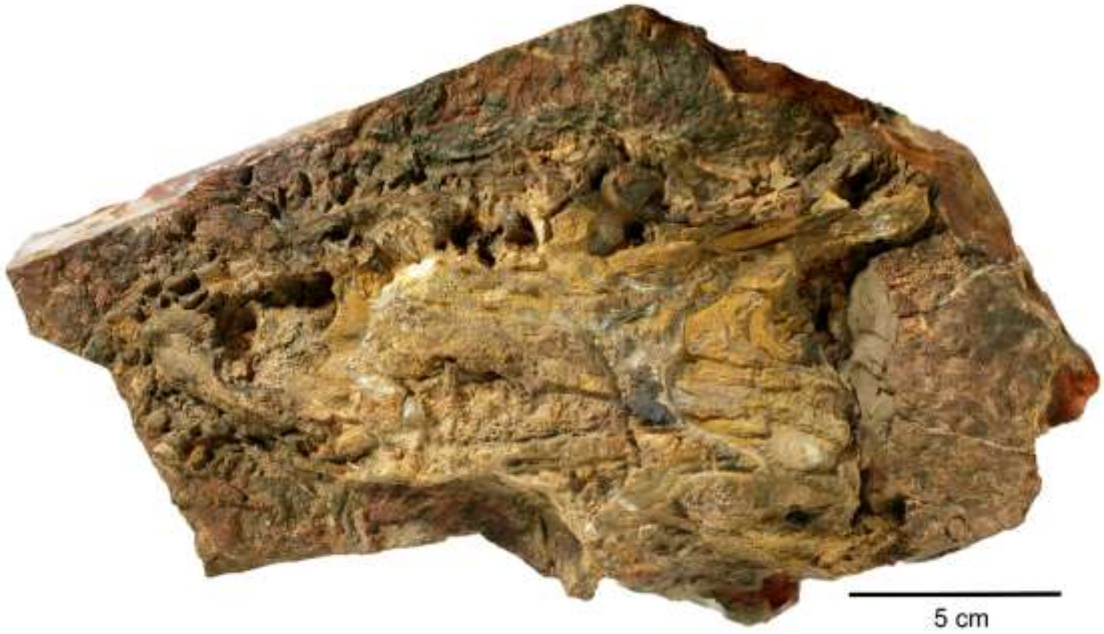

Supplementary Fig. 4 Skull of the incomplete specimen AA.MEM.DS.12 seen from the ventral side. The dentition is partially preserved. Much of the venter of the neurocranium is preserved. For the segmented CT-data, see Fig. 2 to 4.

The skull is 188 mm long (Supplementary Fig. 4, 7). Although weathered and locally fractured, it preserves the chondrocranium and parts of the mandibular arch quite well in three dimensions. Exposed in ventral view, the tooth-bearing Meckel's cartilages flank the chondrocranium. Part of the dentition is exposed and moderately well preserved. The cladodont teeth are robust with a wide base that sustains three larger cusps with minute cusps in between. The main, central cusp is long and conical with a circular cross-section and a pointed tip; the lingual side is concave. Two comparatively very small cusps flank the central one on each side. They are conical, and have a sharp pointed tip. In the anterior most part of the skull, an articulated tooth family can be seen. Posterior to the skull, a few branchial arches are present on the left side. Due to the position of the skull and its weathered surface, part of the braincase is visible in ventral aspect (the carcass was embedded with the ventral side facing obliquely upwards).

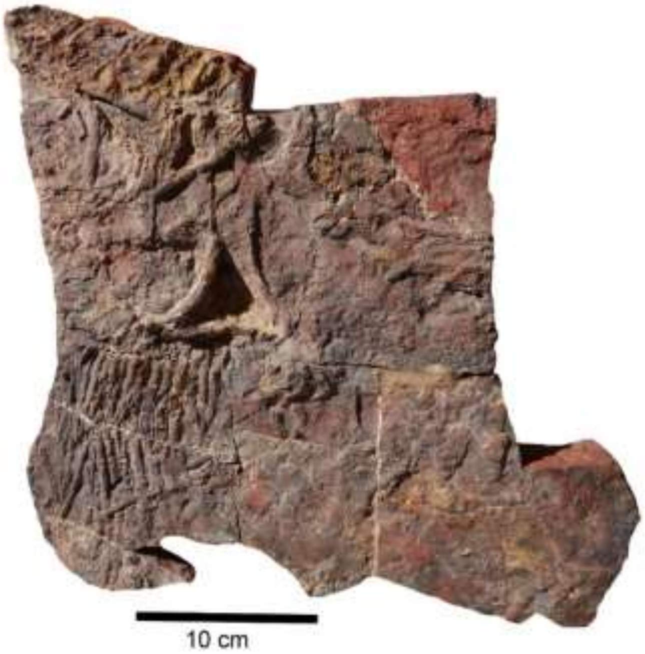

Supplementary Fig. 5 Pectoral girdle and fin of *Maghriboselache* AA.MEM.DS.12. Note how the radials are still partially inserted in the scapulocoracoid.

The left scapulocoracoid (Supplementary Fig. 5) is present on the right, due to the ventral side up-position of the animal, and measures 80 mm in length. The medial shaft is partially preserved. The distal portion is expanded antero-distally forming a broad base for the articulation with the radials of the pectoral fin slightly displaced postero-ventrally from the articular ridge. The most distal side of the scapulocoracoid is slightly concave and the surface of this element is deeply excavated. The procoracoid cannot be discerned. The left pectoral fin is displaced posteriorly. The proximal and distal radials extend laterallyto the scapulocoracoid, consecutively. Some of the longer distal radials are incomplete, but all the elements are articulated. The fin is preserved over a total length of 186.5 mm and a width of 81.7 mm at its base. Probably, it was actually about 200 mm long. The pelvic girdle is not preserved, but some disarticulated radial elements of the pelvic fins are dispersed on the right side of the specimen, roughly in the middle of the specimen.

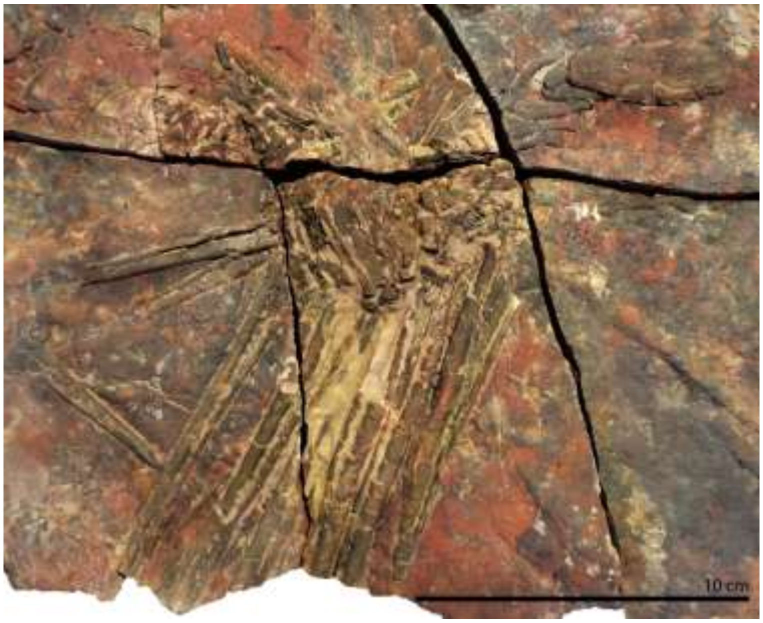

Supplementary Fig. 6 Ventral lobe of caudal fin of *Maghriboselache* AA.MEM.DS.12. Note the dorsal plate at the base of the fin.

The caudal fin is incomplete but relatively well articulated (Supplementary Fig. 6). It measures 127.5 mm at its longer axis. In the proximal part of the caudal fin, it displays its neural and haemal arches. Both are slender elements, short, and slightly curved. They are oriented in opposite directions, forming a chevron-pattern pointing posteriorly. Distally, more or less articulated supraneural radials, haemal spines, and hypochordal radials are visible from left to right of the slab (i.e. right to left of the individual). The supraneural radials are longer than the neural and haemal arches, and show no curvature. The haemal spines are slightly shorter than the supraneural radials, and anteriorly concave. The hypochordal radials extend distally to the haemal spines and reach a length of 110 mm. They are slender, straight elements, extending diagonally in an anteroposterior direction. There are about 13 hypochordal radials discernible, but there might have been more. The distal ends of the supraneural and hypochordal radials are oriented posteriorly. The hypochordal radials are still articulated with the haemal arches, which increase from about 10 mm at the anterior end to about 35 mm in the middle of the ventral lobe of the fin. At the base of the caudal fin, the haemal arches have a rounded v-shapeand are about 25 mm high. Above these haemal arches, there lies an elongate element, which measures 50 x 18 mm. A similar plate is likely present in *Akomonistion* ((M. I. Coates and Sequeira 1998, 2001) Fig. 9A), although it is not shown in the reconstruction in their figure 15. This is not an artefact,

since this element is present in several specimens of *Maghriboselache*. Only a few disarticulated supraneural radials are preserved and most of the dorsal lobe of the caudal fin is missing.

The posterior dorsal fin is nearly completely preserved on the left side of the specimen at mid body (Supplementary Fig. 1). The dorsal fin projects downwards into the sediment, evincing that the fin sank into the then still soft sediment as the carcass laid to rest. Proximally, at the base of the fin, the basal basal cartilage is discernible. It is only partially preserved and rests more or less in the bedding plane that holds most of the skeleton. The proximal radials are up to 20 mm long and extend distally to the basal plate, in the bedding plane. The distal radials are up to 80 mm long and directed downward into the sediment and reach the base of the nodule, where they are bent backwards, possibly by differential compaction and mineralization. The total length of the dorsal fin is 133.04 mm. There is no evidence of the presence of an anterior fin spine or fin and only questionable remains of the fin spine of the posterior fin are present.

Very rarely, small portions of soft tissue are preserved. From their structure, these represents small pieces of taphonomically mineralized musculature and questionable cololites or stomach contents. Only small patches of integument are preserved around the skull.

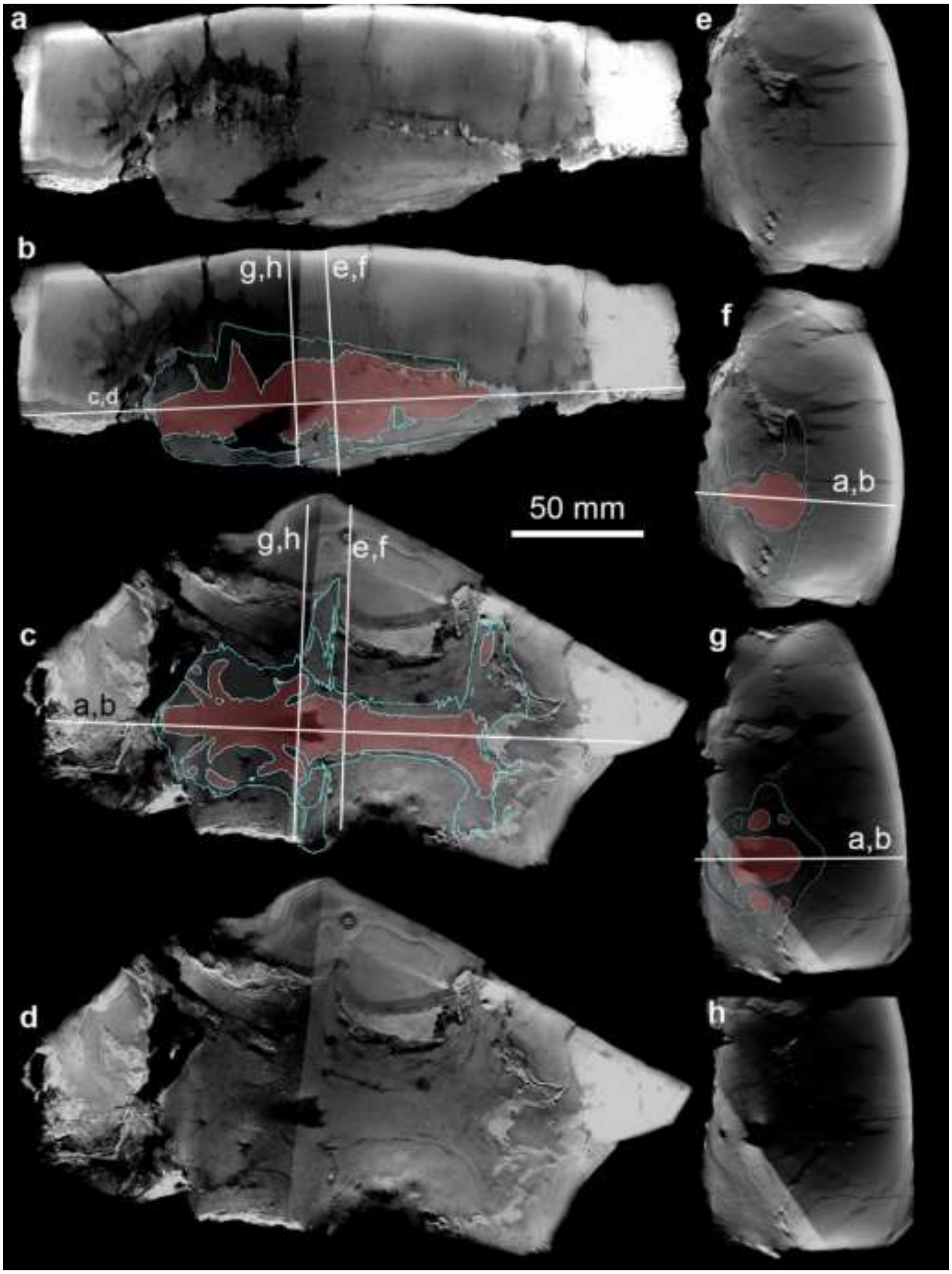

Supplementary Fig. 7 Tomographic slices of the holotype of *Maghriboselache* AA.MEM.DS.12. a, d, e, h slices shown without masks. b, c, f, g slices shown with masks of the neurocranium (bluish outline) and the endocast (reddish surfaces). a, b section in the plane of symmetry. c, d horizontal section. e to h, coronal sections. Placement of sections indicated by white lines and references to the respective image (a to h).

AA.MEM.DS.6 (Supplementary Fig. 8)

Locality: Madene El Mrakib, southern Maïder

Stratigraphic position: Thylacocephalan Layer, early Middle Famennian.

Finder: Saïd Oukherbouch (Tafraoute)

Preparator: Merle Greif (Winterthur)

Description: This specimen is positioned in oblique ventral view. The cartilaginous remains are about two meters long including skull and caudal fin. The skull remains measure about 240 mm in length. The skull, although complete, is heavily fractured and dorso-ventrally compressed. Only parts of the neurocranium can be discerned (Supplementary Fig. 8), except part of the basal plate between the splanchnocranial elements. Of the splanchnocranium, the palatoquadrate can be seen forming an elongated arch with the vertex at the anterior end of the snout (205 mm). The Meckel's cartilage (230 mm) is fractured and rotated laterally exposing the medial side, particularly on the left side of the skull. The palatoquadrate is 205 mm long and also heavily fractured. The hyoid arch is present as the ceratobranchial is partially preserved just posterior to the skull (Supplementary Fig. 8). This element is a relatively thick cartilaginous rod compared to the branchial arches. Fragments of the branchial arches are present posterior to the ceratohyal. They measure roughly 51 mm in length, are heavily fractured and incomplete.

A few teeth are scattered just anterior to the snout. The base of the teeth is curved and kidney-shaped. The main cusp is central with a circular cross section, a relatively wide base and sharply pointed tip. Otherwise, the dentition has a cladodont appearance.

Of the pectoral girdle, the left procoracoid and scapulocoracoid are present, both damaged and incomplete. The sickle-shaped procoracoid is 35.4 mm long, and the fractured remains of the scapulocoracoid are 92.5 mm. The scapulocoracoid articulates with the left pectoral fin. It measures 172.3 mm long with a 89.7 mm wide base. The long and slender radials are disarticulated and some are overlapping each other. Probably, the right pectoral fin came to rest on the left pectoral fin. The right fin displays remains of ten radials, which are up to 200 mm long. The radials of the lower fin are partially covered. Remains of at least ten radials can be counted there as well.

The pelvic fin is only partially preserved. Its radials are scattered around the level of the pelvic girdle. The longest preserved radial measures about 100 mm. The pelvic girdle lieas slightly above the radials and is incomplete.

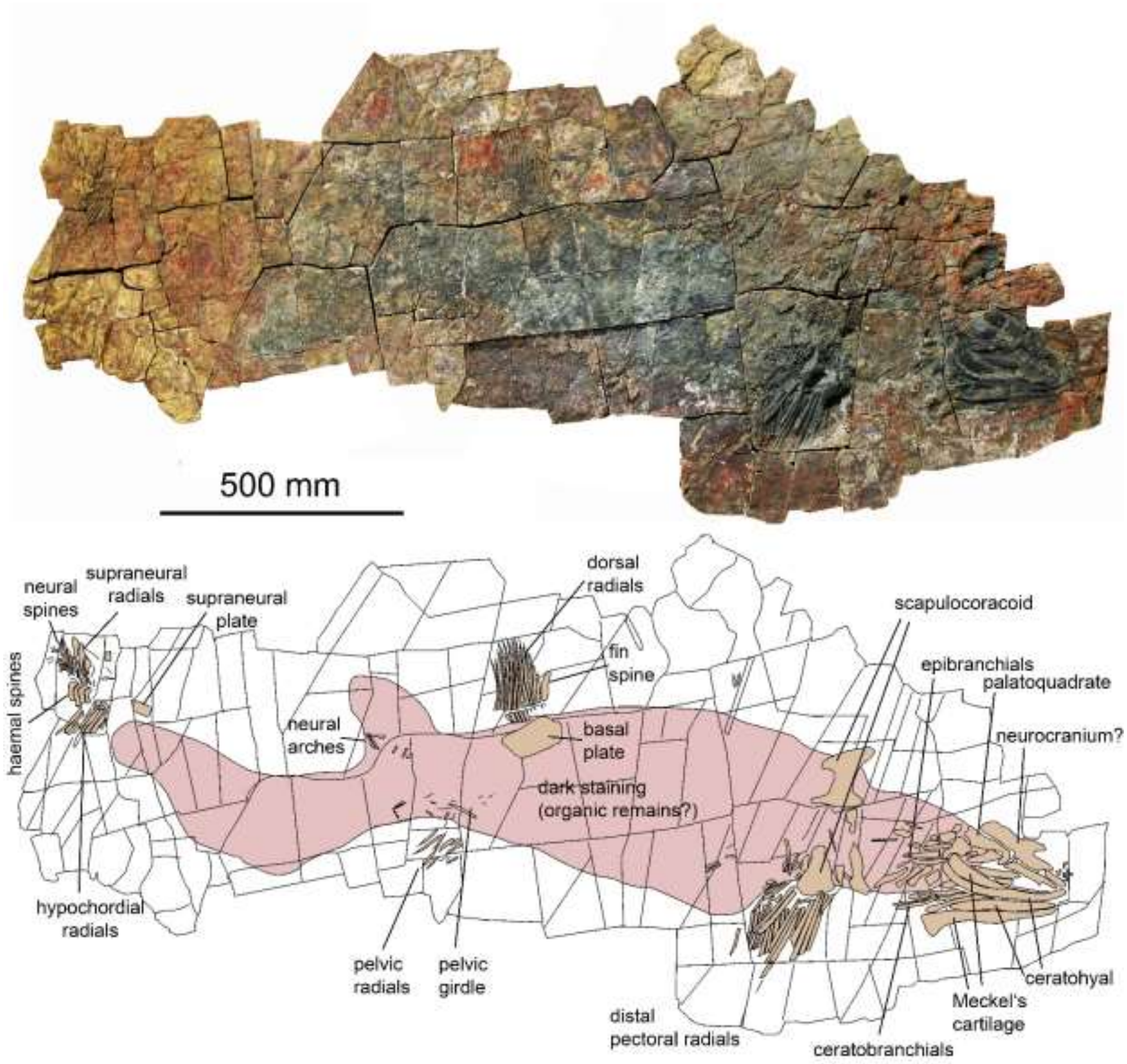

Supplementary Fig. 8 Overview of the specimen AA.MEM.DS.6. Note the slightly compacted skull. Both Meckel's cartilages are still in contact with the ceratohyals. This is the completest of the large specimens.

The caudal fin shows partial preservation of both dorsal and ventral lobes. It measures 83.8 mm along a straight line following the longitudinal axis of the body. Preserved elements of the dorsal lobe include the posteriormost neural spines (up to 39.4 mm long), slim and straight rods with a straight proximal end and a pointed distal end, and the haemal spines ventral to the neural spines. The haemal spines (40 mm) are at the base of the dorsal fin lobe and show a distinct curvature.

All these elements decrease in size in a posterior direction. Only five incomplete hypochordal radials of the ventral fin lobe are discernible, preserving only their proximal half, partially as imprint. The longest of these radials extends over nearly 100 mm, but is incomplete. The radials are straight and more robust than the elements on the dorsal lobe. The proximal end is straight and in contact with fragments of the dorsal haemal spines.

The posterior dorsal fin is particularly well preserved. It is 181 mm high and 85 mm long at the base. The radials lean slightly in a posterior direction and the general outline is lobed. The proximal radials are relatively short rods with straight ends and range between 15 and 22 mm in length. The distal radials are articulated with the proximal radials and are long rods with pointed distal ends. They range in length from 79 mm at the anterior and posterior ends to 116 mm at the center of the fin. Anterior to the dorsal fin, a dorsal fin spine is partially preserved. The fragment is 27.7 mm long.

Impressions of soft body tissue are present as black surfaces on the slab, generally close to the midline (Supplementary Fig. 8). Patches of the integument are sometimes visible close to the skull, around the dentition and the branchial arches. It consists of small multiodontode denticles. The denticles are slightly oval, more or less simple. They measure between 0.5 and 1 mm in length. They have a rhomboid outline and carry 3 to 4 longitudinal ridges.

PIMUZ A/I 5153 (Supplementary Fig. 9 to S10)

Locality: southeastern edge of Jebel Oufatene, southwestern Maïder, N30°47.057’, W4°53.476’

Stratigraphic position: Thylacocephalan Layer, early Middle Famennian.

Finder: Saïd Oukherbouch (Tafraoute)

Preparator: Christian Klug (Zürich) & Merle Greif (Winterthur)

Description: The slab is approximately 160 cm long, and the remains of the specimen cover about 140 cm. The articulated specimen includes the skull and most of the body. Most of the postcranium including remains of all fins, elements of the shoulder girdle are preserved as well as neural arches. The right pectoral fin is partially preserved, and articulated remains of both pelvic fins as well. The posterior dorsal fin is well visible and articulated, as well as both dorsal fin spines.

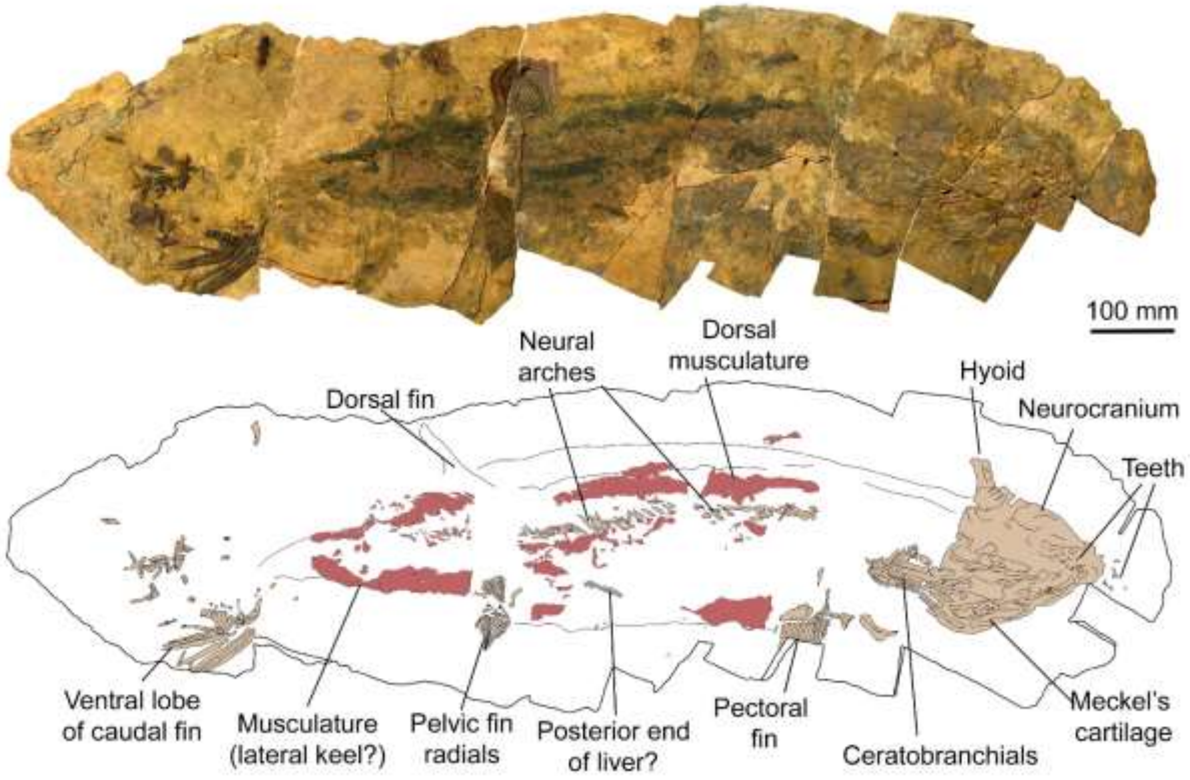

Supplementary Fig. 9 Two nearly complete skeletons of *Maghriboselache mohamezanei* n. gen. et sp., PIMUZ A/I 5153.

The skull, although fairly well articulated, is deeply weathered and compacted. The left Meckel’s cartilage measures 144 mm in length, although considerably fractured. Like in specimen PIMUZ

A/I 36884, the Meckel’s cartilages have rotated medially under the palatoquadrate, probably due to compaction. Thus, a detailed morphological description of the palatoquadrate and braincase will be provided based on other specimens, where these structures are better preserved. The branchial arches are visible posterior to the skull. They still show the original arrangement and position but due to their lateral position, the articulated branchial elements are partially superimposed, thus hampering the identification of all elements.

A tooth family is preserved anterior to the front of the snout; sometimes, only the outlines of the teeth are imprinted in the sediment. We have produced a latex cast of the skull to facilitate the examination of the dentition. The cast reveals details of the dentition showing a median cusp with a subcircular cross section, lingually concave. Flanking the main cusp, this specimen presents two lateral cusps. They are both much smaller in comparison with the main cusp. The outer cusps are roughly as long as their base is wide (approximately 0.5 mm). The intermediate cusps are quite small, delicate and therefore more rarely preserved.

The neural arches extend along the length of the body. They are less well organized than in PIMUZ A/I 36884 and slightly displaced from their original arrangement, but the tesserae of the cartilage are still well visible. Over 30 neural arches are discernible.

The scapulocoracoid resembles that of PIMUZ A/I 5153. The proximal process is, however, relatively narrower and expands abruptly into a somewhat triangular distal part. It measures 77.8 mm from the end of the proximal process to the base of the radials. The distal side is concave for insertion of the radials. Like most of the cartilaginous remains in this specimen, it is quite weathered. The sickle-shaped procoracoid is exposed anteriorly to the scapulocoracoid on the right side. Its proximal process is incomplete. Only the proximal part of a pectoral fin is preserved; remains of 11 or 12 proximal radials are well visible and in articulation with the scapulocoracoid.

Though no elements of the pelvic girdle are visible, both pelvic fins are present. They are slightly displaced, and measure approximately 42.5 mm at their longer axis. The radials are more delicate than those of the pectoral fins and of the posterior dorsal fin. They vary in length and converge distally in a fashion that confers a triangular shape to both fins.

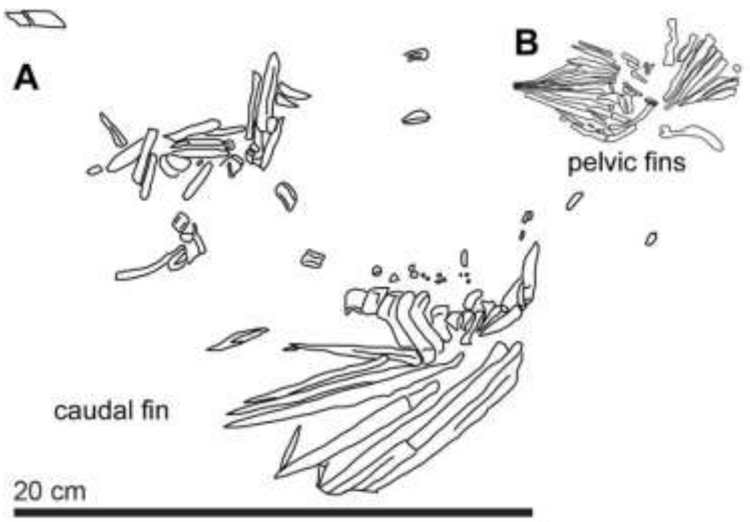

Supplementary Fig. 10
Caudal and pelvic fins of PIMUZ A/I 5153.
A Subdisarticulated caudal fin.
B Remains of the pelvic fins.

The caudal fin is slightly separated from the rest of the skeleton and subdisarticulated (Supplementary Fig. 10). There are eight hypochordial radials, partially still articulated with the haemal spines. There hypochordial radials are up to about 100 mm long and up to 7 mm wide. The haemal spines are short, curved elements about 30 mm long. Only isolated elements of the dorsal part of the tail are preserved.

The anterior dorsal fin spine is present. It is a sturdy element, which is 51.4 mm long and 25.29 mm wide at its base, giving it a broad triangular outline. It was deformed by differential compaction and the tip is broken, and slightly dislodged. The surface is fractured, but there is evidence of a primary very fine and shallow longitudinal striation. There is no evidence of serrations in this specimen. No radial elements of the anterior dorsal fin are present, which can be suggestive of an anterior dorsal fin constituted of only soft tissue as in *Ctenacanthus*.

The posterior dorsal fin is quite well preserved and articulated in a triangular shape. It measures 45.2 mm at its longer axis and it is 38.8 mm wide at the base. A posterior dorsal fin spine is also present. It is located at the anterior edge of the fin and measures 38.4 mm in length and 13 mm in width at the base. The posterior fin spine is not as robust as the anterior one, but like the latter, it has a pointed tip and it is curved posteriorly.

As is common among these exceptionally preserved specimens, hematitized soft tissues including musculature and other internal soft parts are discernible. In this specimen, the muscle tissue remains reveal the general outline of the body, which gives an idea about size and relative proportions of this animal. It is visible as long black bundles of muscle tissues, with diagonal segments possibly representing myomeres and the according fasciae. Anterior to the pelvic fins,

a slim and dark, longitudinally striated element, ventrally positioned and antero-ventrally oriented, likely represents the posterior end of the liver. It is about 50 mm long and 10 mm wide. Between the two dorsal fin spines, a millimeter-wide furrow extends dorsally of the neural arches, which might represent the poorly preserved lateral line. A convoluted structure of hematitized tissues is tentatively interpreted as a filling of the digestive tract. No traces of the integument, or other sensory organs are visible.

Half fish, PIMUZ A/I 5154 (Supplementary Fig. 11 to 13)

Locality: Bid Er Ras, southwestern Maïder, N30.694068684968°, W-4.915991949048543°

Stratigraphic position: Thylacocephalan Layer, early Middle Famennian.

Finder: Saïd Oukherbouch (Tafraoute)

Preparator: Merle Greif (Winterthur)

Description: This specimen rests on its dorsal side, exposing the preserved parts in ventral view. Although the skeleton is incomplete, some structures display remarkable details, namely the pectoral fins, and the anterior dorsal fin spine.

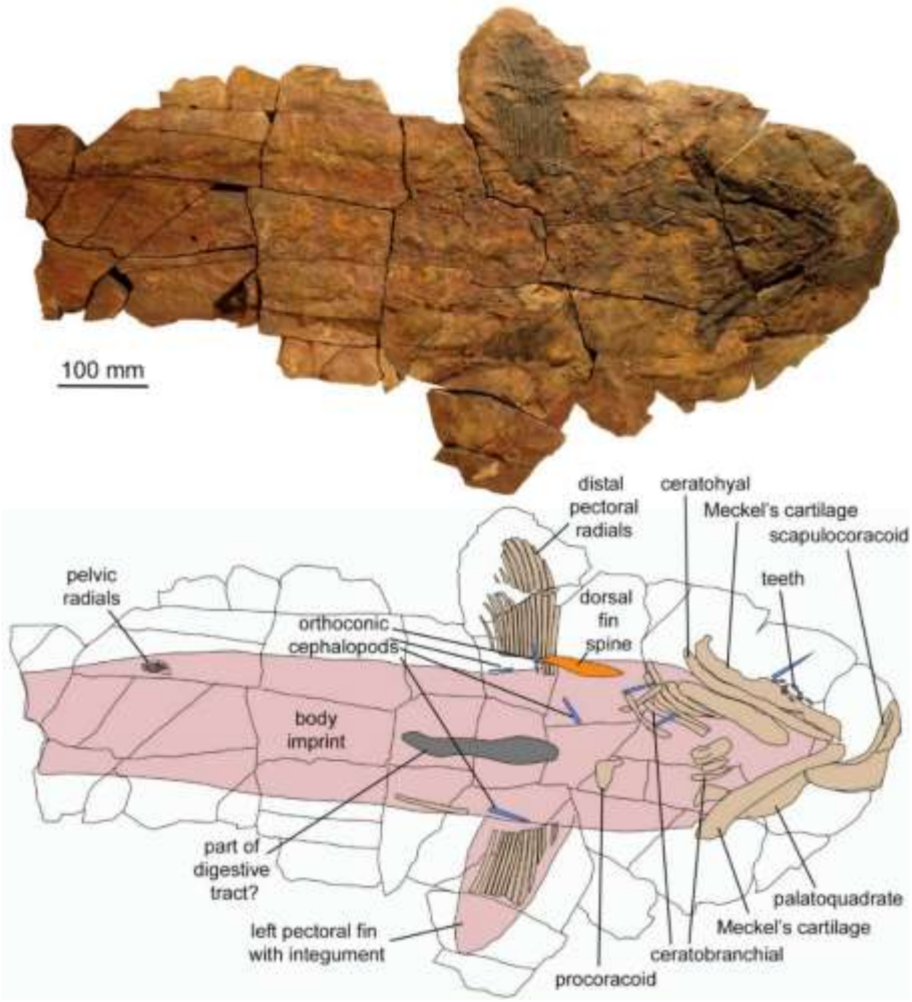

Supplementary Fig. 11 Overview over the incomplete skeleton of *Maghriboselache mohamezanei* n. gen. et sp., PIMUZ A/I 5154. The anterior part is well-preserved, while in the posterior portion, mainly the body outline can be seen. The left pectoral fin and the portion between the gills and pectoral fins preserves some integument.

The slab is one meter long with most skeletal remains corresponding to the anterior half of the skeleton, while the posterior part is heavily weathered and showing mainly the body outline and a few fin radials of the pelvic fin. The skull, much like the remaining skeletal elements, is dorsoventrally compressed. Its total length is 175 mm in the median plane. Both Meckel's cartilages are present (240 mm long), although compressed, fragmented, and rotated laterally. The furrow of the ventrolateral mandibular ridge extends along the longitudinal axis of these flat cartilages. Some parts of the palatoquadrate are exposed adjacent to the overlapping Meckel's cartilages.

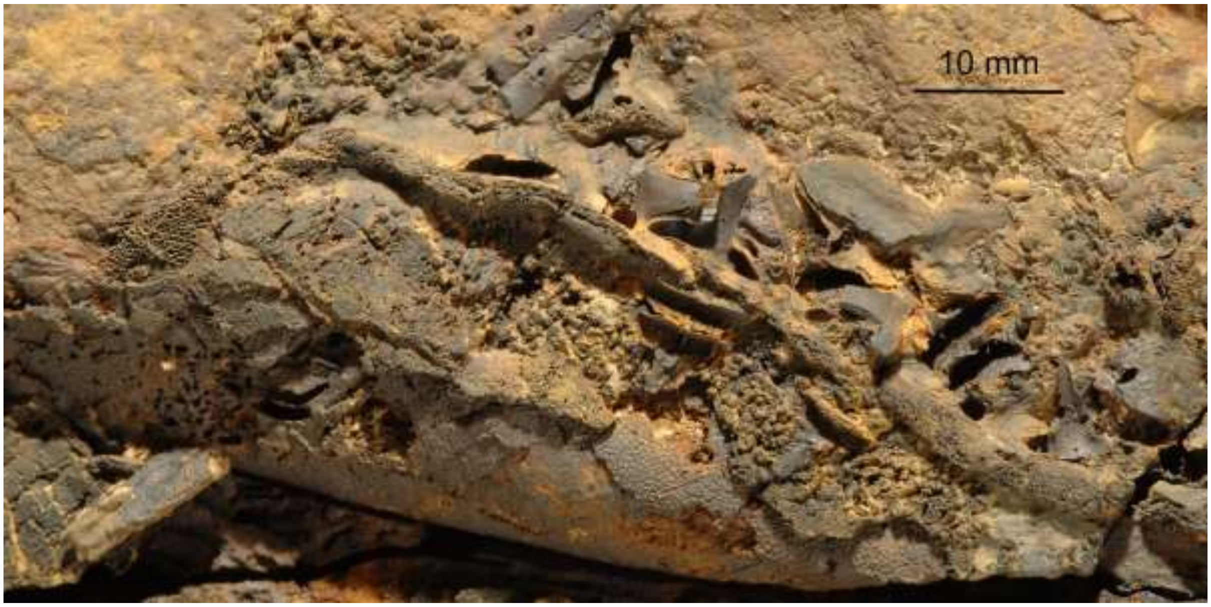

Supplementary Fig. 12 Right Meckel's cartilage, dentition and dermal denticles of *Maghriboselache* PIMUZ A/I 5154.

The branchial arches are scattered along a surface of 152 mm posterior to the skull. The present elements correspond to ceratobranchials, visible in an anteromedial to posterolateral orientation. The average ceratobranchial measures roughly 50 mm.

Remarkably, the anterior dorsal fin spine is preserved over the radials of the right pectoral fin. Astonishingly, this fin spine remained in a specimen where a taphonomic phenomenon displaced at least one of the scapulocoracoids anterior to the snout. The fin spine is an elongate subtriangular element, 118 mm long and 21 mm in cross section. As is common, the fin spine has an elongate but rather narrow base and a pointed tip with a posterior oriented curvature. It is

preserved as external cast, displaying a very fine pattern of blood vessel imprints and minute foramina, as well as about five denticles at the caudal edge of the spine (Fig. 10).

Of the shoulder girdle, only one scapulocoracoid remains, which was displaced from its original position to anterior to the skull. It is slightly incompletely preserved cropped by the limits of the slab. The scapulocoracoid is robust and laterally flattened. The scapular portion is broad and sturdy, broadening as it reaches the coracoid. These two elements meet in a sharp angle.

Both pectoral fins are completely preserved with articulated radials and in parts still covered by skin with dermal denticles *in situ* (Supplementary Fig. 13). The left pectoral fin is not less notable. All its proximal radials are present and articulated, and although the distal radial elements are not themselves preserved, their imprint is so detailed that we can see the entire fin with all the elements being distinguishable in complete and articulated condition. The long axis (proximal-distal) of the right fin measures 207 mm. The shorter proximal radials are 30 mm long and the row they are arranged in is partially overlapped by the metapterygium at the base of the pectoral fin. The longest radials widen and flatten distally. The distal radials extend laterally, and range from 177 mm at the longest axis to 52 mm in the flanks. The dermal denticles have an irregular polygonal outline with three small tubercles on the surface. These polyodontode structures measure between 0.5 and 0.8 mm across and form a honeycomb-like structure (Fig. 9). Towards the tip of the pectoral fins, the denticles decrease in size from about 0.7 to around 0.1 mm (Fig. 9).

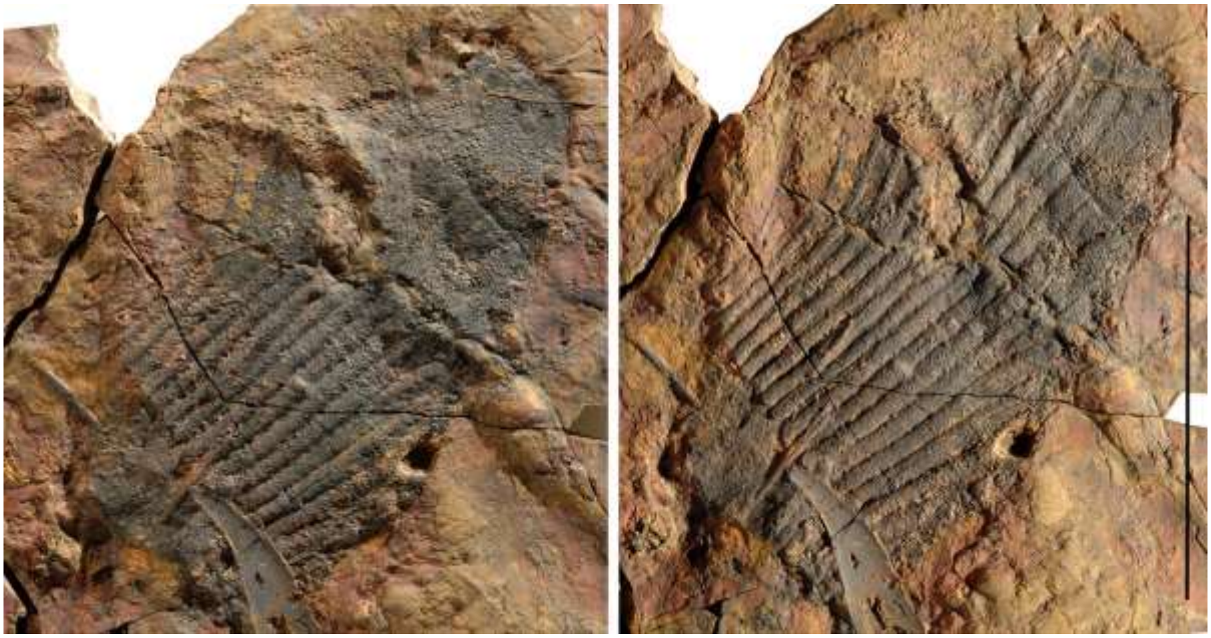

Supplementary Fig. 13 Right pectoral fin of *Maghriboselache mohamezanei* n. gen. et sp., PIMUZ A/I 5154. The left photo was taken with the light coming from the upper left to show the skin at the tip of the fin. The right photo was taken with light from the lower right to enhance the visibility of the radials. The scale bar is 100 mm.

Dark patches surrounding the cartilaginous skeletal elements may represent remains of soft tissue. Skin with dermal denticles *in situ* occurs at the fins and in the skull region. Several small orthocerids and bivalves (buchiolids) are scattered on and around the skeleton (Supplementary Fig. 11).

PIMUZ A/I 5155 (Supplementary Fig. 14 to 16)

Locality: 1.5 km southwest of Madene El Mrakib, southern Maïder, N30.719047258087453°, W-4.726626803690284°

Stratigraphic position: Thylacocephalan Layer, early Middle Famennian.

Finder: Christian Klug (Zürich)

Preparator: Christina Egli (Zell ZH)

Description: This specimen is 102 cm long. As in several other specimens, only the very base of the caudal fin is present. The animal is exposed in ventral view and its body length can be extrapolated to about 1.3 to 1.4 m. The cranial skeleton is very poorly preserved. Due to the orientation of the specimen on the bedding plane, the pectoral and pelvic fins are spread laterally on each side of the body, and the pectoral and pelvic girdles are preserved. The dorsal fins are not visible directly, but the posterior dorsal fin is subvertically embedded and can be examined on a fracture between two slabs. The posterior end of the specimen and the caudal fin are absent. The skull is heavily fractured and corroded, incomplete, and overall poorly preserved. Fragments of the Meckel's cartilage are preserved. Their position suggests the same pattern of preservation previously described for specimen PIMUZ A/I 5155. Parts of the chondrocranium are preserved in fragments between the two Meckel's cartilages. The ceratobranchial segments of the branchial arches are roughly symmetrically arranged posterior to the skull. Since this specimen is ventrally exposed, right and left branchial arches are discernible and articulated. They project in a posterolateral direction. The basibranchials can be seen between the anteromedial ends of the branchial arches.

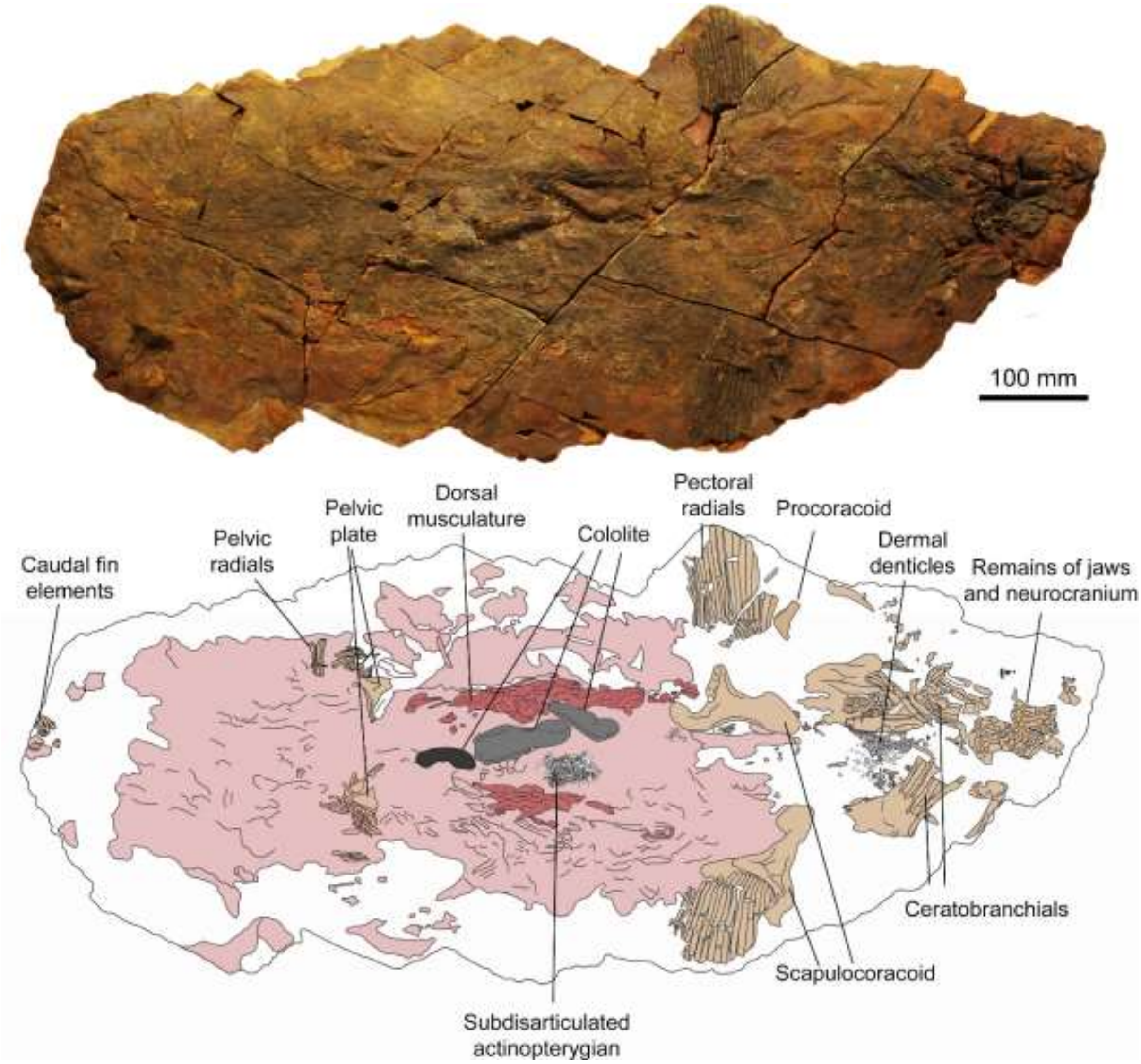

Supplementary Fig. 14 Nearly complete skeleton of *Maghriboselache mohamezanei* n. gen. et sp., PIMUZ A/I 5155, with extensive soft tissue preservation including stomach content with a small partially articulated actinopterygian.

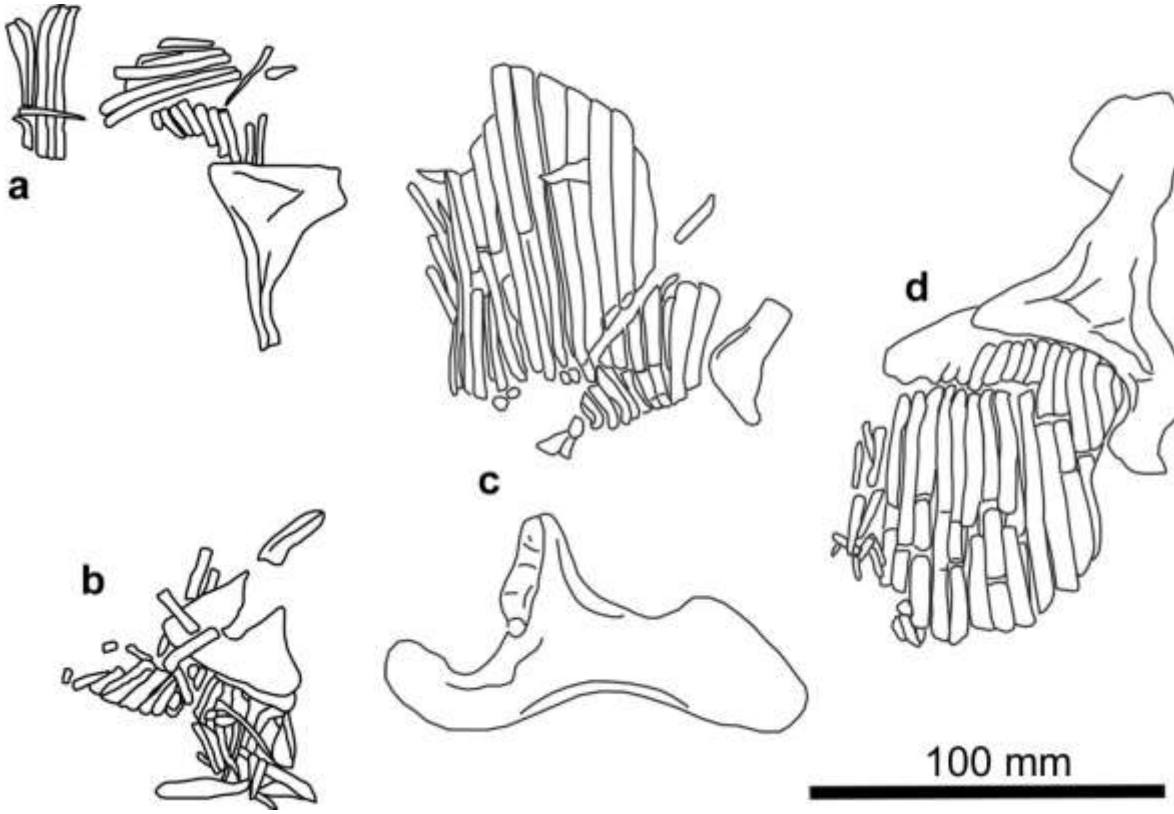

Supplementary Fig. 15 Paired fins of PIMUZ A/I 5155. A, subdisarticulated radials of right pelvic fin with the pelvic girdle preserved. B, subdisarticulated radials of left pelvic fin with the pelvic girdle preserved, shown in the scaled distance to the other pelvic fin. C, Right pectoral fin with the scapulocoracoid slightly displaced but complete. D, Left pectoral fin with radials still in articulation; note the articulation in the anterior radials at the edge of the scapulocoracoid and the distally widened radials.

The pectoral girdle is preserved (Supplementary Fig. 15). Its composing elements are in a relatively good condition, but more or less displaced from their original position. The right scapulocoracoid is separated from the pectoral fin. Its distal part is oriented posteriorly and fractured. Again, the proximal process is relatively narrow. However, in this specimen, the most proximal end expands slightly. The anterior and posterior side of this shaft are thus slightly concave. The distal part is relatively wide, and the distal side is deeply concave. Its total length is 14.6 mm. The right procoracoid is also displaced, and incomplete. The left scapulocoracoid is partially discernible, consisting mostly of the distal part in articulation with the fin's radials. The pectoral fins are present and articulated, but slightly incomplete. The right pectoral fin measures

92.2 mm and the left, 107.41 mm in length. The radials are articulated, extending laterally. There are 12 broad radial elements, which are about 5 mm wide and were maximally about 100 mm long, followed posteriorly by three distinctly more narrow radials, which are about 3 mm wide.

The right pelvic plate has a slim proximal shaft and a triangular distal part (Supplementary Fig. 15a, b). The anterior side is slightly concave and the posterior side is slightly convex. Some of the posterior proximal radials are still articulated. The remaining radial elements are dispersed in proximity. There are between 12 and 15 rays, although it is difficult to say this with certainty because of the disarticulation.

Due to the ventral exposure of this specimen, the posterior dorsal fin is oriented vertically into the underlying concretion. Just medial to the left pelvic fin, on the posteromedial side of the block, the radials can be seen. They are poorly preserved, but articulated. The fin is incomplete except for the most distal tips of the radials. Anterior to the fin, a fragment of the posterior dorsal fin spine is also preserved.

Mineralized soft tissues demarcate the outline of the body. Elongated black rolls of soft tissue extend along the sides of the body. Their finely striated structure suggests that these are muscle bundles replaced by hematite. Accordingly, myomeres can be identified as diagonally oriented segments. In the center of the specimen, between the pectoral and pelvic fins, a partially disarticulated actinopterygian including skull remains and the much of the body scales is preserved as stomach content, which documents prey preferences and that the prey was swallowed whole. The actinopterygian’s head and scales are discernible, but a detailed description will be provided in a separate paper. On the right side, anterior to the pelvic fins, a thick and elongate bulging structure likely represents a cololite.

There is no evidence of sensory organs, but patches of skin are preserved posterior to the skull around the branchial arches (Supplementary Fig. 16). The skin much resembles that of PIMUZ A/I 5155: the texture is rough by the presence of small rounded denticles. These denticles are, however, slightly larger than in the aforementioned specimen. Sometimes, under magnification, pointy projections are visible. Overall, they correspond well to the scales figured by Dean ((B. Dean 1909): fig. 1, 2).

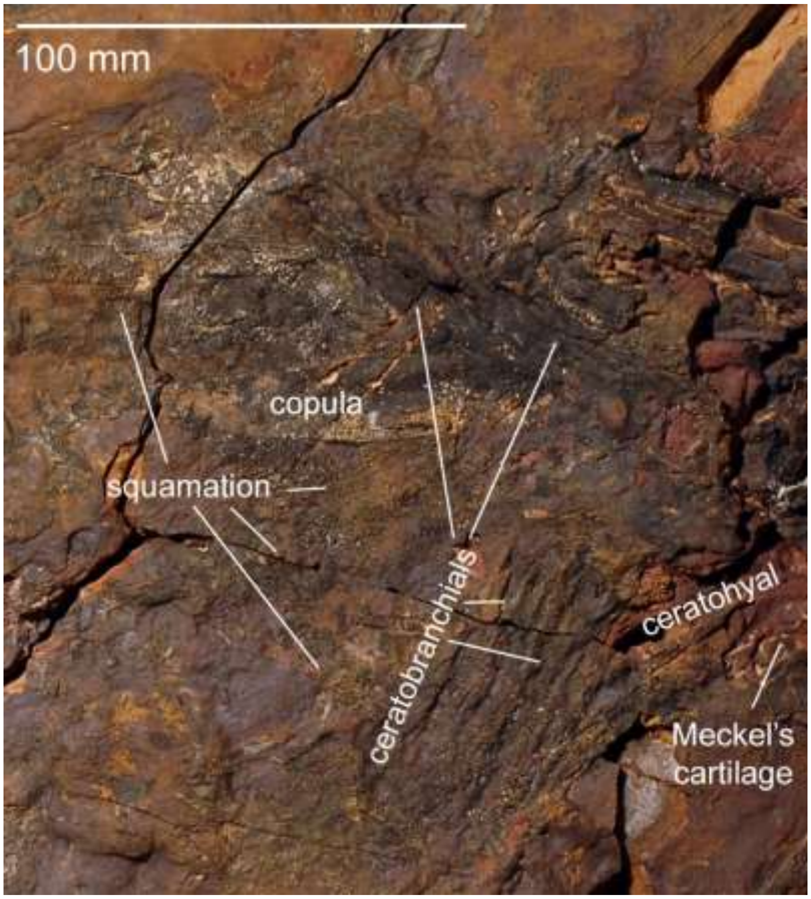

Supplementary Fig. 16 Ventral view of the gill region of PIMUZ A/I 5155, showing preserved integument and articulated splanchnocranium.

AA.BER.DS.01 (Supplementary Fig. 17 to 19)

Locality: Bid Er Ras, southwestern Maïder, N30°45.606', W4°55.637'

Stratigraphic position: Thylacocephalan Layer, early Middle Famennian.

Finder: Saïd Oukherbouch (Tafraoute)

Preparator: Claudine Miserèz (Neuchâtel)

Description: The distinctive features of "Spiny" AA.BER.DS.01 are the preservation of a dorsal fin spine and the caudal fin that preserves a good part of its dorsal lobe, which is lost during taphonomic processes in most other available specimens. The skeleton is preserved in lateral view. The somewhat scattered remains of this specimen extend over 110 cm. The cartilaginous skeleton is largely articulated with some radial elements of the fins dispersed in proximity.

The skull is preserved mostly as an imprint on the concretion. It measures approximately 121 mm in length. Some cartilaginous elements of the neurocranium are present near the midline, but their preservation does not permit to determine their original position. Meckel's cartilage is a rather slender element. It is about 100 mm long and 30 mm high close to the articulation; the anterior end is not well visible. The palatoquadrate has a similar length as the lower jaw and the classic cleaver-shape. The posterior end is well exposed, although it is merely an external mold. There, it is up to 40 mm high. The palatine ramus is rather flat and slightly curved upwards anteriorly.

Twenty-four neural arches can be counted, with four or five more preserved as external moulds. There are 21 articulated neural arches and they extend over 144 mm of the postcranium, directly posterior to the pectoral girdle. Measuring the estimated total length of the vertebral column, we extrapolate the presence of about 94 neural arches.

The right scapulocoracoid measures 83 mm. Similar to the other specimens described herein, it is a robust cartilage with a wide distal end that is deeply concave for insertion of the pectoral fin. The medial stem is narrower with a blunt end. The pectoral fin, best preserved on the left side, measures 121 mm from its base to its distal end. It is quite disarticulated and it is not clear, which element belongs to which of the pectoral fins.

The left pelvic plate measures 32 mm. It is a fan-shaped element with a wide distal portion with a convex distal edge and gradually narrows medially into a blunt end. The pelvic fins are disarticulated, with radials scattered around the pelvic area. The radials are up to about 30 mm long and much slenderer than those of the pectoral fins.

The caudal fin is asymmetrical. The dorsal lobe (142 mm) is leaner and tapers distally. The ventral lobe (86 mm long) is relatively wider. The dorsal lobe preserves mainly the neural spines (up to 23 mm long), which are slim rods with straight ends. Supraneural radials (51.8 mm) are also preserved at the dorsal base of the dorsal lob, as well as neural spines (18 mm). The eleven hypochordal radials of the ventral lobe are incomplete and up to 93 mm long. They are more robust elements than seen in the dorsal lobe. They are slender and long, and together form a wide and laterally compressed lobe adequate for swimming (Supplementary Fig. 17).

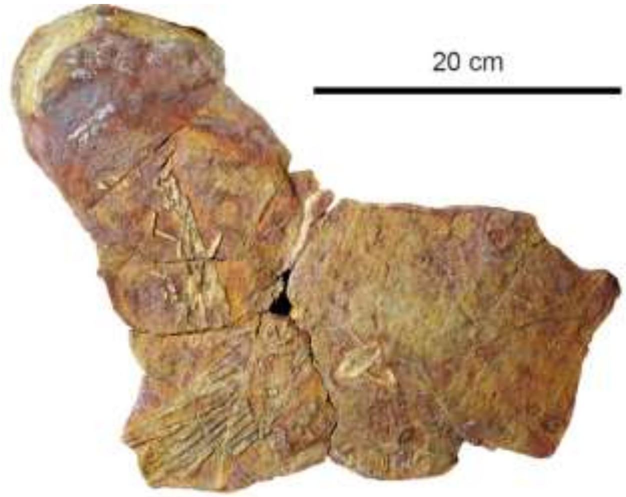

Supplementary Fig. 17 Caudal fin of *Maghriboselache* AA.BER.DS.01.

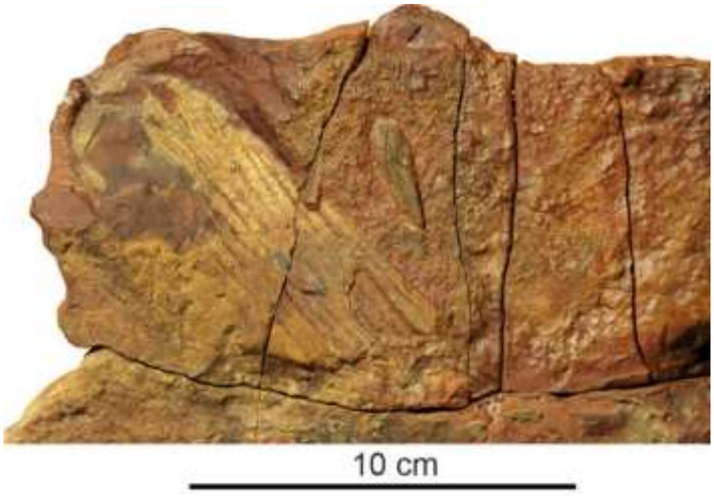

Supplementary Fig. 18 Posterior dorsal fin of *Maghriboselache* AA.BER.DS.01. The rather short and not ornamented fin pine is slightly displaced.

The posterior dorsal fin is very well preserved. It is positioned at the level of the pelvic region, with the radials still articulated and slightly tilted posteriorly, possibly for hydrodynamics. It measures 104 mm in length per 42 mm at the base. It is elongated with a lobate outline. The short proximal radials are 13 mm long, and the distal radials measure 92 mm at the center of the fin. The dorsal fin spine is present anterior to the dorsal fin. It is a robust element, which is 31

mm long. It is distinctly thicker than the fin radials with a pointed proximal end and a curved distal end (Supplementary Fig. 18).

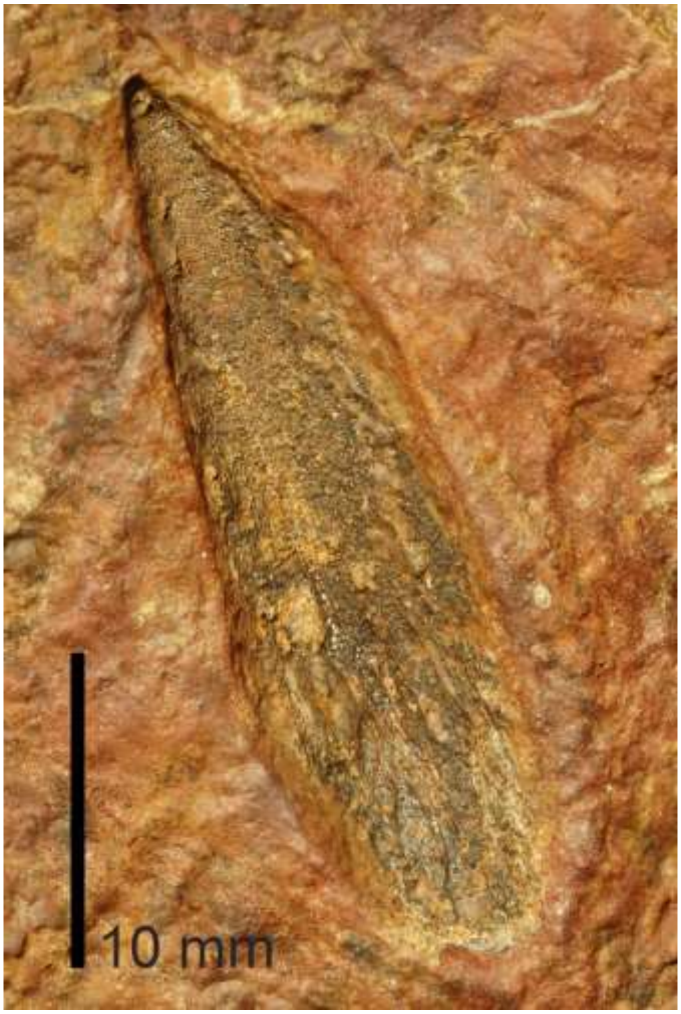

Supplementary Fig. 19 Dorsal fin spine of the posterior dorsal fin of *Maghriboselache mohamezanei* n. gen. et sp., AA.BER.DS.01; note the absence of ornamentation and the short and stout form.

PIMUZ A/I 5156 (Supplementary Fig. 20 to 26)

Locality: Madene El Mrakib, southern Maïder, N30.750475979708618°, W-4.713329273883624°

Stratigraphic position: Thylacocephalan Layer, early Middle Famennian.

Finder: Saïd Oukherbouch (Tafraoute)

Preparator: Christina Egli (Zell ZH)

Description: The specimen measures 79 cm in length, although its posteriormost part, including the caudal fin, is missing. It was probably about 1.1 m long and thus one of the smaller specimens (Fig. 2). It is in an obliquely ventral (anterior part) to obliquely lateral position (posterior part). The remaining skeleton shows exceptional preservation conditions including soft tissues. The skull is, however, quite weathered. Even though it is incomplete, it preserves most of the splanchnocranium. Both pectoral fins are present, one of them complete and still in articulation with the pectoral girdle (on the right side). Only the posterior dorsal fin is preserved. Elements of the shoulder and pelvic girdles are visible and more or less articulated, which allows for a morphological inspection.

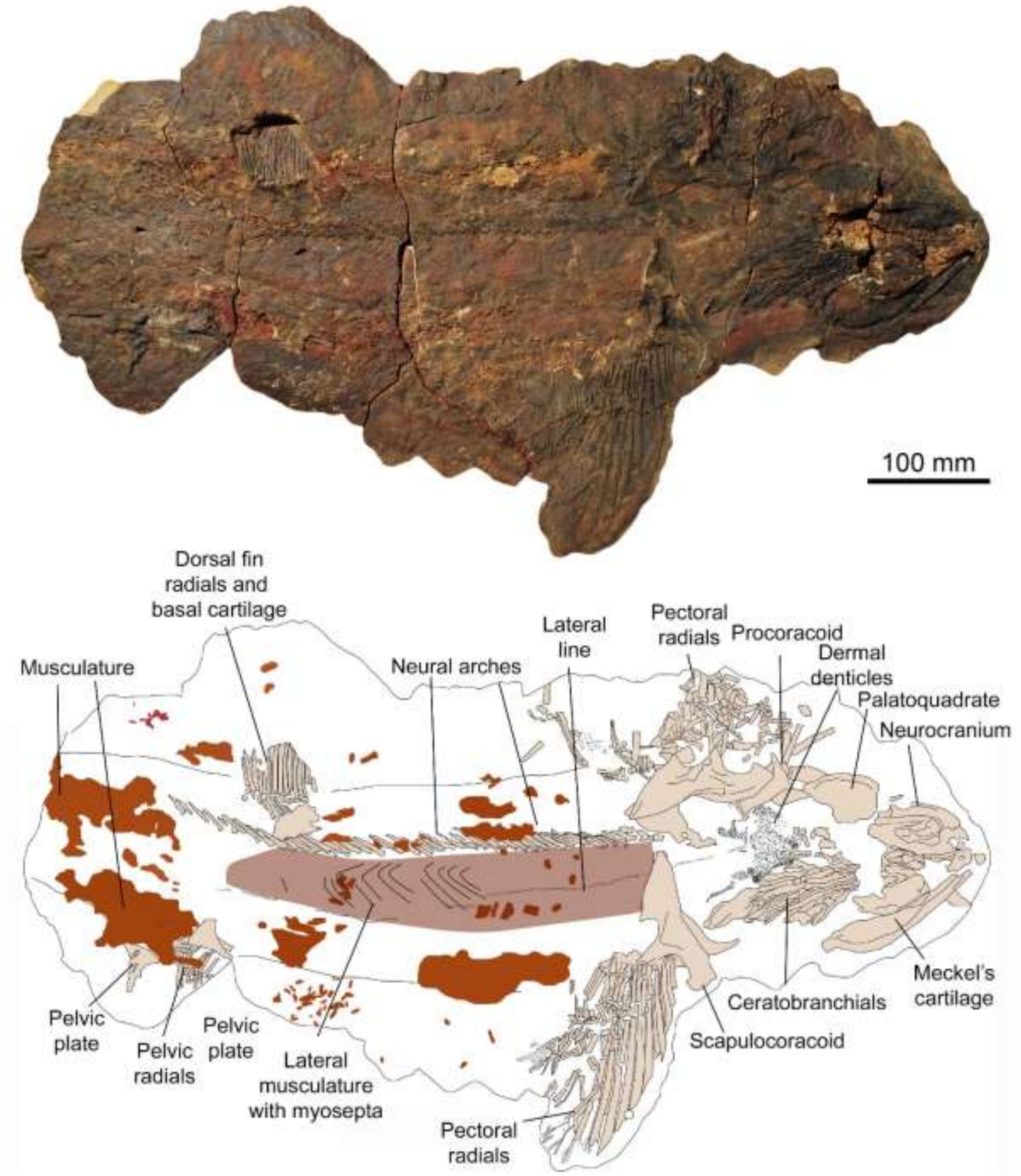

Supplementary Fig. 20 PIMUZ A/I 5156, nearly complete skeleton lacking only the caudal fin. Note the mineralized musculature as well as the lateral line and the obliquely preserved posterior dorsal fin.

The skull is exposed in ventral view. Both Meckel's cartilages are present and measure 123 to 138 mm (uncertainty due to compaction deformation). The Meckel's cartilage is robust, laterally compressed, and ventro-dorsally expanded in its posteriormost part. In this specimen, it appears to have rotated during decay and to be compacted below the palatoquadrate so that the dorsal, slightly concave side is facing medially. Its ventral, convex side is projecting laterally. Between both Meckel's cartilages, a portion of the palatoquadrate is displayed on the right. Slightly posteriorly, basihyals or basibranchials (?), and branchial arches (probably the ceratobranchials) are visible on the right. The braincase is insufficiently preserved to allow the description of its morphology.

Few teeth are visible. The dentition is not very well preserved, of cladodont type and very close in morphology to *Cladoselache*. The teeth have a wide and short base, however without expanding laterally much beyond the base of the crown. The median cusp is triangular, with a relatively wide base, and a circular cross section. It is flanked by one lateral cusp of much smaller size on each side; sometimes, two lateral cusps are visible on both sides.

The neural arches are present and extend along the length of the animal. They are slender, elongated elements, bifurcated. They are five times as high as they are long. The neural canal is barely visible due to the deformation. 40 neural arches are preserved.

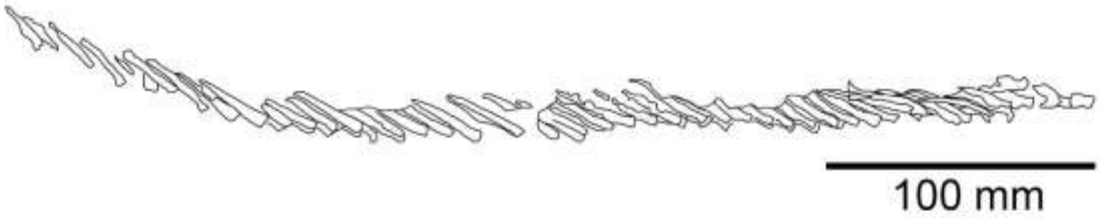

Supplementary Fig. 21 Axial skeleton of *Maghriboselache mohamezanei* n. gen. et sp., PIMUZ A/I 5156, showing most neural arches.

The scapulocoracoid is well preserved on the right side of this specimen. It is a large element, 117 mm long. The proximal portion has a narrow base without anterior and posterior projections. The scapulocoracoid expands slowly distally, assuming a fan-like shape. Both anterior and posterior sides are slightly concave. The distalmost side is deeply concave below the articular crest, where the radials articulate. The procoracoid is 44 mm long. It is a sickle-shaped element just anterior to the scapulocoracoid, and much resembles that of *Akmonistion* (M. I. Coates and

Sequeira 2001). The fin's mesopterygium, 41 mm long, lies on top of the scapulocoracoid. Like the scapulocoracoid, the mesopterygium is proximally narrow, but in this case resembling a long rod. The most distal part expands abruptly in a triangular process. Distal to the mesopterygium, the basal radials, the radials, and ceratotrichia extend successively. The left pectoral fin (Supplementary Fig. 22, 23) displays ten distinct radials, which increase in length from 25 mm at the anterior edge to 155 mm at the greatest length of the fin. The posterior elements are disarticulated. Especially at the posterior edge of the fin, numerous fine ceratotrichia are preserved (Supplementary Fig. 23).

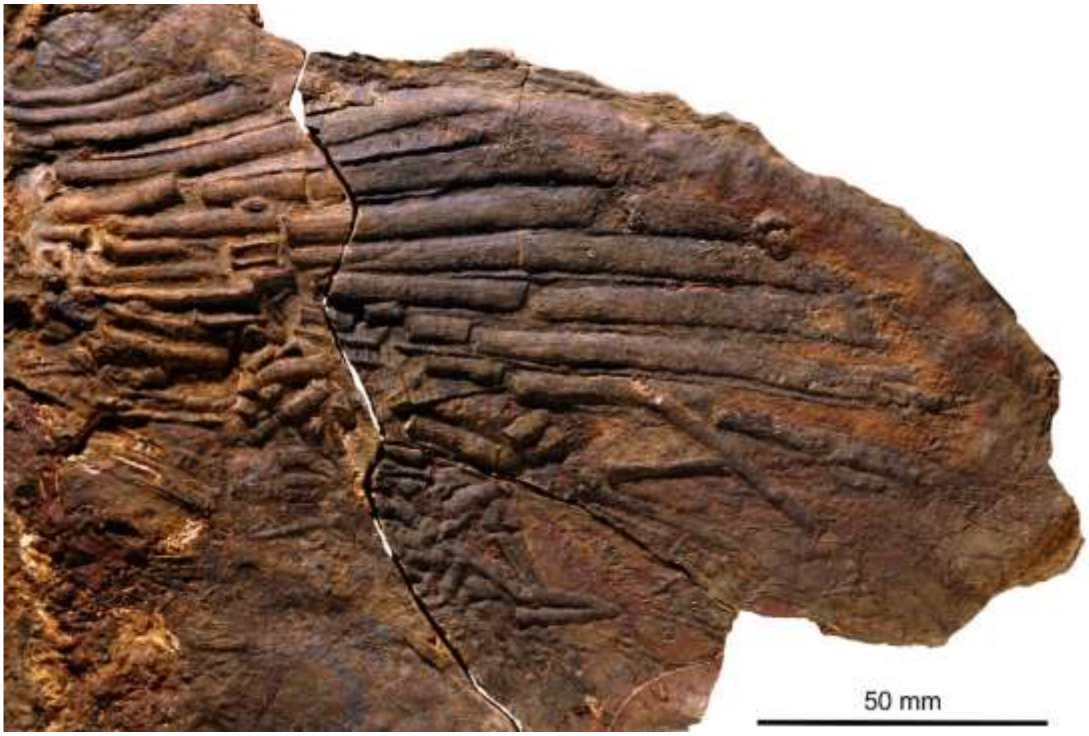

Supplementary Fig. 22 Pectoral fin of *Maghriboselache mohamezanei* n. gen. et sp., PIMUZ A/I 5156. Note that the radials have cracked, hence the furrow at the posterior edge. This is a widespread taphonomic artefact.

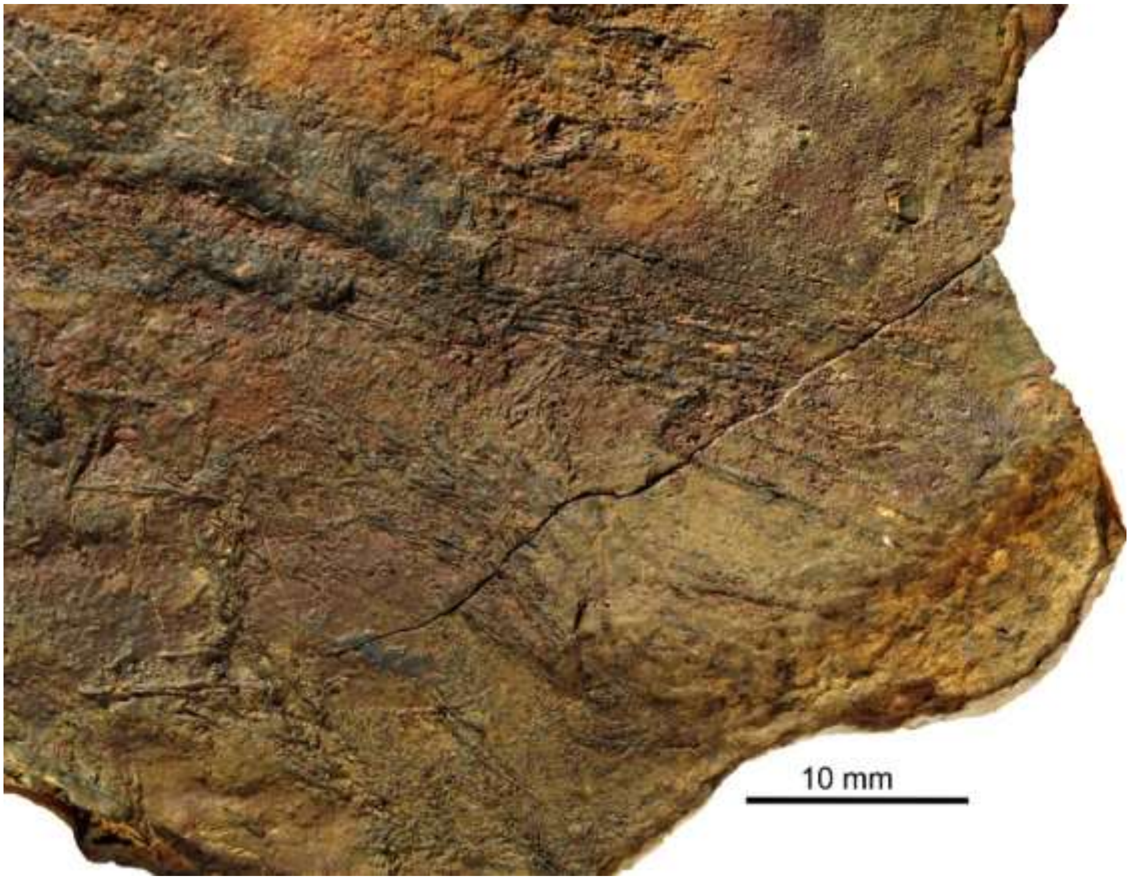

Supplementary Fig. 23 Detail of pectoral fin of *Maghriboselache mohamezanei* n. gen. et sp., PIMUZ A/I 5156. At the posterior end of the pectoral fin, fine ceratotrichia are preserved, more or less in articulation.

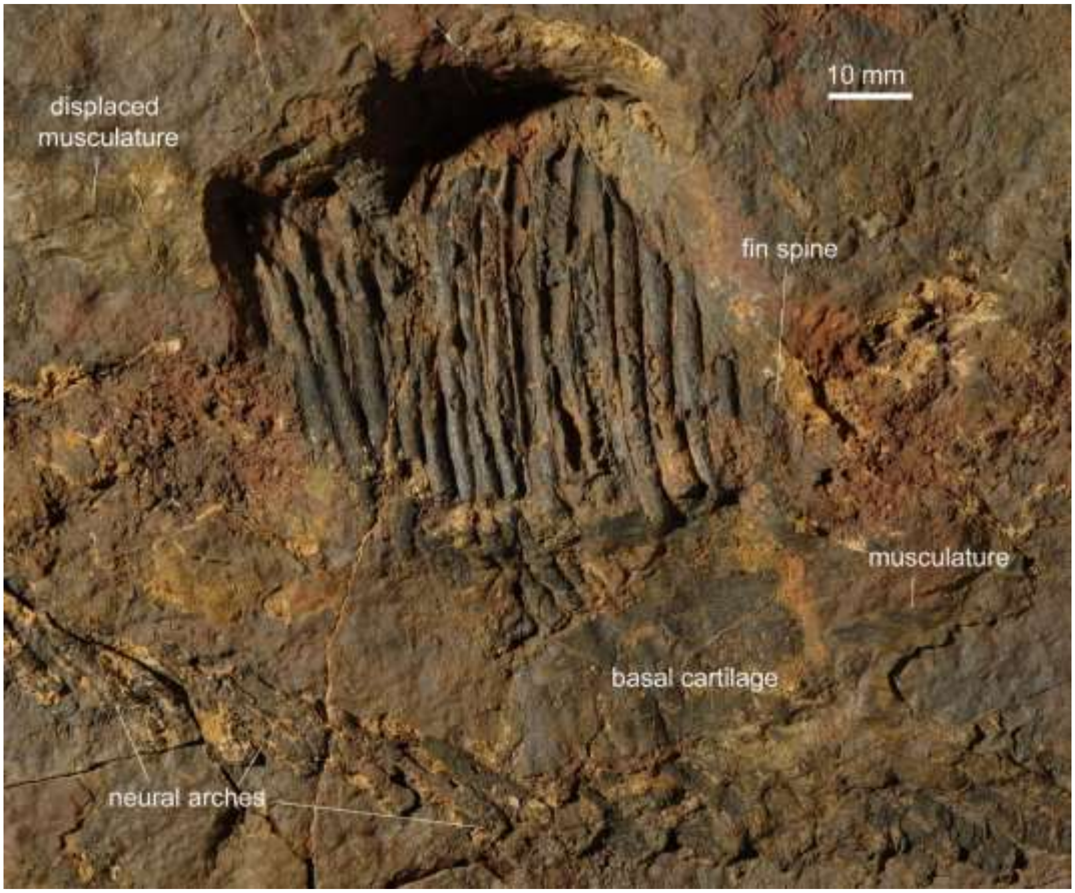

Supplementary Fig. 24 Posterior dorsal fin of *Maghriboselache mohamezanei* n. gen. et sp., PIMUZ A/I 5156. Note the nearly perfect articulation and the remains of mineralized muscle fibers.

An anterior dorsal fin is neither visible, nor is there evidence of an anterior dorsal fin spine. Since the skeleton is very well preserved otherwise, we assume that there was no anterior dorsal fin. The posterior dorsal fin, however, is quite well preserved and articulated (Supplementary Fig. 24). Due to the oblique position of the specimen, the posterior dorsal fin has sunken into the sediment on the left side of our specimen. The basal plate is present at the base of the fin. It measures 35 x 23 mm, but it might be incomplete. It is followed by the basal radials and the radials. The radials are still partially covered by sediment, because it was not possible to differentiate between sediment and fossil during preparation. The radials are between 16 mm (anteriorly) and over 60 mm in the middle of the fin. The basal radials reach about 20 mm in length and become longer posteriorly. Since the specimen is only preserved until shortly posterior to the pelvic fins, the caudal fin is not preserved.

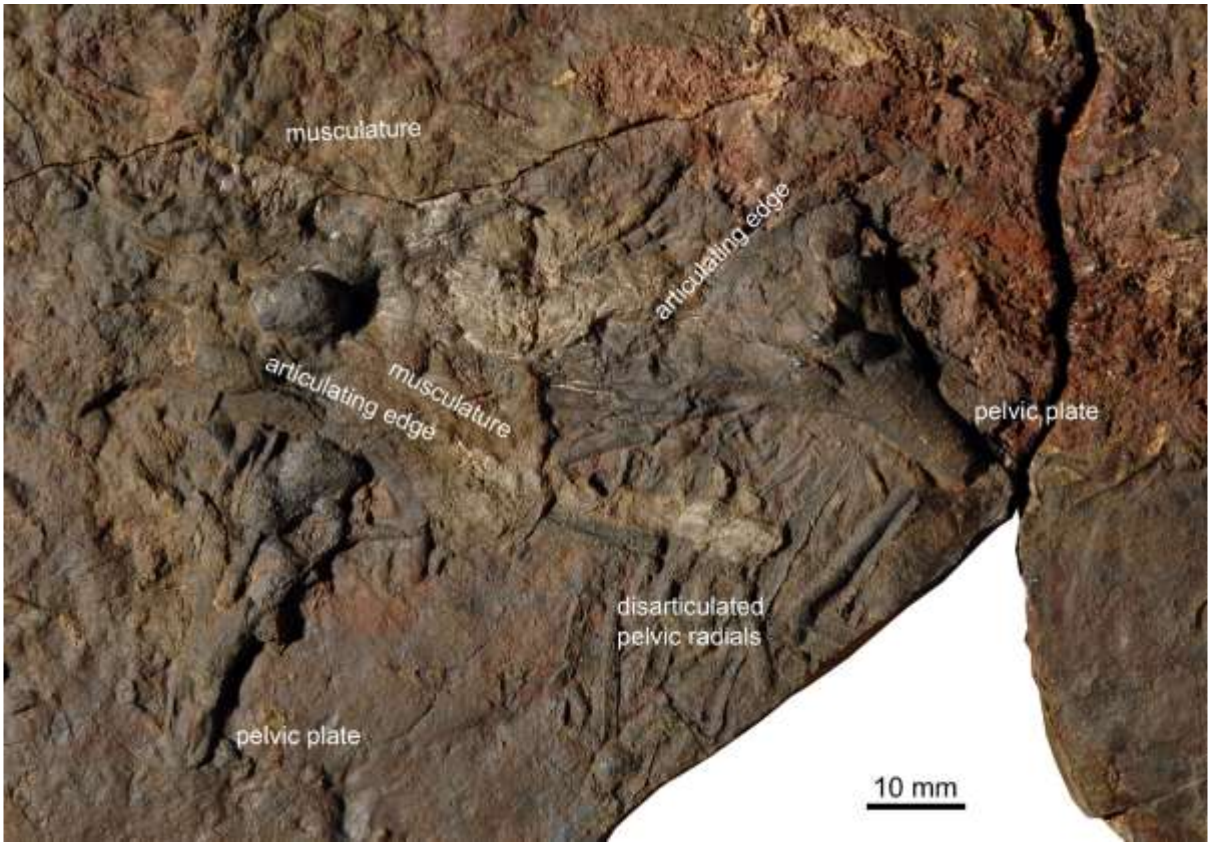

Supplementary Fig. 25 Pelvic fin of *Maghriboselache mohamezanei* n. gen. et sp., PIMUZ A/I 5156. Note the nearly perfect articulation and the remains of mineralized muscle fibers.

The pelvic region is moderately well-preserved, displaying both pelvic plates and numerous radials, surrounded by mineralized muscle bundles (Supplementary Fig. 25). The pelvic plates are about 30 mm high dorsoventrally and 27 mm long. Both the posterior and anterior edges are concave, making the plate nearly symmetrical. The articulating edge is convex mesially and ventrally. No separate articulations for the radials are discernible. The radials are 12 to 30 mm long and rod-shaped.

Skin patches are preserved close to the branchial arches. The integument has a rough appearance. Under magnification, the small dermal denticles can be discerned. They measure roughly one millimeter, but they are better preserved in other specimens. The denticle crown present small, longitudinal ridges.

Between the pectoral girdle and the posterior dorsal fin, the lateral line is preserved with its modified denticles (Fig. 12). These denticles are 1 mm long and 1.5 mm wide. Posteriorly, they display 0.3 mm long rounded lateral processes. They surround a channel, which is about 0.5 mm wide and here filled with a greyish mineral strand.

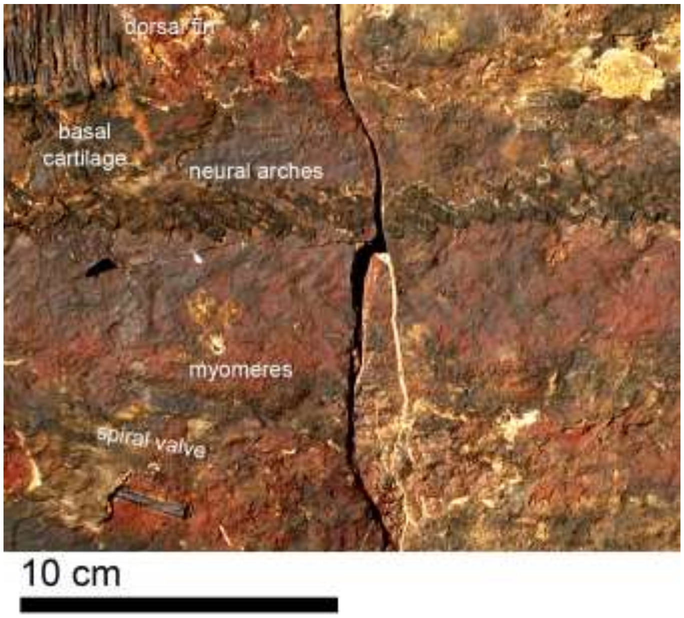

Supplementary Fig. 26 Detail of thorax of *Maghriboselache mohamezanei* n. gen. et sp., PIMUZ A/I 5156, showing myomeres and the spiral valve.

Its exceptional preservation revealed some soft tissue such as musculature remains. Hematitized muscle tissue is seen surrounding the sides of the specimen (Supplementary Fig. 26). In many places, muscle remains can be seen displaying bundles of fine fibers about 0.1 mm thick and up to 5 mm long. Ventral to the neural spines, remains of the V-shaped myomeres and collagenous myosepta are preserved in relief (Supplementary Fig. 26). Their remains form a chevron pattern on the ventrolateral side, close to the pelvic fins. The ventral part of these structures is approximately 4 mm wide and 18 long. It is inclined anteriorly, while the part between this and the neural arches is inclined posteriorly and is around 15 mm long. On the left side, close to the pelvic girdle, dark round elements might represent the spiral valve potentially with gut content or hematite concretions.

PIMUZ A/I 5157 (Supplementary Fig. 27)

Locality: Bid Er Ras, southwestern Maïder, N30.694068684968°, W-4.915991949048543°

Stratigraphic position: Thylacocephalan Layer, early Middle Famennian.

Finder: Saïd Oukherbouch (Tafraoute)

Preparator: Merle Greif (Winterthur)

Description: This specimen preserves much of its dorsal musculature but lacks most of the head region. The nodule is about 890 mm long. Anteriorly, an elongate imprint might be the external mold of the palatoquadrate. Behind it, the poorly preserved anterior dorsal fin spine is visible. It is about 72 mm long. Posterior of the fin spine, there is an oval imprint (light brown in Fig. 24). Perhaps, this is the fin cartilage of the anterior dorsal fin. Remains of the permineralized hematitic musculature extends over 430 mm. The shoulder girdle is not preserved, but the radials of one pectoral fin are present. About 11 radials were counted, which are up to 75 mm long. The posterior dorsal fin is still in situ. It displays the slender dorsal fin spine and 11 radials, which are up to 40 mm long. The basal cartilage is not visible. Posteroventral of the posterior dorsal fin, two elongate furrows might represent imprints of the spiral valve/ posterior digestive tract. The pelvic fins and girdle elements are not exposed. Remarkably, the caudal fin is present: Seven hypochordial rays are preserved, which are up to 55 mm long. Haemal and neural spines are also visible, but in a disarticulated state. Four supraneural radials are still articulated and display a sinusoidal shape. Both sub-millimeter muscle fibers and thicker bundles, which are about 5 mm thick, are discernible.

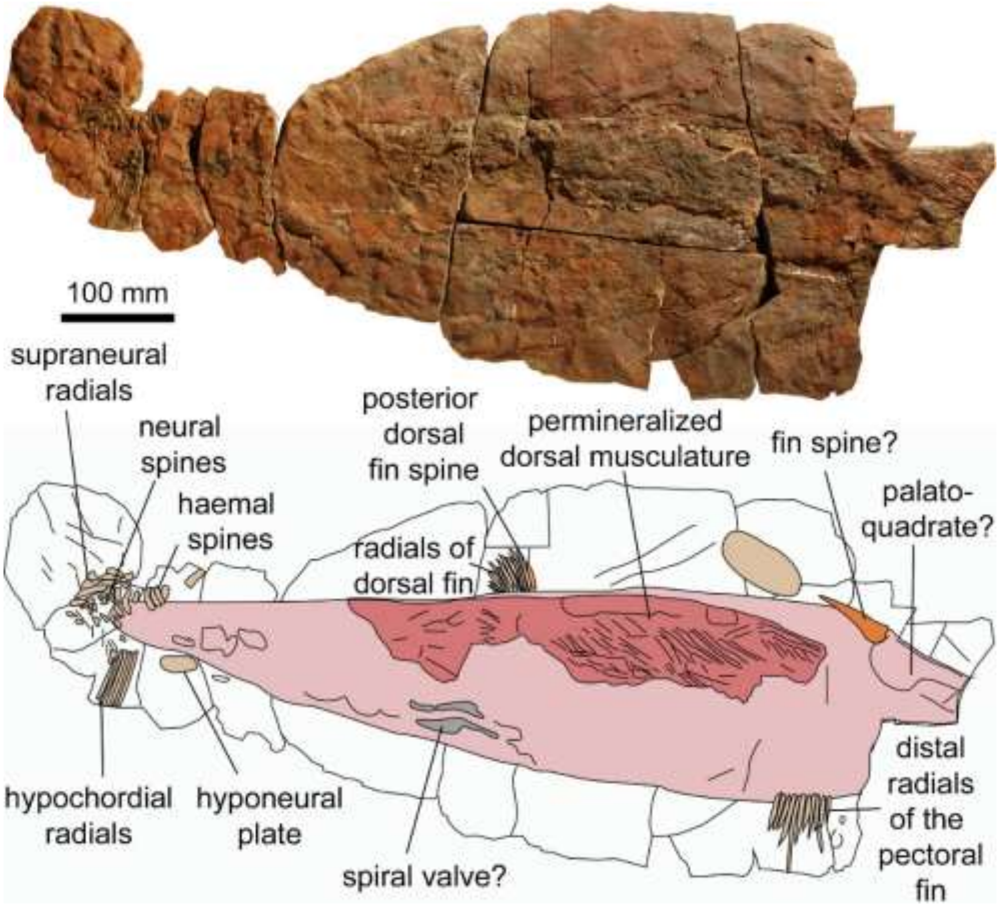

Supplementary Fig. 27 Overview over the incomplete skeleton of *Maghriboselache mohamezanei* n. gen. et sp., PIMUZ A/I 5157. The anterior part is well-preserved, while in the posterior portion, mainly the body outline can be seen. The left pectoral fin and the portion between the gills and pectoral fins preserves a lot of integument.

PIMUZ A/I 5158 (Supplementary Fig. 28)

Locality: W of Jebel Aoufilal, W of Taouz, southern Tafilalt, N30°56’25.2”, W4°01’14.9”

Stratigraphic position: Early Middle Famennian.

Finder: Mohamed Mezane (ElKhraouia)

Preparator: Merle Greif (Winterthur)

Description: This is the only articulated specimen included in this study from the southern Tafilalt. Additionally, it is remarkable for its phosphatized preservation of musculature and its comparatively small size. In its incomplete state, it is only 555 mm long. Its body length was likely between 800 and 900 mm. It thus is the smallest, nearly complete skeleton, which is included here.

The flattened neurocranium is about 83 mm long. Since it is crushed and other specimens are perfectly 3D-preserved, we do not describe morphological details here. Both Meckel’s cartilages are present. Since the skeleton came to rest on its back as most of the available skeletons, the lower jaws dropped down outward on both sides, thus giving the skull a rather broad, nearly frog-like impression. Remarkably, a similar preservation is known from *Cladoselache kepleri* from the Cleveland Shale, as depicted by Dean ((B. Dean 1909): pl. 27). The left ramus of the lower jaw is more complete, about 128 mm long and up to 25 mm high. It is rather straight and curved only shortly before the symphysis and close to the articulation. The ventral ridge is distinct, up to 5 mm high, and strongly developed along most of the lower edge of the ramus. Posteriorly, the Meckel’s cartilage is slightly bent outward and upward towards the articulation. The retroarticular flange is rounded triangular and appears to be slightly shorter than in *Gogoselachus* (Long et al. 2015), while overall, the lower jaw is much more slender and longer.

Both palatoquadrates are deformed and yet quite well-preserved. The right palatoquadrate (on top in Supplementary Fig. 28) is seen from the outside and shows the adductor fossa with the strongly vaulted posterodorsal edge. Its otic part is 55 mm long and 35 mm high. The quadrate condyle is composed of a boss facing outward, which is 3.5 mm long, 5 mm high and the cartilage is about 3.5 mm thick. Anteriorly, there is the articular facet that held the articular process of the lower jaw. It is about 6 mm long and nearly 3 mm deep. Like in *Gladbachus* ((Coates et al. 2018): fig. 2), the dorsal edge of the otic process is slightly concave behind the orbit, forming a small postorbital node at the top of its edge. The palatine process is about 37 mm

long and dorsoventrally flattened with a thickness of around 2 mm. Its width was probably around 10 mm, as can be seen in the left palatoquadrate.

Many elements of the gill arches are preserved in situ but they are strongly flattened, thus hampering their identification. The left ceratohyal is lying on top of the left Meckel's cartilage. It is rather straight, only towards its posterior end, it curves upwards. Its posterior articulation is fairly well preserved three-dimensionally. The entire cartilage is 95 mm long and up to 11 mm high, although it might have been lower before compaction. Its direct association with Meckel's cartilage suggests that these elements were in close contact also during life. Remains of the right hyoid are partially exposed under the fragmentary otic part of the right palatoquadrate. As far as it is visible, it measures 45 mm in length and between 8 and 11 mm in width. The ceratobranchials are slender elements, which are up to 85 mm long and about 5 mm wide in its deformed state. The pharyngobranchials and epibranchials are likely present but probably largely covered by the ceratobranchials. The copula is well-preserved and complete. It is 82 mm long and up to 16 mm wide. The anterior edge is triangular with a rounded tip. Both sides show a marginal elevation, which is roughly 1 mm wide, but this might be an artifact from compaction.

The shoulder girdle is quite well preserved. Both scapulocoracoids are present. The left scapulocoracoid and metapterygium are better preserved, and hence, the description is based on them. The scapulocoracoid measures 102 mm in dorsoventral direction and was about 55 to 60 mm long (slightly fragmented at both ends). Like in *Akmonistion* ((Coates and Sequeira 2001): Fig. 10), the coracoid is strongly turned downwards anteriorly, with a well-rounded posterior edge. It is 28 mm high and about 15 mm high. The diazonal foramen measures about 1 mm across and lies 100 above the articular crest. From the coracoid to the dorsal tip, the scapular portion measures about 80 mm. The posterior edge is rather straight while the anterior edge is gently curved, ending in a blunt dorsal tip. The left procoracoid is somewhat displaced towards the ceratobranchials and fragmentary. It measures 33 mm in maximum length and is up to 12 mm wide. Its posterior edge is slightly concave, while the ventral edge displays a slight convexity. The metapterygium is a subtriangular to pentagonal element, about 20 mm long and 21 mm wide. The gently concave articulation facet is still in articulation with the metapterygial condyle, which is about 7.5 mm wide.

Both pectoral fins are articulated but also not complete. The rod-shaped proximal radials are 11 to 15 mm long and about 3 to 4 mm thick, slightly widened near the articulation. Both

pectoral fins show eleven rays, which articulate with the scapulocoracoid. Telling from the ridges visible at the articular edge of the metapterygium, there were an additional four or five rays, which are not articulated anymore. Also similar to *Akmonistion* ((Coates and Sequeira 2001): fig. 10), there is a posterior axial extension preserved posterior to the metapterygium. This extension is composed of four to five cartilages, which measure about 5 to 7 mm in length. The entire structure extends 28 mm posteriorly from the metapterygium and ends in a bluntly triangular (primarily conical) element.

The pelvic fins are also preserved, although in a subdisarticulated state. Eight radials of the right pelvic fin are still associated and arranged in parallel. They are between 8 and 35 mm long and measure between 2 and 3 mm in diameter. The pelvic girdle is present, but the right fin lost contact of the pelvic plate. The pelvic plates are thin and triangular, with the dorsal portion being elongated. Both the anterior and the posterior edges are slightly concave, while the ventral edge is gently convex. The plate is 33 mm high and 25 mm long.

The anterior dorsal fin spine is a laterally narrow, posteriorly curved subtriangular element. Its surface is weathered, its black hematitic internal mold partially visible, and the tip is missing. Overall, the anterior edge was about 60 mm long and is gently concave. The posterior edge measures 36 mm in length and no denticles are visible, but this is likely due to the weathered state of the tip. The angle between the base and the posterior edge is about 45°. The fin cartilage is partially covered by phosphatized musculature. The visible part suggests that it measured roughly 40 times 20 mm, but its shape is obscured by phosphatized muscle. Although the posterior dorsal fin is reasonably articulated, it is separated from the slender fin spine. The fin spine is 34 mm long and quite weathered. Remains of 13 radials are visible, mostly as external molds. They are up to 40 mm long and 3 mm wide. The basal cartilage is not discernible.

The neural arches are partially well-preserved, but in some places, they are entirely eroded away and, in some places, only imprints remain. In total, traces of 21 arches are visible, but there were at least twice as many. The better-preserved ones are up to 30 mm long, pointed dorsally with the tip bending backwards. On the sides, they display a furrow, but it is unclear whether this is due to compaction and collapse of the cartilage. They are up to 5 mm wide.

Remarkably, this specimen preserves a lot of phosphatized muscle showing sub-millimeter details. Posterior of the pelvic girdle, the chevron-shaped muscle-bundles left imprints in the sediment.

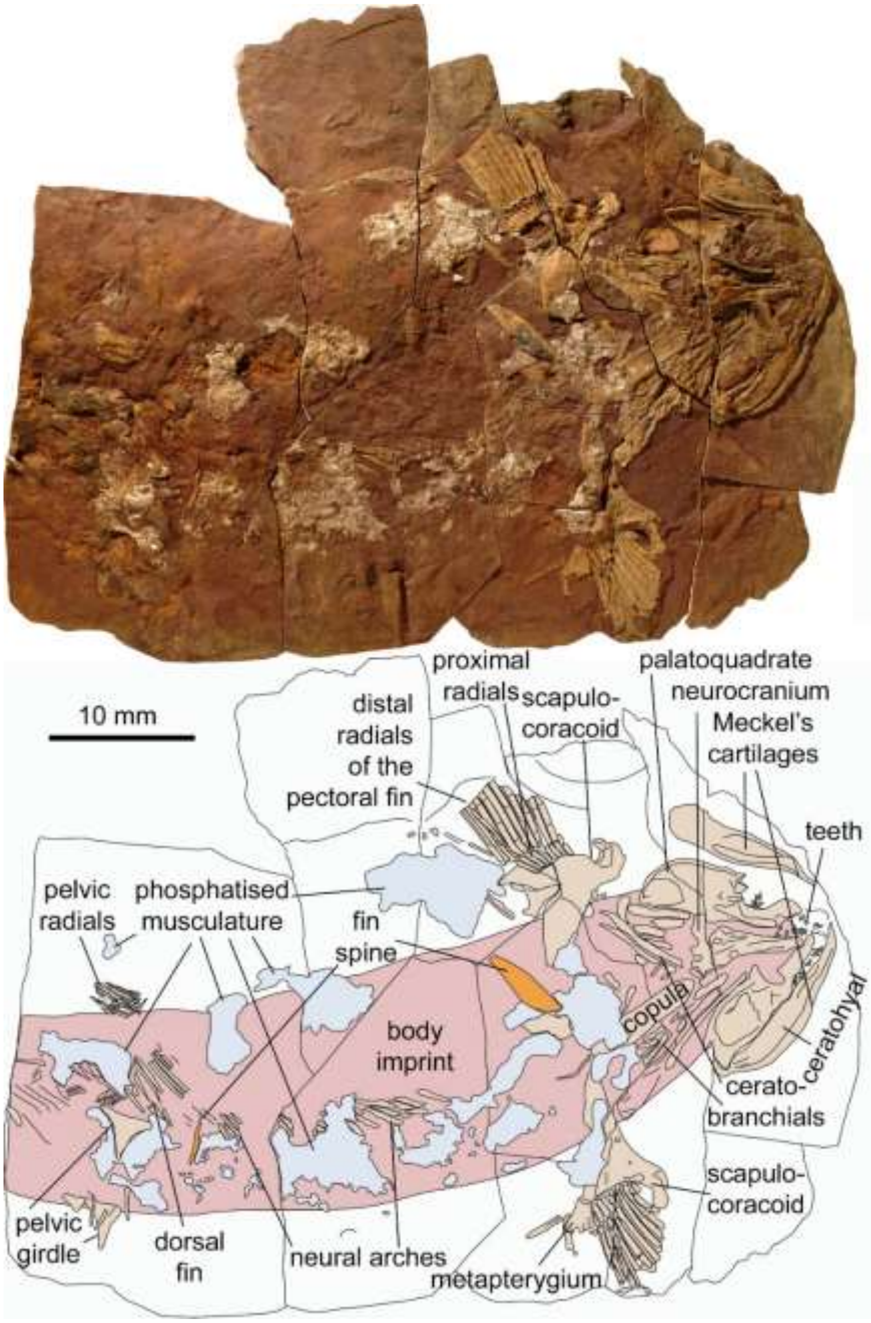

Supplementary Fig. 28 Overview over the incomplete skeleton of *Maghriboselache mohamezanei* n. gen. et sp., PIMUZ A/I 5158. The anterior part is quite well-preserved, while the caudal fin is missing. The phosphatic preservation of the musculature, the well-articulated pectoral fins (except the distal parts), the anterior dorsal fin spine and the complete, but flattened skull are remarkable.

Descriptions of isolated skulls

PIMUZ A/I 5159 (Supplementary Fig. 29 to 34)

Locality: Madene El Mrakib, southern Maïder.

Stratigraphic position: Thylacocephalan Layer, early Middle Famennian.

Finder: Saïd Oukherbouch (Tafraoute)

Preparator: Merle Greif

Description: This ranges among the best-preserved heads of a Paleozoic chondrichthyan, because the jaws and neurocranium are fully three-dimensionally preserved and articulated (Supplementary Figs. 29-33). The specimen is incomplete and preserved in a nodule rich in iron minerals. It is 155 mm long, 100 mm wide, and 109 mm high. The CT-scan revealed the presence of all main components of the jaws and the complete neurocranium, parts of the branchial skeleton and the anterior dorsal fin spine, associated with possible orthocerids. The description is largely based on the segmented data (Figs. 3, 5, Supplementary Figs 29 to 33).

The neurocranium is remarkable because of its undeformed state and the complete nasal region. The nasal capsules are nearly as broad as the postorbital process (Supplementary Fig. 31, 32). The nasal region is broadly rounded with distinct lateral lobes, delimited mesially by the narial openings, which lie on both sides of the very broad and bluntly trapezoidal ethmoid rostrum. The ethmoid region is fully exposed dorsally at the surface of the nodule and is about 70 mm wide. The distance between the narial opening measures 38 mm, which corresponds to the width of the trapezoidal rostrum. The surface of the fossil displays the characteristic honeycomb pattern of the tessellated cartilage. The single tesserae measure about 0.25 mm across.

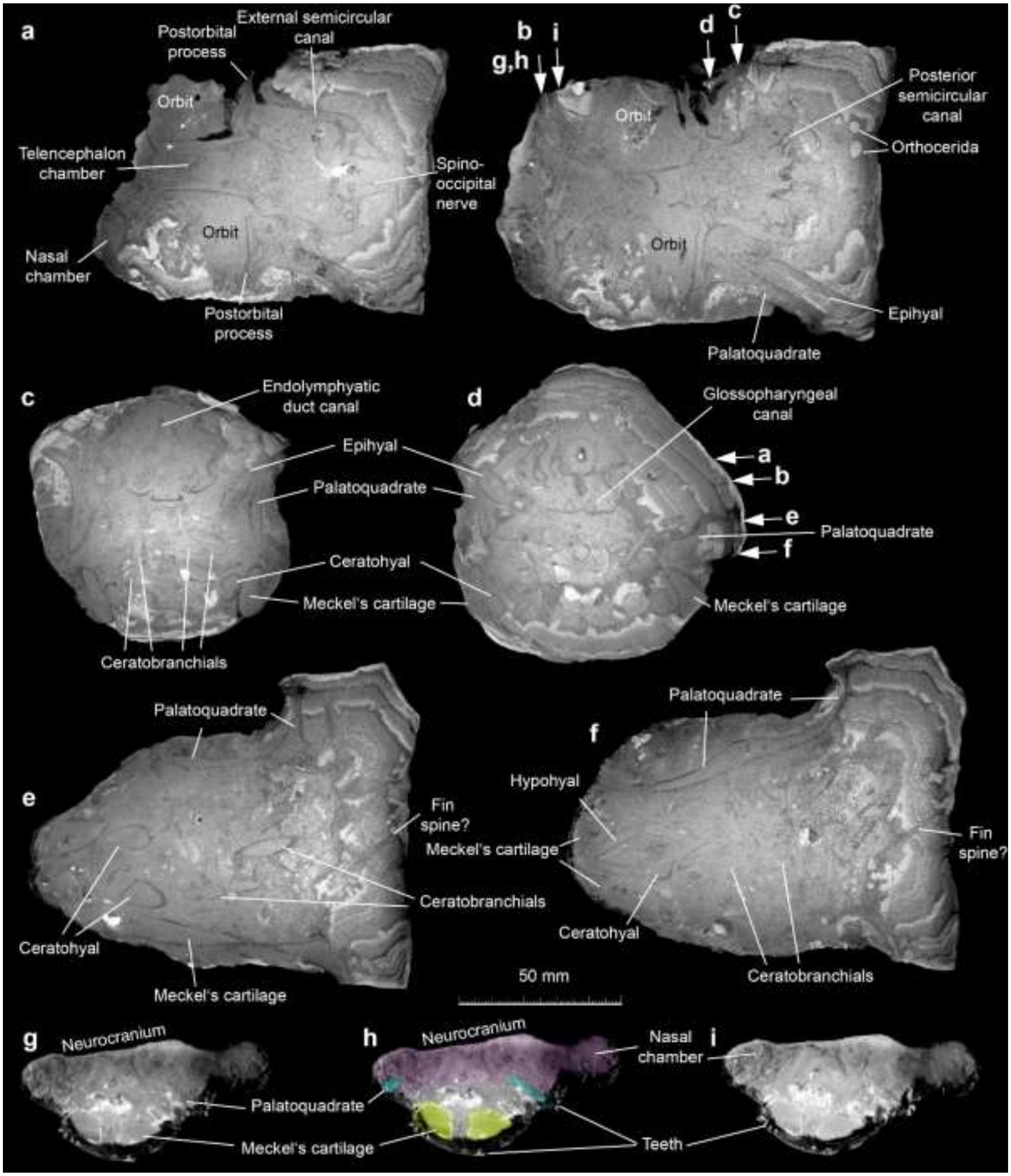

Supplementary Fig. 29 Four horizontal (**a, b, e, f**) and two vertical slices (the latter two perpendicular to the plain of symmetry; **c, d**) from the CT image-stack of the 3D-skull of *Maghriboselache mohamezanei* n. gen. et sp., PIMUZ A/I 5159. The relative positions of the slices are marked in b and d by arrows.

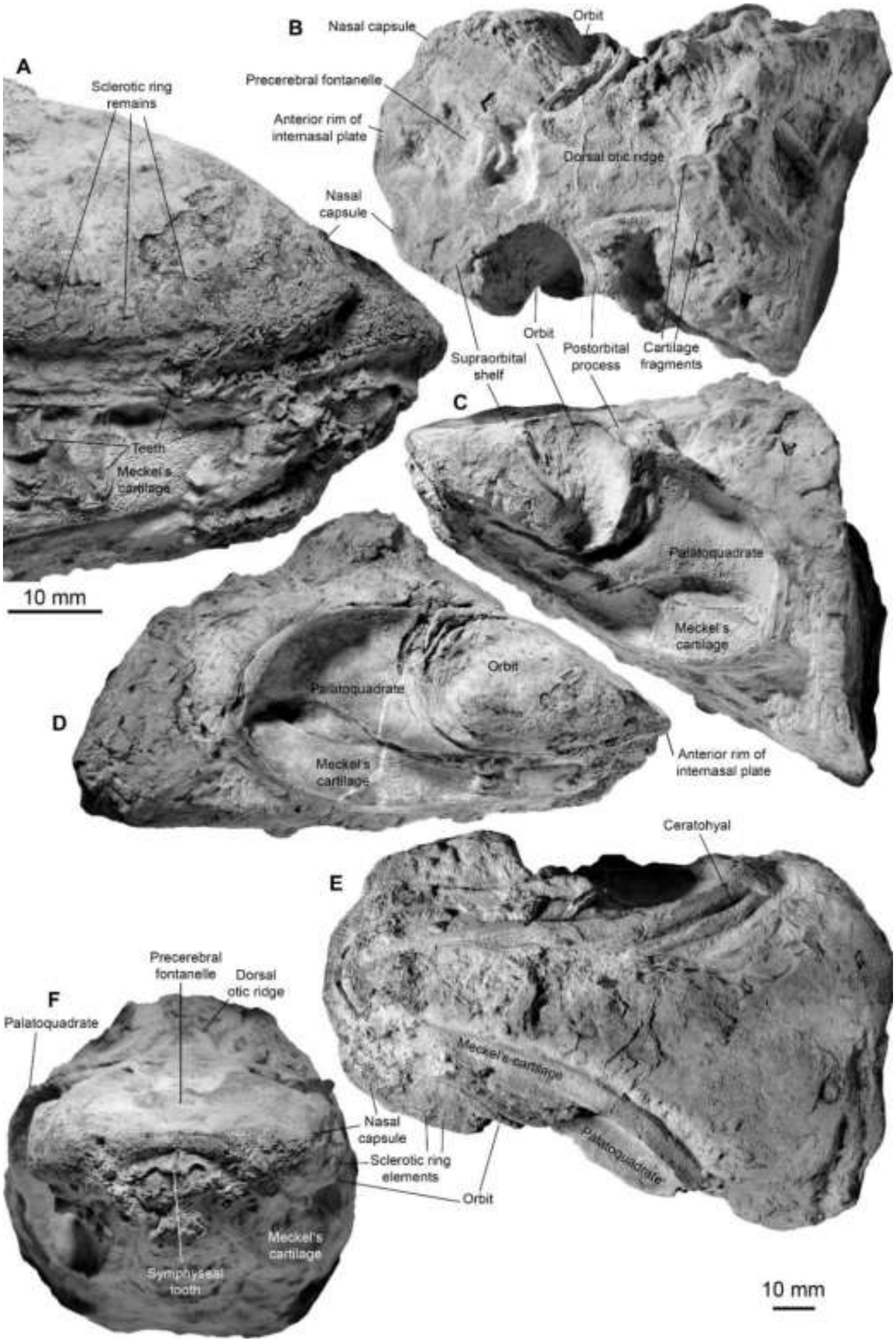

Supplementary Fig. 30 3D-skull of *Maghriboselache mohamezanei* n. gen. et sp., PIMUZ A/I 5159, whitened with NH_4Cl. **A** right detail; **B** dorsal; **C** left; **D** right; **E** ventral, **F** anterior view.

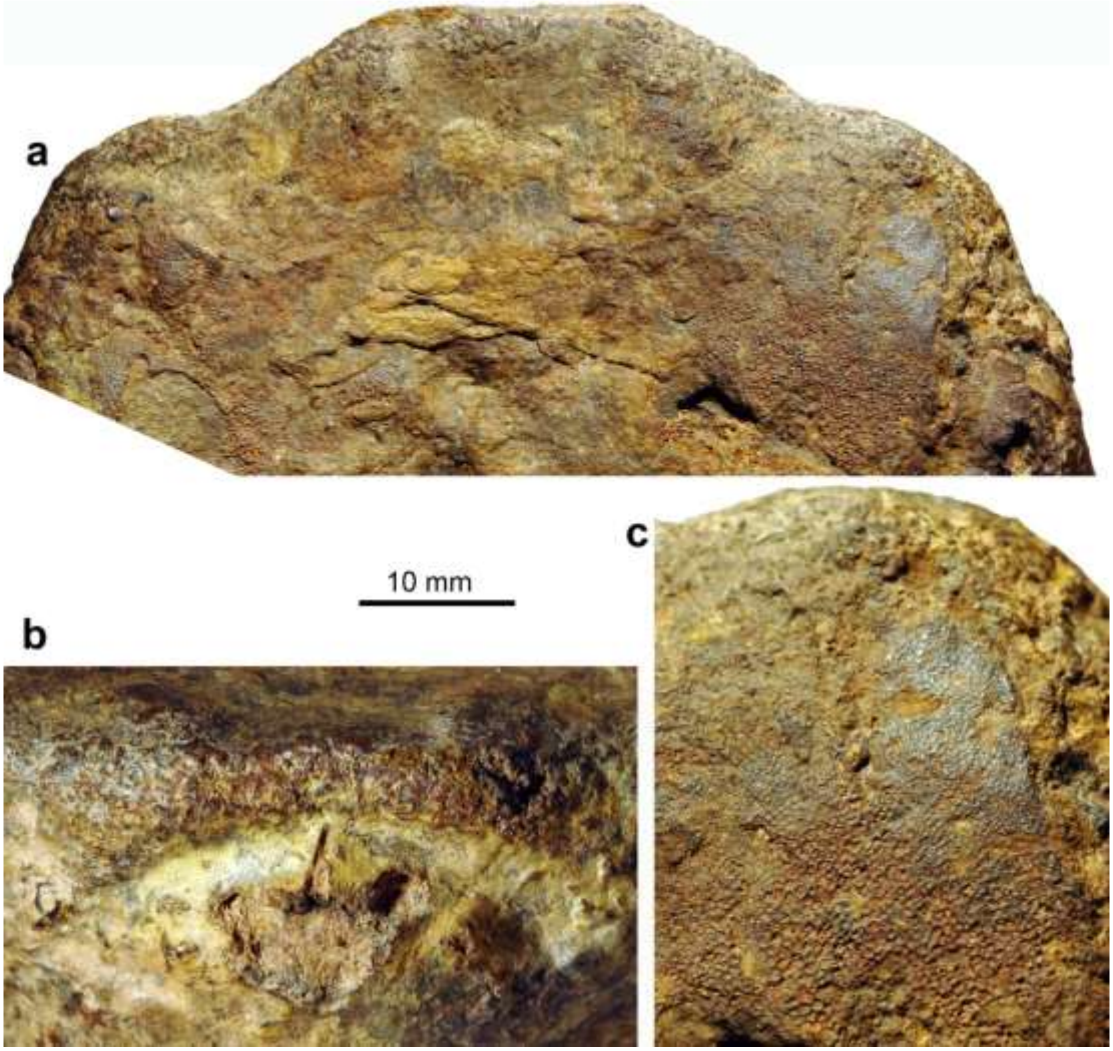

Supplementary Fig. 31 Details of the ethmoid region of the 3D-skull of *Maghriboselache mohamezanei* n. gen. et sp., PIMUZ A/I 5159. **a** the snout with the nasal capsules and the narial openings in dorsal view. **b** detail in anterior view showing the large weathered symphyseal tooth (distinct median cusp, faint imprints of two pairs of lateral cusps); some smaller teeth are partially exposed on the left. **c** enlarged detail of a to show the slightly weathered tessellated cartilage (scale bar equals 20 mm).

The orbits measure about 35 mm in length, 25 mm in height and are approximately 20 mm deep. The postorbital process is distinctly vaulted in all directions and the supraorbital shelf appears to cover much of the orbit. The postorbital processes have a lateral span of about 78 mm. As far as visible in the image stack, the supraorbital shelf covered much of the orbit, while the suborbital shelves are minimal like in, e.g., *Akmonistion* (Coates and Sequeira 2001).

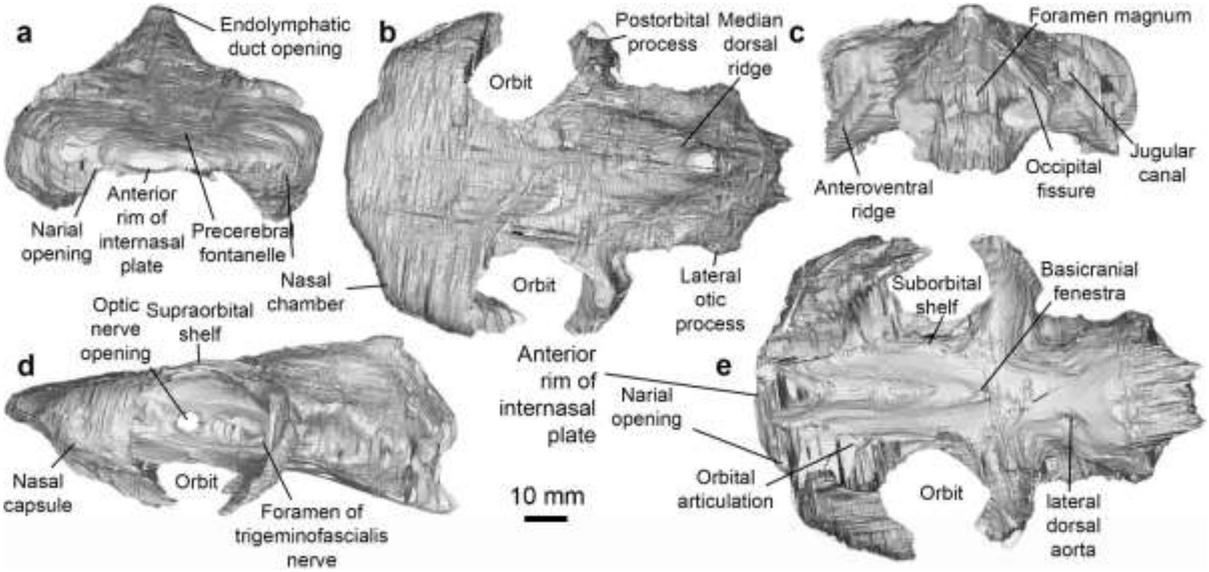

Supplementary Fig. 32 Rendered images of the neurocranium of the 3D-skull of *Maghriboselache mohamezanei* n. gen. et sp., PIMUZ A/I 5159, based on CT-images. **a** anterior, **b** dorsal, **c** posterior, **d** left and **e** ventral views.

The otic-occipital region is only moderately well visible in the images of the CT-scan. All semicircular canals are well discernible in the image stack. The anterior and posterior semicircular canals form the characteristic cross in dorsal aspect (Supplementary Fig. 35). Longitudinally, they span 35 mm and laterally 32 mm. They are about 2 to 3 mm thick. The horizontal semicircular canal is slightly thinner. Unsurprisingly, the proportions and arrangement resemble that of *Dwykaselachus* (Coates et al. 2017) quite closely. Also like in *Dwykaselachus* (Coates et al. 2017), the otic region is shorter than in Devonian stem elasmobranchs such as *Cladodoides*, *Orthacanthus*, or *Phoebodus* (Coates et al. 2017; Frey et al. 2019; Maisey 2007).

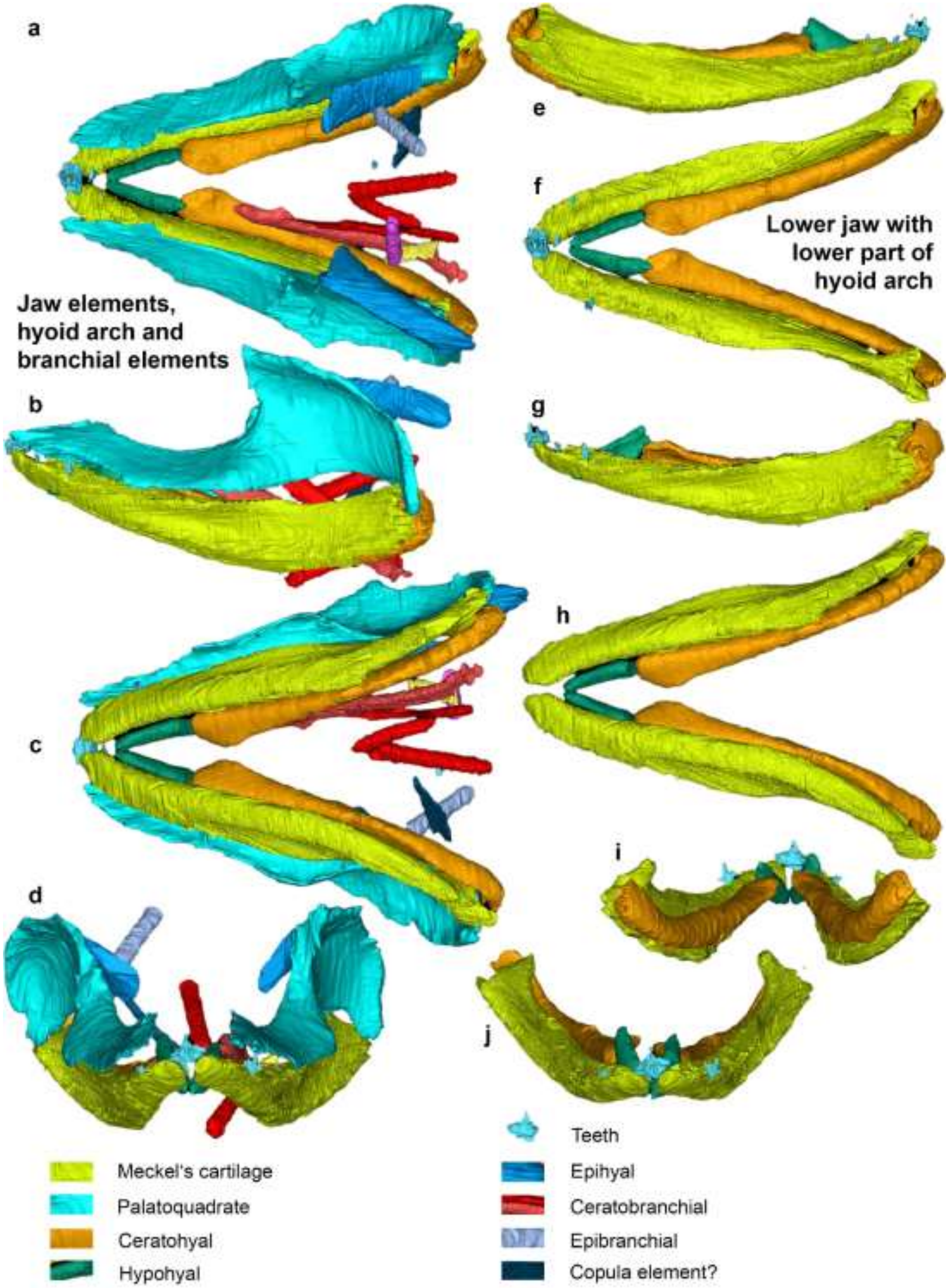

Supplementary Fig. 33 Segmented data of the jaw, hyoid and gill arches of *Maghriboselache mohamezanei* n. gen. et sp., PIMUZ A/I 5159. a to c shows all elements; **a** dorsal, **b** left, **c** ventral, **d** posterior views. **d** to **j** shows only the lower elements, **e** right, **f** dorsal, **g** left, **h** ventral, **i** posterior, **j** anterior views.

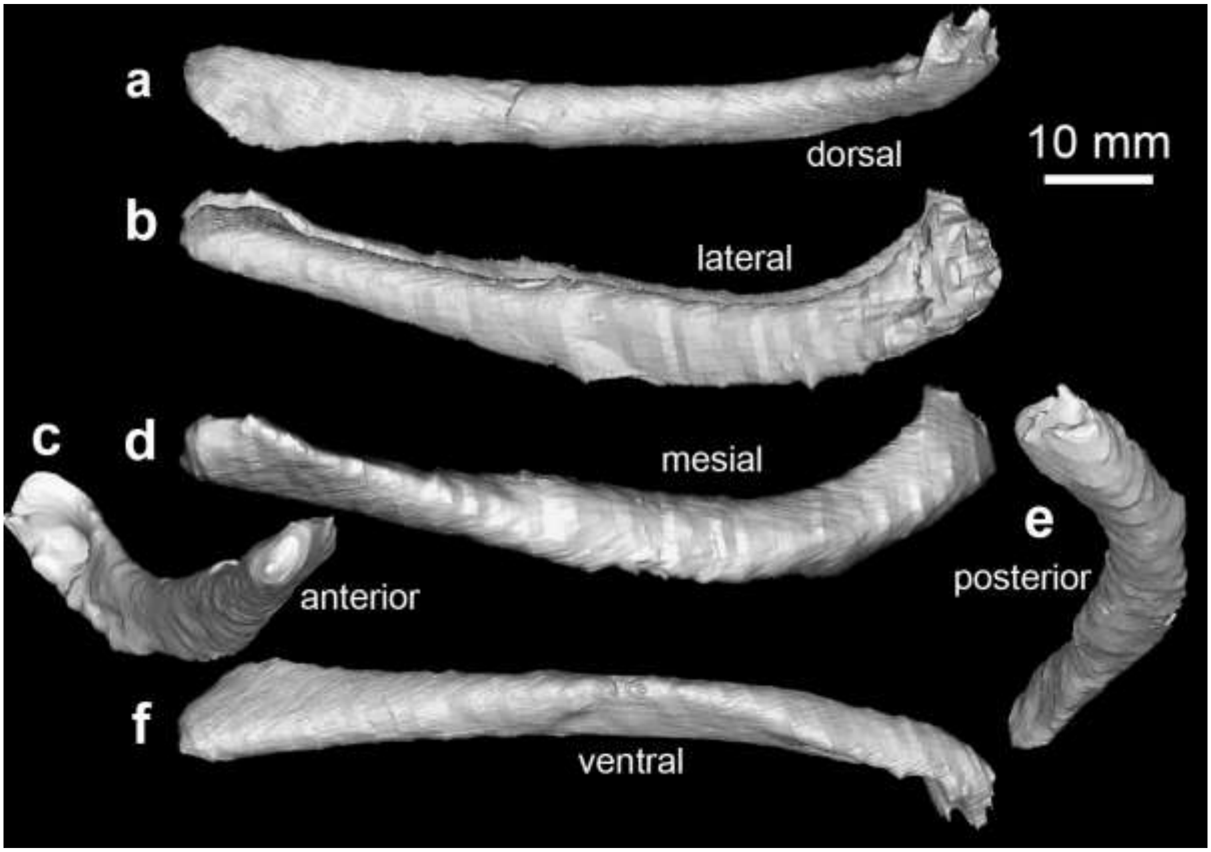

Supplementary Fig. 34 Segmented data of the ceratohyal of *Maghriboselache mohamezanei* n. gen. et sp., PIMUZ A/I 5159.

Meckel’s cartilages measure 107 mm from the articulation to the symphysis (Supplementary Fig. 33). It is up to 10 mm wide and 17 mm high). In ventral aspect, these cartilages have a gently sinusoidal shape: Anterior of the articulation, the rami are laterally concave until close to the symphysis, where they turn towards each other (Supplementary Fig. 39). The presence of a symphyseal tooth row consisting of larger symmetrical teeth, which are already visible from the outside, provides evidence for a stiff sysmphysis in contrast to *Ferromirum* (Frey, Coates, et al. 2020). The ventral edge of the rami is quite concave in its posterior half and becomes straighter anteriorly. This straighter part roughly corresponds to the tooth-bearing portion, which is also evident from the flattened dorsal side of both rami. The two cartilages form an angle of around 30 degrees, which is surprisingly similar to *Phoebodus* (Frey et al. 2019), although the neurocranium is much broader and shorter than in *Phoebodus*.

The palatoquadrates display the classic hatchet shape with the dorsoventrally flattened palatine ramus and the quite strongly vaulted quadrate portion (Supplementary Fig. 33). The posterior half is about 32 mm high and about 15 mm deep in horizontal direction. It is of a similar length as the Meckel's cartilage. The palatine ramus is a rather thin cartilage sheet, which is dorsally concave and about 7 mm wide below the center of the orbit, slightly expanding laterally toward the anterior edge of the orbit. The anterior ends of the palatine rami are not very well discernible in the CT-images, and hence, the shape of the anterior end is poorly known. Behind the tooth-bearing part, the ventral edge of the palatoquadrate is concave, thereby forming a lenticular gap between upper and lower jaw (Supplementary Fig. 30, 33).

Ceratohyal and hypohyal are perfectly visible and still in articulation (Supplementary Fig. 33, 34). The ceratohyals are 85 mm long. At the articulation, they are nearly 8 mm wide, then they taper anteriorly to a width of 4 mm and widen again towards their anterior ends to about 9 mm, before they touch the hypohyals. The hypohyals are almost 20 mm long and less than 5 mm wide, with a cross section that tapers slightly ventrally. They fit quite well into the lingual side of Meckel's cartilages.

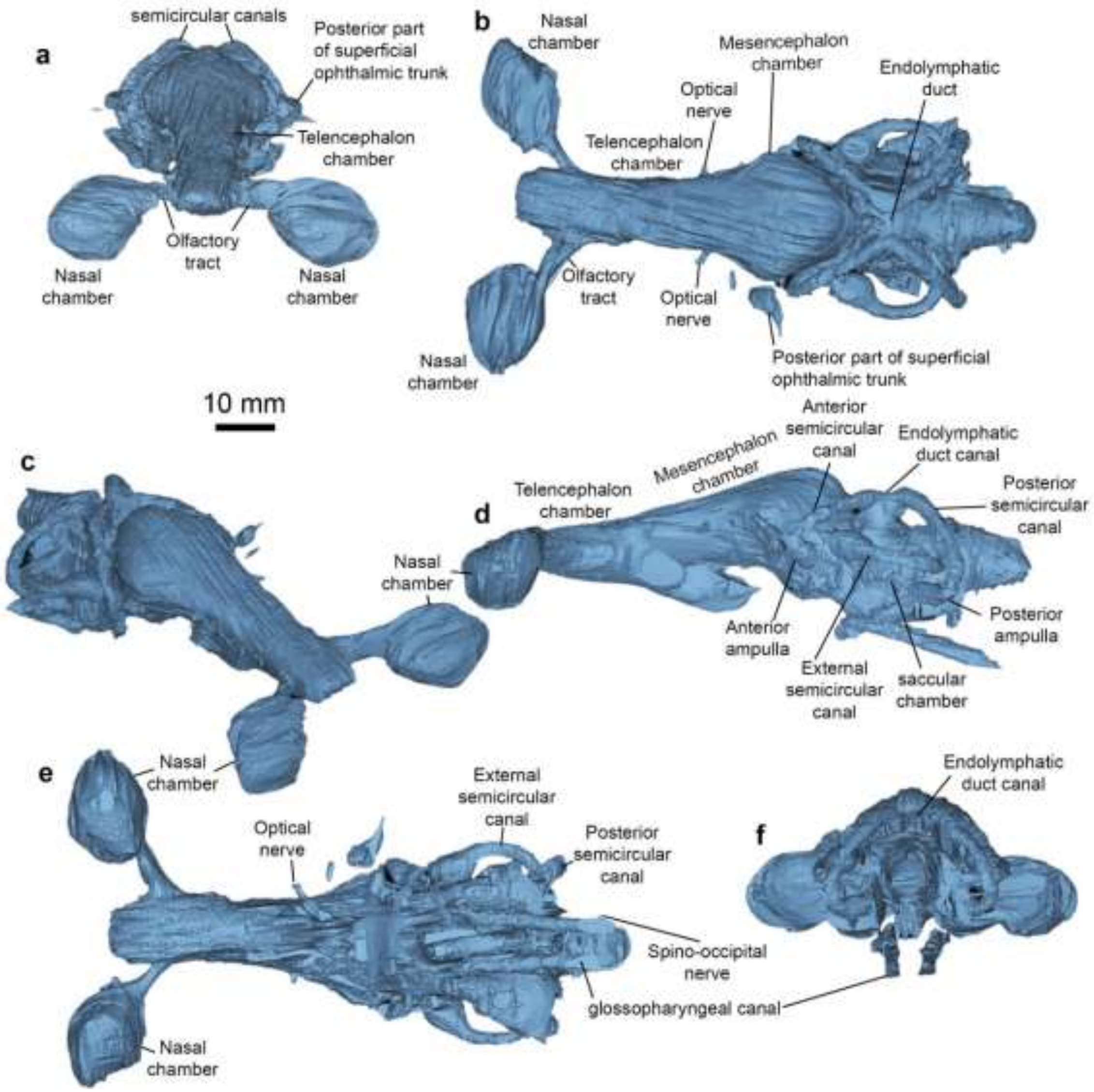

Supplementary Fig. 35 Segmented data of *Maghriboselache mohamezanei* n. gen. et sp., PIMUZ A/I 5159, showing the endocast. **a** anterior, **b** dorsal, **c** oblique anterior, **d** left lateral, **e** ventral and **f** posterior views.

PIMUZ A/I 5160 (Fig. 6, Supplementary Fig. 36)

Locality: Madene El Mrakib, Maïder.

Stratigraphic position: Thylacocephalan Layer, early Middle Famennian.

Finder: Saïd Oukherbouch (Tafraoute)

Preparator: not prepared. The dentition weathered out of the nodule naturally.

Description: PIMUZ A/I 5160 is a nearly complete skeleton, which is flattened and about 1.10 m long. It is mainly remarkable because of the naturally exposed teeth (Fig. 6). The teeth are typically cladodont with a long main cusp and two lateral cusps on each side. The main cusp is finely striated with a flat labial side, that is slightly concave towards its base. The lateral cusps are slightly inclined towards the main cusp and those lying closer to the main cusp are the shortest. The largest teeth have a base that is about 12 mm wide and 7.5 mm long in lateral direction. The lingual side displays two oval bosses (Fig. 6). On the labial side, there is the characteristic basolabial depression, neighbored by the facets for the articulation with the adjacent tooth in the same tooth file. In the upper tooth visible in Supplementary Fig. 36, it appears like there were three lateral cusps on both sides, but this appears unusual.

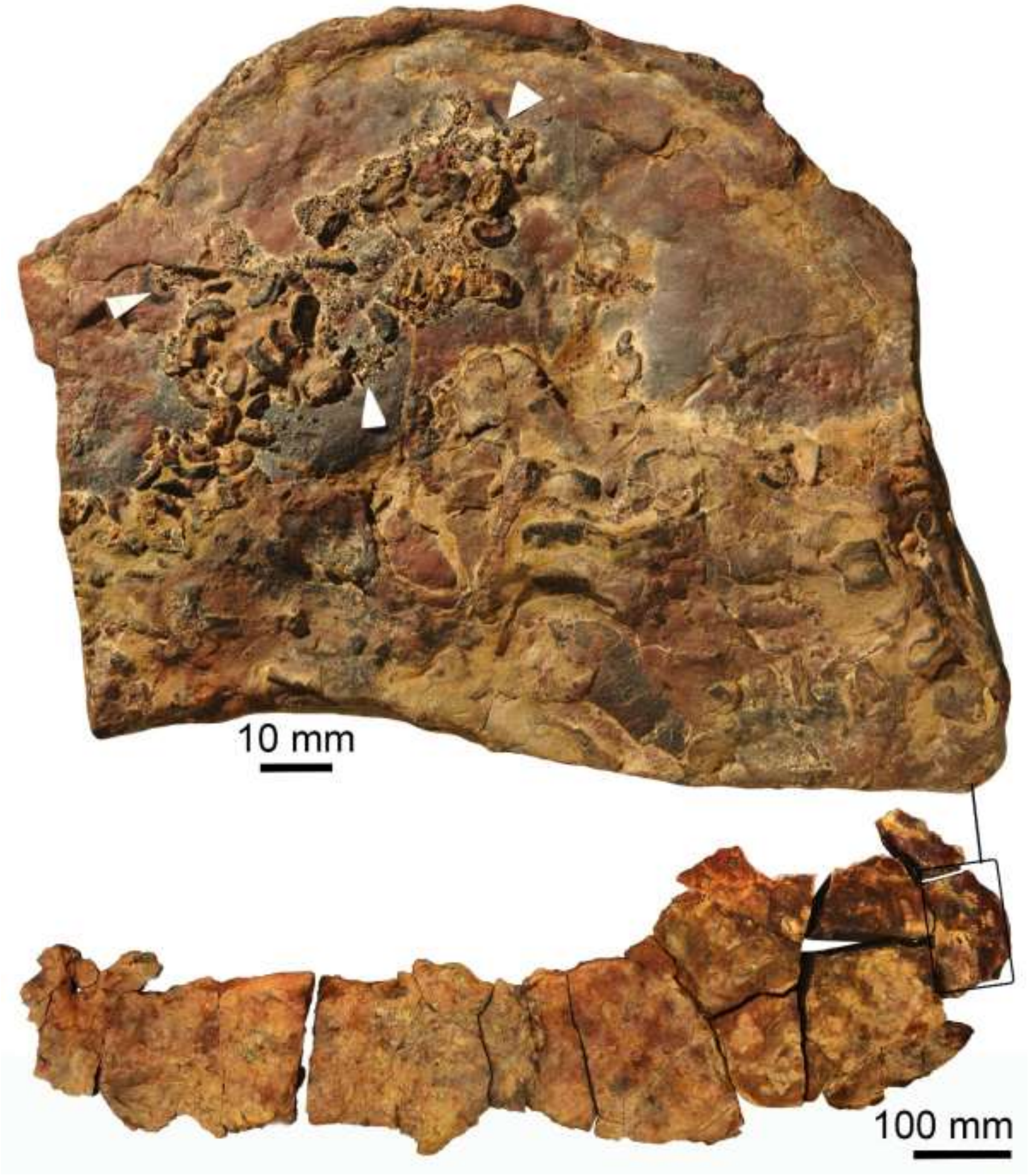

Supplementary Fig. 36 Ventral view of a skull of *Maghriboselache mohamezanei* n. gen. et sp., PIMUZ A/I 5160, with well-exposed dentition. Top, detail of the head region. Bottom: The entire, unprepared skeleton.

PIMUZ A/I 5161 (Supplementary Fig. 37)

Locality: Madene El Mrakib, Maïder.

Stratigraphic position: Thylacocephalan Layer, early Middle Famennian.

Finder: Saïd Oukherbouch (Tafraoute)

Preparator: not prepared. The dentition weathered out of the nodule naturally.

Description: This skull also belongs to a nearly complete, flattened skeleton, which measures about 1 m in length. The skull is flattened (Supplementary Fig. 37) and exposes two teeth each of five tooth files, which are still in situ.

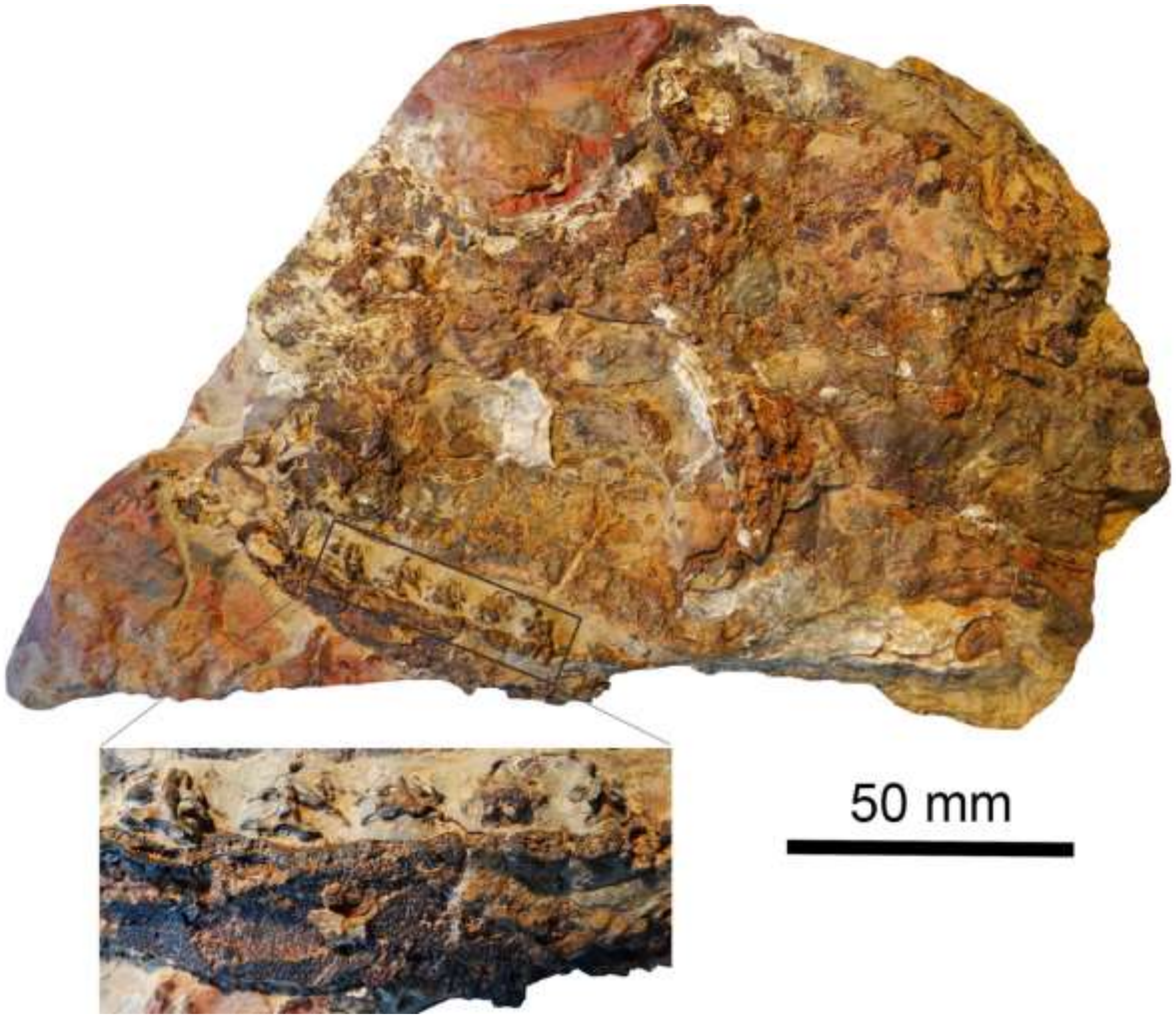

Supplementary Fig.37 Ventral view of a skull of *Maghriboselache mohamezanei* n. gen. et sp., PIMUZ A/I 5161, with well-exposed dentition.

PIMUZ A/I 5162 (Supplementary Fig. 38)

Locality: Tamjerant, Tafilalt (31°00’02.2”N, 4°03’33.3”W).

Stratigraphic position: Thylacocephalan Layer, early Middle Famennian.

Finder: Mohamed Mezane (El Khraouia)

Preparator: not prepared. The dentition weathered out of thee nodule naturally.

Description: This strongly corroded skull is 170 mm long and 92 mm wide. The neurocranium and both palatoquadrates are largely eroded away, but the palatal rami are still present, retaining much of the dentition. Remains of both Meckel’s cartilages are also discernible, but it is the dentition, which makes this specimen remarkable. It appears like remains of all eleven tooth files of upper and lower jaw are preserved on the left side. It is also noteworthy that on the left, most teeth are corroded, displaying a whitish color, while those of the right side and the symphyseal tooth files are black, showing the cladodont tooth morphology reasonably well.

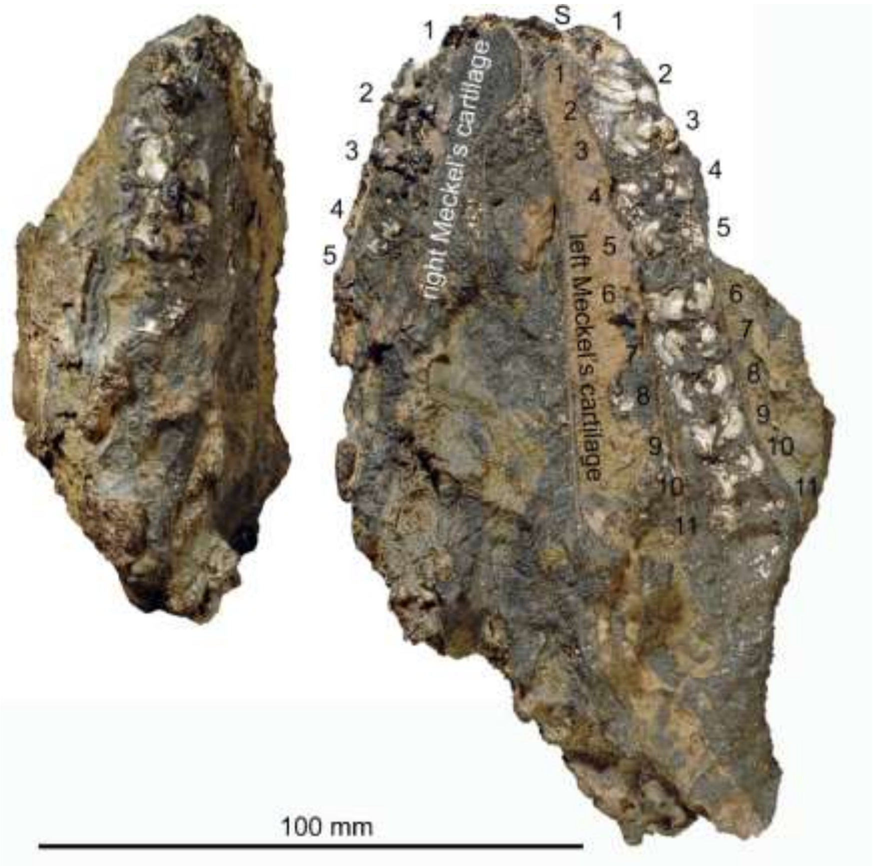

Supplementary Fig. 38 Ventral view of a weathered skull of *Maghriboselache* PIMUZ A/I 5162, with well-exposed dentition (whitish).

AA.TJR.DS.1 (Supplementary Fig. 39)

Locality: Tamjerant, Tafilalt (31°00'02.2"N, 4°03'33.3"W).

Stratigraphic position: Thylacocephalan Layer, early Middle Famennian.

Finder: Mohamed Mezane (El Khraouia)

Preparator: not prepared. The dentition weathered out of the nodule naturally.

Description: This is a limestone nodule, which is heavily corroded by Aeolian erosion telling from the erosional facets and polished surface. The nodule is 163 mm long and 151 mm wide. The posterior edge shows the cross section to the jaws and the otic region of the neurocranium. The skull is largely three dimensionally preserved, but slightly obliquely deformed.

This specimen is mainly described here for its dentition. Although the single tooth files are less well organized and articulated as in the specimen described above, it appears like there were also 11 tooth files plus the symphyseal tooth file in the lower jaw. All teeth are strongly corroded and display the light greyish to white color of weathered apatite.

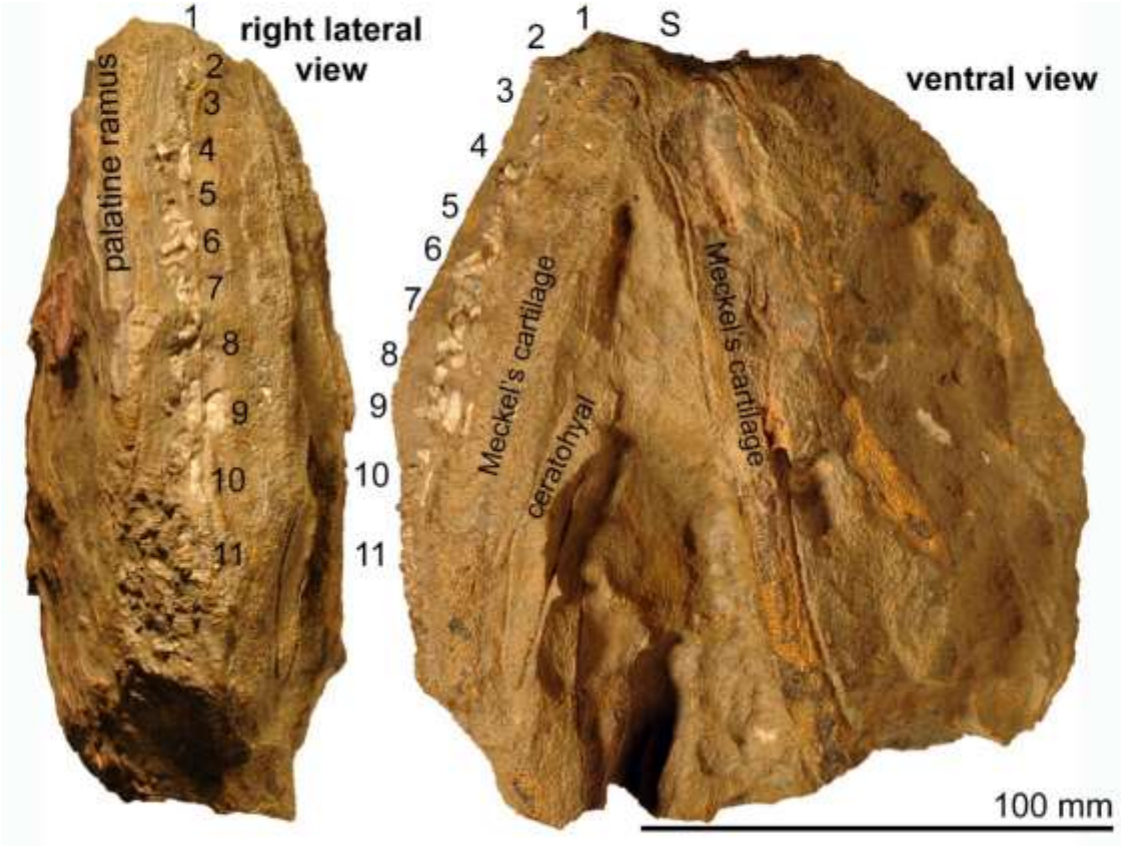

Supplementary Fig. 39 Ventral view of a skull of *Maghriboselache* AA.TJR.DS.1 with well-exposed dentition.

AA.MEM.DS.7 (Supplementary Fig. 40)

Locality: Madene El Mrakib

Stratigraphic position: Thylacocephalan Layer, early Middle Famennian.

Finder: Saïd Oukherbouch (Tafraoute)

Preparator: not prepared. The sclerotic ring and some cartilages weathered out of the nodule naturally.

Description: This skull is still embedded in a large ferric nodule and only slightly deformed. The nodule measures 176 times 150 mm, but the chondrichthyan remains make up only about one third of the nodule. It is mainly remarkable because it is the only specimen preserving much of the semi-disarticulated sclerotic ring. The single plates are 4 to 6 mm long and 3 to 5 mm wide. In total, only 13 plates are visible, but as shown by Dean (B. Dean 1909), there were probably over 80 plates per eye in *Cladoselache kepleri*.

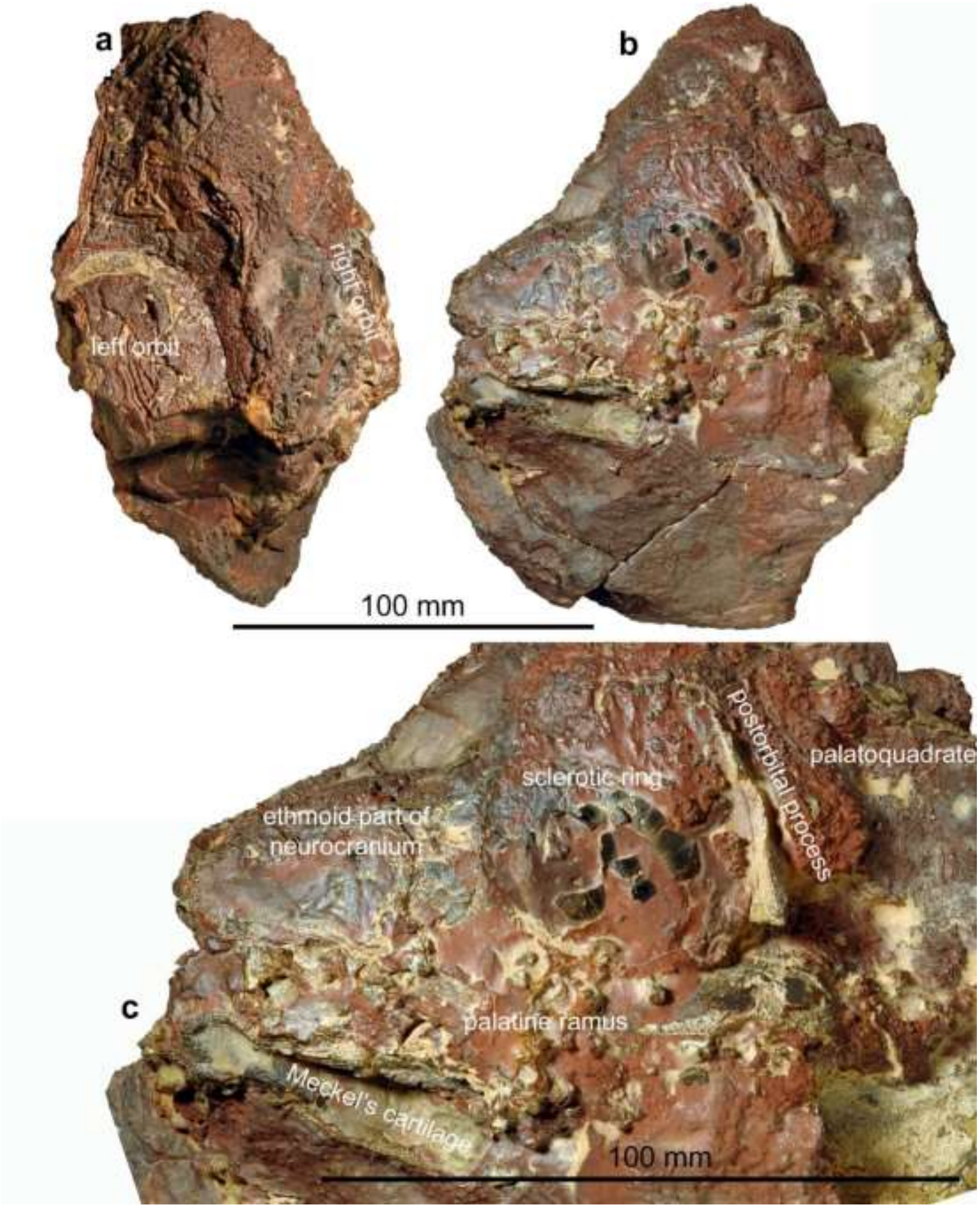

Supplementary Fig. 40 Ventral view of a skull of *Maghriboselache* AA.MEM.DS.7 with well-exposed dentition.

Remarks on phylogenetic analyses

(Supplementary Figs. 41 to 42)

As often around the origin of clades, similarities between early representatives of sister clades are often widespread and hence, these taxa tend to be quite unstable, causing changes in sister group-relationships. To illustrate this, we show some recently proposed phylogenies in the figure below.

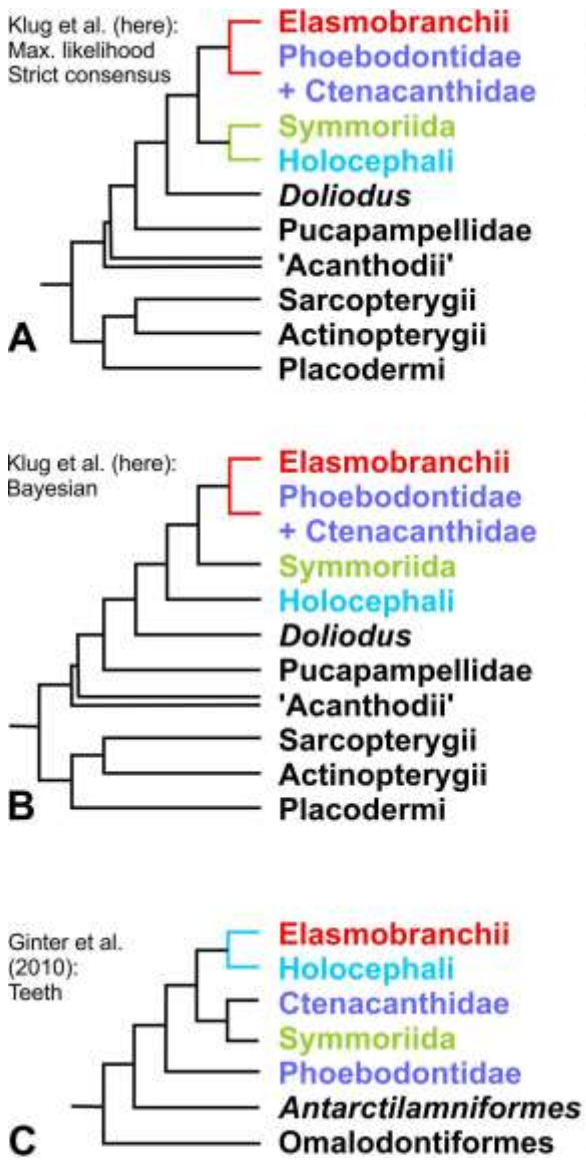

Supplementary Fig. 41 Examples of early vertebrate phylogenies from this paper and other recent publications to demonstrate the contradictions in relationships. These root at least partially in different methods and data sets.

Bayesian tip-dating analyses were performed in the software BEAST 2.6.3 (Bouckaert et al. 2019). We used the fossilized birth-death model with the parametrization of net diversification rate ($d = \lambda - \mu$), turnover rate ($r = \mu / \lambda$) and sampling proportion ($s = \psi / (\mu + \psi)$) (Gavryushkina et al. 2014; Heath et al. 2014; Stadler 2010). Because the low number of post-Paleozoic taxa would introduce a strong bias in sampling rates, potentially impacting the results, we only kept Paleozoic taxa for these analyses, in total 59 taxa. Character evolution was modelled by the Mkv model with invariant site correction (*78*), partitioning characters according to their number of states and their exchangeability values set to half the number of states (King et al. 2016). We applied four discrete gamma distributed rate categories to account for rate heterogeneity. As a morphological clock model, we chose a relaxed log normal clock with an exponential prior (mean = 1.0) on mean clock rate and a gamma distributed prior ($\alpha = 0.5396$, $\beta = 0.3819$) on its standard deviation. We set up an exponential prior on origin time with a mean of 1.0 my and an offset of 423.0 my, corresponding to the upper age limit of the oldest taxon in the dataset, Guiyu (Zhu et al. 2009). This is consistent with previous studies, which reconstruct the clade including all taxa used here to originate not long before the appearance of Guiyu, while still allowing for an older origin to be sampled. Another exponential prior was set on diversification rate (mean = 1.0), while we used uniform priors on turnover rate and sampling proportion, both limited between 0.0 and 1.0. Stratigraphic age uncertainty was modelled by allowing tips to vary according to their stratigraphic ranges (Barido-Sottani et al. 2019). The MCMC was run for 25,000,000 generations in three independent runs, sampling every 10,000 generation. 10% of the samples were discarded as burn-in. The log-files from each run were checked for convergence in Tracer (Rambaut et al. 2018) and the tree-files were combined in LogCombiner (Bouckaert et al. 2019). MCC trees were produced in TreeAnnotator version 1.10.4. (Drummond et al. 2012; Drummond and Rambaut 2007) instead of 2.6.3, as it is currently better able to handle trees that include sampled ancestors with variable tip dates.

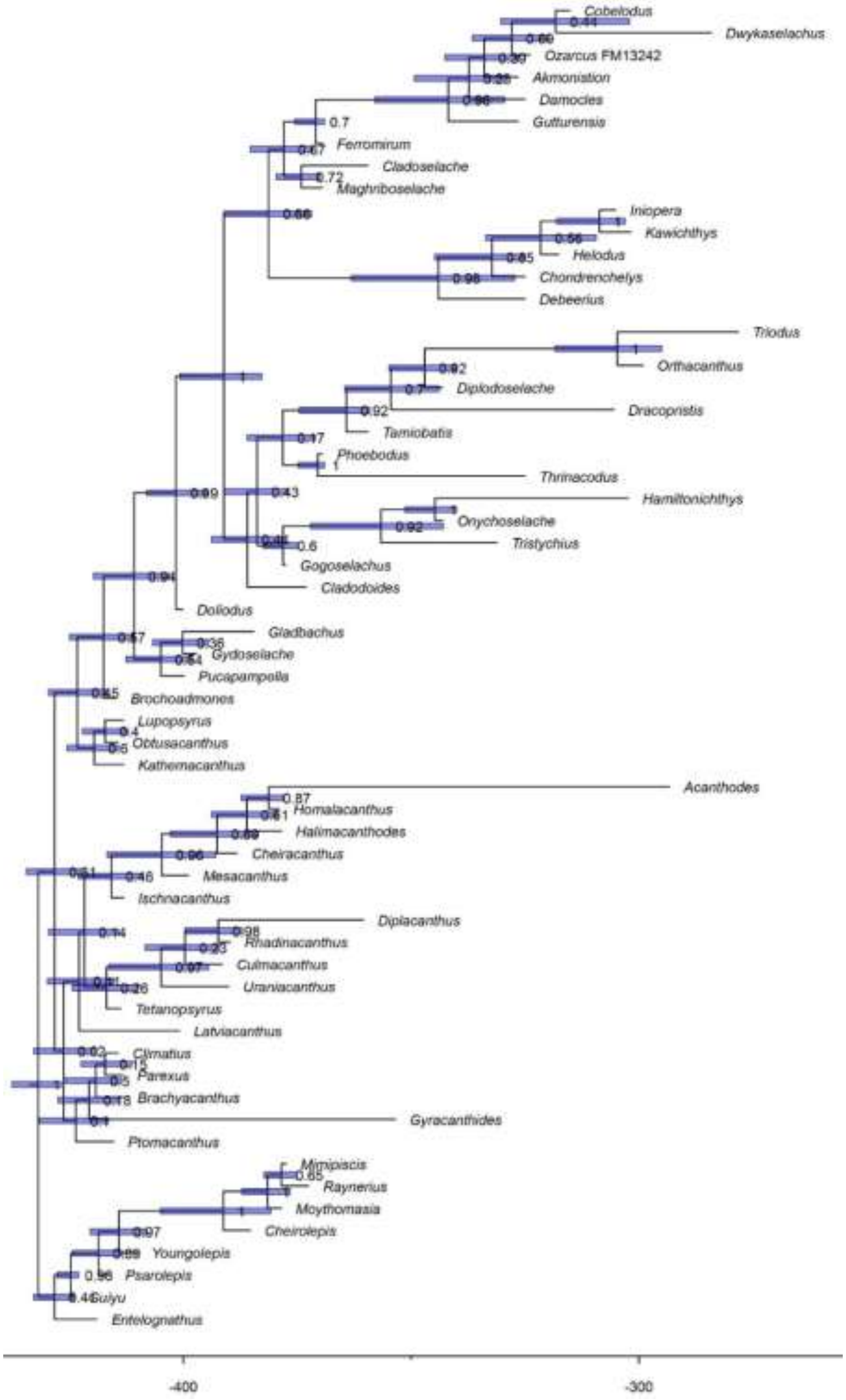

Supplementary Fig. 42 Phylogeny based on the emended character matrix from Coates et al. (Coates et al. 2017) and Frey et al. (Frey et al. 2019). Bayesian tip-dating analyses were performed using the fossilized birth-death model. Character matrix with 238 characters (pruned dental characters).

Taxon and Character lists

Nexus files:

Maghriboselache_grouped_Gogo_Dracopristis_8_new_char.nex

This matrix is based on Frey et al. (Frey, Coates, et al. 2020), which is based on Coates et al. (Coates et al. 2017). We additionally coded *Dracopristis hoffmanorum* Hodnett et al., 2021. Further, we added eight characters describing body proportions etc. For details see the character list, characters 231 to 238. We further added characters 239 to 261 from Hodnett et al. (Hodnett et al. 2021), which are predominantly dental characters. The latter caused a bias towards dental characters.

Maghriboselache_grouped_Gogo_Dracopristis_8_new_char_withoutDental.nex

This is the same matrix as above, but without the characters 239 to 261 from Hodnett et al. (Hodnett et al. 2021).

Maghriboselache_grouped_Gogo_Dracopristis_withoutnew.nex

This is the same matrix as above, but without the new characters 231 to 238 and without characters 239 to 261 from Hodnett et al. (Hodnett et al. 2021).

Maghriboselache_grouped_Gogo_Dracopristis_dentalpruned.nex

This is largely the same matrix as the first, but with only selected characters from 231 to 261.

Taxon list updates relative to Frey *et al*. matrix (Frey et al. 2019)

Dracopristis hoffmanorum Hodnett et al. (Hodnett et al. 2021)

Taxa and Sources

Acanthodes: (Beznosov 2009; Brazeau and de Winter 2015; Coates 1994; Davis et al. 2012; U. Heidtke 1993; Heidtke 2011a, 2011b; Jarvik 1977, 1980; Miles 1971, 1973a, 1973b; Nelson 1968; Watson 1937)

Acronemus: (Maisey 2011; Rieppel 1982)

Akmonistion: (Coates 1998; Coates and Sequeira 1998, 2001; Coates et al. 2017)

Brachyacanthus: (Denison 1979; Miles 1973a; Watson 1937)

Brochoadmones: (Bernacsek and Dineley 1977; Gagnier and Wilson 1996a; Hanke and Wilson 2006)

Callorhinchus/Hydrolagus: (Cole 1897; De Beer and Moy-Thomas 1935; DeBeer 1937; Didier 1995; Didier et al. 1994, 2012; Howard et al. 2013; Kesteven 1933; Patterson 1965; Pradel et al. 2013; Stahl 1999)

Cheiracanthus: (Denison 1979; Miles 1973a; Watson 1937)

Cheirolepis: (Arratia and Cloutier 1996; Giles, Friedman, et al. 2015; Pearson and Westoll 1979)

Chondrenchelys: (Finarelli and Coates 2012, 2014; Lund 1982; Moy-Thomas 1935)

Cladodoides: (Gross 1937, 1938; Maisey 2005)

Cladoselache: (Bendix-Almgreen 1975; Harris 1938a, 1938b; Maisey 1989, 2007; Schaeffer 1975; Williams 2001; Woodward and White 1938)

Climatius: (Miles 1973a, 1973b; Watson 1937)

Cobelodus: (Zangerl and Case 1976; Zidek 1992)

Culmacanthus: (Long 1983)

Damocles: (Lund 1986)

Debeerius: (Grogan and Lund 2000)

Diplacanthus: (Gagnier 1996; Miles 1973a; Watson 1937)

Diplodoselache: (Dick 1981)

Doliodus: (Long et al. 2015; Maisey et al. 2009; Maisey et al. 2013, 2017; Miller et al. 2003)

Dracopristis: (Hodnett et al. 2021)

Dwykaselachus: (Coates et al. 2017; Oelofsen 1986)

Egertonodus: (Lane 2010; Maisey 1982, 1983)

Entelognathus: (Zhu et al. 2013)

Ferromirum: (Frey, Coates, et al. 2020)

Gladbachus: (Burrow and Turner 2013; Coates 2005; Coates and Tietjen 2018; Heidtke 2009; Heidtke and Krätschmer 2001)

Gogoselachus: (Long et al. 2015)

Guiyu: (Zhu et al. 2009)

Gutturensis: (Sequeira and Coates 2000)

Gydoselache: (Maisey et al. 2019)

Gyracanthides: (Miles 1973b; Turner et al. 2005; Warren et al. 2000)

Halimacanthodes: (Burrow et al. 2012)

Hamiltonichthys: (John G. Maisey 1989)

Helodus: (Coates et al. 2021; Johanson et al. 2021; Patterson 1965; Stahl 1999)

Homalacanthus: (Gagnier 1996; Watson 1937)

Homalodontus: (Mutter et al. 2007, 2008)

Iniopera: (Pradel et al. 2009, 2010, 2010; Zangerl and Case 1976)

Ischnacanthus: (Miles 1973a, 1973b)

Kawichthys: (Pradel et al. 2011)

Kathemacanthus: (Gagnier and Wilson 1996b; Hanke and Wilson 2004)

Latviacanthus: (Schultze and Zidek 1982) Schultze & Zidek 1982.

Lupopsyrus: (Bernacsek and Dineley 1977; Hanke and Davis 2012)

Maghriboselache: this paper

Mesacanthus: (Miles 1973a; Watson 1937)

Mimipiscis: (Choo 2012; Gardiner 1984; Gardiner and Bartram 1977; Giles and Friedman 2014)

Moythomasia: (Coates et al. 2017; Gardiner 1984; Gardiner and Bartram 1977) specimen MV P222915.

Obtusacanthus: (Hanke and Wilson 2004) specimen UALVP 41488.

Onychoselache: (Coates and Gess 2007; Dick and Maisey 1980)

Orthacanthus: (Hampe 2002; Heidtke 1982, 1999; Hotton 1952; Lane and Maisey 2009; John Graham Maisey 1983; Schaeffer 1981)

Ozarcus and FMNH PF 13242: (Coates et al. 2017; John G. Maisey 2007; Pradel et al. 2014)

Parexus: (Miles 1973a; Watson 1937)

Phoebodus: (Frey et al. 2019)

Psarolepis: (Qu et al. 2013; Yu 1998; Zhu et al. 1999; Zhu and Schultze 1997)

Ptomacanthus: (Brazeau 2009, 2012; Dearden et al. 2019; Denison 1979; Miles 1973b)

Pucapampella: (Janvier and Maisey 2010; Maisey 2001; Maisey et al. 2019; Maisey and Anderson 2001; Maisey and Lane 2010)

Raynerius: (Giles, Friedman, et al. 2015)

Rhadinacanthus: (C. Burrow et al. 2016)

Squalus: (Gans and Parsons 1981; Marinelli and Strenger 1959; Schaeffer 1981)

Synechodus: (Maisey 1985)

Tamiobatis: (Schaeffer 1981)

Tetanopsyrus: (Gagnier et al. 1999; Gagnier and Wilson 1995; Hanke et al. 2001)

Thrinacodus: (Grogan and Lund 2008)

Tribodus: (Lane 2010; Lane and Maisey 2009, 2012; Maisey and de Carvalho 1997)

Triodus: (Hampe 2002; Heidtke et al. 2004; Soler-Gijón and Hampe 1998)

Tristychius: (Coates et al. 2019; Coates and Gess 2007; Coates and Tietjen 2018; Dick 1978)

Uraniacanthus: (Bernacsek and Dineley 1977; Burrow et al. 2016; Hanke and Davis 2008; Newman et al. 2012)

Youngolepis: (Chang 1982, 1991, 2004; Chang and Yu 1981)

Characters (Supplementary Fig. 43 to S61)

Skeletal tissues

1. **Tessellate calcified cartilage: absent (0); present (1**). (Brazeau 2009; Coates and Sequeira 2001; Coates and Sequeira 2001; Davis et al. 2012; M. N. Dean et al. 2009; M. N. Dean and Summers 2006; Lund and Grogan 2004a, 1997, 2004b; Maisey 1984; Maisey 2001, 2013; Pradel et al. 2014; Seidel et al. 2016)

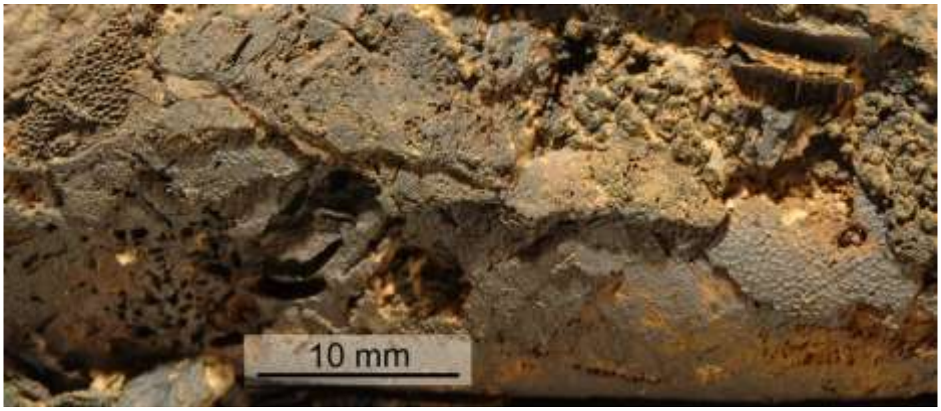

Supplementary Fig. 43 Appearance of tesselate cartilage in Meckel's cartilage of PIMUZ A/I 5154. Note differing appearance on the outside and on the internal cast (right of scale). Squamation remains preserved on the top right.

2. **Perichondral bone: present (0); absent (1).** Janvier (1996); Donoghue & Aldridge (2001); Brazeau (2009); Davis *et al.* (2012).

3. **Extensive endochondral ossification: absent (0); present (1).** (Brazeau 2009; S. P. Davis et al. 2012; Forey 1980; Gardiner 1984)

4. **Extensive calcified cartilage: absent (0); present (1).** To capture all taxa in which the neurocranium, jaws, hyoid and gill arches, as well as parts of the axial and appendicular skeleton are mineralized in the absence of perichondral bone.

5. **Tubular dentine: absent (0); present (1).** Stahl (1999), see also Patterson (1965): present in chimaeroids, edestids, *Helodus*, and petalodonts, but absent in symmoriids and iniopterygians (Zangerl and Case 1976).

6. **Pore canal network: absent (0); present (1).** (Lu et al. 2016)

7. **Acrodin tooth caps (enameloid cap restricted to crown apex): absent (0); present (1).** (Friedman and Brazeau 2010; Lu et al. 2016; Zhu et al. 2009, 2009)

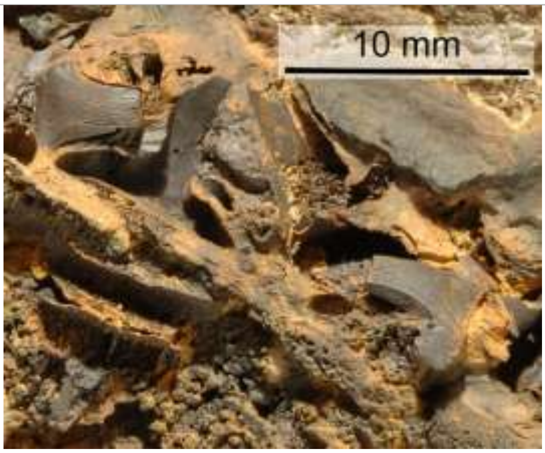

Supplementary Fig.44 Dentition of PIMUZ A/I 5154, partially as external mould, partially broken, showing enameloid covering the whole main cusp and pore canals as internal mould in the center of the image.

Squamation & related structures

8. **Trunk scales monocuspid (0); multicuspid (1).** Revised after Davis *et al.* (S. P. Davis et al. 2012); (C. Burrow et al. 2016; Coates et al. 2018).

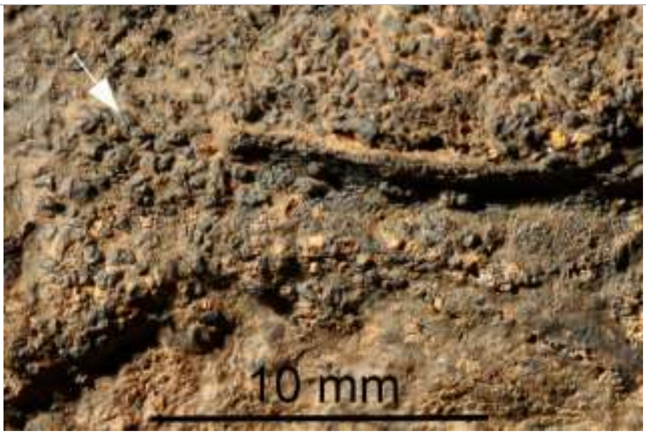

Supplementary Fig. 45 Trunk squamation of PIMUZ A/I 5156. The scales are disarticulated, but some show the multiple cusps (white arrow).

9. **Scale growth concentric: absent (0); present (1).** (Brazeau 2009; Burrow et al. 2016; S. P. Davis et al. 2012; Hanke and Wilson 2004)

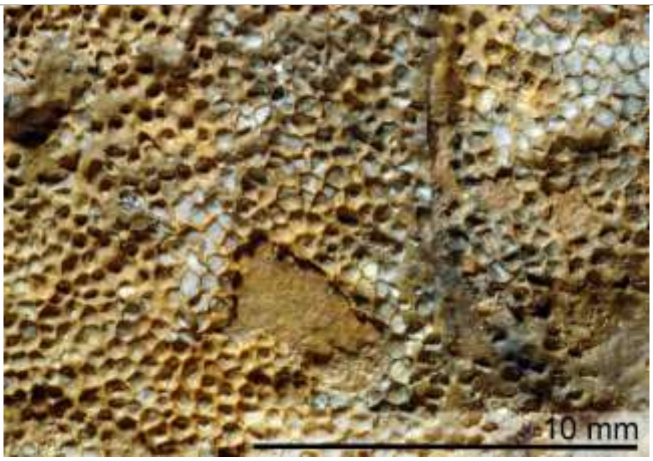

Supplementary Fig. 46 Fin scales in the middle of the pectoral fin of PIMUZ A/I 5154. The scales are eroded partially (light grey, showing faint concentric lines) or entirely (hemispherical moulds of bulging bases).

10. **Peg-and-socket articulation: absent (0); present (1).** (Brazeau 2009; Coates 1999; S. P. Davis et al. 2012; Gardiner 1984)

11. **Anterodorsal process on scale: absent (0); present (1).** (Brazeau 2009; Coates 1999; S. P. Davis et al. 2012; Gardiner 1984; Zhu et al. 2009, 2013)

12. **Body scales with bulging base: absent (0); present (1).** (Brazeau 2009; C. Burrow et al. 2016; S. P. Davis et al. 2012)

13. **Body scales with flattened base: absent (0); present (1).** (Brazeau 2009; C. Burrow et al. 2016; S. P. Davis et al. 2012)

14. **Body scales with basal canal or open basal vascular cavity: absent (0); present (1).**

15. **Neck canal: absent (0) present (1).**

16. **Cranial sensory line canal passes between or beneath scales (0); passes over scales and/or is partially enclosed or surrounded by scales (1); perforates and passes through scales (2).** Character revised after Dearden *et al*. (Dearden et al. 2019).

17. **Postcranial sensory line canal passes between or beneath scales (0); passes over scales and/or is partially enclosed or surrounded by scales (1); perforates and passes through scales (2).** Character revised after Dearden *et al*. (Dearden et al. 2019).

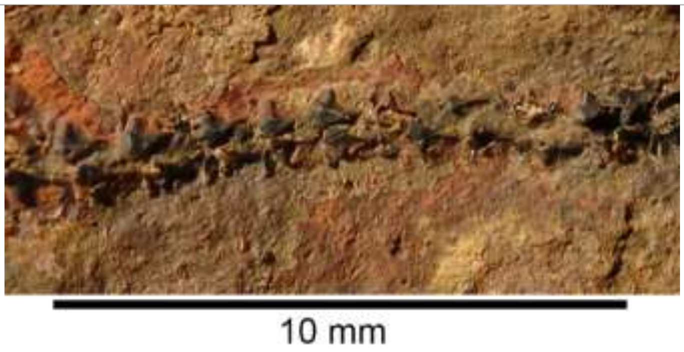

Supplementary Fig. 47 Postcranial sensory line canal is partially enclosed or surrounded by scales of PIMUZ 36884.

18. **Lepidotrichia: absent (0); present (1).**

19. **Fringing fulcra: absent (0); present (1).** (Coates 1999; Zhu et al. 2009, 2013) Zhu *et al.* (2009; 2013); Coates (1999). Scored inapplicable in taxa lacking lepidotrichia.

20. **Scute-like ridge scales (fulcra): absent (0); present (1).** (Giles, Friedman, et al. 2015)

Cranial dermal skeleton

21. **Cranial cap denticles, single-crowned, non-growing: absent (0); present (1).** Scored absent in *Maghriboselache* and *Ferromirum*; if present, it appears likely that they would have been preserved.

22. **Sclerotic ring: absent (0); present (1).** (Burrow et al. 2016; Coates et al. 2018; Giles, Friedman, et al. 2015; Qiao et al. 2016; Zhu et al. 2016)
Rarely preserved in *Cladoselache* and *Maghriboselache* (Supplementary Fig. 35, 36, 44).

23. **Number of sclerotic plates: four or less (0); more than four (1).** (Burrow et al. 2016; Qiao et al. 2016; Zhu et al. 2013, 2016)
In *Maghriboselache*, 13 are preserved, but very likely, there were over 80, like in *Cladoselache*.

The following characters (24 to 48) apply not to crown chondrichthyans.

24. **Dermal skull roof includes large dermal plates (0); consists of plates, tesserae or scales (1); naked or largely scale free (2).** (Brazeau 2009; Brazeau and Friedman 2014; Forey 1980; Gardiner 1984; Zhu et al. 2013)

25. **Dermal ornamentation: smooth (0); parallel, vermiform ridges (1); concentric ridges (2); tuberculate (3).** (Giles, Friedman, et al. 2015) Coded inapplicable where dermal plates absent.

26. **Cranial tessera morphology: large interlocking plates (0); microsquamose, no larger than body squamation (1).** Brazeau (2009) through to Giles *et al.* (Giles, Friedman, et al. 2015). Coded inapplicable where tesserae are absent.

27. **Anterior or mesial edge of nasal notched for anterior nostril: absent (0); present (1)**.

28. **Supraorbital: absent (0); present (1).** (Zhu et al. 2009, 2013)

29. **Broad supraorbital vaults: absent (0); present (1).** (Dennis and Miles 1981; Giles, Friedman, et al. 2015)

30. **Large median bone contributes to posterior margin of skull roof: absent (0); present (1).** (Zhu et al. 2016)

31. **Pineal opening perforates dermal skull roof: present (0); absent (1).** (S. P. Davis et al. 2012; Giles, Friedman, et al. 2015)

32. **Consolidated cheek plates: absent (0); present (1).** (Brazeau 2009; Burrow et al. 2016; J. C. Davis 2002; S. P. Davis et al. 2012; Zhu et al. 2013)

33. **Enlarged postorbital tessera separate from orbital series: absent (0); present (1).** (Brazeau 2009; C. Burrow et al. 2016; S. P. Davis et al. 2012; Zhu et al. 2013)

34. **Dermal intracranial joint: absent (0); present (1).** (Zhu et al. 2009, 2013)

35. **Sensory line network preserved as open grooves (sulci) in dermal bones (0); sensory lines pass through canals enclosed within dermal bones (1).** (J. C. Davis 2002; S. P. Davis et al. 2012; Zhu et al. 2013)

36. **Sensory canal or pit-line associated with maxilla: absent (0); present (1).** (Choo 2012; Friedman 2007; Gardiner 1984)

37. **Jugal portion of infraorbital canal joins supramaxillary canal: present (0); absent (1).** (Brazeau 2009), redefinition in Davis *et al.* (S. P. Davis et al. 2012; Zhu et al. 2013); Zhu *et al.* (2013).

38. **Anterior pit line of skull roof: absent (0); present (1).** (Giles, Friedman, et al. 2015)

39. **Spiracular opening in dermal skull roof bounded by bones carrying otic canal: absent (0); present (1).** (Giles, Friedman, et al. 2015; Lu et al. 2016)

40. **Dermohyal (submarginal) ossification: absent (0); present (1).** Alternative homology hypotheses in Coates *et al*. (Coates et al. 2018).

41. **Branchiostegal series: absent (0); present (1).** (Brazeau 2009; C. Burrow et al. 2016; J. C. Davis 2002; S. P. Davis et al. 2012; Hanke and Wilson 2004; Zhu et al. 2013)

42. **Opercular and subopercular bones: absent (0); present (1).** Scores for opercular bones contingent on branchiostegal series presence.

43. **Branchiostegal plate series along ventral margin of lower jaw: absent (0); present (1).** (Brazeau 2009; J. C. Davis 2002; S. P. Davis et al. 2012; Hanke and Wilson 2004; Lu et al. 2016)

44. **Branchiostegal ossifications plate-like (0); narrow and ribbon-like (1); filamentous (2).** (Brazeau 2009; S. P. Davis et al. 2012; Hanke and Wilson 2004; Lu et al. 2016)

45. **Branchiostegal ossifications ornamented (0); unornamented (1).** (Brazeau 2009; S. P. Davis et al. 2012; Zhu et al. 2013)

46. **Branchiostegals imbricated: absent (0); present (1).** (Brazeau 2009; S. P. Davis et al. 2012; Zhu et al. 2013)

47. **Opercular cover of branchial chamber complete or partial (0); separate gill covers and gill slits (1).** Scores revised after Dearden *et al*. (Dearden et al. 2019), Watson (Watson 1937) and reference to specimen NHMUK P49979.

48. **Gular plates: absent (0); present (1).** (Brazeau 2009; S. P. Davis et al. 2012; Gardiner 1984; Zhu et al. 2013)

Hyoid and gill arches

49. **Gill skeleton mostly beneath otico-occipital region (0); mostly posterior to occipital region (1).** (Lund and Grogan 1997; Stahl 1999; Zangerl 1981) Revised to be consistent with Dearden *et al*. (Dearden et al. 2019).

50. **First branchial arch meets neurocranium ventral to otic region (0); posterior to otic region (1).** Included after Dearden *et al*. (Dearden et al. 2019), they score *Cladoselache* and *Cobelodus* as state '0'; here, the scores are revised to state '1'.

51. **Perforate hyomandibula: absent (0); present (1).** (Lu et al. 2016; Zhu et al. 2009, 2013)

52. **Interhyal: absent (0); present (1).** (S. P. Davis et al. 2012; Zhu et al. 2013)

53. **Ceratohyal with posterior/proximal external fossa: absent (0); present (1).** Fossil examples include *Tristychius* (Coates et al. 2019), *Egertonodus* (Maisey 1983), *Orthacanthus* ((Hotton 1952); pers. obs. M.I.C.), *Gogoselachus* (Long et al. 2015), *Cladoselache* (John G. Maisey 1989), *Ferromirum* (Frey, Coates, et al. 2020) and *Maghriboselache* (Supplementary Fig. 39).

54. **Ceratohyal with broad posteroventral flange or shelf, projecting laterally into recess in Meckel's cartilage: absent (0); present (1).** Present in *Ferromirum* (Frey, Coates, et al. 2020) and *Maghriboselache* (Supplementary Fig. 39), this flange is likely also present in *Cladoselache*, identified as a 'ventral process' (Maisey 1989). In *Maghriboselache* (Supplementary Fig. 39) and *Ferromirum* (Frey, Coates, et al. 2020), the flange fits snugly into a smooth recess within the posteroventral extremity of the medial surface of Meckel's cartilage. Such a flange is also present in *Cobelodus aculeatus* PF 7351 (listed in Zangerl & Case (Zangerl and Case 1976)), and is likely present in many symmoriids.

55. **Ceratohyal spatulate or bladed anteriorly: absent (0); present (1).**

56. **Hypohyals: absent (0); present (1).** (Friedman and Brazeau 2010; Pradel et al. 2014) scores updated after Dearden *et al*. (Dearden et al. 2019). Presence in *Maghriboselache* (Supplementary Fig. 39) and *Ferromirum* (Frey, Coates, et al. 2020) is consistent with conditions *Ozarcus*, *Cobelodus*, and *Akmonistion*.

57. **Basihyal: absent, hyoid arch articulates directly with basibranchial (0); present (1).** (Brazeau et al. 2017; Carr et al. 2009; Dearden et al. 2019; Pradel et al. 2014); Dearden *et al*. (2019) in part.

58. **Separate supra- and infra-pharyngobranchials absent (0); present (1).** (Gardiner 1984; Pradel et al. 2014) Scored as uncertain for *Maghriboselache* (Supplementary Fig. 39) and *Ferromirum* (Frey, Coates, et al. 2020).

59. **Pharyngobranchials directed anteriorly (0); posteriorly (1).** (Pradel et al. 2014) Scored as uncertain for *Maghriboselache* (Supplementary Fig. 39) and *Ferromirum* (Frey, Coates, et al. 2020).

60. **Posteriormost branchial arch bears epibranchial unit: absent (0); present (1).** Scored as uncertain for *Maghriboselache* (Supplementary Fig. 2, 9, 11) and *Ferromirum* (Frey, Coates, et al. 2020).

61. **Epibranchials bear posterior flange: absent (0); present (1).** Scored as uncertain for *Maghriboselache* (Supplementary Fig. 2, 9, 11) and *Ferromirum* (Frey, Coates, et al. 2020).

62. **Hypobranchials directed anteriorly (0); hypobranchials of second and more posterior gill arches directed posteriorly (1).** Scored as uncertain for *Maghriboselache* (Supplementary Fig. 2, 9, 11) and *Ferromirum* (Frey, Coates, et al. 2020).

63. **Multiple unpaired basibranchial mineralisations absent (0); present (1).** Included after Dearden *et al*. (Dearden et al. 2019), but scores differ.

64. **Elongate posterior copula projects posteriorly, beyond rearmost branchial arch: absent (0); present (1).** Present in many early chondrichthyans, including *Maghriboselache* (Supplementary Fig. 2), *Ferromirum* (Frey, Coates, et al. 2020), *Gutturensis, Debeerius,* and *Gladbachus*; possibly an autapomorphy at some level of the chondrichthyan clade.

Dentition & tooth-bearing bones

65. **Oral dermal tubercles borne on jaw cartilages: absent (0); present (1).** (Brazeau 2009; S. P. Davis et al. 2012; Hanke and Wilson 2004; Zhu et al. 2013) This character is concerned only with position and form, and not with histological, and by inference, developmental distinctiveness (Rücklin et al. 2012).

66. **Pharyngeal teeth or denticles: absent (0); present (1).**

67. **Tooth families/generative tooth sets: absent (0); present (1).** (Brazeau 2009; J. C. Davis 2002; S. P. Davis et al. 2012; Hanke and Wilson 2004; Zhu et al. 2013)

68. **Tooth families/generative sets restricted to symphysial region (0); distributed along jaw margin (1).** (Brazeau 2009; S. P. Davis et al. 2012; Hanke and Wilson 2004; Zhu et al. 2013) Revised in light of discussion by Tucker and Fraser (2013).

68. **Symphysial tooth whorl absent (0); present (1).** Present in *Cladoselache* and *Maghriboselache*, absent in *Ferromirum*.

69. **Number of generative tooth sets per jaw ramus: 15 or fewer (0); 20 or more (1).** About 11 in *Maghriboselache* (Supplementary Fig. 42, 43).

70. **Bases of tooth families/ generative sets: single, continuous plate (0); some or all whorls consist of separate tooth units (1).** Adjusted from Brazeau (Brazeau 2009); (S. P. Davis et al. 2012; Giles, Friedman, et al. 2015; Zhu et al. 2013).

71. **Lingual torus: absent (0); present (1).** (Ginter et al. 2010) Present in *Maghriboselache* and *Cladoselache*.

72. **Basolabial shelf: absent (0); present (1).** (Ginter et al. 2010) Present in *Maghriboselache* and *Cladoselache*.

73. **Teeth with three slim main cusps almost equal to each other, strongly recurved: absent (0); present (1).** Adapted from Ginter *et al*. (Ginter et al. 2010).

74. **Toothplates absent (0); present (1).** (Patterson 1965; Stahl 1999)

75. **Toothplates consolidated into one to three large posterior plates, and one to three smaller anterior tooth plates, occupying each quadrant of the jaw: absent (0); present (1).** Adapted from Stahl (Stahl 1999).

76. **Toothplate complement restricted to two pairs in the upper jaw and a single pair in the lower jaw: absent (0); present (1).** After Patterson (Patterson 1965).

77. **Mandibular teeth fused to dermal plates on biting surfaces of jaw cartilages: absent (0); present (1).** (Brazeau 2009; S. P. Davis et al. 2012; Hanke and Wilson 2004; Zhu et al. 2013)

78. **Dermal plates on biting surface of jaw cartilages: absent (0); present (1).** (Brazeau 2009; S. P. Davis et al. 2012; Giles, Friedman, et al. 2015; Zhu et al. 2013)

79. **Gnathal plates mesial to and/or above (or below) jaw cartilage: absent (0); present (1).** (Zhu et al. 2016)

80. **Maxilla and premaxilla *sensu stricto* (upper gnathal plates lateral to jaw cartilage without palatal lamina): absent (0); present (1)**. (Zhu et al. 2016).

81. **Dentary bone encloses mandibular sensory canal: absent (0); present (1).** (Gardiner 1984) see also Zhu *et al.* (Zhu et al. 2009, 2013).

82. **Infradentary foramen and groove, series: absent (0); present (1**). (Zhu et al. 2010)

83. **Tooth-bearing median rostral: absent (0); present (1).** (Zhu et al. 2009, 2013)

84. **Median dermal bone of palate (parasphenoid): absent (0); present (1).** (Brazeau 2009; S. P. Davis et al. 2012; Gardiner 1984; Zhu et al. 2013)

85. **Denticulated field of parasphenoid: without spiracular groove (0); with spiracular groove (1).** (Friedman 2007; Zhu et al. 2009, 2013)

86. **Denticle field of parasphenoid with multifid anterior margin: absent (0); present (1).** (Friedman 2007; Lu et al. 2016; Zhu et al. 2009, 2013)

Mandibular arch

87. **Large otic process of the palatoquadrate: absent (0); present (1).** (Brazeau 2009; Coates and Sequeira 2001; J. C. Davis 2002; Zhu et al. 2009, 2013)

88. **Oblique ridge or groove along medial face of palatoquadrate: absent (0); present (1).** (Brazeau 2009; S. P. Davis et al. 2012; Lu et al. 2016; Zhu et al. 2013) Likely more widespread than previously thought: in *Maghriboselache* and *Ferromirum* the ridge matches the position of that in *Acanthodes*, although the cross-sectional profile is more rounded.

89. **Fenestration of palatoquadrate at basipterygoid articulation: absent (0); present (1).** (Brazeau 2009; S. P. Davis et al. 2012; Lu et al. 2016; Zhu et al. 2013)

90. **Perforate or fenestrate anterodorsal (metapterygoid) portion of palatoquadrate: absent (0); present (1).** (Brazeau 2009; J. C. Davis 2002; S. P. Davis et al. 2012; Zhu et al. 2013)

91. **Articulation surface of the palatoquadrate with the postorbital process directed anteriorly (0); laterally (1); dorsally (2).** Supplementary Fig. 39.

92. **Palatoquadrate fused to the neurocranium: absent (0); present (1).** Not fused in *Maghriboselache*.

93. **Pronounced dorsal process on Meckelian bone or cartilage: absent (0); present (1).** (Brazeau 2009; C. Burrow et al. 2016; S. P. Davis et al. 2012; Hanke and Wilson 2004; Zhu et al. 2013)

94. **Mandibular knob or mesial process: absent (0); present (1).** (Brazeau 2009; C. Burrow et al. 2016; S. P. Davis et al. 2012; Zhu et al. 2013)

95. **Jaw articulation located on rearmost extremity of mandible: absent (0); present (1).** (S. P. Davis et al. 2012; Zhu et al. 2013)

96. **Meckel's cartilage with flange or shelf projecting posteriorly from the lateral cotylus (glenoid): absent (0); present (1).** *Tristychius* (Coates et al. 2019)) and *Gogoselachus* (Long et al. 2015), figs 1C and 2A, G) exhibit the same derived condition in which this retroarticular flange is an uninterrupted, smooth extension of the lateral wall of the mandible. Also present in *Maghriboselache*, but shorter (Supplementary Fig. 39).

97. **Dental trough adjacent to oral rim on Meckel’s cartilage and palatoquadrate: absent (0); present (1).**

98. **Dental trough divided, scalloped tooth-bearing margin on Meckel’s cartilage and palatoquadrate: absent (0); present (1).**

99. **Mandibular symphysis fused: absent (0); present (1).**

Neurocranium

100. **Internasal vacuities: absent (0); present (1).** (Lu et al. 2016)

101. **Precerebral fontanelle: absent or minimal (0); present and large (1).** (Brazeau 2009; Brazeau and Friedman 2014; M. I. Coates and Sequeira 1998, 2001; Michael I. Coates and Sequeira 2001; S. P. Davis et al. 2012; Lund and Grogan 1997; John Graham Maisey 2001; Pradel et al. 2011; Schaeffer 1981; Zhu et al. 2013)

102. **Space for forebrain and (at least) proximal portion of olfactory tracts narrow and elongate, extending between orbits: absent (0); present (1**).

103. **Rostral bar: absent (0); present (1).** Maisey (John Graham Maisey 1985). Absent in *Maghriboselache*.

104. **Internasal groove absent (0); present (1).**

105. **Orbitonasal lamina expanded: absent (0); present (1).** (Patterson 1965) fig. 39.

106. **Elongate, tooth-bearing, pre-nasal ethmo-rostral region: absent (0); present (1).** Absent in *Maghriboselache*.

107. **Palatobasal (or orbital) articulation posterior to the optic foramen (0); anterior to the optic foramen, grooved, and overlapped by process or flange of palatoquadrate (1); anterior to optic foramen, smooth, and overlaps or flanks articular surface on palatoquadrate (2).** (Coates et al. 2017; Maisey 2005; Pradel et al. 2011) P

108. **Trochlear nerve foramen anterior to optic nerve foramen: absent (0); present (1).**

109. **Supraorbital shelf broad with convex lateral margin: absent (0); present (1).** (Brazeau 2009; S. P. Davis et al. 2012; Zhu et al. 2013)

110. **Interorbital space broad (0); narrow (1).** (Brazeau 2009; Coates et al. 2017; S. P. Davis et al. 2012; Zhu et al. 2013)

111. **Optic pedicel: absent (0); present (1).** (Coates et al. 2017; Dupret et al. 2014; Zhu et al. 2009, 2013)

112. **Large prootic foramen separated from optic fenestra by antotic pillar bearing optic pedicel: absent (0); present (1).** Adapted from Maisey et al. (Maisey et al. 2019).

113. **Ophthalmic foramen in anterodorsal extremity of orbit communicates with enclosed cranial space: absent (0); present (1).**

114. **Extended prehypophysial portion of sphenoid: absent (0); present (1).** (Brazeau 2009; S. P. Davis et al. 2012; Zhu et al. 2013)

115. **Canal for efferent pseudobranchial artery within basicranial cartilage: absent (0); present (1).** (Brazeau 2009; S. P. Davis et al. 2012; Zhu et al. 2013)

116. **Entrance of internal carotids: through separate openings flanking the hypophyseal opening or recess (0); through a common opening at the central midline of the basicranium (1).** (Brazeau 2009; Coates and Sequeira 1998; S. P. Davis et al. 2012; Schaeffer 1981; Zhu et al. 2013)

117. **Internal carotids: entering single or paired openings in the basicranium from a posterolateral angle (0); entering basicranial opening(s) head-on from an extreme, lateral angle (1); absent (2).**

118. **Ascending basisphenoid pillar pierced by common internal carotid: absent (0); present (1).** (Brazeau 2009; S. P. Davis et al. 2012; Friedman and Brazeau 2010; Miles 1973b; Zhu et al. 2013)

119. **Spiracular groove on basicranial surface: absent (0); present (1).** (S. P. Davis et al. 2012; Zhu et al. 2013)

120. **Spiracular groove on lateral or transverse wall of jugular canal: absent (0); present (1).** (S. P. Davis et al. 2012; Zhu et al. 2013)

121. **Spiracular groove open (0); enclosed by spiracular bar or canal (1).** (Lu et al. 2016); (Patterson 1982)

122. **Orbit larger than otic capsule: absent (0); present (1)**. (Coates et al. 2017; Lund and Grogan 1997)

123. **Postorbital process and arcade: absent (0); present (1).** (Pradel et al. 2011)

124. **Postorbital process and arcade short and deep - width not more than maximum braincase width (excluding arcade) (0); process and arcade wide - width exceeds maximum width of braincase, and anteroposteriorly narrow (1); process and arcade massive (2); arcade forms postorbital pillar (3).**

125. **Postorbital process downturned, with anhedral angle relative to basicranium: absent (0); present (1).** (Maisey 2011)

126. **Jugular canal diameter small (0); large (1); canal absent (2).** (Pradel et al. 2011)

127. **Canal, likely for trigeminal nerve (V) mandibular ramus, passes through the postorbital process from proximal dorsal entry to distal and ventral exit: absent (0); present (1).**

128. **Postorbital process expanded anteroposteriorly: absent (0); present (1).**

129. **Postorbital process articulates with palatoquadrate: absent (0); present (1).** (Coates and Sequeira 1998; S. P. Davis et al. 2012; Maisey 2001; Pradel et al. 2011; Schaeffer 1981; Zhu et al. 2013, 2013)

130. **Trigemino-facial recess: absent (0); present (1).** (S. P. Davis et al. 2012; Gardiner 1984; Goodrich 1930; Pradel et al. 2010, 2011)

131. **Jugular canal long, extends throughout most of otic capsule wall posterior to the postorbital process (0); short and/or groove present on exterior of otic wall (1); absent, path of jugular removed from otic wall (2**). (Brazeau 2009; Coates et al. 2017; S. P. Davis et al. 2012; Giles, Friedman, et al. 2015; Zhu et al. 2013)

132. **C-bout notch separates postorbital process from supraotic shelf: absent (0); present (1).**

133. **Hyoid ramus of facial nerve (N. VII) exits through posterior jugular opening: absent (0); present (1).** (Brazeau 2009; S. P. Davis et al. 2012; Friedman 2007; Friedman and Brazeau 2010; Zhu et al. 2013)

134. **Periotic process: absent (0); present (1).** (Coates et al. 2017; Maisey 2007)

135. **Relative position of jugular groove and hyomandibular articulation: hyomandibula dorsal or same level (i.e. on bridge) (0); jugular vein passing dorsal or lateral to hyomandibula (1).** (Brazeau and de Winter 2015)

136. **Transverse otic process: absent (0); present (1).** (Giles, Coates, et al. 2015; Lu et al. 2016; Zhu et al. 2016)

137. **Craniospinal process: absent (0); present (1).** (Giles, Friedman, et al. 2015; Zhu et al. 2016)

138. **Lateral otic process: absent (0); present (1).** (Brazeau 2009; Coates and Sequeira 1998; S. P. Davis et al. 2012; Schaeffer 1981; Zhu et al. 2013)

139. **Hyomandibula articulates with neurocranium beneath otic shelf: absent (0); present (1).**

140. **Sub-otic occipital fossa: absent (0); present (1).**

141. **Postotic process: absent (0); present (1)** (Pradel et al. 2011)

142. **Otic capsule extends posterolaterally relative to occipital arch: absent (0); present (1)** (John Graham Maisey 1985)

143. **Otic capsules: widely separated (0); approaching dorsal midline (1).**

144. **Otic capsules project anteriorly between postorbital processes: absent (0); present (1)** (John Graham Maisey 1983)

145. **Endocranial roof anterior to otic capsules domelike, smoothly convex dorsally and anteriorly: absent (0); present (1).**

146. **Roof of skeletal cavity for cerebellum and mesencephalon significantly higher than dorsal-most level of semicircular canals: absent (0); present (1).**

147. **Roof of the endocranial space for telencephalon and olfactory tracts offset ventrally relative to level of mesencephalon: absent (0); present (1).** (Coates et al. 2017)

148. **Labyrinth cavity separated from the main neurocranial cavity by a cartilaginous or ossified capsular wall (0); skeletal medial capsular wall absent (1).** (S. P. Davis et al. 2012; Pradel et al. 2011; Zhu et al. 2013)

149. **Double octaval nerve foramina in chondrified mesial wall of otic capsule: absent (0); present (1).**

150. **External (horizontal) semicircular canal joins the vestibular region dorsal to posterior ampulla (0); joins level with posterior ampulla (1).** (S. P. Davis et al. 2012; Zhu et al. 2013)

151. **Angle of external semicircular canal: in lateral view, straight line projected through canal intersects anterior ampulla, external ampullae, and base of foramen magnum: absent (0); present (1)** (Maisey 2007).

152. **Left and right external semicircular canals approach or meet the posterodorsal midline of the hindbrain roof: absent (0); present (1).**

153. **Preampullary portion of posterior semicircular canal absent (0); present (1).**

154. **Crus commune connecting anterior and posterior semicircular canals: present (0); absent (1).**

155. **Sinus superior: absent or indistinguishable from union of anterior and posterior canals with saccular chamber (0); present, elongate and nearly vertical (1).** (S. P. Davis et al. 2012; Zhu et al. 2013).

156. **Lateral cranial canal: absent (0); present (1).** (Coates 1998; Gardiner 1984; Lu et al. 2016; Zhu et al. 2009, 2013)

157. **Endolymphatic ducts: posteriodorsally angled tubes (0); tubes oriented vertically through endolymphatic fossa/posterior dorsal fontanelle (1).** (Brazeau 2009; M. I. Coates and Sequeira 1998; Coates and Sequeira 2001; J. C. Davis 2002; S. P. Davis et al. 2012; Schaeffer 1981; Zhu et al. 2013)

158. **Posterior dorsal fontanelle connected to persistent otico-occipital fissure (0); posterior tectum separates fontanelle from fissure (1).** (Coates and Sequeira 1998; Pradel et al. 2011; Schaeffer 1981)

159. **Subcircular endolymphatic foramen: absent (0); present (1).** (Maisey and Lane 2010; Pradel et al. 2014)

160. **External opening for endolymphatic ducts anterior to crus commune: absent (0); present (1).** (Coates et al. 2017)

161. **Supraotic shelf broad: absent (0); present (1).**

162. **Dorsal otic ridge: absent (0); present (1).** (Brazeau and Friedman 2014; Coates and Sequeira 1998; Coates and Sequeira 2001; J. C. Davis 2002; S. P. Davis et al. 2012; Maisey 2001; Zhu et al. 2013)

163. **Dorsal otic ridge forms a crest posteriorly: absent (0); present (1).** (Coates and Sequeira 1998; Michael I. Coates and Sequeira 2001; Pradel et al. 2011)

164. **Endolymphatic fossa: absent (0); present (1).** (Pradel et al. 2011)

165. **Endolymphatic fossa elongate (slot-shaped), dividing dorsal otic ridge along midline: absent (0); present (1).** (Coates et al. 2017)

166. **Perilymphatic fenestra within the endolymphatic fossa: absent (0); present (1).** (Coates et al. 2017; Pradel et al. 2011)

167. **Ventral cranial fissure: absent (0); present (1).** (Brazeau 2009; Coates and Sequeira 2001; J. C. Davis 2002; S. P. Davis et al. 2012; Janvier 2002; Maisey 2001; Pradel et al. 2011; Zhu et al. 2013)

168. **Endoskeletal intracranial joint: absent (0); present (1).** (S. P. Davis et al. 2012; Janvier 2002; Zhu et al. 2013) Janvier (1996, and references therein).

169. **Metotic (otic-occipital) fissure: absent (0); present (1).** (Brazeau 2009; Coates 1998; J. C. Davis 2002; S. P. Davis et al. 2012; Janvier 2002; Maisey 2001; Pradel et al. 2011; Schaeffer 1981; Zhu et al. 2013)

170. **Vestibular fontanelle: absent (0); present (1).** (Brazeau 2009; S. P. Davis et al. 2012; Friedman and Brazeau 2010; Zhu et al. 2013)

171. **Hypotic lamina: absent (0); present (1).** (Brazeau 2009; Maisey 1984; Maisey 2001; Pradel et al. 2011, 2013; Schaeffer 1981; Zhu et al. 2013) Note recent discussions in Coates *et al*. (Coates et al. 2017) and Maisey *et al*. (Maisey et al. 2019).

172. **Glossopharyngeal nerve path: directed laterally, across floor of the saccular chamber and exits via foramen in side wall of the otic capsule (0); directed posteriorly, and exits through metotic fissure or foramen in posteroventral wall of otic capsule (1); exits laterally through a canal contained ventrally (floored) by the hypotic lamina (2); exits through a foramen anterior to the posterior ampulla (3).** (Brazeau 2009; Coates and Sequeira 1998, 2001; Coates et al. 2017; S. P. Davis et al. 2012; Pradel et al. 2011, 2013; Schaeffer 1981; Zhu et al. 2013)

173. **Glossopharyngeal and vagus nerves share common exit from neurocranium: absent (0); present (1).**

174. **Basicranial morphology: platybasic (0); tropibasic (1).** (Brazeau 2009; S. P. Davis et al. 2012; Maisey 2007; Pradel et al. 2011, 2011; Zhu et al. 2013)

175. **Channel for dorsal aorta and/ or lateral dorsal aortae passes through basicranium (0): external to basicranium (1).** (Brazeau 2009; Brazeau and Friedman 2014; M. I. Coates and Sequeira 1998; Coates et al. 2017; Pradel et al. 2011; Schaeffer 1981)

176. **Dorsal aorta divides into lateral dorsal aortae posterior to occipital level (0); anterior to level of the occiput (1).** (Coates et al. 2017; Giles, Friedman, et al. 2015; Pradel et al. 2011)

177. **Posterior openings of lateral aortic canals positioned lateral to occipital cotylus: absent (0); present (1).** Adapted from Maisey et al. (Maisey et al. 2019).

178. **Ventral portion of occipital arch wedged between rear of otic capsules: absent (0); present (1).** (Brazeau 2009; Coates and Sequeira 1998; Coates et al. 2017; S. P. Davis et al. 2012; Maisey 2001; Pradel et al. 2011; Schaeffer 1981)

179. **Dorsal portion of occipital arch wedged between otic capsules: absent (0); present (1).** (Brazeau 2009; Coates and Sequeira 1998; Coates et al. 2017; S. P. Davis et al. 2012; John Graham Maisey 2001; Pradel et al. 2011; Schaeffer 1981)

180. **Occipital crest anteroposteriorly elongate, and extends from the roof of the posterior tectum: absent (0); present (1).**

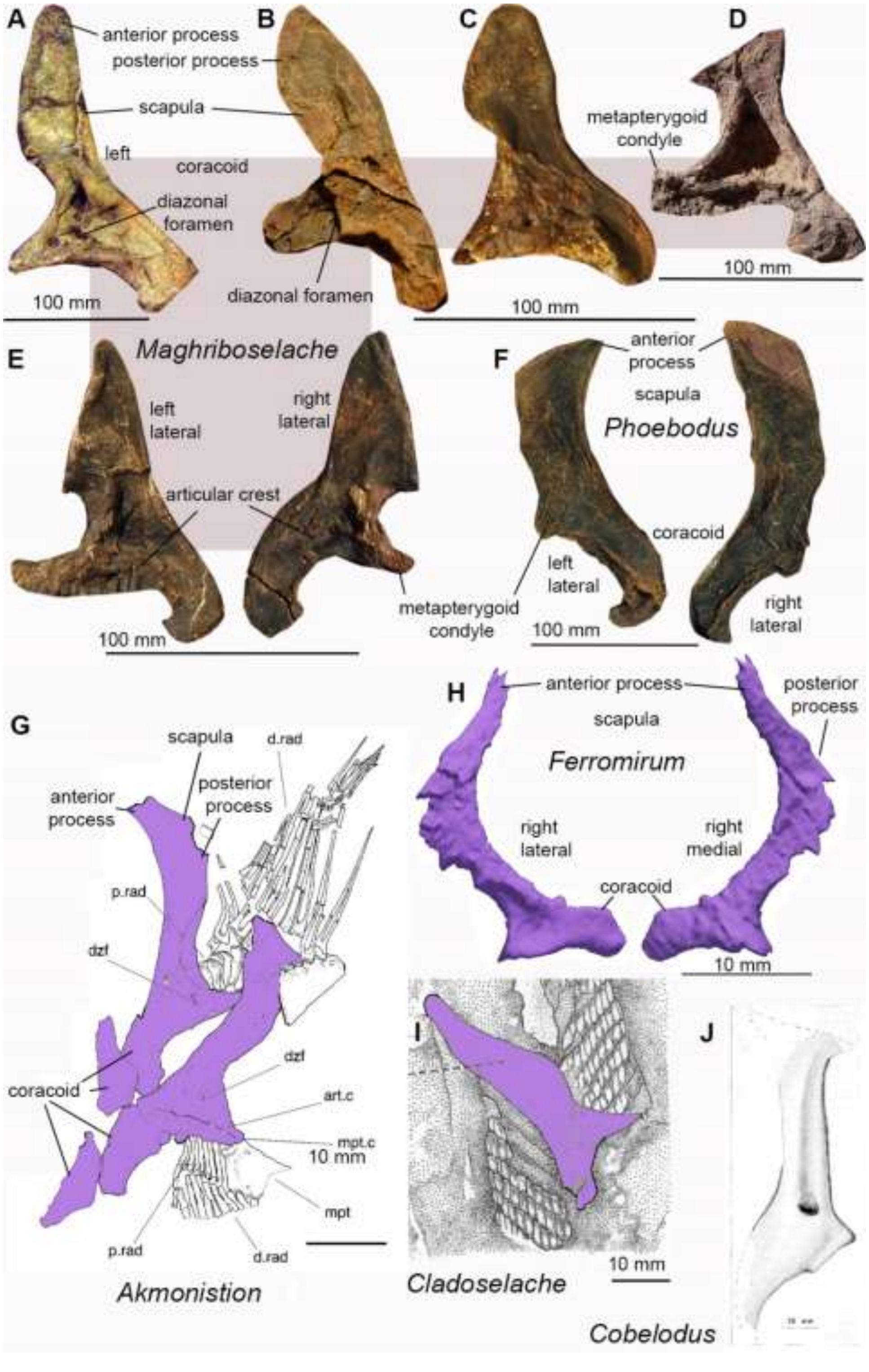

Supplementary Fig. 48 Scapulocoracoids of *Maghriboselache* and other Palaeozoic chondrichthyans. **A** PIMUZ A/I 5152. **B**, **C** AA.MEM.DS.12. **E**, **F** unpublished, ongoing research. **G** Coates & Sequeira (2001). **H** Frey et al. (Frey, Coates, et al. 2020). **I** Dean (B. Dean 1909). **J** Zangerl & Case (Zangerl and Case 1976).

Axial and appendicular skeleton

181. Calcified vertebral centra: absent (0); present (1). (Coates et al. 2017; Maisey 1985)

182. Chordacentra: absent (0); present (1). (Coates et al. 2017; Coates and Sequeira 2001; Stahl 1999) Stahl (1999)

183. Chordacentra polyspondylous and consist of narrow closely packed rings: absent (0); present (1). (Coates et al. 2017; Patterson 1965)

184. Synarcual: absent (0); present (1). (Brazeau 2009; Coates et al. 2017; S. P. Davis et al. 2012; Stahl 1999; Zhu et al. 2013)

185. Macromeric dermal pectoral girdle (0); micromeric or lacking dermal skeleton entirely (1). (Brazeau 2009; S. P. Davis et al. 2012; Zhu et al. 2013)

186. Macromeric dermal pectoral girdle composition: ventral and dorsal components (0); ventral components only (1). (Brazeau 2009; S. P. Davis et al. 2012; Zhu et al. 2013)

187. Macromeric pectoral dermal skeleton forms complete ring around the trunk: present (0); absent (1). (Brazeau 2009; S. P. Davis et al. 2012; Goujet and Young 2004; Zhu et al. 2013)

188. Median dorsal plate: absent (0); present (1). (Brazeau 2009; S. P. Davis et al. 2012; Zhu et al. 2013)

189. Scapular process (dorsal) of shoulder endoskeleton: absent (0); present (1). (Brazeau 2009; Brazeau and Friedman 2014; Coates and Sequeira 2001; J. C. Davis 2002; S. P. Davis et al. 2012; Zhu et al. 2013; Zhu and Schultze 2001)

190. Ventral margin of separate scapular ossification: horizontal (0); deeply angled (1). (Brazeau 2009; S. P. Davis et al. 2012; Hanke and Wilson 2004; Zhu et al. 2013)

191. Cross sectional shape of scapular process: flattened or strongly ovate (0); subcircular (1). (Brazeau 2009; C. Burrow et al. 2016; J. C. Davis 2002; S. P. Davis et al. 2012; Zhu et al. 2013)

192. **Flange on trailing edge of scapulocoracoid: absent (0); present (1).** (Brazeau 2009; Burrow et al. 2016; J. C. Davis 2002; S. P. Davis et al. 2012; Zhu et al. 2013) Davis (2002); Brazeau (2009); Davis *et al.* (2012); Zhu *et al.* (2013); Burrow *et al.* (2016).

193. **Scapular process with posterodorsal process. Absent (0); present (1).** (Coates and Sequeira 2001; S. P. Davis et al. 2012; Zhu et al. 2013)

194. **Mineralisation of internal surface of scapular process: mineralised all around (0); un-mineralised on internal face forming a hemicylindrical cross-section.** (Brazeau 2009; C. Burrow et al. 2016; Hanke and Davis 2012; Zhu et al. 2013)

195. **Coracoid process: absent (0); present (1).** (Brazeau 2009; S. P. Davis et al. 2012; Zhu et al. 2013)

196. **Procoracoid mineralisation: absent (0); present (1).** (Brazeau 2009; J. C. Davis 2002; Hanke and Wilson 2004)

197. **Fin base articulation on scapulocoracoid: stenobasal, deeper than wide (0); eurybasal, wider than deep (1).** (Lu et al. 2016)

198. **Pectoral fin articulation monobasal (0); dibasal (1); three or more basals (2).**

199. **Metapterygium pectinate subtriangular plate or bar supporting numerous (six or more) radials along distal edge: absent (0); present (1).**

200. **Metapterygial whip absent (0); present (1).** (Coates et al. 2017)

201. **Biserial pectoral fin endoskeleton: absent (0); present (1).** (Lu et al. 2016)

202. **Propterygium perforated: absent (0); present (1).** (S. P. Davis et al. 2012; Patterson 1982; Rosen et al. n.d.; Zhu et al. 2013)

203. **Pelvic girdle with fused puboischiadic bar: absent (0); present (1).** (Coates et al. 2017; Coates and Sequeira 2001; John G. Maisey 1984)

204. **Mixipterygial/mixopterygial claspers: absent (0), present (1).** (Brazeau and Friedman 2014; Coates and Sequeira 2001; Coates and Sequeira 2001; Compagno 1999; Liem and Summers 1999; Long et al. 2015; Trinajstic et al. 2015)

205. **Pre-pelvic clasper or tenaculum: absent (0); present (1).** (Coates et al. 2017; Patterson 1965)

206. **Number of dorsal fins, if present: one (0); two (1); one, extending from pectoral to anal fin level (2).** (Brazeau 2009; Coates and Sequeira 2001; S. P. Davis et al. 2012; Zhu et al. 2013) This is complicated by the fact that this can differ between sexes. The presence of an anterior dorsal fin spine is considered as indication of the presence of a fin. Sexual dimorphism in the number of dorsal fins occurs in *Falcatus*, possibly in *Cobelodus*, *Maghriboselache* and in *Cladoselache*. These are coded as state (1) here.

207. **Brush complex of bilaterally distributed calcified tubes flanking or embedded in calcified cartilage core: absent (0); present (1).**

208. **Posterior or pelvic-level dorsal fin with calcified base plate: absent (0); present (1).** (Coates and Sequeira 2001; Coates and Sequeira 2001)

209. **Posterior dorsal fin with delta-shaped cartilage: absent (0); present (1).** (Coates and Sequeira 2001; Coates and Sequeira 2001)

210. **Posterior or pelvic-level dorsal fin shape, base approximately as broad as tall and not broader than other median fins (0); base much longer than fin height, substantially longer than other median fins (1).** (Brazeau and de Winter 2015; Lu et al. 2017)

211. **Anal fin: absent (0); present (1).** (Brazeau 2009; Coates and Sequeira 2001; S. P. Davis et al. 2012; Zhu et al. 2013)

212. **Anal fin base narrow, posteriormost proximal segments radials broad: absent (0); present (1).**

213. **Caudal radials restricted to axial lobe (0); extend beyond level of body wall and deep into hypochordal lobe (1).** (S. P. Davis et al. 2012; Zhu et al. 2013)

214. **Caudal neural and/or supraneural spines or radials short (0); long, expanded, and supporting high aspect-ratio (lunate) tail with notochord extending to posterodorsal extremity (1); notochord terminates pre-caudal extremity, neural and heamal radial lengths near symmetrical and support epichordal and hypochordal lobes respectively (2).**

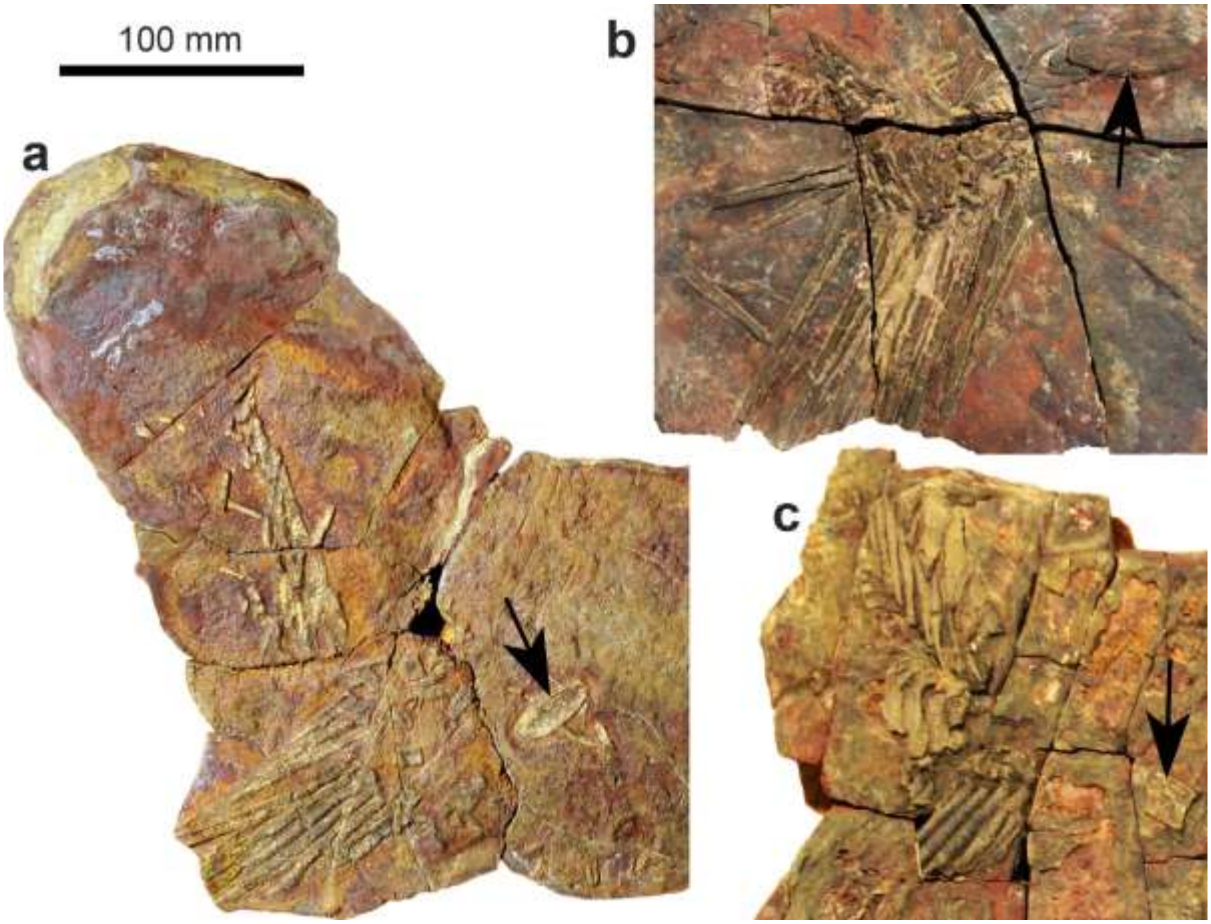

Supplementary Fig. 49 Oval cartilaginous plates at the base of the caudal fin of *Maghriboselache*. **a** AA.BER.DS.01; **b** AA.MEM.DS.12; **c** AA.MEM.DS.6.

Spines: fins, cranial and elsewhere

215. **Dorsal fin spine or spines: absent (0); present (1).** (Brazeau 2009; S. P. Davis et al. 2012; Friedman 2007; Lu et al. 2016; Zhu et al. 2013; Zhu and Yu 2002)

216. **Dorsal fin spine at anterior (pectoral level) location: absent (0); present in males and females (1); present only in one sex/ sexually dimorphic (2).** This was adapted to the insight that only about 50% of the skeletons of *Maghriboselache* preserve this taphonomically resistant structure. We assume the same condition in *Cladoselache* and it has been demonstrated for *Falcatus* (Lund 1985). It is questionable for, e.g., *Damocles* (Lund 1986) and *Cobelodus*.

217. **Anterior dorsal fin spine cross section: horseshoe shaped (0); flat sided with rectangular profile (1); subcircular (2).** (Brazeau and de Winter 2015; Hampe 2002)

218. **Anterior dorsal fin spine curved posteriorly near the apex mainly: absent (0); present (1).** Present in *Maghriboselache*, *Ferromirum* and *Cladoselache*. In *Dracopristis*, the entire fin spine is curved.

219. **Anterior dorsal fin spine leading edge concave in lateral view: absent (0); present (1).**

220. **Anal fin spine: absent (0); present (1).** (Brazeau 2009; J. C. Davis 2002; Maisey 1984) Maisey (1986)

221. **Pectoral fin spines: absent (0); present (1).** (Brazeau 2009; J. C. Davis 2002; Hanke and Davis 2012; Zhu et al. 2013)

222. **Pectoral fin spine with denticles along posterior surface: absent (0); present (1).** (Burrow et al. 2016)

223. **Prepectoral fin spines: absent (0); present (1).** (Brazeau 2009; J. C. Davis 2002; Hanke and Davis 2012; Hanke and Wilson 2004; Zhu et al. 2013) Present in *Doliodus* (Maisey *et a*l. 2017); see also Dearden *et al*. (2019).

224. **Admedian pectoral spines absent (0); present (1).** (Burrow et al. 2016)

225. **Median fin spine insertion: shallow, not greatly deeper than dermal bones/ scales (0); deep (1).** (Brazeau 2009; J. C. Davis 2002; Hanke and Davis 2012; Hanke and Wilson 2004; Zhu et al. 2013)

226. **Intermediate (pre-pelvic) fin spines: absent (0); present (1).** (Brazeau 2009; J. C. Davis 2002; Hanke and Davis 2012; Hanke and Wilson 2004; Zhu et al. 2013)

227. **Fin spines with ridges: absent (0); present (1).** (Brazeau 2009; J. C. Davis 2002; Hanke and Davis 2012; Zhu et al. 2013)

228. **Fin spines with nodes: absent (0); present (1).** (Brazeau 2009; J. C. Davis 2002; Hanke and Davis 2012; Hanke and Wilson 2004; Zhu et al. 2013)

229. **Fin spines (dorsal) with rows of large denticles: absent (0); on posterior surface (1); on lateral surface (2).** (Brazeau 2009; Brazeau and Friedman 2014; Maisey 1989; Zhu et al. 2013)

230. **Cephalic spines: absent (0); present (1).** (Coates et al. 2017; Maisey 1989)

New characters

Scales

231. **Body scale shape: polygonal with similar length and width (0), at least 50% broader than long (1).**
New character. In *Maghriboselache* and *Cladoselache*, most scales have a polygonal outline with subequal length and width. In *Phoebodus*, most body scales are at least twice as wide (lateral direction, transversely) as long (sagitally; own, unpublished observation).

232. **Differentiation of pectoral fin scales: Scale size reduced slightly towards distal tip (0); Scale size reduced significantly towards distal tip (1).**
New character: In *Maghriboselache* and *Cladoselache*, the fin scale diameter becomes larger by about 50% from the tip (0.05 mm) to the base of the pectoral fin (0.1 mm). In *Phoebodus*, the pectoral fin scales range from a width of 0.11 mm at the tip to about 0.45 at the proximal anterior edge of the fin (own, unpublished observation).

Axial skeleton

233. **Cartilaginous plate dorsal of the neural arches: absent (0); present (1).** New character, see Supplementary Fig. 51. Also present in *Cladoselache* (B. Dean 1909), *Akmonistion* (Coates and Sequeira 2001).

Body and head proportions

Here, we introduce five new characters, which are based on proportions between dimensions from the head, pectoral fins and the body. These are illustrated in Supplementary Figure 52.

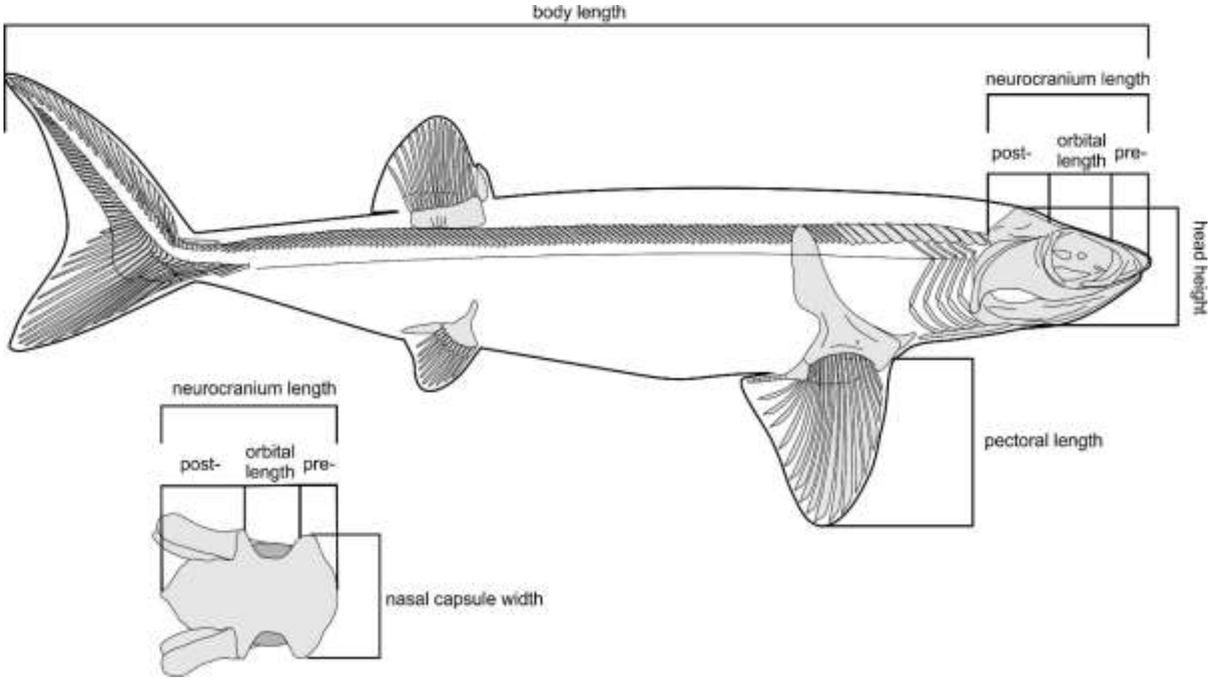

Supplementary Fig. 50: Measurements used for characters 234 to 238.

234. Body form: short and stout (2), intermediate (1) or long and slender (0): Based on the ratio height of the head to length of the body from snout to tip of caudal fin (Supplementary Fig. 51). Is the ratio below 0.1, it is coded as 0, equal to or above 1.5 as 2, and in between as 1.

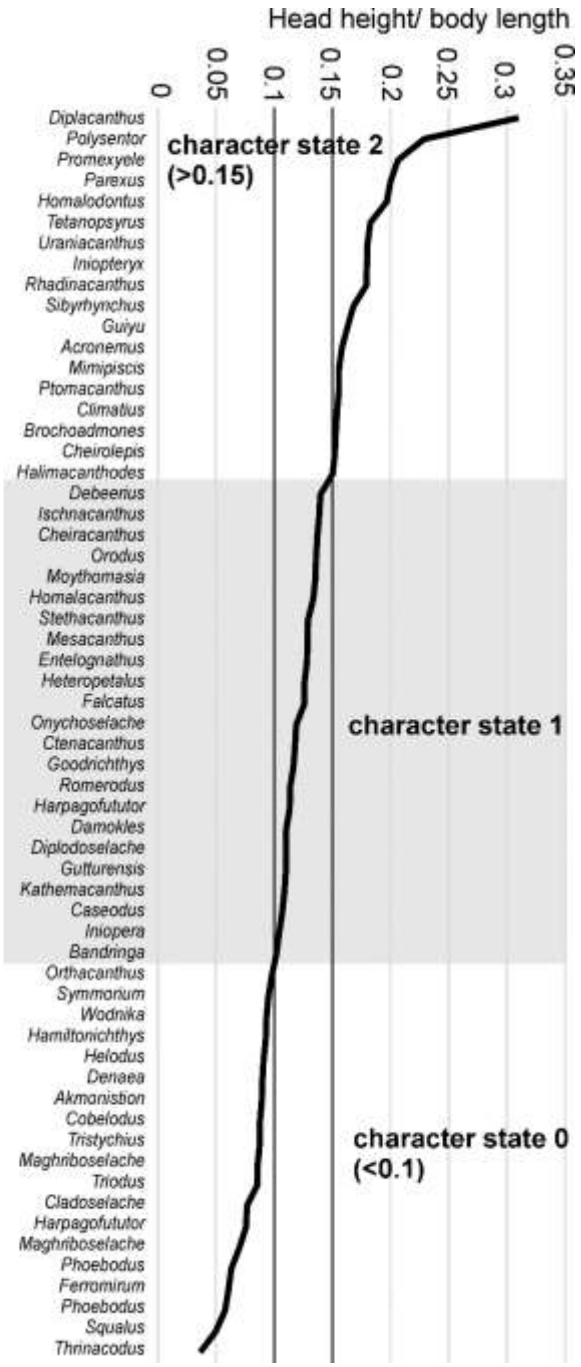

Supplementary Fig. 51: Ratios of head height to body length of some chondrichthyan genera with their character coding.

235. Relative dimensions of pectoral fins: short (0) or long (1): Based on the ratio length of the pectoral fins from their base at the scapulocoracoid to the distal tip to the length of the body from snout to tip of caudal fin (Supplementary Fig. 52). Is the ratio below 0.15, it is coded as 0, equal to or above 1.5 it is coded as 1.

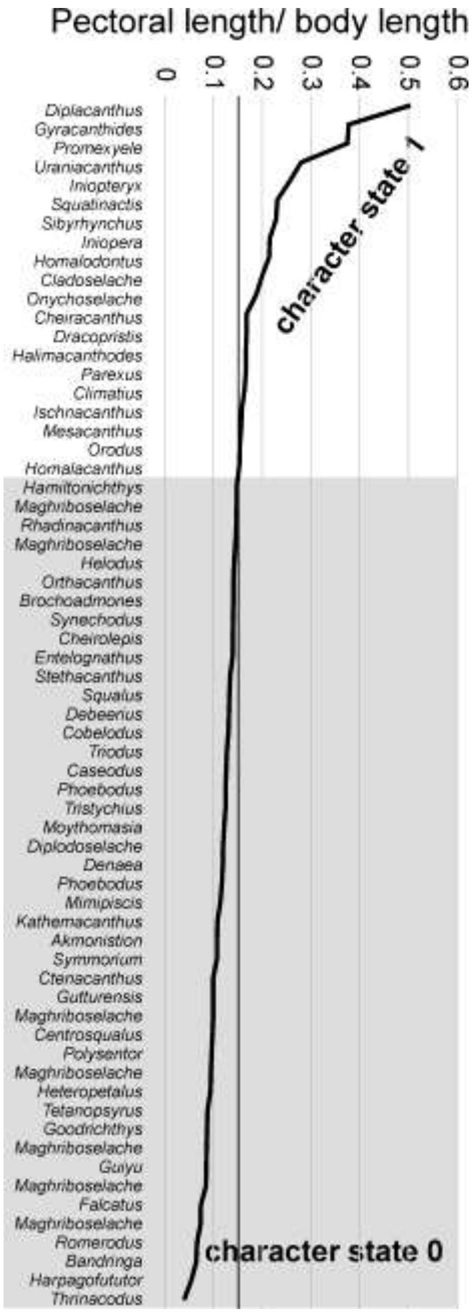

Supplementary Fig. 52: Ratios of pectoral fin length to body length of some chondrichthyan genera with their character coding.

236. **Orbit length relative to neurocranium length: small (0) or large (1):** Based on the ratio sagittal length of the orbit to the length of the neurocranium (Supplementary Fig. 53). Is the ratio below 0.3, it is coded as 0, equal to or above 0.3 it is coded as 1. As shown by Coates et al. (2017), holocephalans and many of their stem group-representatives have comparatively large orbits.

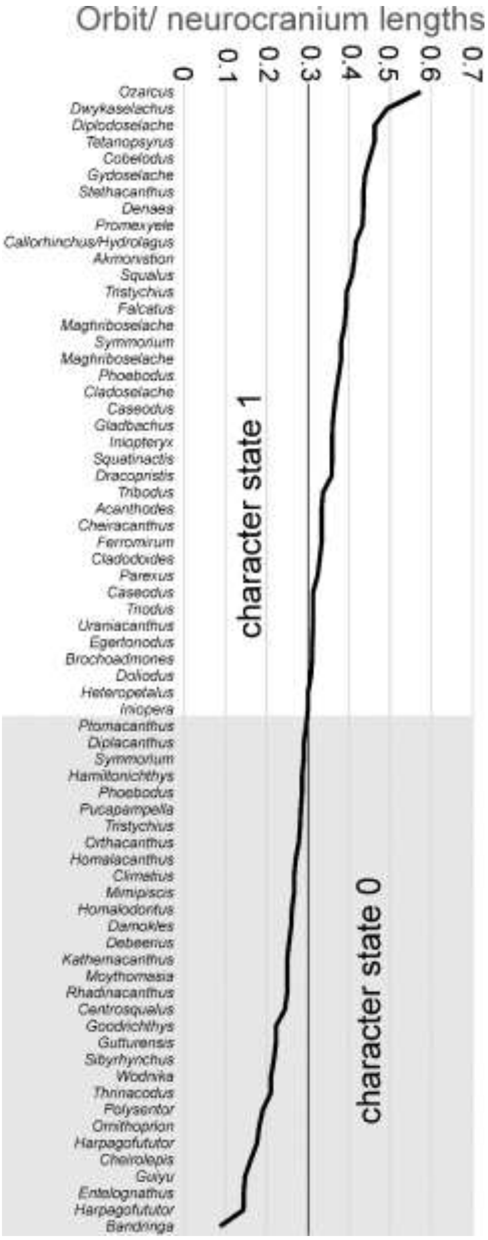

Supplementary Fig. 53: Ratios of the length of the orbit to that of the neurocranium in some chondrichthyan genera with their character coding.

237. Width of olfactory capsule relative to neurocranium length: narrow (0) or wide (1): Based on the ratio between the width of the nasal capsules to the length of the neurocranium (Supplementary Fig. 54). Is the ratio below 0.3, it is coded as 0, equal to or above 0.3 it is coded as 1. Unfortunately, the frontal part including the olfactory capsules is often incompletely preserved and the character state remains uncertain in many taxa.

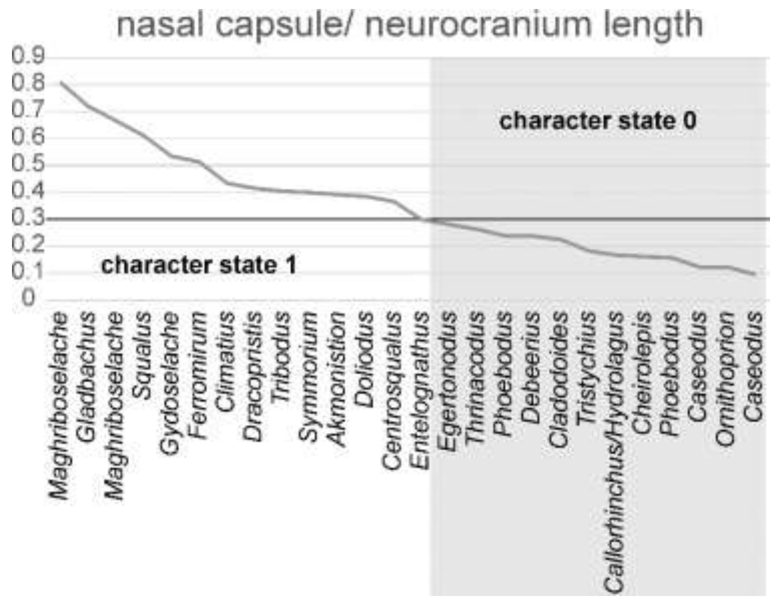

Supplementary Fig. 54: Ratios of the width of the olfactory capsule to the length of the neurocranium of some chondrichthyan genera with their character coding.

238. Postorbital/ preorbital length or position of the orbit within the neurocranium: long ethmoid/ nasal with short otic/ occipital region (0) or short ethmoid/ nasal with long otic/ occipital region (1): Based on the ratio between the lengths of the preorbital part (front of orbit to front of neurocranium/ olfactory capsules) and the postorpbital part (back of orbit to posterior end of neurocranium; Supplementary Fig. 55). Is the ratio below 0.3, it is coded as 0, equal to or above 0.3 it is coded as 1.

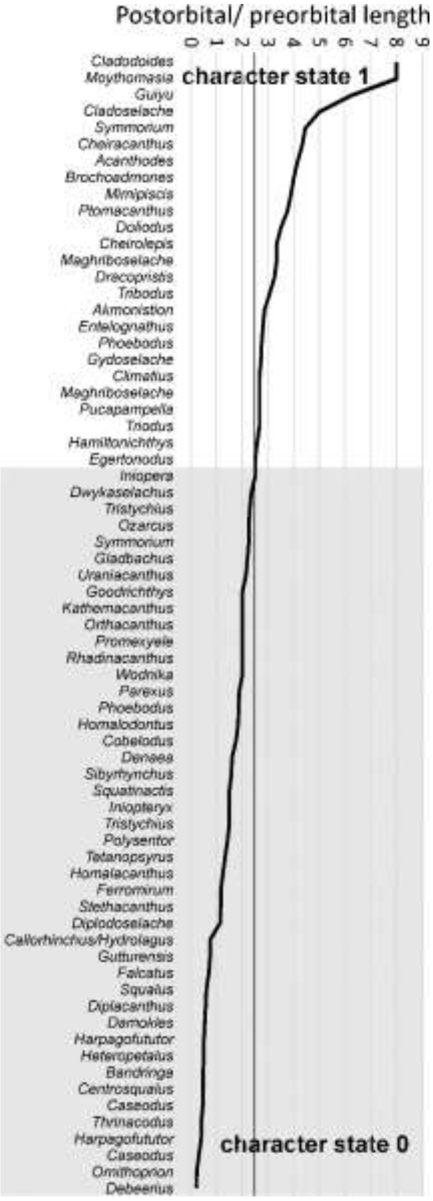

Supplementary Fig. 55 Ratios of the length of the region from the posterior end of the neurocranium to the rear end of the orbit to the region from the anterior tip of the neurocranium to the anterior edge of the orbit of some chondrichthyan genera with their character coding.

Additional tooth characters adopted from Hodnett et al. (Hodnett et al. 2021)

The character number in the original publication is provided in brackets [ ].

239. [50] Anterior extragnathic dentition, (0) absent, (1) ethmoid/ between palatoquadrates, (2) rostral, (3) bony premaxilla.

240. [51] Tooth/ jaw association, (0) absent, (1) individual tooth wells per family, both jaws, (2) all tooth families in single sulcus along jaws, (3) individual tooth wells per family, upper jaw only, (4) teeth follow jaw margin.

241. [52] In line cusp relationship, (0) absent, (1) unicuspid, (2) parallel, (3) highly divergent from base, (4) divergent twisted.

242. [53] Relative cusp sizes, (0) absent, (1) unicuspid, (2) all cusps equal, (3) laterals largest/central small or absent, (4) central largest/others lower.

243. [54] Cusp alignment relative to jaw axis, (0) absent, (1) parallel, (2) lingual concave, (3) twisted contorted alignment, (4) other.

244. [55] Cusp condition, (0) absent, (1) unicuspid, (2) multiple in-line, (3) descending from central, (4) cusps reduced/suppressed, (5) ridge descending from central.

245. [56] Cusp cross section, (0) absent, (1) rounded, (2) reduced/ suppressed, (3) compressed blade-like.

246. [57] Cusp numbers, (0) absent, (1) unicuspid, (2) bicuspid, (3) tricuspid, (4) >3, (5) variable.

247. [58] Crown/root height, (0) absent, (1) equal/subequal, (2) very high crowned, (3) brachydont, (4) variable.

248. [59] Enamel cusp separation, (0) absent, (1) distinctly separate on base, (2) cusps confluent, (3) cusps reduced/suppressed.

249 [60] Functional jaw tooth families, (0) absent, (1) 1-2 per family, (2) pavement occlusion, (3) teeth & tooth plates, (4) tooth plates only.

250. [61] Tooth shapes on jaw, (0) absent, (1) homodont, (2) monognathic heterodonty, (3) dignathic heterodonty, (4) teeth and tooth plates, (5) plates alone.

251. [62] Lower symphysial family, (0) absent, (1) generalized, (2) prominent, (3) whorl, (4) fused plate, (5) paired whorl, (6) multiple families

252. [63] Upper parasymphysial family, (0) absent, (1) generalized, (2) prominent, (3) whorl, (4) fused plate.

253. [64] Crown base, (0) absent, (1) generalized, (2) lingual heel, (3) lingual, labial ridges, (4) basin and ridges.

254. [65] Crown ling1ual/labial buttresses, (0) absent, (1) crenellated, (2) buttressed.

255. [66] Tooth root vascular pattern, (0) absent, (1) few nutrient foramina aborally and lingually, (2) multiple nutrient foramina labiolingually and aborally, (3) few nutriend foramina labiolingually, (4) apical

256. [67] Tooth root length, (0) absent, (1) short below crown, (2) long below crown, (3) extended lingual, (4) fused in family.

257. [68] Root direction, (0) absent, (1) straight below and wide as crown, (2) straight below and narrow than crown, (3) aboral/ lingual s-shape, (4) lingual shelf, (5) proximo-distally arched, (6) separated by neck, contorted.

258. [69] Basolabial/orolingual root projection widths, (0) absent, (1) ridges narrower than primary cusp(s), (2) ridges wider than primary cusp(s).

259. [70] Basolabial root projection structure, (0) absent, (1) basolabial ridge/peg single, (2) basolabial ridge/peg divided.

260. [71] Orolingual root projection structure, (0) absent, (1) orolingual ridge single, (2) orolingual ridge divided.

261. [72] Basolabial depression, (0) absent, (1) shallow, (2) moderate, (3) deep.

262. [73] Intermediate cusps structure, (0) absent. (1) shorter than lateral cusps, (2) taller than lateral cusps.

263. [74] Intermediate cusp number, (0) absent, (1) 1 to 2, (2) greater than 2.

264. [75] Lateral cusps, (0) absent, (1) taller than median, (2) shorter than median.

265. [76] Accessory labial cusps, (0) absent, (1) present, (2) variable.

Supplementary references

Arratia, G., & Cloutier, R. (1996). Reassessment of the morphology of *Cheirolepis canadensis* (Actinopterygii). In *Devonian Fishes and Plants of Miguasha, Quebec, Canada* (pp. 165–197).

Barido-Sottani, J., Aguirre-Fernández, G., Hopkins, M. J., Stadler, T., & Warnock, R. (2019). Ignoring stratigraphic age uncertainty leads to erroneous estimates of species divergence times under the fossilized birth–death process. *Proceedings of the Royal Society B: Biological Sciences*, *286*(1902), 20190685. https://doi.org/10.1098/rspb.2019.0685

Bendix-Almgreen, S. E. (1975). The paired fins and shoulder girdle in Cladoselache, their morphology and phyletic significance. *Colloques Internationaux Centre National de la Recherche Scientifique*, (218), 111–123.

Bernacsek, C. M., & Dineley, C. L. (1977). New acanthodians from the Delorme Formation (Lower Devonian) of N.W.T., Canada. *Palaeontographica. Abteilung A, Paläozoologie, Stratigraphie*, *158*, 1–25.

Beznosov, P. (2009). A redescription of the Early Carboniferous acanthodian *Acanthodes lopatini* Rohon, 1889. *Acta Zoologica*, *90*, 183–193. https://doi.org/10.1111/j.1463-6395.2008.00352.x

Bouckaert, R., Vaughan, T. G., Barido-Sottani, J., Duchêne, S., Fourment, M., Gavryushkina, A., et al. (2019). BEAST 2.5: An advanced software platform for Bayesian evolutionary analysis. *PLOS Computational Biology*, *15*(4), e1006650. https://doi.org/10.1371/journal.pcbi.1006650

Brazeau, M. D. (2009). The braincase and jaws of a Devonian 'acanthodian' and modern gnathostome origins. *Nature*, *457*(7227), 305–308. https://doi.org/10.1038/nature07436

Brazeau, M. D. (2012). A revision of the anatomy of the Early Devonian jawed vertebrate *Ptomacanthus anglicus* Miles: ANATOMY OF PTOMACANTHUS. *Palaeontology*, *55*(2), 355–367. https://doi.org/10.1111/j.1475-4983.2012.01130.x

Brazeau, M. D., & de Winter, V. (2015). The hyoid arch and braincase anatomy of *Acanthodes* support chondrichthyan affinity of 'acanthodians.' *Proceedings of the Royal Society B: Biological Sciences*, *282*(1821), 20152210. https://doi.org/10.1098/rspb.2015.2210

Brazeau, M. D., & Friedman, M. (2014). The characters of Palaeozoic jawed vertebrates: Early Gnathostome Characters. *Zoological Journal of the Linnean Society*, *170*(4), 779–821. https://doi.org/10.1111/zoj.12111

Brazeau, M. D., Friedman, M., Jerve, A., & Atwood, R. C. (2017). A three-dimensional placoderm (stem-group gnathostome) pharyngeal skeleton and its implications for primitive gnathostome pharyngeal architecture. *Journal of Morphology*, *278*(9), 1220–1228. https://doi.org/10.1002/jmor.20706

Burrow, C., den Blaauwen, J., Newman, M., & Davidson, R. (2016). The diplacanthid fishes (Acanthodii, Diplacanthiformes, Diplacanthidae) from the Middle Devonian of Scotlan. *Palaeontologia Electronica*. https://doi.org/10.26879/601

Burrow, C. J., Trinajstic, K., & Long, J. (2012). First acanthodian from the Upper Devonian (Frasnian) Gogo Formation, Western Australia. *Historical Biology*, *24*(4), 349–357. https://doi.org/10.1080/08912963.2012.660150

Burrow, C. J., & Turner, S. (2013). Scale structure of putative chondrichthyan *Gladbachus adentatus* Heidtke & Krätschmer, 2001 from the Middle Devonian Rheinisches Schiefergebirge, Germany. *Historical Biology*, *25*(3), 385–390. https://doi.org/10.1080/08912963.2012.722761

Carr, R. K., Johanson, Z., & Ritchie, A. (2009). The phyllolepid placoderm *Cowralepis mclachlani* : Insights into the evolution of feeding mechanisms in jawed vertebrates. *Journal of Morphology*, *270*(7), 775–804. https://doi.org/10.1002/jmor.10719

Chang, M. M. (1982). *The braincase of Youngolepis, a Lower Devonian crossopterygian from Yunnan, south-western China*. Stockholm University, Stockholm.

Chang, M. M. (1991). Head exoskeleton and shoulder girdle of *Youngolepis*. In *Early Vertebrates and Related Problems of Evolutionary Biology* (pp. 355–378).

Chang, M. M. (2004). Synapomorphies and scenarios - more characters of *Youngolepis* betraying its affinity to the Dipnoi. In *Recent Advances in the Origin and Early Radiation of Vertebrates* (pp. 665–686). Verlag Dr. Friedrich Pfeil.

Chang, M. M., & Yu, X. (1981). A new crossopterygian, *Youngolepis praecursor*, gen. et sp. nov., from Lower Devonian of E. Yunnan, China, *24*, 89–97.

Choo, B. (2012). Revision of the actinopterygian genus *Mimipiscis* (= *Mimia*) from the Upper Devonian Gogo Formation of Western Australia and the interrelationships of the early Actinopterygii. *Earth and Environmental Science Transactions of the Royal Society of Edinburgh*, *102*(2), 77–104. https://doi.org/10.1017/S1755691011011029

Coates, M. I. (1998). Actinopterygians from the Namurian of Bearsden, Scotland, with comments on early actinopterygian neurocrania. *Zoological Journal of the Linnean Society*, *122*(1–2), 27–59. https://doi.org/10.1111/j.1096-3642.1998.tb02524.x

Coates, M. I., & Sequeira, S. E. K. (1998). The braincase of a primitive shark. *Transactions of the Royal Society of Edinburgh: Earth Sciences*, *89*(2), 63–85. https://doi.org/10.1017/S026359330000701X

Coates, M. I., & Sequeira, S. E. K. (2001). A new stethacanthid chondrichthyan from the lower Carboniferous of Bearsden, Scotland. *Journal of Vertebrate Paleontology*, *21*(3), 438–459. https://doi.org/10.1671/0272-4634(2001)021[0438:ANSCFT]2.0.CO;2

Coates, Michael I. (1994). The origin of vertebrate limbs. *Development*, (Supplement), 169–180.

Coates, Michael I. (1999). Endocranial preservation of a Carboniferous actinopterygian from Lancashire, UK, and the interrelationships of primitive actinopterygians. *Philosophical Transactions of the Royal Society of London. Series B: Biological Sciences*, *354*(1382), 435–462. https://doi.org/10.1098/rstb.1999.0396

Coates, Michael I. (2005). *Gladbachus adentatus* Heidtke & Kratschmer; is it a chondrichthyan, and if so, where does it fit in phylogeny? *Paleobios*, *25*, 31–32.

Coates, Michael I., Finarelli, J. A., Sansom, I. J., Andreev, P. S., Criswell, K. E., Tietjen, K., et al. (2018). An early chondrichthyan and the evolutionary assembly of a shark body plan. *Proceedings of the Royal Society B: Biological Sciences*, *285*(1870), 20172418. https://doi.org/10.1098/rspb.2017.2418

Coates, Michael I., & Gess, R. W. (2007). A new reconstruction of *Onychoselache traquairi*, comments on early chondrichthyan pectoral girdles and hybodontiform phylogeny. *Palaeontology*, *50*(6), 1421–1446. https://doi.org/10.1111/j.1475-4983.2007.00719.x

Coates, Michael I., Gess, R. W., Finarelli, J. A., Criswell, K. E., & Tietjen, K. (2017). A symmoriiform chondrichthyan braincase and the origin of chimaeroid fishes. *Nature*, *541*(7636), 208–211. https://doi.org/10.1038/nature20806

Coates, Michael I., & Sequeira, S. E. K. (2001). Early sharks and primitive gnathostome interrelationships. In *Major Events in Early Vertebrate Evolution* (pp. 241–262). Taylor and Francis.

Coates, Michael I., & Tietjen, K. (2018). The neurocranium of the Lower Carboniferous shark *Tristychius arcuatus* (Agassiz,). *Earth and Environmental Science Transactions of the Royal Society of Edinburgh*, *108*(1), 19–35.

Coates, Michael I., Tietjen, K., Johanson, Z., Friedman, M., & Sang, S. (2021). The cranium of *Helodus simplex* (Agassiz, 1838) revised. In *Ancient Fishes and their Living Relatives. A Tribute to John G. Maisey* (pp. 193–204). München: Verlag Dr. Friedrich Pfeil.

Coates, Michael I., Tietjen, K., Olsen, A. M., & Finarelli, J. A. (2019). High-performance suction feeding in an early elasmobranch. *Science Advances*, *5*(9), eaax2742. https://doi.org/10.1126/sciadv.aax2742

Cole, F. J. (1897). XIX.— *On the Cranial Nerves of* Chimæra monstrosa (Linn. 1754); *with a Discussion of the Lateral Line System, and of the Morphology of the* Chorda tympani. *Transactions of the Royal Society of Edinburgh*, *38*(3), 631–680. https://doi.org/10.1017/S0080456800033433

Compagno, L. J. V. (1999). Checklist of living elasmobranchs. In *Sharks, Skates, and Rays, the Biology of Elasmobranch Fishes* (pp. 69–92). Johns Hopkins University.

Davis, J. C. (2002). *Statistics and data analysis in geology* (3rd ed.). New York: J. Wiley.

Davis, S. P., Finarelli, J. A., & Coates, M. I. (2012). *Acanthodes* and shark-like conditions in the last common ancestor of modern gnathostomes. *Nature*, *486*(7402), 247–250. https://doi.org/10.1038/nature11080

De Beer, G. R., & Moy-Thomas, J. A. (1935). V I.—On the skull of Holocephali. *Philosophical Transactions of the Royal Society of London. Series B, Biological Sciences*, *224*(514), 287–312. https://doi.org/10.1098/rstb.1935.0001

Dean, B. (1909). Studies on fossil fishes (sharks, chimaeroids and arthrodires). *Memoirs of the AMNH*, *9*(5), 209–287.

Dean, M. N., Mull, C. G., Gorb, S. N., & Summers, A. P. (2009). Ontogeny of the tessellated skeleton: insight from the skeletal growth of the round stingray *Urobatis halleri*. *Journal of Anatomy*, *215*(3), 227–239. https://doi.org/10.1111/j.1469-7580.2009.01116.x

Dean, M. N., & Summers, A. P. (2006). Mineralized cartilage in the skeleton of chondrichthyan fishes. *Zoology*, *109*(2), 164–168. https://doi.org/10.1016/j.zool.2006.03.002

Dearden, R. P., Stockey, C., & Brazeau, M. D. (2019). The pharynx of the stem-chondrichthyan *Ptomacanthus* and the early evolution of the gnathostome gill skeleton. *Nature Communications*, *10*(1), 2050. https://doi.org/10.1038/s41467-019-10032-3

DeBeer, G. R. (1937). *The Development of the Vertebrate Skull*. University of Chicago.

Denison, R. H. (1979). *Acanthodii*. Stuttgart; New York: G. Fischer.

Dennis, K., & Miles, R. S. (1981). A pachyosteomorph arthrodire from Gogo, Western Australia. *Zoological Journal of the Linnean Society*, *73*(3), 213–258. https://doi.org/10.1111/j.1096-3642.1981.tb01594.x

Dick, J. R. F. (1978). On the Carboniferous shark *Tristychius arcuatus* Agassiz from Scotland. *Transactions of the Royal Society of Edinburgh*, *70*(4), 63–108. https://doi.org/10.1017/S0080456800012898

Dick, J. R. F. (1981). *Diplodoselache woodi* gen. et sp. nov., an early Carboniferous shark from the Midland Valley of Scotland. *Transactions of the Royal Society of Edinburgh: Earth Sciences*, *72*(2), 99–113. https://doi.org/10.1017/S0263593300009937

Dick, J. R. F., & Maisey, J. G. (1980). The Scottish Lower Carboniferous shark *Onychoselache traquairi*. *Palaeontology*, *22*, 363–374.

Didier, D. A. (1995). Phylogenetic systematics of extant chimaeroid fishes (Holocephali, Chimaeroidei), *3119*, 1–86.

Didier, D. A., Kemper, J. M., & Ebert, D. A. (2012). Phylogeny, Biology, and Classification of Extant Holocephalans. In *Biology of Sharks and their Relatives* (pp. 97–124). Boca Raton: CRC Press.

Didier, D. A., Stahl, B. J., & Zangerl, R. (1994). Development and growth of compound tooth plates in *Callorhinchus milii* (chondrichthyes, holocephali). *Journal of Morphology*, *222*(1), 73–89. https://doi.org/10.1002/jmor.1052220108

Drummond, A. J., & Rambaut, A. (2007). BEAST: Bayesian evolutionary analysis by sampling trees. *BMC Evolutionary Biology*, *7*(1), 214. https://doi.org/10.1186/1471-2148-7-214

Drummond, A. J., Suchard, M. A., Xie, D., & Rambaut, A. (2012). Bayesian Phylogenetics with BEAUti and the BEAST 1.7. *Molecular Biology and Evolution*, *29*(8), 1969–1973. https://doi.org/10.1093/molbev/mss075

Dupret, V., Sanchez, S., Goujet, D., Tafforeau, P., & Ahlberg, P. E. (2014). A primitive placoderm sheds light on the origin of the jawed vertebrate face. *Nature*, *507*(7493), 500–503. https://doi.org/10.1038/nature12980

Elder, R. L., & Smith, G. R. (1988). Fish taphonomy and environmental inference in paleolimnology. *Palaeogeography, Palaeoclimatology, Palaeoecology*, *62*, 577–592.

Finarelli, J. A., & Coates, M. I. (2012). First tooth-set outside the jaws in a vertebrate. *Proceedings of the Royal Society B: Biological Sciences*, *279*(1729), 775–779. https://doi.org/10.1098/rspb.2011.1107

Finarelli, J. A., & Coates, M. I. (2014). *Chondrenchelys problematica* (Traquair, 1888) redescribed: a Lower Carboniferous, eel-like holocephalan from Scotland. *Earth and*

Environmental Science Transactions of the Royal Society of Edinburgh, *105*(1), 35–59. https://doi.org/10.1017/S1755691014000139

Forey, P. L. (1980). *Latimeria*: a paradoxical fish. *Proceedings of the Royal Society of London. Series B, Biological Sciences*, *208*, 369–384.

Frey, L., Coates, M., Ginter, M., Hairapetian, V., Rücklin, M., Jerjen, I., & Klug, C. (2019). The early elasmobranch *Phoebodus*: phylogenetic relationships, ecomorphology and a new time-scale for shark evolution. *Proceedings of the Royal Society B: Biological Sciences*, *286*(1912), 20191336. https://doi.org/10.1098/rspb.2019.1336

Frey, L., Coates, M. I., Tietjen, K., Rücklin, M., & Klug, C. (2020). A symmoriiform from the Late Devonian of Morocco demonstrates a derived jaw function in ancient chondrichthyans. *Communications Biology*, *3*(1), 681. https://doi.org/10.1038/s42003-020-01394-2

Frey, L., Pohle, A., Rücklin, M., & Klug, C. (2020). Fossil-Lagerstätten, palaeoecology and preservation of invertebrates and vertebrates from the Devonian in the eastern Anti-Atlas, Morocco. *Lethaia*, *53*(2), 242–266. https://doi.org/10.1111/let.12354

Frey, L., Rücklin, M., Korn, D., & Klug, C. (2018). Late Devonian and Early Carboniferous alpha diversity, ecospace occupation, vertebrate assemblages and bio-events of southeastern Morocco. *Palaeogeography, Palaeoclimatology, Palaeoecology*, *496*, 1–17. https://doi.org/10.1016/j.palaeo.2017.12.028

Friedman, M. (2007). *Styloichthys* as the oldest coelacanth: Implications for early osteichthyan interrelationships. *Journal of Systematic Palaeontology*, *5*(3), 289–343. https://doi.org/10.1017/S1477201907002052

Friedman, M., & Brazeau, M. D. (2010). A reappraisal of the origin and basal radiation of the Osteichthyes. *Journal of Vertebrate Paleontology*, *30*(1), 36–56. https://doi.org/10.1080/02724630903409071

Gagnier, P.-Y. (1996). Acanthodii. In *Devonian Fishes and Plants of Miguasha, Quebec, Canada* (pp. 149–163). München: Verlag Dr. Friedrich Pfeil.

Gagnier, P.-Y., Hanke, G. F., & Wilson, M. V. H. (1999). *Tetanopsyrus lindoei* gen. et sp. nov., an Early Devonian acanthodian from the Northwest Territories, Canada. *Acta Geologica Polonica*, *49*, 81–96.

Gagnier, P.-Y., & Wilson, M. V. H. (1995). New evidences on jaw bones and jaw articulations in acanthodians. *Geobios*, *28*, 137–143. https://doi.org/10.1016/S0016-6995(95)80101-4

Gagnier, P.-Y., & Wilson, M. V. H. (1996a). An unusual acanthodian from Northern Canada: revision of *Brochoadmones milesi*. *Modern Geology*, *20*, 235–251.

Gagnier, P.-Y., & Wilson, M. V. H. (1996b). Early Devonian acanthodians from northern Canada. *Palaeontology*, *39*, 241–258.

Gans, C., & Parsons, T. S. (1981). *A photographic atlas of shark anatomy: the gross morphology of* Squalus acanthias (University of Chicago Press ed.). Chicago: University of Chicago Press.

Gardiner, B. G. (1984). The relationships of the palaeoniscoid fishes, a review based on new specimens of *Mimia* and *Moythomasia* from the Upper Devonian of Western Australia, *37*, 173–428.

Gardiner, B. G., & Bartram, A. W. H. (1977). The homologies of ventral cranial fissures in osteichthyans. In *Problems in Vertebrate Evolution* (pp. 227–245).

Gavryushkina, A., Welch, D., Stadler, T., & Drummond, A. J. (2014). Bayesian inference of sampled ancestor trees for epidemiology and fossil calibration. *PLoS Computational Biology*, *10*(12), e1003919. https://doi.org/10.1371/journal.pcbi.1003919

Giles, S., Coates, M. I., Garwood, R. J., Brazeau, M. D., Atwood, R., Johanson, Z., & Friedman, M. (2015). Endoskeletal structure in *C heirolepis* (Osteichthyes, Actinopterygii), an early ray-finned fish. *Palaeontology*, *58*(5), 849–870. https://doi.org/10.1111/pala.12182

Giles, S., & Friedman, M. (2014). Virtual reconstruction of endocast anatomy in early ray-finned fishes (Osteichthyes, Actinopterygii). *Journal of Paleontology*, *88*(4), 636–651. https://doi.org/10.1666/13-094

Giles, S., Friedman, M., & Brazeau, M. D. (2015). Osteichthyan-like cranial conditions in an Early Devonian stem gnathostome. *Nature*, *520*(7545), 82–85. https://doi.org/10.1038/nature14065

Ginter, Michal, Hairapetian, V., & Klug, C. (2002). Famennian chondrichthyans from the shelves of North Gondwana. *Acta Geologica Polonica*, *52*, 169–215.

Ginter, Michał, Hampe, O., & Duffin, C. J. (2010). *Handbook of paleoichthyology: teeth*. München: F. Pfeil.

Goodrich, E. S. (1930). *Studies on the structure & development of vertebrates*. London: Macmillan. https://doi.org/10.5962/bhl.title.82144

Goujet, D., & Young, G. C. (2004). Placoderm anatomy and phylogeny: new insights. In *Recent Advances in the Origin and Early Radiation of Vertebrates* (pp. 109–126). München: Verlag Dr. Friedrich Pfeil.

Grogan, E. D., & Lund, R. (2000). Debeerius ellefseni (Fam. Nov., Gen. Nov., Spec. Nov.), an autodiastylic chondrichthyan from the Mississippian Bear Gulch Limestone of Montana

(USA), the relationships of the chondrichthyes, and comments on gnathostome evolution. *Journal of Morphology*, *243*(3), 219–245. https://doi.org/10.1002/(SICI)1097-4687(200003)243:3<219::AID-JMOR1>3.0.CO;2-1

Grogan, E. D., & Lund, R. (2008). A basal elasmobranch, *Thrinacoselache gracia* n. gen and sp., (Thrinacodontidae, new family) from the Bear Gulch Limestone, Serpukhovian of Montana, USA. *Journal of Vertebrate Paleontology*, *28*(4), 970–988. https://doi.org/10.1671/0272-4634-28.4.970

Gross, W. (1937). Das Kopfskelett on *Cladodus wildungensis* Jaekel; 1. Teil. Endocranium und Palatoquadratum. *Senckenbergiana*, *19*, 80–107.

Gross, W. (1938). Das Kopfskelett on *Cladodus wildungensis* Jaekel; 2. Teil. Der Kieferbogen. Anhang: Protractodus vetusus Jaekel. *Senckenbergiana*, *20*, 123–145.

Hampe, O. (2002). Revision of the Xenacanthida (Chondrichthyes: Elasmobranchii) from the Carboniferous of the British Isles. *Transactions of the Royal Society of Edinburgh: Earth Sciences*, *93*(3), 191–237. https://doi.org/10.1017/S0263593300000419

Hanke, G. F., & Davis, S. P. (2008). Redescription of the acanthodian *Gladiobranchus probaton* Bernacsek & Dineley, 1977, and comments on diplacanthid relationships. *Geodiversitas*, *30*, 303–330.

Hanke, G. F., & Davis, S. P. (2012). A re-examination of *Lupopsyrus pygmaeus* Bernacsek & Dineley, 1977 (Pisces, Acanthodii). *Geodiversitas*, *34*(3), 469–487. https://doi.org/10.5252/g2012n3a1

Hanke, G. F., Davis, S. P., & Wilson, M. V. H. (2001). New species of the acanthodian genus Tetanopsyrus from northern Canada, and comments on related taxa. *Journal of Vertebrate Paleontology*, *21*(4), 740–753.

Hanke, G. F., & Wilson, M. V. H. (2004). The putative stem-group chondrichthyans *Kathemacanthus* and *Seretolepis* from the Lower Devonian MOTH locality, Mackenzie Mountains, Canada (pp. 189–216). München: Verlag Dr. Friedrich Pfeil.

Hanke, G. F., & Wilson, M. V. H. (2006). Anatomy of the Early Devonian Acanthodian *Brochoadmones milesi* based on nearly complete body fossils, with comments on the evolution and development of paired fins. *Journal of Vertebrate Paleontology*, *26,* 526–537.

Harris, J. E. (1938a). The dorsal fin spine of *Cladoselache*. *Scientific Publications of the Cleveland Museum of Natural History*, *8*, 7–12.

Harris, J. E. (1938b). The neurocranium and jaws of *Cladoselache*. *Scientific Publications of the Cleveland Museum of Natural History*, *8*, 7–12.

Heath, T. A., Huelsenbeck, J. P., & Stadler, T. (2014). The fossilized birth–death process for coherent calibration of divergence-time estimates. *Proceedings of the National Academy of Sciences*, *111*(29). https://doi.org/10.1073/pnas.1319091111

Heidtke, U. (1993). Studien über Acanthodes. 4. *Acanthodes boyi* n. sp., die dritte Art der Acanthodier (Acanthodii: Pisces) aus dem Rotliegend (Unterperm) des Saar-Nahe-Beckens (SW-Deutschland). *Paläontologische Zeitschrift*, *67*(3–4), 331–341. https://doi.org/10.1007/BF02990286

Heidtke, U. H. J. (1982). Der Xenacanthide *Orthacanthus senckenbergianus* aus dem pfälzischen Rotliegenden (Unter-Perm). *Pollichia*, *70,* 65–86.

Heidtke, U. H. J. (1999). *Orthacanthus* (*Lebachacanthus*) *senckenbergianus* Fritsch 1889 (Xenacanthida: Chondrichthyes): Revision, Organisation und Phylogenie. *Freiberger Forschungsheft*, *481,* 63–106.

Heidtke, U. H. J. (2009). *Gladbachus adentatus*, die Geschichte des weltweit ältesten Hais – untersucht und beschrieben aus dem AK Geowissenschaften. *Pollichia Kurrier*, *25*, 24–26.

Heidtke, U. H. J. (2011a). Neue Erkenntnisse über *Acanthodes bronni* Agassiz 1833, *95*, 1–14.

Heidtke, U. H. J. (2011b). Revision der unterpermischen Acanthodier (Acanthodii: Pisces) des südwestdeutschen Saar-Nahe-Beckens, *95*, 15–41.

Heidtke, U. H. J., & Krätschmer, K. (2001). *Gladbachus adentatus* nov. gen. et sp., ein primitiver Hai aus dem Oberen Givetium (Oberes Mitteldevon) der Bergisch Gladbach – Paffrath-Mulde (Rheinisches Schiefergebirge), *30*, 105–122.

Heidtke, U. H. J., Schwind, C., & Krätschmer, K. (2004). Über die Organisation des Skelettes und die verwandtschaftlichen Beziehungen der Gattung *Triodus* Jordan 1849 (Elasmobranchii: Xenacanthida), *32*, 9–54.

Hodnett, J.-P. M., Grogan, E., Lund, R., Lucas, S. G., Suazo, T., Elliott, D. K., & Pruitt, J. (2021). Ctenacanthiform sharks from the Late Pennsylvanian (Missourian) Tinajas member of the Atrasado Formation, Central New Mexico. In *Kinney Brick Quarry Lagerstätte* (pp. 391–424). New Mexico Museum of Natural History and Science.

Hotton, N. (1952). Jaws and teeth of American xenacanth sharks. *Journal of Paleontology*, *26*, 489–500.

Howard, L. E., Holmes, W. M., Ferrando, S., Maclaine, J. S., Kelsh, R. N., Ramsey, A., et al. (2013). Functional nasal morphology of chimaerid fishes: Chimaerid Nasal Anatomy. *Journal of Morphology*, *274*(9), 987–1009. https://doi.org/10.1002/jmor.20156

Janvier, P. (2002). *Early vertebrates* (Repr.). Oxford: Clarendon Press.

Janvier, P., & Maisey, J. G. (2010). The Devonian vertebrates of South America and their biogeographical relationships. In *Morphology, Phylogeny and Paleobiogeography of Fossil Fishes* (pp. 431–459). München: Verlag Dr. Friedrich Pfeil.

Jarvik, E. (1977). Problems in Vertebrate Evolution. In *Problems in Vertebrate Evolution* (pp. 199–225). London: Linnean Symposium Series.

Jarvik, E. (1980). *Basic structure and evolution of vertebrates. 2*. London: Academic Pr.

Jobbins, M., Haug, C., & Klug, C. (2020). First African thylacocephalans from the Famennian of Morocco and their role in Late Devonian food webs. *Scientific Reports*, *10*(1), 5129. https://doi.org/10.1038/s41598-020-61770-0

Johanson, Z., Underwood, C. J., Coates, M. I., Fernandez, V., Clark, B., & Smith, M. M. (2021). The stem-holocephalan *Helodus* (Chondrichthyes; Holocephali) and the evolution of modern chimaeroid dentitions. In *John Maisey symposium* (pp. 205–214). München: Verlag Dr. Friedrich Pfeil.

Kesteven, H. L. (1933). The Anatomy of the Head of *Callorhynchus antarcticus*. *Journal of Anatomy*, *67*(Pt 3), 443-474.3.

King, B., Qiao, T., Lee, M. S. Y., Zhu, M., & Long, J. A. (2016). Bayesian Morphological Clock Methods Resurrect Placoderm Monophyly and Reveal Rapid Early Evolution in Jawed Vertebrates. *Systematic Biology*, syw107. https://doi.org/10.1093/sysbio/syw107

Lane, J. A. (2010). Morphology of the braincase in the Cretaceous hybodont shark *Tribodus limae* (Chondrichthyes: Elasmobranchii), based on CT scanning, *2758*, 1–70.

Lane, J. A., & Maisey, J. G. (2009). Pectoral anatomy of *Tribodus limae* (Elasmobranchii: Hybodontiformes) from the Lower Cretaceous of Northeastern Brazil. *Journal of Vertebrate Paleontology*, *29*, 25–38.

Lane, J. A., & Maisey, J. G. (2012). The visceral skeleton and jaw suspension in the durophagous hybodontid shark *Tribodus limae* from the Lower Cretaceous of Brazil. *Journal of Paleontology*, *86*, 886–905.

Liem, K. F., & Summers, A. P. (1999). Muscular system: microscopical anatomy, physiology and biochemistry of Elasmobranch muscle fibers. In *Sharks, skates, and rays : the biology of elasmobranch fishes* (pp. 93–114). Baltimore: John Hopkins University Press.

Long, J. A. (1983). A new diplacanthoid acanthodian from the Late Devonian of Victoria. *Memoirs of the Association of Australasian Palaeontologists*, *1*, 51–65.

Long, J. A., Burrow, C. J., Ginter, M., Maisey, J. G., Trinajstic, K. M., Coates, M. I., et al. (2015). First Shark from the Late Devonian (Frasnian) Gogo Formation, Western Australia Sheds New Light on the Development of Tessellated Calcified Cartilage. *PLOS ONE*, *10*(5), e0126066. https://doi.org/10.1371/journal.pone.0126066

Lu, J., Giles, S., Friedman, M., den Blaauwen, J. L., & Zhu, M. (2016). The Oldest Actinopterygian Highlights the Cryptic Early History of the Hyperdiverse Ray-Finned Fishes. *Current Biology*, *26*(12), 1602–1608. https://doi.org/10.1016/j.cub.2016.04.045

Lu, J., Giles, S., Friedman, M., & Zhu, M. (2017). A new stem sarcopterygian illuminates patterns of character evolution in early bony fishes. *Nature Communications*, *8*(1), 1932. https://doi.org/10.1038/s41467-017-01801-z

Lund, R. (1982). *Harpagofututor volsellorhinus* new genus and species (Chondrichthyes, Chondrenchelyiformes) from the Namurian Bear Gulch Limestone, *Chondrenchelys problematica* and their sexual dimorphism. *Journal of Paleontology*, *56*, 938–958.

Lund, R. (1985). The Morphology of *Falcatus falcatus* (St. John and Worthen), a Mississippian Stethacanthid Chondrichthyan from the Bear Gulch Limestone of Montana. *Journal of Vertebrate Paleontology*, *5*(1), 1–19.

Lund, R. (1986). On *Damocles serratus* nov. gen. et sp., (Elasmobranchii: Cladodontida) from the Upper Mississippian Bear Gulch Limestone of Montana. *Journal of Vertebrate Paleontology*, *6*, 12–19.

Lund, R., & Grogan, E. (2004a). The Origin and Relationships of Early Chondrichthyes. In J. Musick, J. Carrier, & M. Heithaus (Eds.), *Biology of Sharks and Their Relatives* (Vol. 20043354, pp. 3–31). CRC Press. https://doi.org/10.1201/9780203491317.pt1

Lund, R., & Grogan, E. D. (1997). Relationships of the Chimaeriformes and the basal radiation of the Chondrichthyes. *Reviews in Fish Biology and Fisheries*, *7*, 65–123.

Lund, R., & Grogan, E. D. (2004b). Five new euchondrocephalan Chondrichthyes from the Bear Gulch Limestone (Serpukhovian, Namurian E2b) of Montana, USA. In *Recent Advances in the Origin and Early Radiation of Vertebrates* (pp. 505–531). München: Verlag Dr. Friedrich Pfeil.

Maisey, J., Miller, R., & Turner, S. (2009). The braincase of the chondrichthyan *Doliodus* from the Lower Devonian Campbellton Formation of New Brunswick, Canada. *Acta Zoologica*, *90*, 109–122. https://doi.org/10.1111/j.1463-6395.2008.00330.x

Maisey, John G. (1984). Chondrichthyan phylogeny: a look at the evidence. *Journal of Vertebrate Paleontology*, *4*(3), 359–371. https://doi.org/10.1080/02724634.1984.10012015

Maisey, John G. (1989). Visceral skeleton and musculature of a Late Devonian shark. *Journal of Vertebrate Paleontology*, *9*, 174–190.

Maisey, John G. (2005). Braincase of the Upper Devonian shark *Cladodoides wildungensis* (Chondrichthyes, Elasmobranchii), with observations on the braincase in early chondrichthyans, *288*, 1–103.

Maisey, John G. (2007). The braincase in Paleozoic symmoriiform and cladoselachian sharks, *307*, 1–122.

Maisey, John G., & de Carvalho, M. R. (1997). A new look at old sharks. *Nature*, *385*(6619), 779–780. https://doi.org/10.1038/385779a0

Maisey, John G., Miller, R., Pradel, A., Denton, J. S. S., Bronson, A., & Janvier, P. (2017). Pectoral Morphology in *Doliodus*: Bridging the 'Acanthodian'-Chondrichthyan Divide. *American Museum Novitates, 3875*(3875), 1–15. https://doi.org/10.1206/3875.1

Maisey, John G., Turner, S., Naylor, G. J. P., & Miller, R. F. (2013). Dental patterning in the earliest sharks: Implications for tooth evolution: DENTAL PATTERNING IN EARLY SHARKS. *Journal of Morphology*, n/a-n/a. https://doi.org/10.1002/jmor.20242

Maisey, J. G.. (1982). The anatomy and interrelationships of Mesozoic hybodont sharks, *2724*, 1–48.

Maisey, J. G.. (1983). Cranial anatomy of *Hybodus basanus* Egerton from the Lower Cretaceous of England. *American Museum Novitates, 2758,* 1–64.

Maisey, J. G.. (1985). Cranial Morphology of the Fossil Elasmobranch *Synechodus dubrisiensis*. *American Museum Novitates, 2804,* 1–28.

Maisey, J. G.. (2001). A Primitive Chondrichthyan Braincase from the Middle Devonian of Bolivia. In *Major Events in Early Vertebrate Evolution* (pp. 263–288). Taylor & Francis.

Maisey, J. G.. (2011). The braincase of the Middle Triassic shark *Acronemus tuberculatus* (Bassani, 1886). *Palaeontology, 54,* 417–428.

Maisey, J. G.. (2013). The diversity of tessellated calcification in modern and extinct chondrichthyans. *Revue de Paléobiologie, Genève*, *32*, 355–371.

Maisey, J. G., & Anderson, M. E. (2001). A primitive chondrichthyan braincase from the Early Devonian of South Africa. *Journal of Vertebrate Paleontology*, *21*, 702–713.

Maisey, J. G., Janvier, P., Pradel, A., Denton, J. S. S., Bronson, A., Miller, R. F., & Burrow, C. J. (2019). *Doliodus* and pucapampellids. In *Evolution and Development of Fishes* (pp. 87–109). Cambridge.

Maisey, J. G., & Lane, J. A. (2010). Labyrinth morphology and the evolution of low-frequency phonoreception in elasmobranchs. *Comptes Rendus Palevol*, *9*, 289–309.

Marinelli, W., & Strenger, A. (1959). *Vergleichende Anatomie und Morphologie der Wirbeltiere. vol. 3, Squalus acanthias*. Wien: F. Deuticke.

Miles, R. S. (1971). Acanthodii. In *Encyl. Sci. Tech.* (pp. 33–34). McGraw-Hill.

Miles, R. S. (1973a). Articulated acanthodian fishes from the Old Red Sandstone of England, with a review of the structure and evolution of the acanthodian shoulder-girdle, *24*, 111–213.

Miles, R. S. (1973b). Acanthodii. In *Interrelationships of Fishes* (pp. 63–103).

Miller, R. F., Cloutier, R., & Turner, S. (2003). The oldest articulated chondrichthyan from the Early Devonian period. *Nature*, *425*(6957), 501–504. https://doi.org/10.1038/nature02001

Moy-Thomas, J. A. (1935). The structure and affinities of Chondrenchelys problematica Tr. *Proceedings of the Zoological Society, London*, *1935*, 391–403.

Mutter, R. J., de Blanger, K., & Neuman, A. G. (2007). Elasmobranchs from the Lower Triassic Sulphur Mountain Formation near Wapiti Lake (BC, Canada). *Zoological Journal of the Linnean Society*, *149*, 309–337.

Mutter, R. J., Neuman, A. G., & de Blanger, K. (2008). *Homalodontus* nom. nov. a replacement for *Wapitiodus* Mutter, de Blanger and Neuman 2007 (Homalodontidae nom. nov., ?Hybodontoidea), preoccupied by *Wapitiodus* Orchard, 2005. *Zoological Journal of the Linnean Society*, *154*, 419–420.

Nelson, G. J. (1968). Gill-arch structure in *Acanthodes*. In *Gill-arch structure in Acanthodes*. Stockholm: Almqvist & Wiksell.

Newman, M. J., Davidson, R. G., Blaauwen, J. L. D., & Burrow, C. J. (2012). The Early Devonian Acanthodian *Uraniacanthus curtus* (Powrie, 1870) n. comb. from the Midland Valley of Scotland. *Geodiversitas*, *34*(4), 739–759. https://doi.org/10.5252/g2012n4a2

Oelofsen, B. W. (1986). A fossil shark Neurocranium from the Permo-Carboniferous (lowermost Ecca Formation) of South Africa. In *Indo-Pacific Fish Biology: Proceedings of the Second International Conference on Indo-Pacific Fishes* (pp. 107–124). Japan: Ichthyological Society of Japan.

Patterson, C. (1965). The phylogeny of the chimaeroids. *Philosophical Transactions of the Royal Society of London. Series B, Biological Sciences*, *249*(757), 101–219. https://doi.org/10.1098/rstb.1965.0010

Patterson, C. (1982). Morphology and Interrelationships of Primitive Actinopterygian Fishes. *American Zoologist*, *22*(2), 241–259. https://doi.org/10.1093/icb/22.2.241

Pearson, D. M., & Westoll, T. S. (1979). The Devonian actinopterygian *Cheirolepis* Agassiz. *Earth and Environmental Science Transactions of the Royal Society of Edinburgh*, *70*(13–14), 337–399. https://doi.org/10.1017/S0080456800012850

Pradel, A., Didier, D., Casane, D., Tafforeau, P., & Maisey, J. G. (2013). Holocephalan embryo provides new information on the evolution of the glossopharyngeal nerve, metotic fissure

and parachordal plate in gnathostomes. *PLoS ONE*, *8*(6), e66988. https://doi.org/10.1371/journal.pone.0066988

Pradel, A., Maisey, J. G., Tafforeau, P., & Janvier, P. (2009). An enigmatic gnathostome vertebrate skull from the Middle Devonian of Bolivia. *Acta Zoologica*, *90*, 123–133. https://doi.org/10.1111/j.1463-6395.2008.00350.x

Pradel, A., Maisey, J. G., Tafforeau, P., Mapes, R. H., & Mallatt, J. (2014). A Palaeozoic shark with osteichthyan-like branchial arches. *Nature*, *509*(7502), 608–611. https://doi.org/10.1038/nature13195

Pradel, A., Tafforeau, P., & Janvier, P. (2010). Study of the pectoral girdle and fins of the Late Carboniferous sibyrhynchid iniopterygians (Vertebrata, Chondrichthyes, Iniopterygia) from Kansas and Oklahoma (USA) by means of microtomography, with comments on iniopterygian relationships. *Comptes Rendus Palevol*, *9*(6–7), 377–387. https://doi.org/10.1016/j.crpv.2010.07.015

Pradel, A., Tafforeau, P., Maisey, J. G., & Janvier, P. (2011). A New Paleozoic Symmoriiformes (Chondrichthyes) from the Late Carboniferous of Kansas (USA) and Cladistic Analysis of Early Chondrichthyans. *PLoS ONE*, *6*(9), e24938. https://doi.org/10.1371/journal.pone.0024938

Qiao, T., King, B., Long, J. A., Ahlberg, P. E., & Zhu, M. (2016). Early Gnathostome Phylogeny Revisited: Multiple Method Consensus. *PLOS ONE*, *11*(9), e0163157. https://doi.org/10.1371/journal.pone.0163157

Qu, Q., Sanchez, S., Blom, H., Tafforeau, P., & Ahlberg, P. E. (2013). Scales and Tooth Whorls of Ancient Fishes Challenge Distinction between External and Oral 'Teeth.' *PLoS ONE*, *8*(8), e71890. https://doi.org/10.1371/journal.pone.0071890

Rambaut, A., Drummond, A. J., Xie, D., Baele, G., & Suchard, M. A. (2018). Posterior Summarization in Bayesian Phylogenetics Using Tracer 1.7. *Systematic Biology*, *67*(5), 901–904. https://doi.org/10.1093/sysbio/syy032

Reisdorf, A. G., Bux, R., Wyler, D., Benecke, M., Klug, C., Maisch, M. W., et al. (2012). Float, explode or sink: postmortem fate of lung-breathing marine vertebrates. *Palaeobiodiversity and Palaeoenvironments*, *92*(1), 67–81. https://doi.org/10.1007/s12549-011-0067-z

Rieppel, O. A. (1982). A new genus of shark from the Middle Triassic of Monte San Giorgio, Switzerland. *Palaeontology*, *25*, 399–412.

Rosen, D. E., Forey, P. L., Gardiner, B. G., & Patterson, C. (n.d.). Lungfishes, tetrapods, paleontology, and plesiomorphy. *Bulletin of the AMNH*, *167*(4), 163–275.

Rücklin, M., Donoghue, P. C. J., Johanson, Z., Trinajstic, K., Marone, F., & Stampanoni, M. (2012). Development of teeth and jaws in the earliest jawed vertebrates. *Nature, 491*(7426), 748–751. https://doi.org/10.1038/nature11555

Schaeffer, B. (1975). Comments on the origin and basic radiation of the gnathostome fishes with particular reference to the feeding mechanism. In *Problémes actuels de paléontologie: evolution des vertébrés* (Vol. 218, pp. 101–109).

Schaeffer, B. (1981). The xenacanth shark neurocranium, with comments on elasmobranch monophyly, *169*, 1–66.

Schultze, H.-P., & Zidek, J. (1982). Ein primitiver Acanthodier (Pisces) aus dem Unterdevon Lettlands. *Paläontologische Zeitschrift*, *56*(1–2), 95–105. https://doi.org/10.1007/BF02988788

Seidel, R., Lyons, K., Blumer, M., Zaslansky, P., Fratzl, P., Weaver, J. C., & Dean, M. N. (2016). Ultrastructural and developmental features of the tessellated endoskeleton of elasmobranchs (sharks and rays). *Journal of Anatomy*, *229*(5), 681–702. https://doi.org/10.1111/joa.12508

Sequeira, S. E. K., & Coates, M. I. (2000). Reassessment of '*Cladodus*' *neilsoni* Traquair: a primitive shark from the Lower Carboniferous of East Kilbridge, Scotland. *Palaeontology, 43*(1), 153–172. https://doi.org/10.1111/1475-4983.00122

Soler-Gijón, R., & Hampe, O. (1998). Evidence of *Triodus* Jordan 1849 (Elasmobranchii: Xenacanthidae) in the Lower Permian of the Autun basin (Muse, France). *Neues Jahrbuch für Geologie und Paläontologie - Monatshefte*, *1998*(6), 335–348. https://doi.org/10.1127/njgpm/1998/1998/335

Stadler, T. (2010). Sampling-through-time in birth–death trees. *Journal of Theoretical Biology*, *267*(3), 396–404. https://doi.org/10.1016/j.jtbi.2010.09.010

Stahl, B. J. (1999). *Holocephali*. München: Pfeil.

Trinajstic, K., Boisvert, C., Long, J., Maksimenko, A., & Johanson, Z. (2015). Pelvic and reproductive structures in placoderms (stem gnathostomes): Pelvic and reproductive structures in placoderms. *Biological Reviews*, *90*(2), 467–501. https://doi.org/10.1111/brv.12118

Turner, S., Burrow, C. J., & Warren, A. (2005). *Gyracanthides hawkinsi* sp. nov. (Acanthodii, Gyracanthidae) from the lower Carboniferous of Queensland, Australia, with a review of gyracanthid taxa. *Palaeontology*, *48*(5), 963–1006. https://doi.org/10.1111/j.1475-4983.2005.00479.x

Warren, A., Currie, B. P., Burrow, C., & Turner, S. (2000). A redescription and reinterpretation of *Gyracanthides murrayi* Woodward 1906 (Acanthodii, Gyracanthidae) from the Lower Carboniferous of the Mansfield Basin, Victoria, Australia. *Journal of Vertebrate Paleontology*, *20*(2), 225–242. https://doi.org/10.1671/0272-4634(2000)020[0225:ARAROG]2.0.CO;2

Watson, D. M. S. (1937). II - The Acanthodian fishes. *Philosophical Transactions of the Royal Society of London. Series B, Biological Sciences*, *228*(549), 49–146. https://doi.org/10.1098/rstb.1937.0009

Williams, M. E. (2001). Tooth retention in cladodont sharks: with a comparison between primitive grasping and swallowing, and modern cutting and gouging feeding mechanisms. *Journal of Vertebrate Paleontology*, *21*(2), 214–226. https://doi.org/10.1671/0272-4634(2001)021[0214:TRICSW]2.0.CO;2

Woodward, A. S., & White, E. I. (1938). XLIII.— *The dermal tubercles of the Upper Devonian shark* , Cladoselache. *Annals and Magazine of Natural History*, *2*(10), 367–368. https://doi.org/10.1080/00222933808526863

Yu, X. (1998). A new porolepiform-like fish, *Psarolepis romeri* , gen. et sp. nov. (Sarcopterygii, Osteichthyes) from the Lower Devonian of Yunnan, China. *Journal of Vertebrate Paleontology*, *18*(2), 261–274. https://doi.org/10.1080/02724634.1998.10011055

Zangerl, R. (1981). *Chondrichthyes I, Paleozoic Elasmobranchii*. Gustav Fischer.

Zangerl, R., & Case, G. R. (1976). *Cobelodus aculeatus* (Cope), an anacanthous shark from Pennsylvanian Black Shales of North America. *Palaeontographica. Abteilung A, Paläozoologie, Stratigraphie, 154,* 107–57.

Zhu, M., Ahlberg, P. E., Pan, Z., Zhu, Y., Qiao, T., Zhao, W., et al. (2016). A Silurian maxillate placoderm illuminates jaw evolution. *Science, 354*(6310), 334–336. https://doi.org/10.1126/science.aah3764

Zhu, M., & Schultze, H.-P. (1997). The oldest sarcopterygian fish. *Lethaia*, *30*, 293–304.

Zhu, M., & Schultze, H.-P. (2001). Interrelationships of basal osteichthyans. In *Major Events in Early Vertebrate Evolution (Palaeontology, phylogeny, genetics, and development)* (pp. 289–314). Taylor and Francis.

Zhu, M., Wang, W., & Yu, X. (2010). *Meemannia eos*, a basal sarcopterygian fish from the Lower Devonian of China – expanded description and significance. In *Morphology, Phylogeny and Paleobiogeography of Fossil Fishes* (pp. 199–214). Verlag Dr. Friedrich Pfeil, München.

Zhu, M., & Yu, X. (2002). A primitive fish close to the common ancestor of tetrapods and lungfish. *Nature*, *418*(6899), 767–770. https://doi.org/10.1038/nature00871

Zhu, M., Yu, X., Ahlberg, P. E., Choo, B., Lu, J., Qiao, T., et al. (2013). A Silurian placoderm with osteichthyan-like marginal jaw bones. *Nature*, *502*(7470), 188–193. https://doi.org/10.1038/nature12617

Zhu, M., Yu, X., & Janvier, P. (1999). A primitive fossil fish sheds light on the origin of bony fishes. *Nature, 397*(6720), 607–610. https://doi.org/10.1038/17594

Zhu, M., Zhao, W., Jia, L., Lu, J., Qiao, T., & Qu, Q. (2009). The oldest articulated osteichthyan reveals mosaic gnathostome characters. *Nature*, *458*(7237), 469–474. https://doi.org/10.1038/nature07855

Zidek, J. (1992). Late Pennsylvanian Chondrichthyes, Acanthodii, and deep-bodied Actinopterygii from the Kinney Quarry, Manzanita Moutains, New Mexico. In *Geology*

and paleontology of the Kinney Brick Quarry, Late Pennsylvanian, central New Mexico (pp. 199–214). Socorro: New Mexico Bureau of Mines & Mineral Resources.
